# Microwave-Assisted Buchwald–Hartwig Double Amination: A Rapid and Promising Approach for the Synthesis of TADF Compounds

**DOI:** 10.1021/acsomega.4c07563

**Published:** 2024-12-09

**Authors:** Nor Shafiq Mohd Jamel, Levani Skhirtladze, Aqeel A. Hussein, Yumiao Ma, Kai Lin Woon, Muhammad Kumayl Abdulwahab, Juozas V. Grazulevicius, Azhar Ariffin

**Affiliations:** † Department of Chemistry, Faculty of Science, Universiti Malaya, 50603 Kuala Lumpur, Malaysia; ‡ Department of Science and Technology, Linköping University, Bredgatan 33, Norrkoping SE 601 74, Sweden; § Department of Biology, College of Science, Al-Qasim Green University, 51013 Al-Qasim, Babylon, Iraq; ∥ BSJ Institute, Beijing 100084, People’s Republic of China; ⊥ Beijing Orienda Instrument Co., Ltd., Beijing 102200, People’s Republic of China; # Department of Physics, Faculty of Science, Universiti Malaya, 50603 Kuala Lumpur, Malaysia; ∇ Department of Polymer Chemistry and Technology, Kaunas University of Technology, Baršausko 59, Kaunas 51423, Lithuania

## Abstract

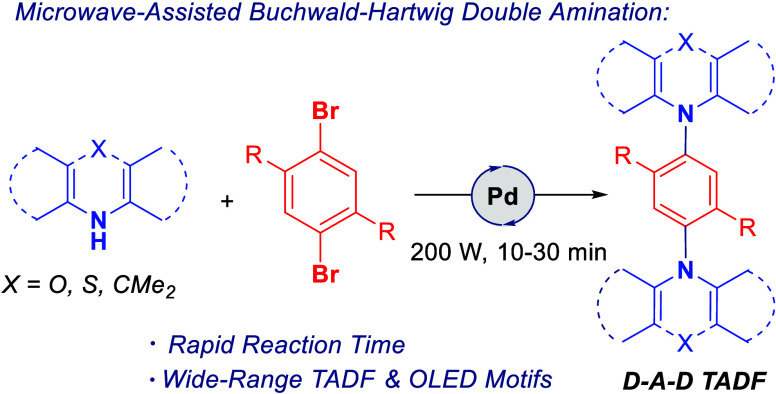

We herein report a microwave-assisted Buchwald–Hartwig double amination reaction to synthesize potential thermally activated delayed fluorescence compounds, forming C(sp^2^)-N bonds between donor and acceptor units. Our approach reduces reaction times from 24 h to 10–30 min and achieves moderate to excellent yields, outperforming conventional heating methods. The method is compatible with various aryl bromides and secondary amines, including phenoxazine, phenothiazine, acridine, and carbazole. Density functional theory calculations have attributed the lack of reactivity with high energy barriers in the reductive elimination (RE) steps. Electron-withdrawing groups such as CF_3_ increase the RE barrier, resulting in a 0% yield, while substituting carbazole with acridine lowers the barriers and enhances higher yields. Distortion–interaction analysis highlights steric hindrance as a key factor affecting the reaction outcome when the RE barrier is low and steric hindrance is minimal. This microwave-assisted method not only demonstrates a superior performance in terms of higher yields and shorter reaction times but also offers significant potential for reducing production costs of these materials.

## 1. Introduction

The field of organic synthesis has seen remarkable advancements in recent years, with microwave-assisted techniques emerging as a transformative approach in organic chemistry. Microwave-assisted organic synthesis is particularly notable for its ability to accelerate reaction times and enhance product yields compared with conventional heating methods. These advantages make it a compelling choice for the synthesis of complex organic molecules, particularly in the realm of advanced optoelectronic materials.

One area where microwave-assisted techniques can show significant promise is in the synthesis of thermally activated delayed fluorescence (TADF) materials for organic light-emitting diodes. Organic light-emitting diodes (OLEDs) are part of an emerging technology that is revolutionizing digital displays.^1^ These molecules are capable of achieving high efficiencies by harvesting both singlet and triplet excitons. To be a good TADF molecule, one of the criteria is to have a vanishingly small energy gap (Δ*E*_ST_) between the first excited singlet state (S_1_) and the first excited triplet state (T_1_), which can be achieved through spatial separation between the highest occupied molecular orbital (HOMO) and the lowest unoccupied molecular orbital (LUMO).^2^ The Δ*E*_ST_ value can be minimized by designing molecules with a twisted C(sp^2^)-N connection between donor (D) and acceptor (A) moieties. Building TADF molecules containing multiple D molecules such as the donor–acceptor–donor (D–A–D) configuration can help to improve the photoluminescence quantum yield (PLQY) and the external quantum efficiency (EQE).^3^

**Figure 1:**
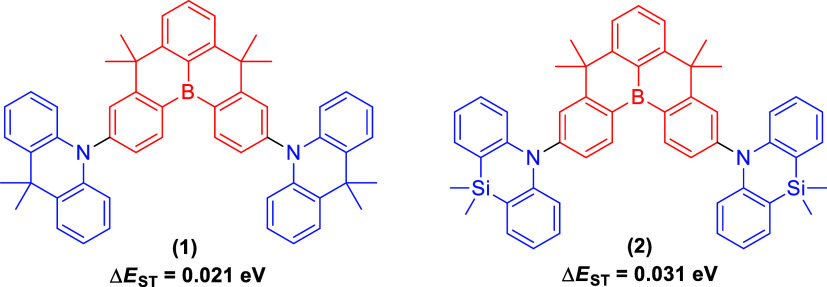
Design of the symmetrical D–A–D configuration.

The symmetrical D–A–D configuration has demonstrated its superiority as a TADF design when compared to the unsymmetrical counterpart.^1^ Compounds (**1**) and (**2**), for example, were designed by Tu et al. (Figure 1) with calculated Δ*E*_ST_ values of 0.021 and 0.031 eV, respectively, and are expected to be suitable for constructing deep-blue D–A–D TADF emitters.^1^

**Figure 2:**
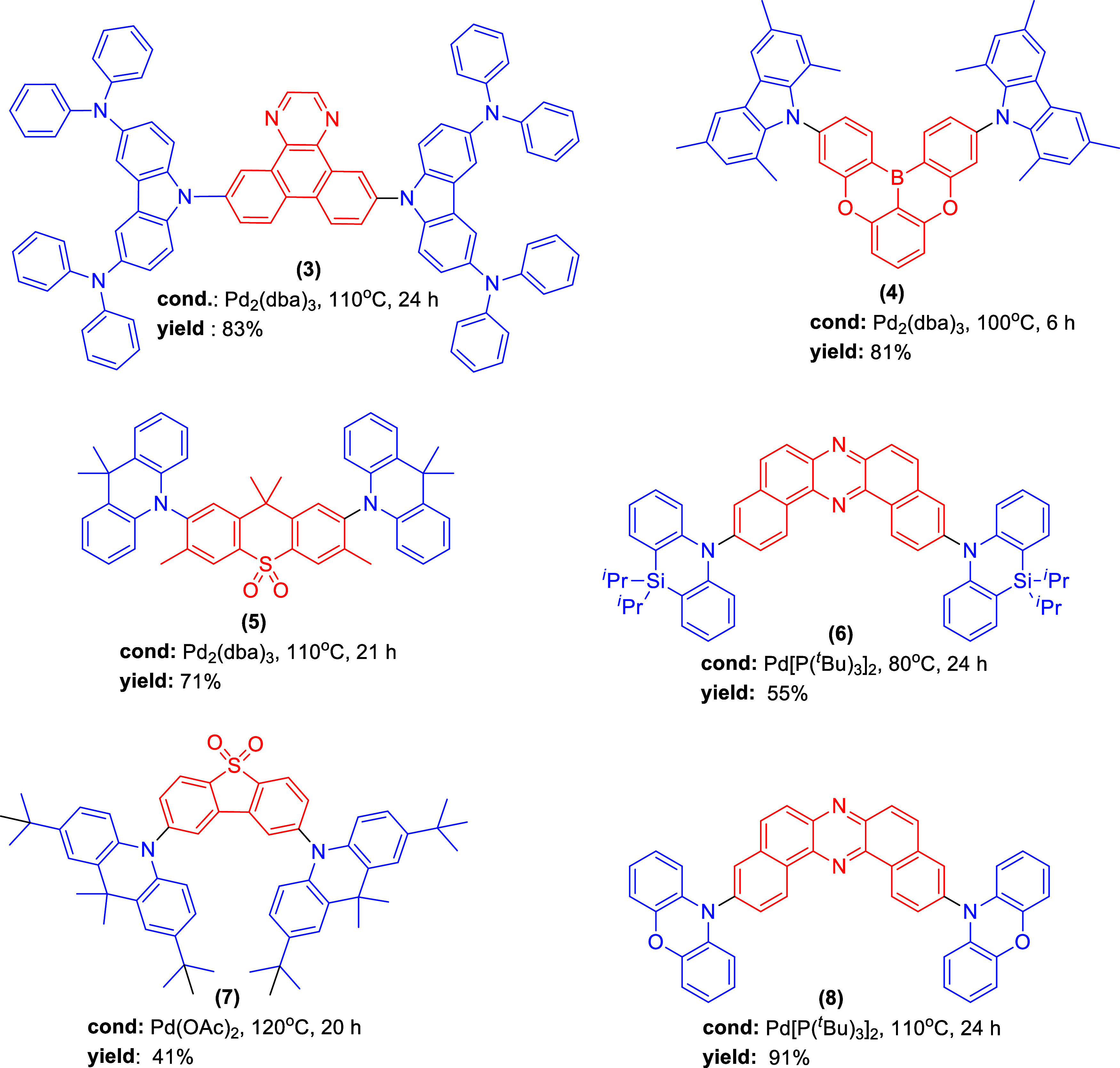
TADF compounds with the D–A–D configuration. Compounds **3** through **8** were previously synthesized through Buchwald–Hartwig 2-fold amination using a conventional heating method.

The symmetrical D–A–D molecules are primarily synthesized through the conventional Buchwald–Hartwig double amination reaction. For instance, compound (**3**), which features a linear D–A–D configuration, exhibits green TADF.^4^ Compound (**4**), characterized by the U-shaped D–A–D configuration, displays pure blue emission,^5^ while compound (**5**) showcases blue TADF emission.^6^ Compound (**6**) is identified as a room-temperature phosphorescence (RTP)-TADF material,^7^ compound (**7**) is also a TADF material,^8^ and compound (**8**) exhibits TADF ranging from green to red.^9^ Compounds (**3**), (**4**), (**5**), (**6**), (**7**), and (**8**) were synthesized using the Buchwald–Hartwig 2-fold amination reaction under conventional heating conditions at temperatures between 80 and 110 °C for periods of 12–24 h (Figure 2).

Microwave-assisted organic synthesis falls under the sixth principle of the 12 principles of green chemistry^10^ and is one of the key developments in organic chemistry. Its effectiveness in accelerating organic reactions has been utilized in several fields of organic synthesis.^11−17^ Since the first time it was introduced in 1986 by Gedye et al. and Giguere et al.,^18,19^ numerous articles have been published in the field of microwave-assisted organic synthesis. The major benefits of microwave synthesis include the increase in product yields, reduction of reaction times, and minimizing unwanted side reactions.^11,20^ The use of microwave synthesis is considered a form of green chemistry.^21^ These advantages have also been exploited in various fields of science, including polymer synthesis,^22^ materials science,^23^ nanotechnology,^24^ and biomedical process.^25^

**Scheme 1:**
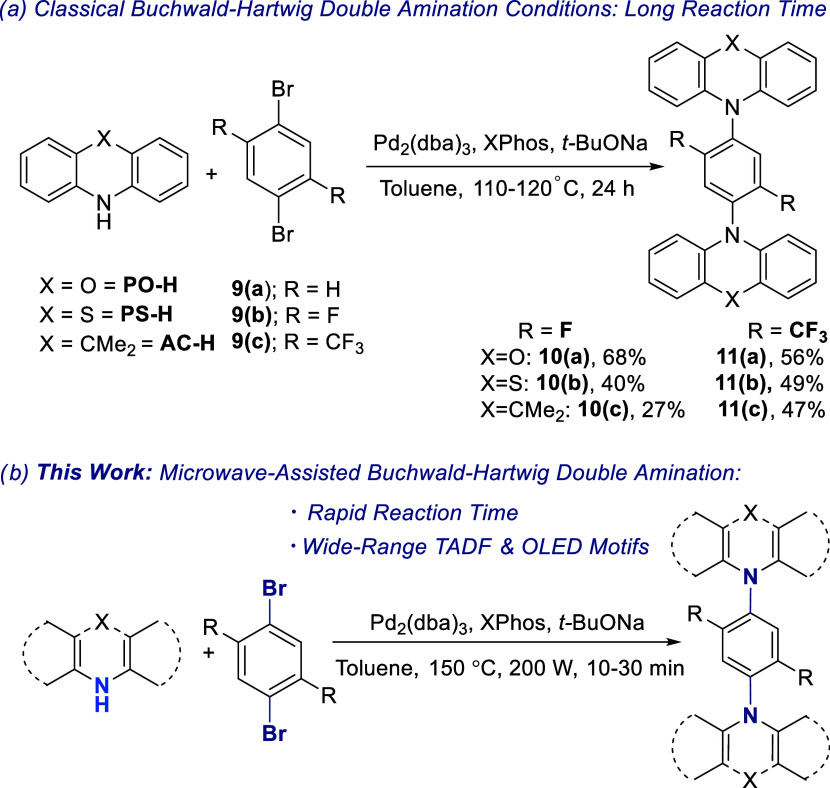
Buchwald–Hartwig Double Amination between Various Secondary Amines and 1,4-Dibromobenzene Derivatives Using the (a) Classical and (b) Microwave Heating Methods

Our interest in the development of D–A–D TADF molecules has prompted us to explore methods that can be utilized for the formation of C(sp^2^)-N bonds between the donor and the acceptor. Carbazole (**Cz-H**) and its derivatives,^3,26−29^ 10*H*-phenoxazine (**PO-H**)^30−32^ and 10*H*-phenothiazine (**PS-H**),^31,33,34^ and derivatives of acridan such as 9,9-dimethyl-10*H*-acridan (**AC-H)**^35,36^ are among the secondary amines normally used as the D molecules, whereas in this study, the 1,4-disubstituted phenylene motif serves as the A unit. Recently, we have reported the synthesis of D–A–D TADF compounds via a 2-fold Buchwald–Hartwig reaction (Scheme 1a). The synthesized compounds were obtained in moderate yields using a combination of Pd_2_(dba)_3_/XPhos as the catalyst, where XPhos refers to the ancillary ligand, sodium *tert*-butoxide (*t*-BuONa) as the base, and the reaction mixtures were refluxed in dry toluene for 24 h.^31^ The list of isolated yields obtained through conventional heating is given in Scheme 1a.

Based on the literature survey, several reports have demonstrated the utility of microwave irradiation onto the Buchwald–Hartwig cross-coupling procedure.^37−42^ For instance, Shaya et al. reported the 2-fold amination of a dibrominated fluorene derivative under microwave conditions.^200^ However, the published method does not utilize a typical donor–acceptor–donor (D–A–D) molecular arrangement characteristic of thermally activated delayed fluorescence (TADF) compounds. Additionally, the amines studied as coupling partners do not reflect those commonly employed in the synthesis of TADF molecules. Thus, to the best of our knowledge, there is no report on the synthesis of D–A–D TADF using the Buchwald–Hartwig 2-fold amination reaction under microwave conditions. Thus, we herein report the impact of microwave energy onto the synthesis of TADF materials via the 2-fold Buchwald–Hartwig cross-coupling protocol following the D–A–D molecular architecture (Scheme 1b). In addition, the lack of reactivity for the 2-fold amination under specific substrates is rationalized through density functional theory (DFT) from a mechanistic point of view.

## 2. Materials and Methods

### 2.1 Materials and Instrumentation

All chemicals were purchased from commercial suppliers and used as received, unless otherwise stated. Industrial-grade *n*-hexane was distilled beforehand. Carbazole was recrystallized from toluene prior to usage. The microwave reaction was carried out in a CEM SP Discover microwave synthesizer and an Anton Paar Monowave 300 microwave reactor. For microwave-assisted reaction, all reactions were carried out in a sealed tube, and the temperature was monitored using an IR temperature sensor, built into the microwave synthesizer. On the other hand, reactions that required conventional heating were carried out in an oil bath with a water-cooled condenser attached to the reaction flask. Flash column chromatography was performed using silica gel (pore size 60 Å, 230–400 mesh particle size, and 40–63 μm particle size). Thin-layer chromatography (TLC) was performed using TLC silica gel 60 F_254_ (aluminum plate). The TLC plates were visualized by using UV light. The detailed synthetic procedure can be found in the Supporting Information (SI).

^1^H, ^13^C, and ^19^F NMR spectra were recorded using either an FT-NMR ECX 400 (JEOL), FT-NMR ECX 500 (JEOL), or FT-NMR BRUKER AVANCE III 400 spectrometer. ^13^C and ^19^F NMR experiments carried out were proton-decoupled. The chemical shift (δ) and coupling constant were recorded in parts per million and Hertz (Hz) units, respectively. The multiplicity of the signals is given as follows; s = singlet, br s = broad singlet, d = doublet, dd = doublet of doublet, t = triplet, td = triplet of doublets, and q = quartet. Mass spectra (MS) were recorded using a Waters ZQ 2000 mass spectrometer. High-resolution mass spectroscopy (HRMS) spectra were recorded using a JMS-T100LP AccuTOF LC-plus mass spectrometer. For both mass measurements, the masses of the compounds were reported as the mass-to-charge ratio (*m*/*z*). Mass measurement was carried out in positive mode with electrospray ionization (ESI) as the ion source. The samples were diluted in the appropriate solvent for mass measurement. For compounds **13(a**–**e)**, **21**, and **22**, the sample solution was spiked with sodium tetrachloroaurate(III) to assist the ionization of the compounds under ESI mode.^43^ The melting points were recorded using a MEL-TEMP II Laboratory Devices melting point apparatus and were not corrected.

### 2.2 Synthesis

#### 2.2.1. General Procedure for Conventional Heating (**PO-H**, **PS-H**, **AC-H**) (GP1)

Aryl bromide (1.0 equiv), secondary amine (2.2 equiv), Pd_2_(dba)_3_ (5 mol %), XPhos (7 mol %), and *t*-BuONa (2.2 equiv) were placed into a reaction flask. Dry toluene (10–30 mL/1.0 g of aryl bromide) was added into the flask. The reaction mass was heated between 110 and 120 °C in an oil bath for 24 h under an argon atmosphere. After 24 h, the reaction mass was cooled down to room temperature before diluting it with dichloromethane (DCM). The organic phase was washed with water and brine, dried over anhydrous Na_2_SO_4_, filtered, and concentrated. The crude material was purified by column chromatography over silica gel, recrystallization, or both.

#### 2.2.2. General Procedure for Microwave Heating (**PO-H**, **PS-H**, **AC-H**) (GP2)

Aryl bromide (1.0 equiv), secondary amine (2.2 equiv), Pd_2_(dba)_3_ (5 mol %), XPhos (7 mol %), *t*-BuONa (2.2 equiv), and dry toluene (20 mL/1.0 g aryl bromide) were weighed into a microwave vial under an argon atmosphere. The reaction mixture was irradiated with a microwave at 150 °C for 10 min or 130 °C for 30 min. The microwave power was set at 200 W. Then, the reaction mass was cooled down to room temperature before diluting it with DCM. The organic phase was washed with water and brine, dried over anhydrous Na_2_SO_4_, filtered, and concentrated. The crude material was purified by either column chromatography over silica gel or recrystallization.

#### 2.2.3. General Procedure for Microwave Heating (**Cz-H** and Its Derivatives) (GP3)

Aryl bromide (1.0 equiv), secondary amine (2.1 equiv), Pd_2_(dba)_3_ (5 mol %), XPhos (10 mol %), *t*-BuONa (2.5 equiv), and toluene (4.0 mL/1.0 mmol aryl bromide) were weighed into a microwave vial. The reaction mixture was irradiated with a microwave at 150 °C for 30 min. The microwave power was set at 300 W. Then, the reaction mass was cooled to room temperature before diluting with chloroform (CHCl_3_) followed by filtration through a pad of Celite. The filtrate was collected and concentrated under reduced pressure. The crude material was purified either by column chromatography over silica gel, recrystallization, or both.

### 2.3 Computational Method

DFT calculations of the failed coupling reactions were performed using Gaussian 09, Revision C.0.^44^ The DFT functional ωB97X-D^45^ was used for all geometry optimizations, where the SDD pseudopotential basis set was employed for Pd and Br^46^ and the 6-31G(d,p) basis set was employed for other atoms.^47,48^ All minimums and transition states were verified by a frequency calculation. Single-point energies were calculated with the def2-TZVP basis set being applied on all atoms.^49^ The solvent effect of toluene was included via the SMD implicit solvation model in the single-point energy calculations.^50^ Gibbs free energies were obtained by adding thermochemical corrections derived from vibrational frequencies at 423.15 K using unscaled frequencies into the single-point energies.

## 3. Results and Discussion

### 3.1 Microwave-Assisted 2-fold Amination

The microwave-assisted Buchwald–Hartwig 2-fold reaction began with the optimization of the reaction temperature. The coupling between **PO-H** and 1,4-dibromobenzene, **9(a)**, to furnish **14(a)** was selected as the model study. A combination of Pd_2_(dba)_3_, XPhos, and *t*-BuONa was used as the precatalyst, supporting ligand, and strong, inorganic base, respectively. Anhydrous toluene was utilized as the solvent, and the microwave power was set at 200 W.

Initially, the coupling reaction was carried out at 130 °C, and subsequently, the reaction was repeated twice with a temperature increment of 10 °C between each attempt (see Table 1, entries 1–3). The highest yield, 93% (Table 1, entry 3), was achieved when the reaction was performed at 150 °C for the double amination of **9(a)** with **PO-H**. However, the yields obtained for the reaction mixtures heated between 130 and 150 °C were similar (Table 1, entries 1–3), indicating that the optimal reaction temperature under microwave heating falls within this range. Next, the optimum reaction time was investigated. It was discovered that 10 min is sufficient to generate the structure **14(a)** with the highest yield of 91% (entry 4–8). Time settings beyond 10 min did not significantly raise the yield; instead, it reached a plateau.

**Table 1:**
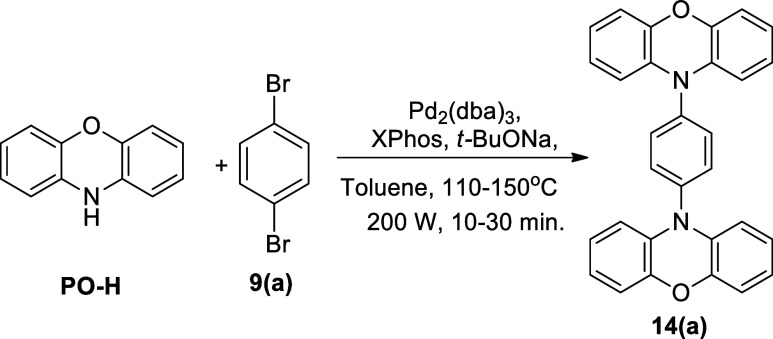
Optimization of the Buchwald–Hartwig Double Amination Reaction of Phenoxazine (**PO-H**) with 1,4-Dibromobenzene, **9(a)**, Assisted by Microwave Irradiation

entry	temperature (T/^o^C)	time (t/min)	products	yield (%)
1	130	30	**14(a)**	91
2	140	30	**14(a)**	90
3	150	30	**14(a)**	93
4	150	5	**14(a)**	80
5	150	10	**14(a)**	91
6	150	20	**14(a)**	90
7	150	25	**14(a)**	89
8	150	30	**14(a)**	91

**Scheme 2:**
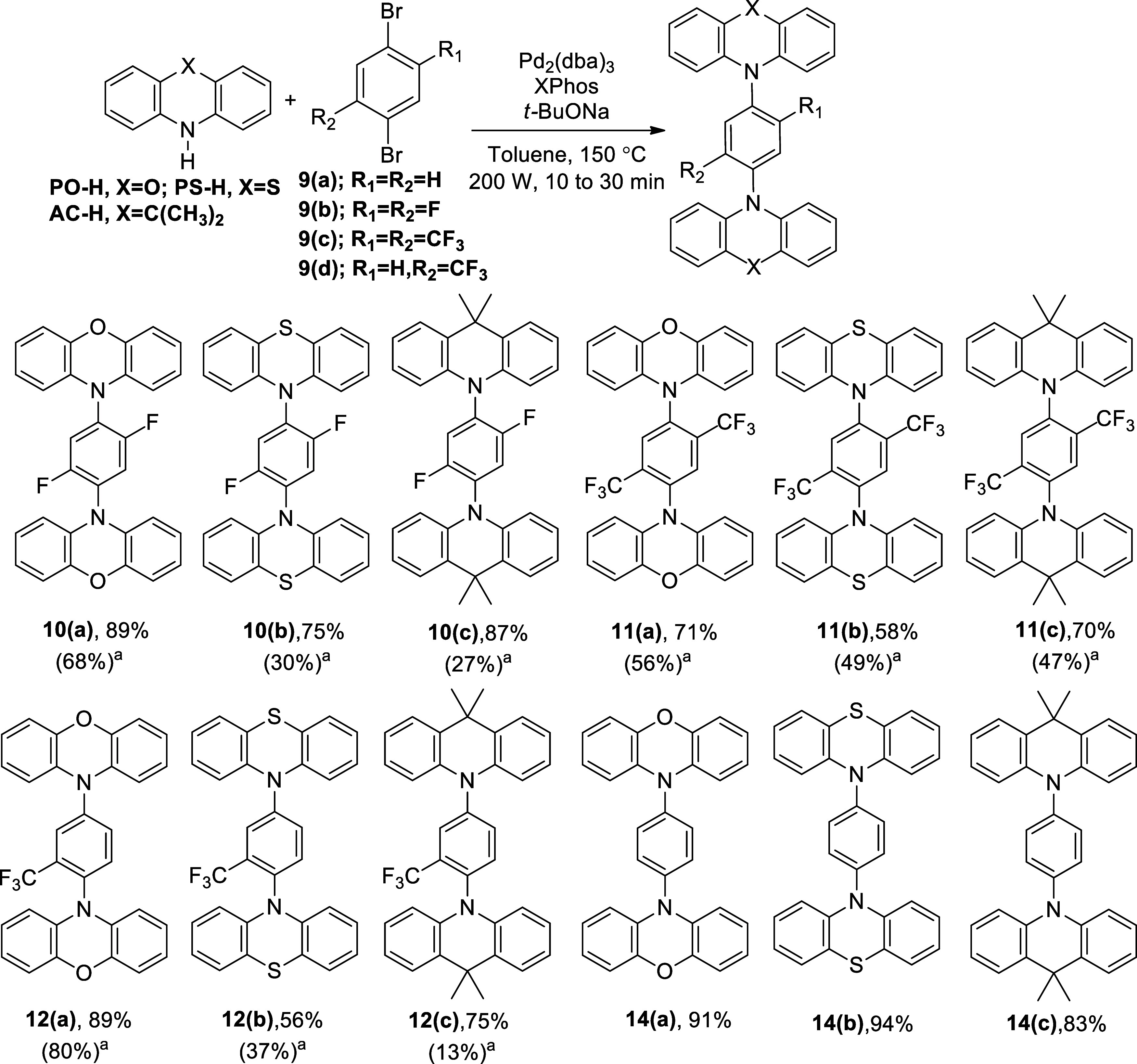
Buchwald–Hartwig 2-fold Amination Reaction of **PO-H**, **PS-H**, and **AC-H** with **9(a)**, **9(b)**, **9(c)**, and **9(d)** under Microwave and Conventional Heating Conditions Conventional heating at 110–120 °C, 24 h.

After the optimum temperature (150 °C) and time (10 min) settings were established for the formation of **14(a)**, the couplings of **9(a)** with **PS-H** into **14(b)** and **9(a)** and **AC-H** into **14(c)** were next explored (Scheme 2). Using the same catalyst, reagent, and solvent, as shown in Table 1, the coupling reaction proceeded smoothly, yielding the targeted compounds with an excellent yield. The compounds **14(b)** and **14(c)** were obtained in high yields of 94 and 83%, respectively.

Next, the 1,4-phenylene core was changed to 1,4-dibromo-2,5-difluorobenzene, **9(b)**, as the A unit, while **PO-H**, **PS-H**, and **AC-H** remained as the D unit (Scheme 2). The structure of **9(b)** possesses two additional fluorine atoms on the 1,4-dibromobenzene core. The presence of fluorine atoms at C2 and C5 of the aromatic ring will create an electron deficiency within the phenylene core. This was done to explore whether the presence of substituents at the *ortho* positions relative to the bromine atoms is compatible with the microwave-assisted Buchwald–Hartwig double amination reaction. Under the same condition as illustrated in Table 1, the coupling between **9(b)** and **PO-H**, **PS-H**, and **AC-H** was a success, as indicated by the high isolated yield of the desired compounds listed in Scheme 2. In addition, the comparison of synthetic yields between microwave and conventional heating methods is included in Scheme 2.

Next, microwave-assisted, 2-fold Buchwald–Hartwig amination was expanded to 1,4-dibromo-2-(trifluoromethyl)benzene, **9(d)**, and 1,4-dibromo-2,5-bis(trifluoromethyl)benzene, **9(c)**, as the coupling partner while maintaining the same D molecules (Scheme 2). Compounds **12(a)** (89%), **12(b)** (56%), **12(c)** (75%), **11(a)** (71%), **11(b)** (58%), and **11(c)** (70%) were obtained in high yields when compared to the conventional heating equivalent (80, 37, 13, 56, 49, and 47%, respectively). From the results obtained in Scheme 2, it can be observed that the reaction was consistent, regardless of the substrates being studied. The introduction of microwave irradiation has the ability to drastically shorten the reaction time from 24 h to only 10 min and it also increases the synthetic yield of all of the coupling products, in comparison to the classical heating approach.

**Scheme 3:**
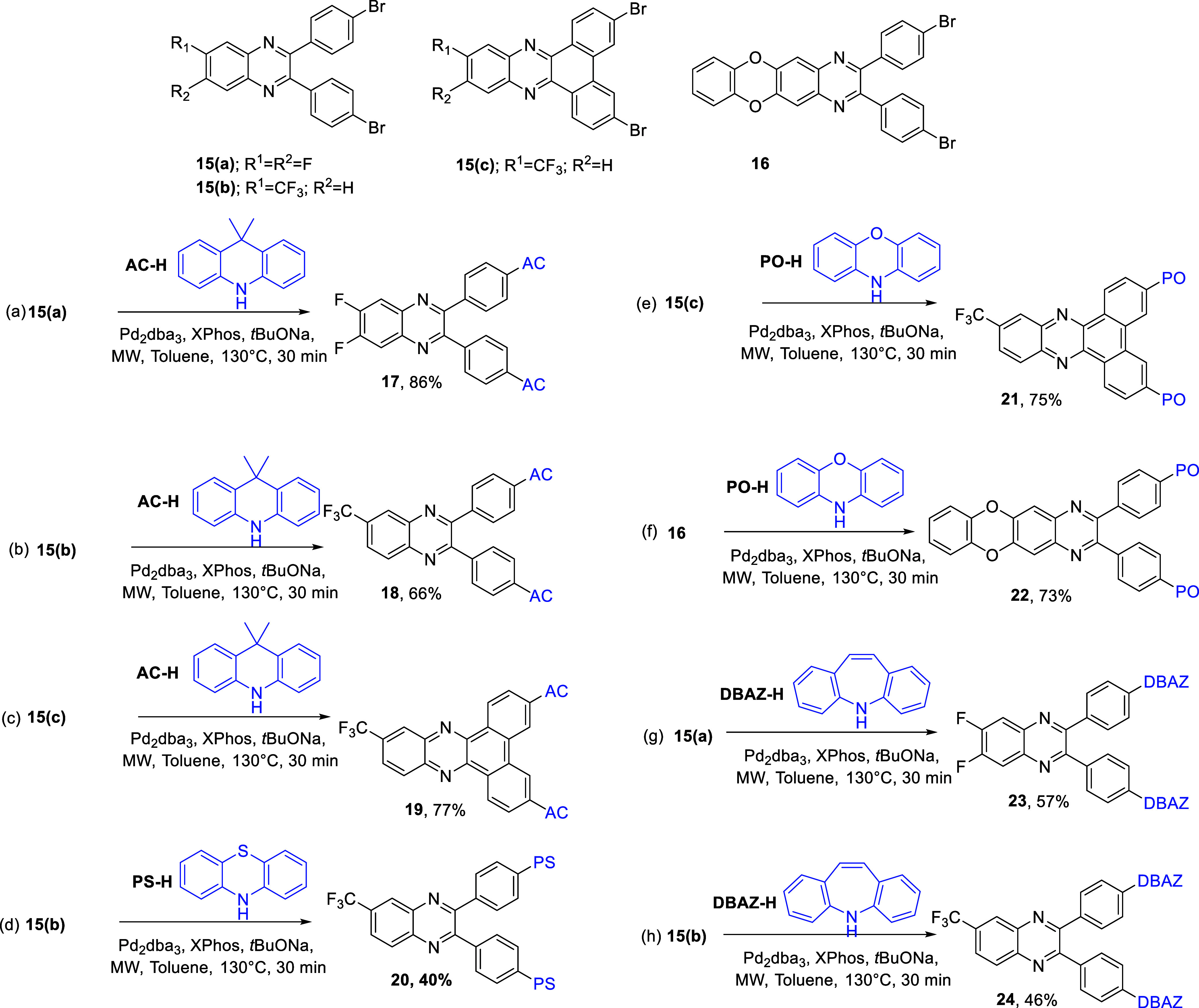
Scope of Microwave Irradiation on Buchwald–Hartwig Double Amination on Various Reactants **PO-H**, **PS-H**, **AC-H**, and **DBAZ-H** were used as the D molecules, whereas **15(a)**, **15(b)**, **15(c)**, and **16** were used as the A molecules.

Intrigued by the results above (Scheme 2), microwave-assisted Buchwald–Hartwig 2-fold amination was then applied to other D and A systems to illustrate its synthetic utility (Scheme 3). The reaction conditions remained consistent with those outlined in Scheme 2, except for the two parameters. First, the reaction time was prolonged to 30 min followed by a reduction in the reaction temperature to 130 °C. As depicted in Scheme 3a–3c, **AC-H** exhibited successful reactions with three different A molecules, numbered as **15(a)**, **15(b)**, and **15(c)**, yielding the 2-fold aminated products **17**, **18**, and **19**, respectively, with high isolated yields (66–86%). On the other hand, **PS-H** underwent a cross-coupling reaction with **15(b)**, resulting in a moderate yield to generate the structure **20** (40%, Scheme 3d). In this regard, **PO-H** reacted with compounds **15(c)** and **16** (Scheme 3e,f), generating the compounds **21** and **22**, respectively, in good yields (75 and 73%, respectively). Furthermore, a new donor, 5*H*-dibenzo[*b,f*]azepine, **DBAZ-H** (Scheme 3g,h), also engaged in the Pd-catalyzed, 2-fold amination reactions with **15(a)** and **15(b)**, affording compounds **23** and **24** in moderate yields (57 and 46%, respectively). Since the reactions presented in Scheme 3 were intended to demonstrate the synthetic utility of microwave-assisted Buchwald–Hartwig double amination, no efforts were made to optimize the yields. Each reaction shown in Scheme 3 was performed only once. To the best of our knowledge, all of the compounds **17**, **18**, **19**, **20**, **21**, **22**, **23**, and **24** are novel compounds.

**Scheme 4:**
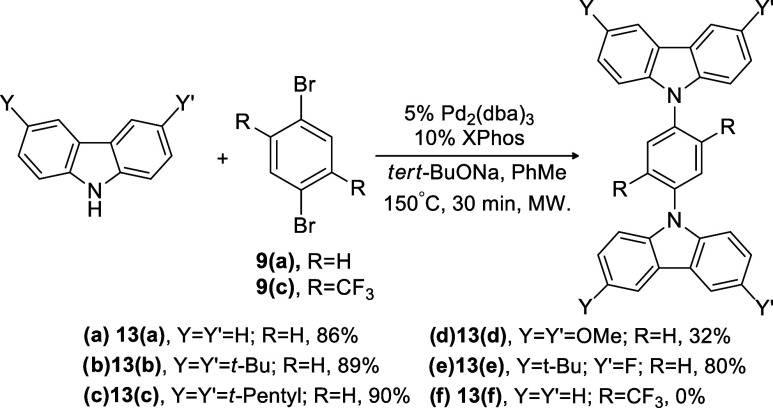
Buchwald–Hartwig Double Amination Reaction Assisted by Microwave Irradiation of Carbazole (**Cz-H**) with 1,4-Dibromobenzene (**9(a)**) Compounds **13(b**–**e)** are novel compounds.

Furthermore, to further expand the synthetic utility of microwave irradiation, Buchwald–Hartwig double amination using **Cz-H** as the D unit was tested for its compatibility with microwave irradiation. **9(a)** was chosen as the coupling partner, and its reaction with **Cz-H** to form **13(a)** was selected as the model study (Scheme 4). For this reaction, the microwave power used was 300 W and the reaction time was 30 min to ensure complete conversion. **13(a)** was obtained with an excellent yield of 86% (Scheme 4a). Encouraged by this outcome, **Cz-H** derivatives with various substituents at the C3 and C6 positions on carbazole were coupled with **9(a)** under the same conditions. As can be seen in Scheme 4, except for **13(d)** (32%), other products such as **13(b)**, **13(c)**, and **13(e)** were isolated with excellent yields of 89, 90, and 80%, respectively. The compound **13(d)** has solubility issues, where it is partially soluble in CHCl_3_. Hence, making the general workup procedure as described in the Synthesis section unsuitable for this product led to the significant drop in the isolated yield.

One common question is why we use aryl bromides instead of aryl chlorides in our reactions. One primary reason is that bromination reactions to form compounds **9(a)** and **9(c)** are generally easier and more selective than chlorination. In fact, most of the reported Buchwald–Hartwig double amination reactions in the literature that use conditions similar to ours have employed aryl bromides.^4−9,31,32^ Additionally, Zheng et al. reported that Buchwald–Hartwig amination using electron-deficient aryl chlorides did not yield the desired coupling products.^51,52^ Our compounds **9(a)** and **9(c)** are indeed electron-deficient aryl halides (Scheme S1 of the SI).

Since the cross-coupling of **Cz-H** and its derivatives with **9(a)** was successful, the scope of the microwave-assisted Buchwald–Hartwig 2-fold amination was further extended to include **9(c)** as the A unit. Being an electron-withdrawing group, trifluoromethyl can reduce the electron density within the A core, thus making the 1,4-phenylene motif a better and more electron-deficient A unit as compared to **9(a)**. Furthermore, the presence of the extending trifluoromethyl groups can also ensure a large dihedral twisting between the D and A moieties, which is crucial in the spatial separation of the frontier orbitals.

Surprisingly, the reaction did not proceed as expected, and the structure **13(f)** was not detected by TLC, ^1^H, and ^13^C analyses (Scheme 4f). This observation seems to be contradicting earlier data, where we managed to obtain the compounds **11(a)**, **11(b)**, and **11(c)** via Buchwald–Hartwig amination of **9(c)** with **PO-H**, **PS-H**, and **AC-H**, respectively, using the conventional heating method^31^ as well as microwave irradiation (Scheme 2). Replacement of Pd_2_(dba)_3_ with Pd(OAc)_2_ and changing the ligand from XPhos to P(*t*-Bu)_3_ and RuPhos also did not produce the desired products. One could argue that **Cz-H** is responsible for the failure of the Buchwald–Hartwig reaction; however, the reactions illustrated in Scheme 4a–e proceed successfully. Similarly, it can be suggested that the presence of two CF_3_ groups at the C2 and C5 positions contributes to the reaction’s lack of success, whereas the reaction shown in Scheme 2 (formation of compound **11(a**–**c)**) did not encounter any issues. Because of the lack of reactivity between **Cz-H** and **9(c)**, we therefore turned our attention to mechanistic computational investigation to provide some insights on the origin of nonreactivity.

### 3.2 Computational Investigation of the Setback Experienced and Shown in Scheme 4f

**Figure 3:**
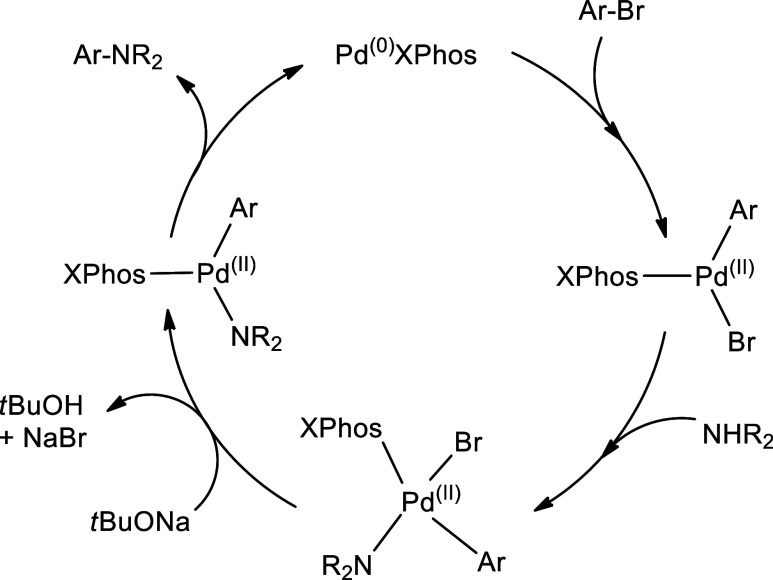
Proposed catalytic cycle of Buchwald–Hartwig cross-coupling.

To rationalize the cause of nonreactivity between **Cz-H** and **9(c)**, as shown in Scheme 4f, we performed DFT calculations based on the synthetic method established earlier in literature and the general catalytic cycle, as shown in Figure 3.^53−55^ The catalytic cycle begins with the combination of Pd_2_(dba)_3_ and XPhos to generate the active catalyst, which is monoligated Pd^(0)^XPhos through the displacement of the dba ligand by XPhos.^56^ The active catalyst initiates the catalytic cycle by oxidative addition onto the C–Br bond to form the σ-bonded Pd(II) complex. Next, the amine coordinates to the Pd center followed by deprotonation by the *tert*-butoxide ion. Then, the complex undergoes a reductive elimination (RE) process to form the C(sp^2^)-N bond, thus regenerating Pd(0) to restart the cycle. It is well known that the rate-determining step of the catalytic cycle for the construction of C(sp^2^)-N is the reductive elimination (RE) step. This has been proven both experimentally^57,58^ and theoretically.^59−63^ Thus, we use the DFT approach to understand the RE step for different amines with the aryl halides **9(a)**, **9(b)**, and **9(c)**.

**Figure 4:**
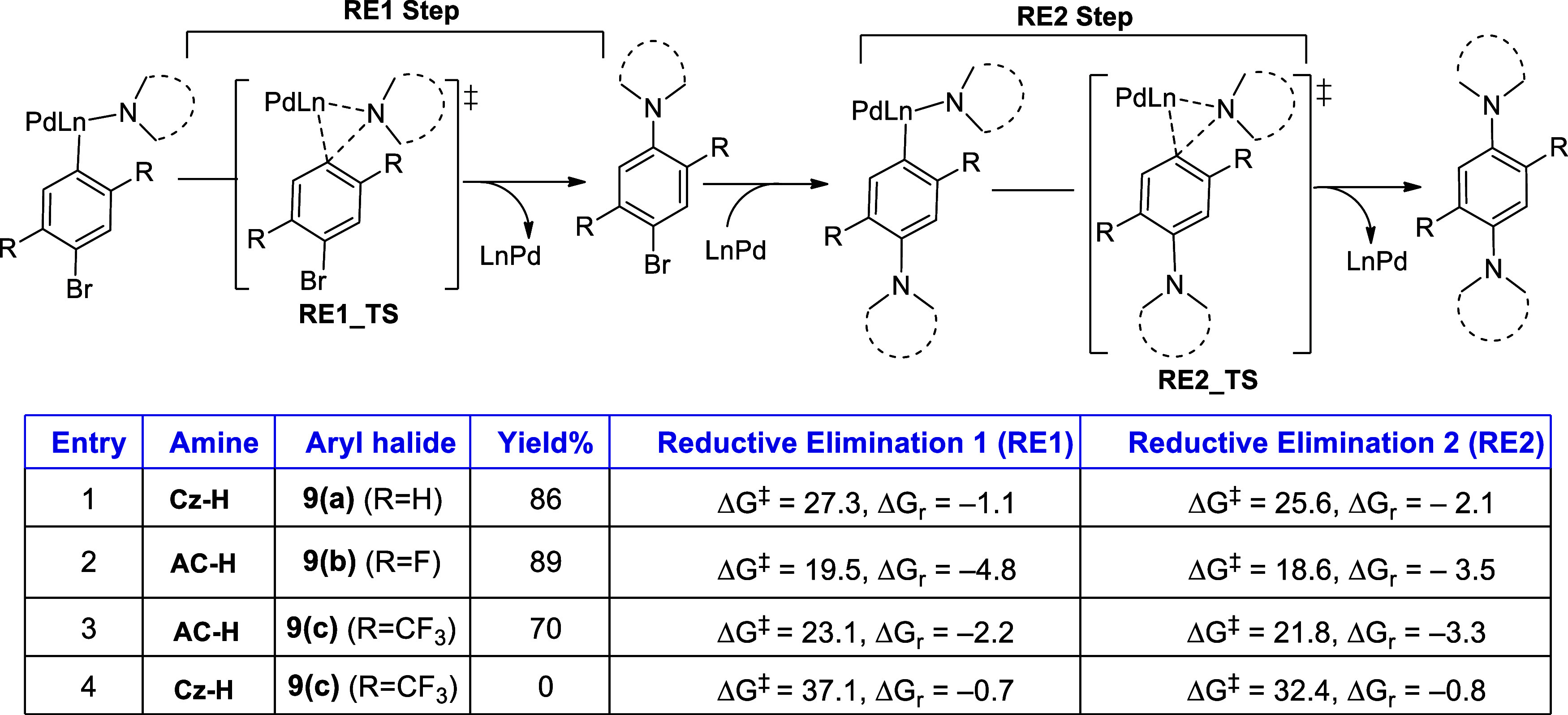
Calculated reductive elimination free energy barriers (Δ*G*^‡^) and energy profile (Δ*G*_r_) by DFT calculations of the successful and unsuccessful 2-fold C–N bond coupling across various substrates.

Our DFT calculations were performed with (SMD/Toluene)-ωB97X-D/def2-TZVP//ωB97X-D/6-31G(d,p) at the SDD level of theory at 423.15 K (Figure 4) (see the SI for computational details). We calculated the energy barriers of the reductive elimination (RE) step for the double amination of **9(a)** and its derivatives, **9(b)** and **9(c)**. For the first amination, the first RE is called as RE1, and RE2 refers to the second reductive elimination of the catalytic C–N bond formation.

Experimentally, when **9(a)** is not substituted (R = H), the use of **Cz-H** gave a C–N bond coupling of high yield (86%). The calculated barriers for RE1 and RE2 are 27.3 and 25.6 kcal/mol, respectively, as slightly exergonic steps (entry 1, Figure 4). Despite the high energy barriers for both RE1 and RE2, these calculated values are still in accord with the high temperature and microwave conditions. However, when **9(a)** is changed to **9(c)** (R = CF_3_) and reacted with **Cz-H**, the calculated energy barriers for both RE1 and RE2 for the reaction are dramatically higher (RE1: Δ*G*^‡^ = 37.1 kcal/mol, Δ*G*_r_ = −0.7 kcal/mol; RE2: Δ*G*^‡^ = 32.4 kcal/mol, Δ*G*_r_ = −0.8 kcal/mol, entry 4, Figure 4). This is an expected result due to the strong electron-withdrawing effect of the CF_3_ groups onto **9(c)**. Experimentally, this reaction resulted in a lack of reactivity (0% yield, entry 4, Figure 4). The high energy barriers for both RE1 and RE2 steps, as well as the thermoneutral nature of the steps, result in the impediment of product formation.

When the amine is switched from **Cz-H** to **AC-H** (entry 3, Figure 4), the calculated energy barriers of its reaction with **9(c)** (RE1: Δ*G*^‡^ = 23.1 kcal/mol, Δ*G*_r_ = 2.2 kcal/mol; RE2: Δ*G*^‡^ = 21.8 kcal/mol, Δ*G*_r_ = 3.3 kcal/mol, entry 3, Figure 4) become significantly less in comparison to the coupling of **Cz-H** with **9(c)** (entry 4, Figure 4), and also lower than that between **Cz-H** and **9(a)**. This indicates that the nature of amine has a significant effect on the energy barriers. The energy barrier calculated here for the 2-fold coupling of **AC-H** with **9(c)** is reflected in the success of the reaction experimentally (70% yield, entry 3, Figure 4). The reaction of **AC-H** and **9(b)** gives an RE1 value of 19.5 kcal/mol and an RE2 value of 18.6 kcal/mol, which are the lowest among the four studied systems. This indicates that the changes from **Cz-H** to **AC-H** and **9(c)** to **9(b)** both provide favorable effects to the energy barriers and, consequently, result in a high yield of 89% (entry 2, Figure 4). Therefore, the success and failure of the C–N coupling reactions can clearly be understood by their calculated energy barriers due to the electronic effect, but the steric effect cannot be excluded, as revealed by further calculations (see below).

**Figure 5:**
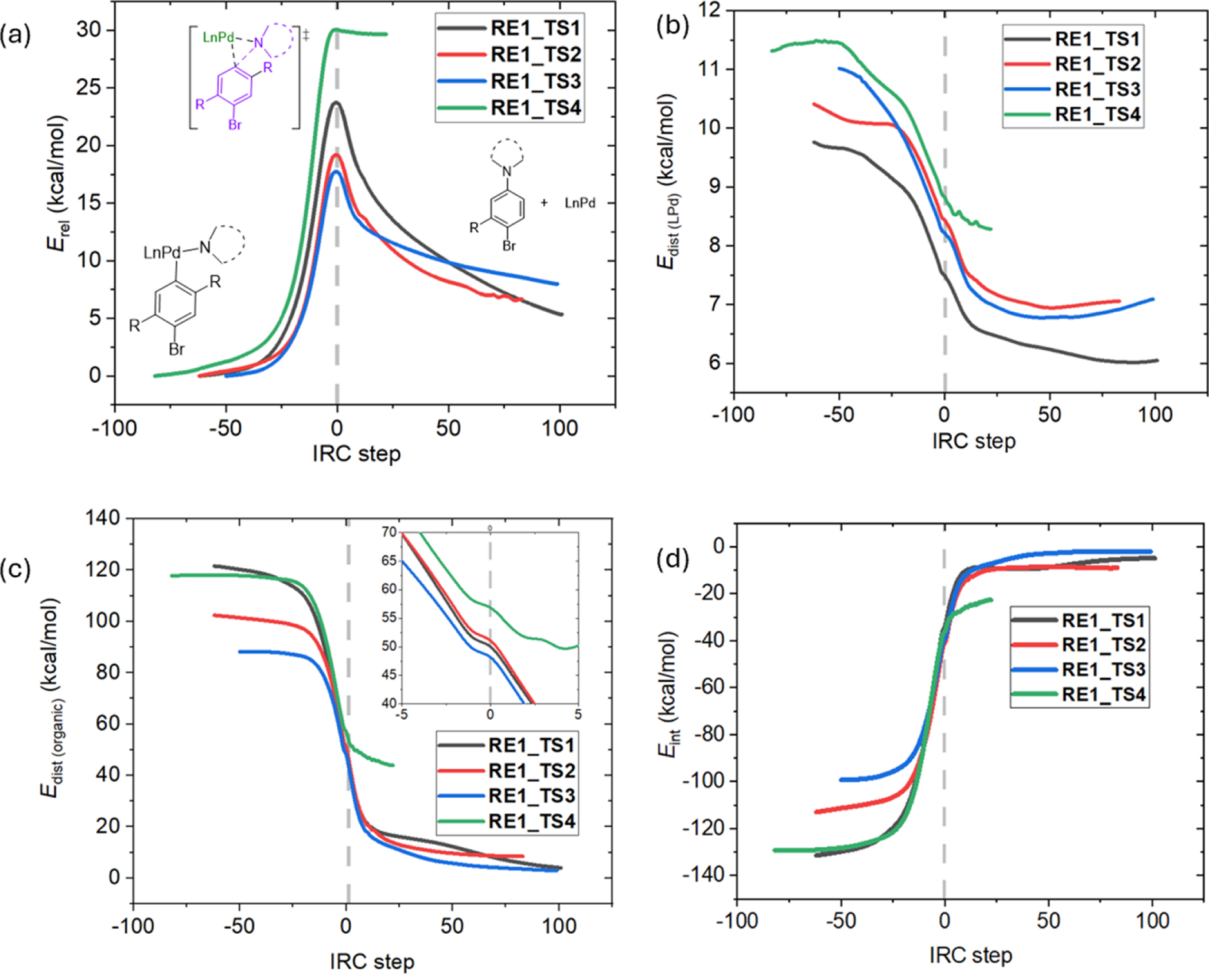
(a) IRC energy curve for the four **RE1_TS**s. **RE1_TS1** refers to the coupling between **Cz-H** and **9(a)**, **RE1_TS2** refers to the coupling between **AC-H** and **9(b)**, **RE1_TS3** refers to the coupling between **AC-H** and **9(c)**, and **RE1_TS4** refers to the coupling between **Cz-H** and **9(c)**. DIA curves for all four **RE1_TS**s: (b) distortion on **LPd**, (c) distortion on the organic part, and (d) the interaction energies.

To further elucidate the origin of the difference in RE barriers, especially the reason why entry 4 of Figure 4 exhibits an unexpectedly low reactivity, distortion–interaction analysis (DIA) was performed on all four transition states of the first reductive elimination steps to form the one-fold coupling product (**RE1_TS**). The molecule was partitioned into two fragments: **LPd** (the ligand and Pd atom) and organic moieties. Along the intrinsic reaction coordinate (IRC) pathway, the distortion and interaction energies of each fragment were monitored (Figure 5). The difference in energy barriers is clearly depicted in the IRC electronic energy curve (Figure 5a), where the two reactions involving **Cz-H** (**RE1_TS1** and **RE1_TS4**) exhibit significantly higher energy barriers within a range of 10 kcal/mol. However, the distortion experienced by the **LPd** fragment is quite similar among the four transition states, with a variation of only ∼2 kcal/mol. Likewise, the interaction energy curves nearly overlap in the transition-state region. Therefore, the difference in barriers must have originated from the distortion of the organic moiety. Indeed, **RE1_TS4** (cross-coupling between **Cz-H** and **9(c)**) features a significantly larger distortion on the organic part, which is 7 kcal/mol higher than all of the others. Interestingly, although the overall Gibbs free energy of the RE through **RE1_TS4** is −0.8 kcal/mol, the product–catalyst complex depicted by the IRC is highly endergonic (∼30 kcal/mol, as shown in Figure 5a). This is also reflected by its much higher distortion energy on the product side.

**Figure 6:**
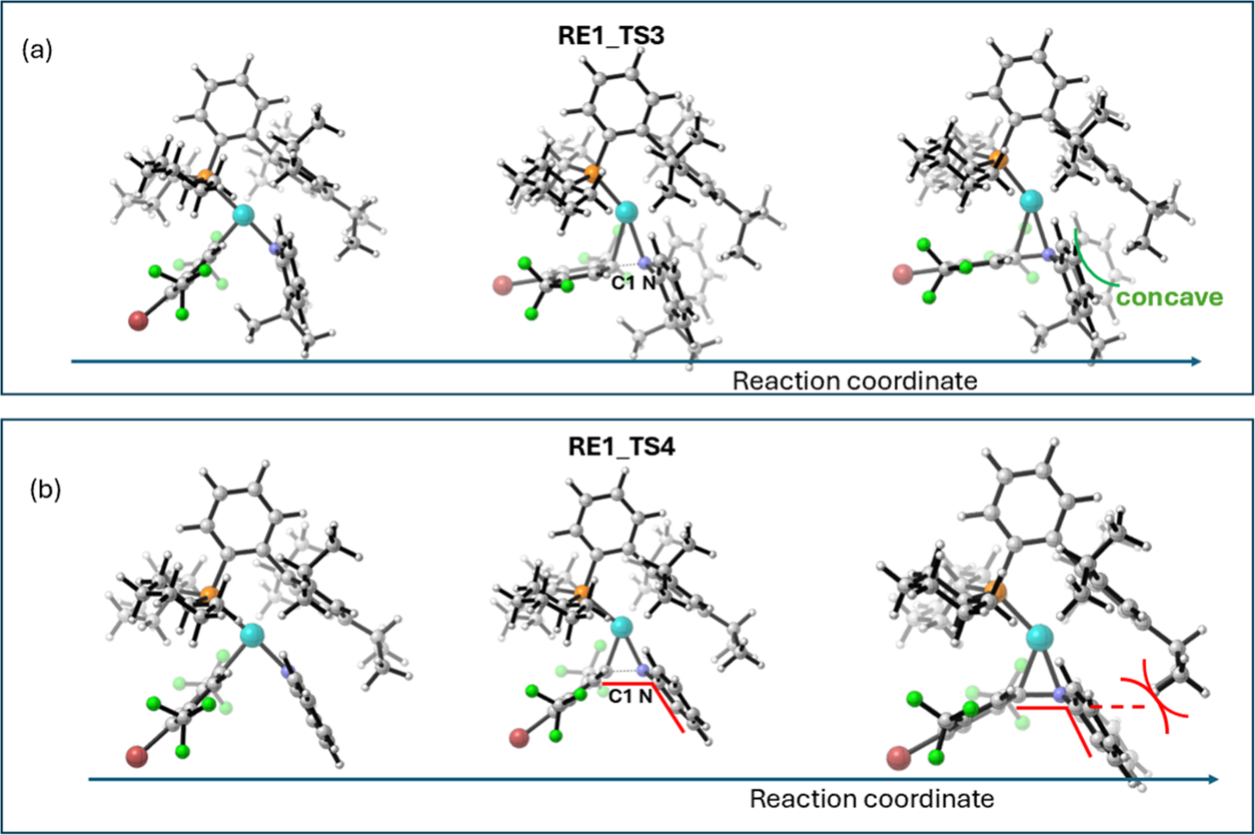
Selected geometries along the IRC pathways through (a) **RE1_TS3** and (b) **RE1_TS4**.

The difference in the distortion energy of the organic part can be attributed to the geometric requirements of the **Cz-H** and **AC-H** rings. For the aromatic **Cz-H** ring, the N-substituent group (C1 in Figure 6) is expected to be coplanar with the ring. However, due to the steric bulk imposed by the ligand’s 2,4,6-tri(isopropyl)phenyl ring, a planar geometry at the N atom is inaccessible until the product fully dissociates. The deviation from the ideal geometry of the sp^2^ N atom leads to a substantial compensation in the distortion energy. In contrast, the **AC-H** group bears an almost sp^3^ N atom, and its trigonal pyramidal geometry is perfectly satisfied along the reaction pathway. Furthermore, the **AC-H** ring features a nonplanar, concave geometry, which also accommodates well with the convex shape of the ligand’s isopropyl group. Consequently, the deviation from its ideal planar geometry leads to a higher RE barrier for **Cz-H**, as shown by **RE1_TS1** versus **RE1_TS2** and **RE1_TS4** versus **RE1_TS3**. This energetic compensation is further exacerbated in CF_3_-substituted **RE1_TS4**, as compared to its H-analogue **RE1_TS1**, because it features a shorter C1–N distance (1.65 versus 1.79 Å), which accentuates the tendency to adopt a planar geometry around the N atom, and therefore **RE1_TS4** exhibits an even higher barrier than **RE1_TS1**.

## 4. Conclusions

In conclusion, a microwave-assisted Buchwald–Hartwig cross-coupling procedure between the D and A units has been developed for the synthesis of novel optoelectronic molecules, particularly TADF compounds. The results demonstrate the utility of this synthetic technique in forging C(sp^2^)-N bonding. The incorporation of microwave irradiation into the coupling process led to a significant reduction in the reaction time, from 24 h under conventional heating conditions to 10–30 min. The obtained yields range from moderate to excellent, surpassing those achieved with their conventional heating counterparts. Our method exhibited substantial compatibility with various aryl bromides and secondary amines, including phenoxazine (**PO-H**), phenothiazine (**PS-H**), acridine (**AC-H**), and carbazole (**Cz-H**). While the coupling between **Cz-H** and its derivatives with **9(a)** generated the coupled products with well-obtained yields, the coupling between **Cz-H** and **9(c)** was unsuccessful. DFT calculations were carried out to rationalize the lack of reactivity observed, which was attributed to the high energy barriers of the reductive elimination (RE) steps. While the reaction between **9(a)** (R = H) and **Cz-H** generated the product with an excellent yield (86%) despite the high energy barrier during the reductive elimination (RE) steps, substituting **9(a)** with the electron-withdrawing CF_3_ groups at the C2 and C5 positions dramatically increased the barrier, resulting in a 0% yield. Conversely, replacing **Cz-H** with **AC-H** lowered the RE barriers and enhanced the isolated yield, underscoring the significance of the amine’s electronic properties under the current conditions. DIA reveals that the higher barriers are due to the greater distortion originating from the organic fragment, particularly for **Cz-H** with **9(c)**, where the ideal planar geometry around the nitrogen atom enhances the steric hindrance between the carbazole ring and the isopropyl group of the XPhos ligand, thereby increasing the energy barriers and preventing the coupling reaction to proceed. Thus, based on computational calculations, both electronic and steric effects are key determinants of the reaction outcomes. In conclusion, despite the numerous breakthroughs made, the development of efficient TADF emitters necessitates further exploration, a venture currently underway in our research group.

## Supporting Information Available

Computational investigations with regard to the singlet states, triplet states, and HOMO and LUMO levels; a brief collection of the photophysical data of selected synthesized compounds; detailed synthetic procedures; chemical characterization data; and copies of NMR spectra (PDF)
