# Supplementary material for: Microwave-Assisted Buchwald–Hartwig Double Amination: A Rapid and Promising Approach for the Synthesis of TADF Compounds
Source: ACS Omega. 2024 Dec 9;9(51):50446–57. doi: 10.1021/acsomega.4c07563 (PMC11684481; doi:10.1021/acsomega.4c07563)
Supplement: Supplementary file 1 — ao4c07563_si_001.pdf [file ao4c07563_si_001.pdf]

Supporting Information

Microwave-Assisted Buchwald-Hartwig Double Amination: A Rapid and Promising Approach for the Synthesis TADF Compounds

Nor Shafiq Mohd Jamel[a], Levani Skhirtladze[b], Aqeel A. Hussein*[c], Yumiao Ma[d],[e], Kai Lin Woon[f], Muhammad Kumayl Abdulwahab[a], Juozas V. Grazulevicius**[g], Azhar Ariffin***[a,g]

[a] Department of Chemistry, Faculty of Science, Universiti Malaya, 50603 Kuala Lumpur, Malaysia

[b] Linköping University Department of Science and Technology Bredgatan 33 Norrkoping, SE 601 74, Sweden

[c] Department of Biology, College of Science, Al-Qasim Green University, 51013 Al-Qasim, Babylon, Iraq

[d] BSJ Institute, Beijing 100084, People's Republic of China

[e] Beijing Orienda Instrument Co. Ltd. Beijing, 102200, People's Republic of China

[f] Department of Physics, Faculty of Science, Universiti Malaya, 50603 Kuala Lumpur, Malaysia

[g] Department of Polymer Chemistry and Technology, Kaunas University of Technology, Baršausko 59, Kaunas, 51423, Lithuania

Table of Content

1. Computational Method for Determination of S_1, T_1, and Frontier Orbitals 3
   1.1. Outcome of TD-DFT and DFT Calculation of Selected Compounds 3
   1.2. Calculated Values of S_1, T_1, and ΔE_{ST} of the Remaining Proposed Compounds 5
2. Brief Summary of Photophysical Characterization of Selected Compounds 8
3. Synthetic Procedure 9
   3.1. Bromination of 1,4-bis(trifluoromethyl)benzene into 9(c) 9
   3.2. Microwave-assisted, Two-fold Buchwald-Hartwig Coupling..................................... 9
   3.3. Acidic condensation between substituted 1,2-phenylene diamine and benzil derivatives into quinoxaline skeleton.. 19
   3.4. Microwave-assisted aromatic nucleophilic substitution (S_NAr) of 15(a) into 16....... 20
   3.5. Microwave-assisted, Two-fold Buchwald-Hartwig Coupling (Contd.)...................... 21
   3.6. Microwave-assissted, Two-fold, Buchwald-Hartwig Amination of Aryl Chloride 25
   3.7. Friedel-Crafts alkylation of carbazole into 25(a) and 25(b) 26
   3.8. Two-step synthesis of carbazole 25(c) and carbazole 25(d). 27
4. Copies of ^{1}H, ^{13}C, and ^{19}F Spectrums 30
5. DFT Computation (Mechanistic Studies) 53
6. Optimized structures from DFT Calculations involved in mechanistic study 54
7. Optimized Molecular structure From TD-DFT Calculations 111
8. References.. 146

1. Computational Method for Determination of S_1, T_1, and Frontier Orbitals

Density functional theory (DFT) using range-separated hybrid functional LC-ωPBEh was used to obtain the ground state molecular geometry at def2-svp basis set. ω was tuned using the golden ratio algorithm under polarizable continuum model (PCM) with a dielectric constant of 2.38 corresponding to toluene and solvent radius of 3.48 Å. Time-dependent DFT (TD-DFT) calculation was performed using Terachem 1.93[1] software 10 and a Graphic Processing Unit server that had 64 GB RAM installed to support eight Tesla K10 graphic cards at the University of Malaya Data Intensive Computing Center. The results were visualized using Visual Molecular Dynamics (VMD) software.

1.1. Outcome of TD-DFT and DFT Calculation of Selected Compounds

To support our claim that symmetrical, D-A-D configuration is a good design for TADF molecules (the list of the designed molecules can be seen in **Figure S1**), we calculated the singlet and triplet excited states (S_1, T_1, and T_2) and the distribution of the frontier orbitals (HOMO and LUMO levels). Initially, the use of (Trifluoromethyl)benzene rather than 1,4-bis(trifluoromethyl)benzene as an acceptor breaks the symmetry of the molecules in a D-A-D configuration, resulting in electron density being located at HOMO of the donor next to the trifluoromethyl group. This effect is stronger in **12(b),** followed by **12(c)** and then **12(a)** with electron density of HOMO located at the donor are 93.9%, 74.5%, and 73.0% respectively. With 1,4-bis(trifluoromethyl)benzene as an acceptor, the HOMO lobes of **13(f)**, **13(g)**, and **13(h)** are located equally between the two donors (see **Figure S2**).

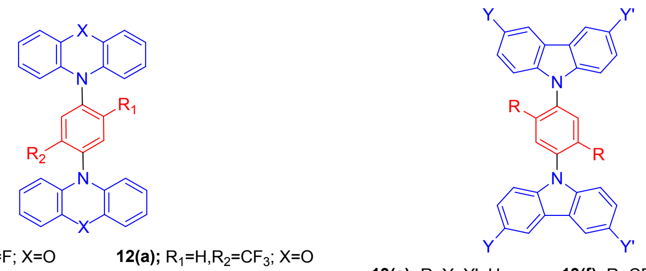

10(a); R_1=R_2=F; X=O
10(b); R_1=R_2=F; X=S
10(c); R_1=R_2=F; X=CMe_2
11(a); R_1=R_2=CF_3; X=O
11(b); R_1=R_2=CF_3; X=S
11(c); R_1=R_2=CF_3; X=CMe_2

12(a); R_1=H,R_2=CF_3; X=O
12(b); R_1=H,R_2=CF_3; X=S
12(c); R_1=H,R_2=CF_3; X=CMe_2
14(a); R_1=R_2=H; X=O
14(b); R_1=R_2=H; X=S
14(c); R_1=R_2=H; X=CMe_2

13(a); R=Y=Y'=H
13(b); R=H; Y=Y'=^{t}Bu
13(c); R=H; Y=Y'=^{t}Pent
13(d); R=H; Y=Y'=OMe
13(e); R=H; Y=F; Y'=^{t}Bu

13(f); R=CF_3; Y=Y'=H
13(g); R=CF_3; Y=Y'=^{t}Bu
13(h); R=CF_3; Y=Y'=^{t}Pent
13(i); R=CF_3; Y=Y'=OMe
13(j); R=CF_3; Y=F; Y'=^{t}Bu

Figure S1. Structure of proposed molecules

The energy levels between HOMO and HOMO-1 are nearly degenerate in all cases even for **13(f) 13(g)**, and **13(i)**, respectively. The vertical transitions for all six compounds are dominated by the transition from the HOMO to the LUMO levels, hence constituted charge transfer states with little electronic density overlap between HOMO and LUMO levels. As a result, the singlet-triplet splitting is small of 0.02-0.11 eV for all the selected compounds (**Figure S2**).

The results from TD-DFT calculations presented here suggested that the designed compounds, **12(b)** (S_1=3.14eV), **12(c)** (S_1=3.16eV), **13(f)** (S_1=3.21eV) and **13(i)** (S_1=3.11eV) have potentially good blue TADF properties, whereas compounds **12(a)** (S_1=2.87eV) and **13(g)** (S_1=2.74eV) have potentially good bluish green TADF properties. As proven by the TD-DFT calculation above, symmetrical D-A-D configurations are a good design for potential TADF molecules. Driven by these promising TD-DFT results, the microwave-assisted two-fold Buchwald-Hartwig amination syntheses will follow the symmetrical D-A-D configurations as shown in **Figure S1**.

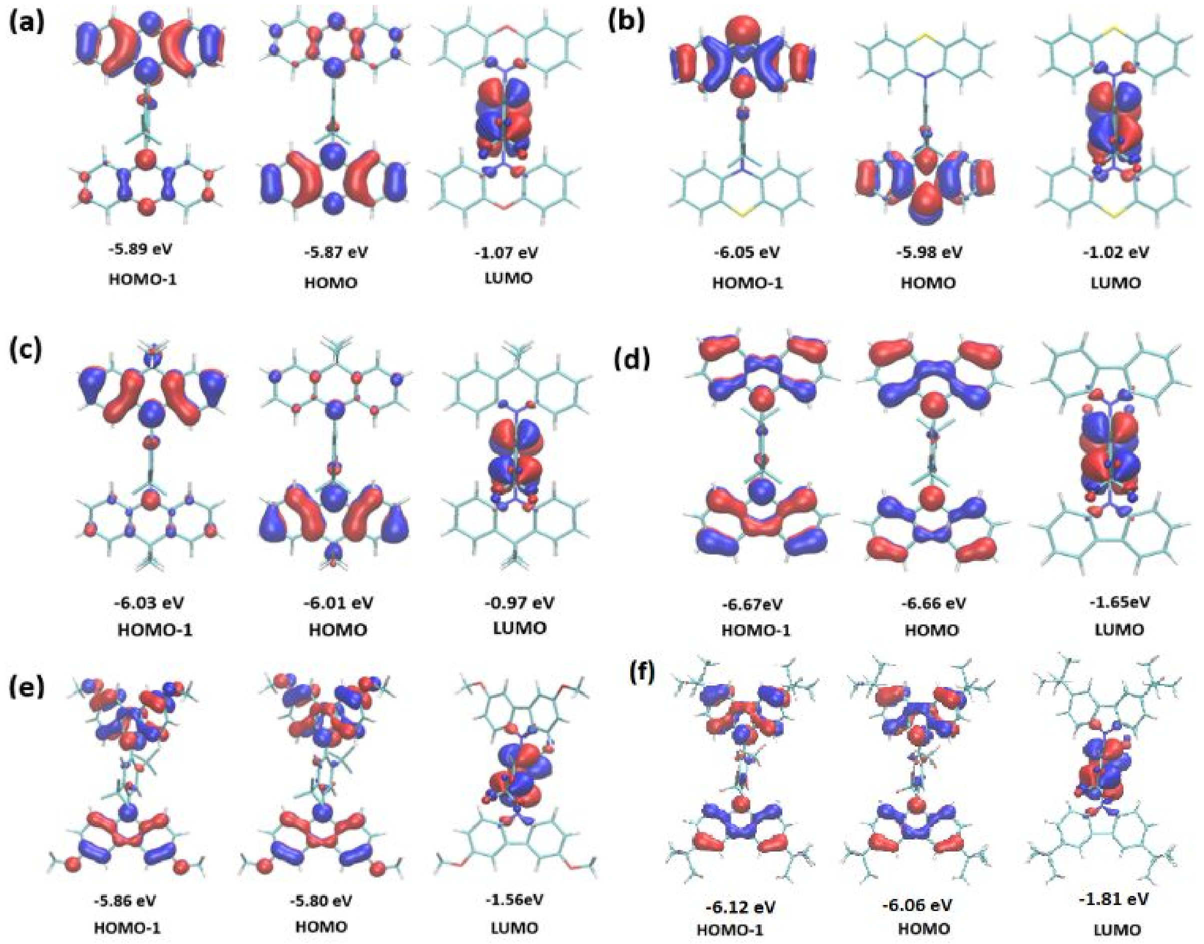

Figure S2. HOMO, HOMO-1, and LUMO distributions and their respective energy levels of the compound (a) **12(a)**, (b) **12(b)**, (c) **12(c)**, (d) **13(f)**, (e) **13(i)**, and (f) **13(g)**.

Table S1. S_1, T_1, T_2, and $\Delta E_{S1\text{-}T1}$ of **12(a)-(c)**, **13(f)-(g)**, and **13(i)**

Compound	S_1 (eV)	T_1 (eV)	T_2 (eV)	$\Delta E_{S1\text{-}T1}$ (eV)
**12(a)**	2.87	2.82	2.83	0.05
**12(b)**	3.14	3.03	3.04	0.11
**12(c)**	3.16	3.13	3.15	0.03
**13(f)**	3.21	3.19	3.20	0.02

**13(g)**	2.74	2.64	2.73	0.10
**13(i)**	3.11	3.01	3.11	0.11

1.2. Calculated Values of S_1, T_1, and ΔE_{ST} of the Remaining Proposed Compounds

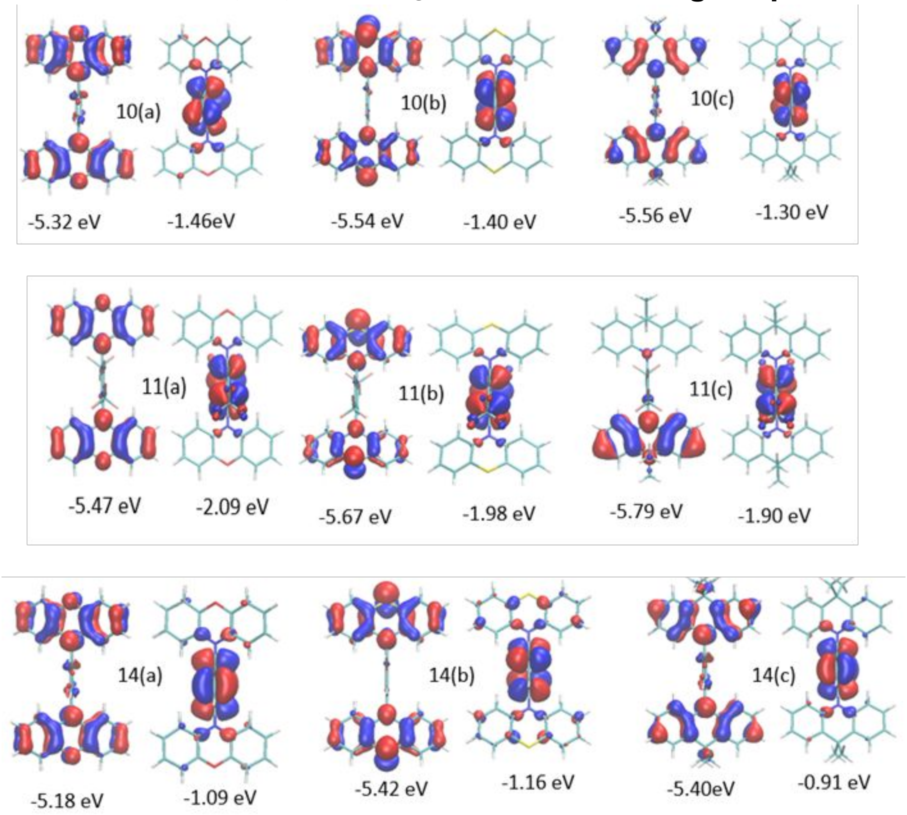

Figure S3. HOMO and LUMO distributions and their respective energy levels of **10(a)**-**(c)**, **11(a)**-**(c)**, and **14(a)**-**(c)** with their respective energy levels

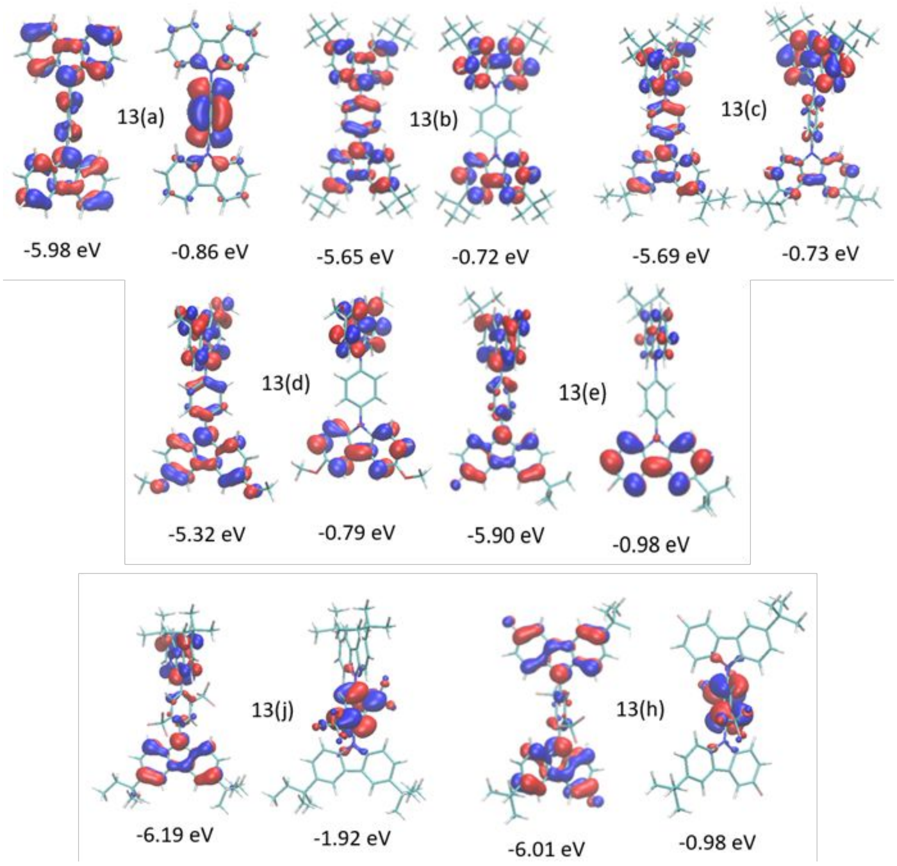

Figure S4. HOMO and LUMO distribution for compound **13(a)**-**(e)** and **13(h)**-**(j)** with their respective energy levels.

For compounds **13(a)**-**(e)**, due to the absence of trifluoromethyl group presence, there is no clear spatial separation between the HOMO and LUMO levels, which consequently leads to overlapping between these two frontier orbitals. The effect of this phenomenon is reflected in the large ΔE_{ST} values (exceeding 0.1 eV) of these compounds as tabulated in **Table S1**. Therefore, these compounds are not considered to possess TADF behaviour. On the other hand, the presence of the extending trifluoromethyl groups on the structures **13(f)**-**(j)** leads to a clearer separation between the frontier orbitals. As a result, the ΔE_{ST} values become smaller due to smaller degree of overlapping between the HOMO and LUMO levels.

Table S1: Theoretical calculation of S_1, T_1, and ΔE_{ST}

Compounds	Triplet (ev)	Singlet (eV)	ΔE_{ST} (eV)
**10(a)**	2.77	2.91	0.14
**10(b)**	3.10	3.21	0.11
**10 (c)**	3.18	3.23	0.05
**11(a)**	2.38	2.40	0.02
**11(b)**	2.74	2.76	0.02
**11(c)**	2.85	2.88	0.02
**12(a)**	2.73	2.77	0.04
**12(b)**	3.01	3.11	0.09
**12(c)**	3.07	3.10	0.03
**13(a)**	3.38	3.96	0.59
**13(b)**	3.22	3.83	0.60
**13(c)**	3.23	3.85	0.62
**13(d)**	2.80	3.52	0.72
**13(e)**	3.14	3.84	0.70
**13(h)**	2.89	3.10	0.22
**13(j)**	2.94	3.13	0.19
**14(a)**	2.88	3.13	0.25
**14(b)**	2.69	3.11	0.42
**14(c)**	3.30	3.46	0.16

2. Brief Summary of Photophysical Characterization of Selected Compounds

We have successfully synthesized compounds **10(a)**-**(c)**, **11(a)**-**(c)**, and compound **12(a)-(b)** through the Buchwald-Hartwig double amination reaction using conventional heating method.[2-4] Our experimental findings demonstrate that all these compounds exhibit a narrow energy gap between their S_1 and T_1, with ΔE_{ST} values ranging from 0.01 to 0.17 eV, as summarized in **Table S2**. Unlike 1,4-difluorobenzene, which acts as an acceptor in the **10(a)**-**10(c)** series and displays higher-energy singlet states (evidenced by an emission onset of ≥ 3.00 eV), 1,4- bis(trifluoromethyl)benzene behaves as a more potent acceptor. Consequently, the **11(a)**-**11(c)** series exhibits lower singlet energies. When employing only (trifluoromethyl)benzene, as observed in **12(a)** and **12(b)** for thermally activated delayed fluorescence (TADF) design, the emission onset increases compared to 1,3-bis(trifluoromethyl)benzene. This suggests that the acceptor's strength correlates with the number of fluorinated groups present. All molecules listed in **Table S2** display a small ΔE_{ST} (<0.10 eV), except for **10(b)**. **AC-H**, serving as a donor, demonstrated a higher photoluminescence quantum yield (PLQY), evident in **10(c)** and **11(c)**, whereas **PO-H** significantly reduced the PLQY when used as a donor in the TADF D-A-D design. Additionally, the **11(a)**-**11(c)** series showcased delayed fluorescence and slightly longer prompt fluorescence lifetimes compared to the **10(a)**-**10(c)** and **12(a)**-**12(b)** series. The absence of delayed fluorescence in other molecules is attributed to the substantial gap between the locally excited triplet state (^{3}LE) and the triplet charge transfer state (^{3}CT), leading to inefficient vibronic coupling between these two triplet states.

Table S2. Summary of the photophysical data of selected compounds

Compound	Quantum Yield[a] (%)	Prompt Fluorescence Lifetime[a] (ns)	Delayed Fluorescence Lifetime[a] (μs)	Emission onset[a] (eV)	ΔE_{ST}[b] (eV)
**10(a)**	9.0	5.72	NA	3.05	0.07
**10(b)**	12.8	3.83	NA	3.00	0.17
**10(c)**	27.8	7.05	NA	3.37	0.01
**11(a)**	4.6	21.5	0.93	2.32	0.02
**11(b)**	5.2	18.2	1.41	2.31	0.01
**11(c)**	38.5	35.4	2.56	2.60	0.02
**12(a)**	7.0	3.72	NA	2.83	0.02
**12(b)**	4.0	0.60	NA	2.76	0.07

[a] The values are obtained from 1% of the emitters doped in Zeonex. NA indicated no delayed fluorescence was observed.

[b] Values are obtained in 2-MeTHF in 77K. All values for compounds 10, 11 and 12 are extracted from published articles [2-4]

3. Synthetic Procedure

3.1. Bromination of 1,4-bis(trifluoromethyl)benzene into 9(c)

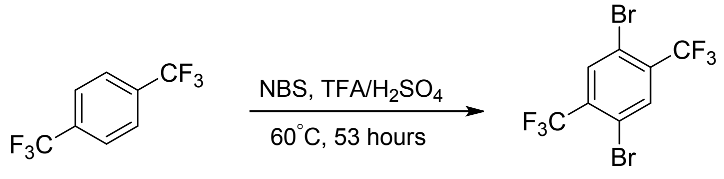

9(c)

1,4-dibromo-2,5-bis(trifluoromethyl)benzene, 9(c).[5] 1,4-bis(trifluoromethyl) benzene (2.36 g, 11.0 mmol 1.0 eq.) was dissolved in trifluoroacetic acid (TFA, 32 mL) and concentrated H_2SO_4 (7.5 mL) at room temperature. Then, *N*-bromosuccinimide (NBS, 5.89 g, 33.0 mmol, 3.0 eq.) was added in one portion. The resulting brown reaction mixture was heated to 60°C for 53 hours. The reaction mass was cooled down to room temperature before pouring into crushed ice. The mixture was left to stand until most of the ice has melted before filtering under vacuum. The solid was washed with water a few times and left under vacuum suction for 30-60 minutes to remove most of the water. The yellowish precipitate was continued to dry in a vacuum desiccator over P_2O_5 overnight. The product is pure enough to be used in the next step without any purification. Yield: 3.04 g (74%). **^{1}H NMR** (400 MHz, $CDCl_3$): δ 8.01 (s, 2H). **^{19}F NMR** (376 MHz, $CDCl_3$): δ -63.49. **GC-MS** (EI) *m/z* calcd for $C_8H_2Br_2F_6$: 371.84 $[M]^+$; found 371.90. The spectral data is consistent with reported data in literature.[6]

3.2. Microwave-assisted, Two-fold Buchwald-Hartwig Coupling

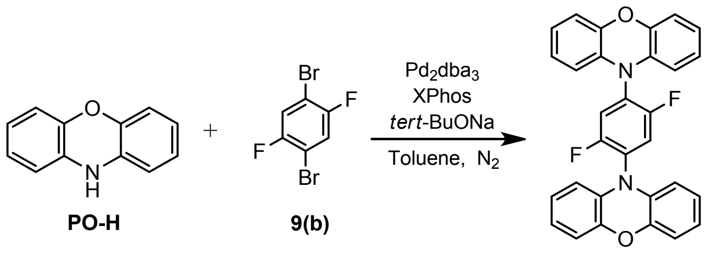

10,10'-(2,5-difluoro-1,4-phenylene)bis(10*H*-phenoxazine) (**10(a)**). Following **GP1**. **9(b)** (1.0 g, 3.69 mmol, 1.0 eq.), **PO-H** (1.48 g, 8.09 mmol, 2.2 eq.), $Pd_2(dba)_3$ (0.17 g, 0.18 mmol, 5 mol%), XPhos (0.12 g, 0.26 mmol, 7 mol%), sodium *tert*-butoxide (0.78 g, 8.08 mmol, 2.2 eq.), and dry toluene (10 mL). The crude material was purified *via* column chromatography using *n*-hexane/DCM (5:1 v/v) as the eluent followed by recrystallization from *n*-hexane/DCM to afford the product as yellow crystals. Yield: 1.18 g (68%).[3]

Following **GP2**. **9(b)** (0.50 g, 1.84 mmol, 1.0 eq.), **PO-H** (0.70 g, 3.80 mmol, 2.2 eq.), $Pd_2(dba)_3$ (84 mg, 91 μmol, 5 mol%), XPhos (61 mg, 0.13 mmol, 7 mol%), sodium *tert*-butoxide (0.39 g, 4.04 mmol, 2.2 eq.), and dry toluene (10 mL). The crude material was purified *via* column chromatography using *n*-hexane/THF (4:1 v/v) as the eluent to afford the product as yellow powder. Yield: 0.78 g (89%). **m.p.** 259°C. **^{1}H NMR** (400 MHz, $CDCl_3$):

δ 7.38 (t, J = 7.9 Hz, 2H), 6.77–6.65 (m, 12H), 6.00 (d, J = 7.0 Hz, 4H). **^{13}C NMR** (100 MHz, $CDCl_3$): δ 158.8, 156.3, 144.0, 132.4, 127.5, 123.5, 122.6, 122.0, 116.1, 112.8. **MS** (ESI) *m/z* calcd for $C_{30}H_{18}F_2N_2O_2$: 476.13 $[M]^+$; found 475.89.

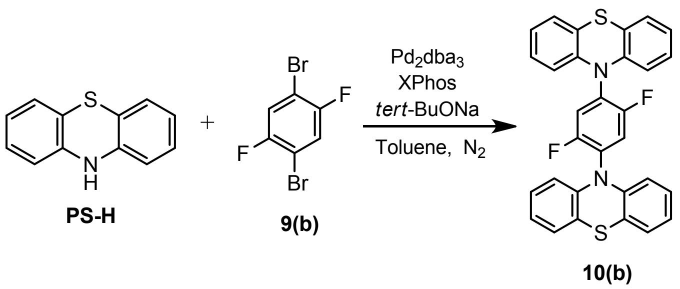

10,10'-(2,5-difluoro-1,4-phenylene)bis(10*H*-phenothiazine) (**10(b)**). Following **GP1**. **9(b)** (0.50 g, 1.84 mmol, 1.0 eq.), **PS-H** (0.81 g, 4.04 mmol, 2.2 eq.), $Pd_2(dba)_3$ (84 mg, 91 µmol, 5 mol%), XPhos (61 mg, 0.13 mmol, 7 mol%), sodium *tert*-butoxide (0.39 g, 4.04 mmol, 2.2 eq.), and dry toluene (10 mL). The crude material was purified *via* column chromatography using *n*-hexane/DCM (5:1 v/v) as the eluent to afford the product as white powder. Yield: 0.4g (40%).[3]

Following **GP2**. **10(b)** (0.50 g, 1.84 mmol, 1.0 eq.), **PS-H** (0.81 g, 4.04 mmol, 2.2 eq.), $Pd_2(dba)_3$ (84 mg, 91 µmol, 5 mol%), XPhos (61 mg, 0.13 mmol, 7 mol%), sodium *tert*-butoxide (0.39 g, 4.04 mmol, 2.2 eq.), and dry toluene (10 mL). The crude material was purified *via* column chromatography using *n*-hexane/THF (4:1 v/v) as the eluent to afford the product as white powder. Yield: 0.78 g (89%). **m.p.** 258°C. **^{1}H NMR** (400 MHz, $CDCl_3$): δ 7.45 (t, J = 7.9 Hz, 2H), 7.03 (d, J = 7.5 Hz, 4H), 6.99–6.89 (m, 4H), 6.85 (t, J = 7.2 Hz, 4H), 6.27 (d, J = 8.1 Hz, 4H). **^{13}C NMR** (100 MHz, $CDCl_3$): δ 159.4, 156.8, 142.7, 130.1, 127.3, 127.2, 123.5, 121.4, 115.4. **MS** (ESI) *m/z* calcd for $C_{30}H_{18}F_2N_2S_2$: 508.09 $[M]^+$; found 507.97.

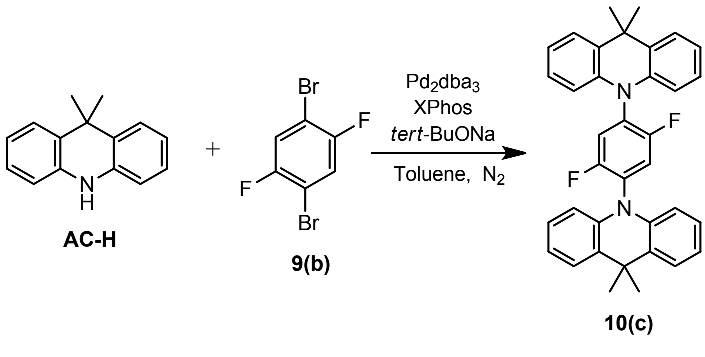

10,10'-(2,5-difluoro-1,4-phenylene)bis(9,9-dimethyl-9,10-dihydroacridine) (**10(c)**). Following **GP1**. **9(b)** (0.50 g, 1.84 mmol, 1.0 eq.), **AC-H** (0.85 g, 4.04 mmol, 2.2 eq.), $Pd_2(dba)_3$ (84 mg, 91 µmol, 5 mol%), XPhos (61 mg, 0.13 mmol, 7 mol%), sodium *tert*-butoxide (0.39 g, 4.04 mmol, 2.2 eq.), and dry toluene (10 mL). The crude material was

purified *via* column chromatography using *n*-hexane/DCM (5:1 v/v) as an eluent to afford the product as white powder. Yield: 0.28 g (27%).[3]

Following **GP2**. **9(b)** (0.50 g, 1.84 mmol, 1.0 eq.), **AC-H** (0.85 g, 4.04 mmol, 2.2 eq.), $Pd_2(dba)_3$ (84 mg, 91 μmol, 5 mol%), XPhos (61 mg, 0.13 mmol, 7 mol%), sodium *tert*-butoxide (0.39 g, 4.04 mmol, 2.2 eq.), and dry toluene (10 mL). The crude material was purified *via* column chromatography using *n*-hexane/THF (4:1 v/v) as an eluent to afford the product as white powder. Yield: 0.85 g (87%). **m.p.** 338°C. **^{1}H NMR** (400 MHz, $CDCl_3$): δ 7.45 (d, *J* = 7.7 Hz, 4H), 7.41 (t, *J* = 7.9 Hz, 2H), 7.07 (d, *J* = 7.6 Hz, 4H), 6.97 (t, *J* = 7.4 Hz, 4H), 6.41 (d, *J* = 8.1 Hz, 4H), 1.65 (s, 12H, CH_3). **^{13}C NMR** (100 MHz, $CDCl_3$): δ 158.8, 139.5, 130.9, 129.5, 126.8, 125.5, 122.2, 121.7, 113.2, 36.1, 30.8. **MS** (ESI) *m/z* calcd for $C_{36}H_{30}F_2N_2$: 528.24 $[M]^+$; found 528.73.

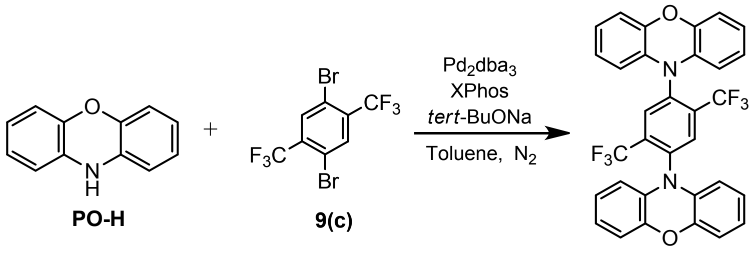

10,10'-(2,5-bis(trifluoromethyl)-1,4-phenylene)bis(10*H*-phenoxazine) (**11(a)**). Following **GP1.** **9(c)** (0.50 g, 1.34 mmol, 1.0 eq.), **PO-H** (0.54 g, 2.95 mmol, 2.2 eq.), $Pd_2(dba)_3$ (60 mg, 70 μmol), XPhos (40 mg, 90 μmmol), sodium *tert*-butoxide (0.28 g, 2.96 mmol) and dry toluene (10 mL). after cooling down, the crude material was filtered and washed with water, ethyl acetate, acetone and DCM to obtain yellow powder. The yellow powder was recrystallized from THF to afford the product. Yield: 56 % (0.43 g).[4]

Following **GP2.** **9(c)** (0.2g, 0.53 mmol, 1.0 eq.), **PO-H** (0.21 g, 1.18 mmol, 2.2 eq.), $Pd_2(dba)_3$ (18 mg, 20 μmol, 5 mol%), XPhos (10 mg, 30 μmol, 7 mol%), sodium *tert*-butoxide (0.113 g, 1.18 mmol, 2.2 eq.) and dry toluene (15 mL). After cooling down, the crude material was filtered and washed with water, ethyl acetate, acetone and DCM to obtain yellow powder. The yellow powder was recrystallized from THF to afford the product. Yield: 71 % (0.22 g). **m.p.** 386^{0}C. **^{1}H NMR** (400 MHz, $CDCl_3$): δ 8.02 (s, 2H), 6.74 (m, 12H), 5.68 (d, *J* = 7.7 Hz, 4H). **^{13}C NMR** spectrum was not recorded due to the low solubility of the material. Thus, X-ray data was obtained instead to confirm the structure.[4] **MS** (ESI) *m/z* calcd for $C_{32}H_{18}F_6N_2O_2$: 576.13 $[M]^+$; found 576.01.

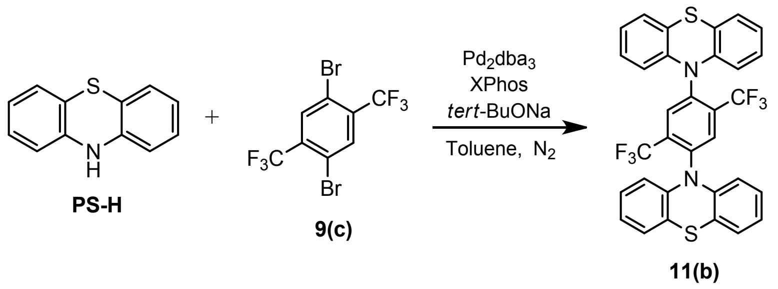

10,10'-(2,5-bis(trifluoromethyl)-1,4-phenylene)bis(10*H*-phenothiazine) (**11(b)**). Following **GP1**. **9(c)** (0.50 g, 1.34 mmol, 1.0 eq.), **PS-H** (0.59 g, 2.96 mmol, 2.2 eq.), $Pd_2(dba)_3$ (60 mg, 70 µmol, 5 mol%), XPhos (40 mg, 90 µmol, 7 mol%), sodium *tert*-butoxide (0.28 g, 2.96 mmol, 2.2 eq.), and dry toluene (10 mL). The crude material was purified *via* column chromatography using *n*-hexane/DCM (3:2 v/v) as the eluent to afford the product as yellow powder. Yield: 0.40 g (49%).[4]

Following **GP2. 9(c)** (0.2 g, 0.53 mmol, 1.0 eq.), **PS-H** (0.23 g, 1.18 mmol, 2.2 eq.), $Pd_2(dba)_3$ (0.02 g, 0.02 mmol), XPhos (0.017 g, 0.03 mmol), sodium *tert*-butoxide (0.113 g, 1.18 mmol), and dry toluene (15 mL). The crude material was purified *via* column chromatography using *n*-hexane/DCM (3:2 v/v) as the eluent to afford the product as yellow powder. Yield: 0.19 g (58%). **m.p.** 321°C. **^{1}H NMR** (400 MHz, $CDCl_3$): δ 8.15 (s, 2H), 7.07 (dd, *J* = 7.5, 1.3 Hz, 4H), 6.97 – 6.79 (m, 8H), 6.02 (d, *J* = 7.9 Hz, 4H). **^{13}C NMR** (100 MHz, $CDCl_3$): δ 143.4, 127.4, 127.1, 123.6, 121.0. **MS** (ESI) *m/z* calcd for $C_{32}H_{18}F_6N_2S_2$: 608.08 $[M]^+$; found 608.60.

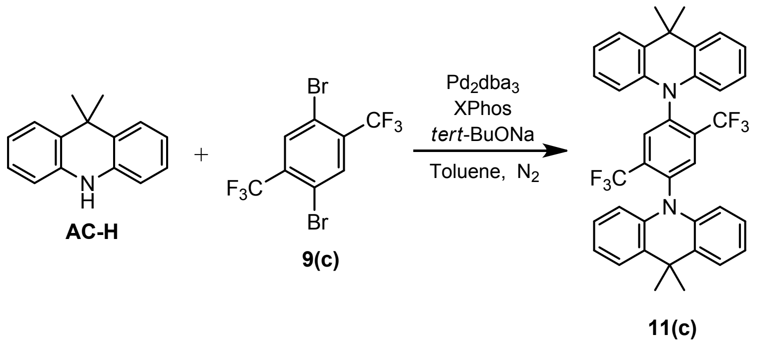

10,10'-(2,5-bis(trifluoromethyl)-1,4-phenylene)bis(9,9-dimethyl-9,10-dihydroacridine) (**11(c)**). Following **GP1**. **9(c)** (0.50 g, 1.34 mmol, 1.0 eq.), **AC-H** (0.62 g, 2.96 mmol, 2.2 eq.), $Pd_2(dba)_3$ (60 mg, 70 µmol, 5 mol%), XPhos (40 mg, 90 µmol, 7 mol%), sodium *tert*-butoxide (0.28 g, 2.96 mmol) and dry toluene (10 mL). The crude material was purified *via* column chromatography using *n*-hexane/DCM (3:2 v/v) as the eluent to afford the product as yellow powder. Yield: 0.40 g (47%).[4]

Following GP2. **9(c)** (1.00 g, 2.68 mmol, 1.0 eq.), **AC-H** (1.20 g, 5.91 mmol, 2.2 eq.), $Pd_2(dba)_3$ (0.12 g, 0.13 mmol), XPhos (0.08 mg, 0.19 mmol), sodium *tert*-butoxide (0.56 g, 5.9 mmol) and dry toluene (15 mL). The crude material was purified via column chromatography using *n*-hexane/THF (4:1 v/v) as the eluent to afford the product as yellow powder. Yield: 1.2 g (70%). **m.p.** 311°C. **^{1}H NMR** (400 MHz, $CDCl_3$): δ 7.99 (s, 2H), 7.49 (d, *J* = 7.6 Hz, 4H), 7.06 (t, *J* = 7.6 Hz, 4H), 6.99 (t, *J* = 7.4 Hz, 4H), 6.06 (d, *J* = 8.0 Hz, 4H),

1.90 (s, 6H, CH_3), 1.41 (s, 6H, CH_3).**^{13}C NMR** (100 MHz, $CDCl_3$): δ 140.6, 135.7, 130.5, 126.7, 125.5, 121.9, 113.6, 36.1, 27.4. **MS** (ESI) *m/z* calcd for $C_{38}H_{30}F_6N_2$: 628.23 $[M]^+$; found 628.38.

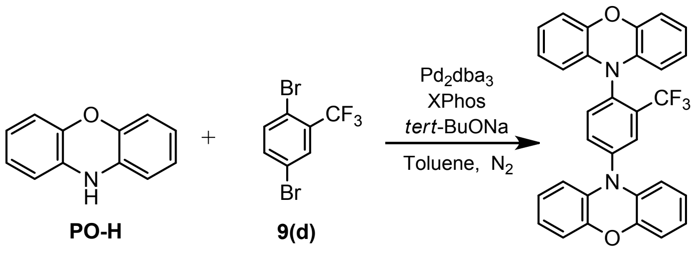

PO-H 9(d) 12(a)

10,10'-(2-(trifluoromethyl)-1,4-phenylene)bis(10*H*-phenoxazine) (**12(a)**). Following **GP1. 9(d)** (1.00 g, 3.29 mmol, 1.0 eq.), **PO-H** (1.33 g, 7.24 mmol, 2.2 eq.), $Pd_2(dba)_3$ (0.15 g, 0.16 mmol, 5 mol%), XPhos (0.11 g, 0.23 mmol, 7 mol%), sodium *tert*-butoxide (0.70 g, 7.23 mmol, 2.2 eq.) and dry toluene (15 mL). The crude material was purified *via* column chromatography using *n*-hexane/DCM (5:1 v/v) as an eluent to afford the product as yellow powder. Yield: 1.37 g (80%).[2]

Following **GP2. 9(d)** (0.50 g, 1.64 mmol, 1.0 eq.), **PO-H** (0.66 g, 3.60 mmol, 2.2 eq.), $Pd_2(dba)_3$ (70 mg, 80 µmol, 5 mol%), XPhos (50 mg, 0.12 mmol, 7 mol%), sodium *tert*-butoxide (0.34 g, 3.60 mmol, 2.2 eq.) and dry toluene (15 mL). The crude material was purified *via* column chromatography using *n*-hexane/DCM (5:1 v/v) as an eluent to afford the product as yellow powder. Yield: 0.75 g (89%). **m.p.** 135°C. **^{1}H NMR** (400 MHz, $CDCl_3$): δ 7.95 (s, 1H), 7.85 (dd, *J* = 8.3, 1.7 Hz, 1H), 7.73 (d, *J* = 8.3 Hz, 1H), 6.82 – 6.64 (m, 12H), 6.00 (d, *J* = 7.7 Hz, 2H), 5.82 (d, *J* = 7.7 Hz, 2H). **^{13}C NMR** (100 MHz, $CDCl_3$): δ 144.1, 143.8, 140.2, 137.9, 137.7, 136.7, 135.6, 133.8, 133.39, 131.3, 123.4, 123.3, 122.4, 122.2, 116.1, 115.8, 113.7, 113.2. **MS** (ESI) *m/z* calcd for $C_{31}H_{19}F_3N_2O_2$: 508.50 $[M]^+$; found 508.24.

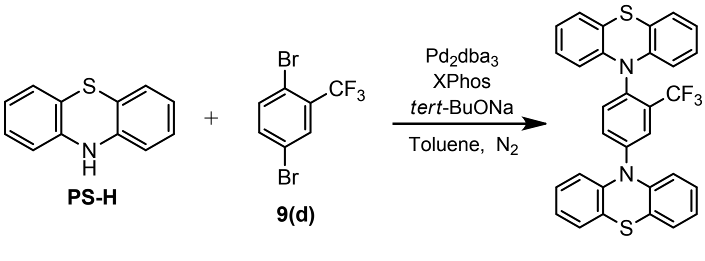

PS-H 9(d) 12(b)

10,10'-(2-(trifluoromethyl)-1,4-phenylene)bis(10*H*-phenothiazine) (**12(b)**). Following **GP1**. **9(d)** (1.00 g, 3.29 mmol, 10 eq.), **PS-H** (1.44 g, 7.24 mmol, 2.2 eq.), $Pd_2(dba)_3$ (0.15 g, 0.16 mmol, 5 mol%), XPhos (0.11 g, 0.23 mmol, 7 mol%), sodium *tert*-butoxide (0.70 g, 7.23 mmol, 2.2 eq.) and dry toluene (15 mL). The crude material was purified *via* column

chromatography using *n*-hexane/DCM (5:1 v/v) as an eluent to afford the product as white powder. Yield: 0.67 g (37%).[2]

Following **GP2**. **9(d)** (0.50 g, 1.64 mmol, 1.0 eq.), **PS-H** (0.72 g, 3.61 mmol, 2.2 eq.), $Pd_2(dba)_3$ (70 mg, 80 µmol, 5 mol%), XPhos (50 mg, 0.12 mmol, 7 mol%), sodium *tert*-butoxide (0.34 g, 3.60 mmol, 2.2 eq.) and dry toluene (15 mL). The crude material was purified *via* column chromatography using *n*-hexane/DCM (5:1 v/v) as an eluent to afford the product as white powder. Yield: 0.50 g (56%). **m.p.** 90°C. **^{1}H NMR** (400 MHz, $CDCl_3$): δ 7.68 (d, J = 2.5 Hz, 1H), 7.53 (dd, J = 8.6, 2.5 Hz, 1H), 7.46 (d, J = 8.6 Hz, 1H), 7.40 (d, J = 7.7 Hz, 2H), 7.28 (s, 2H), 7.16 (dd, J = 13.2, 7.6 Hz, 4H), 7.04 (dd, J = 7.2, 1.5 Hz, 2H), 6.94 – 6.78 (m, 4H), 6.14 (d, J = 7.9 Hz, 2H). **^{13}C NMR** (100 MHz, $CDCl_3$): δ 144.8, 144.1, 141.8, 136.2, 132.9, 132.1, 130.4, 128.5, 127.4, 126.8, 126.7, 125.6, 124.3, 123.7, 122.7, 120.1, 119.9, 115.3. **MS** (ESI) *m/z* calcd for $C_{31}H_{19}F_3N_2S_2$: 540.09 $[M]^+$; found 540.21.

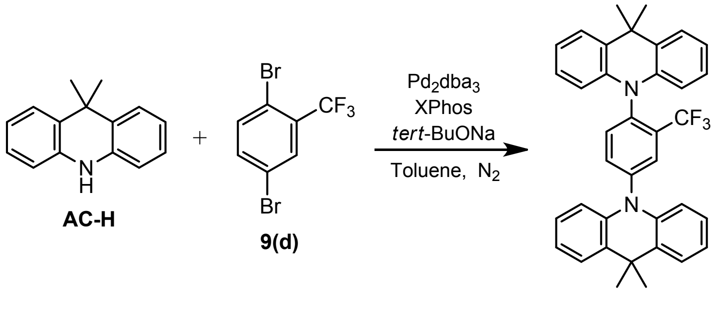

10,10'-(2-(trifluoromethyl)-1,4-phenylene)bis(9,9-dimethyl-9,10-dihydroacridine) (**12(c)**). Following **GP1**. **9(d)** (1.00 g, 3.29 mmol, 1.0 eq.), **AC-H** (1.52 g, 7.24 mmol, 2.2 eq.), $Pd_2(dba)_3$ (0.15 g, 0.16 mmol, 5 mol%), XPhos (0.11 g, 0.23 mmol, 7 mol%), sodium *tert*-butoxide (0.70 g, 7.23 mmol, 2.2 eq.) and dry toluene (10 mL). The crude material was purified *via* column chromatography using *n*-hexane/DCM (5:1 v/v) as an eluent to afford the product as white powder. Yield: 0.25 g (13%).[2]

Following **GP2**. **9(d)** (0.50 g, 1.64 mmol, 1.0 eq.), **AC-H** (0.75 g, 3.61 mmol, 2.2 eq.), $Pd_2(dba)_3$ (70 mg, 80 µmol, 5 mol%), XPhos (50 mg, 0.11 mmol, 7 mmol%), sodium *tert*-butoxide (0.34 g, 3.61 mmol, 2.2 eq.) and dry toluene (10 mL). The crude material was purified *via* column chromatography using *n*-hexane/DCM (5:1 v/v) as an eluent to afford the product as white powder. Yield: 0.70 g (76%). **m.p.** 84°C. **^{1}H NMR** (400 MHz, $CDCl_3$): δ 7.99 (s, 1H), 7.86 (d, *J* = 8.2 Hz, 1H), 7.69 (d, *J* = 8.3 Hz, 1H), 7.55 (d, *J* = 7.6 Hz, 4H), 7.17 – 7.01 (m, 8H), 6.42 (d, *J* = 8.1 Hz, 2H), 6.23 (d, *J* = 8.0 Hz, 2H), 1.97 (s, 3H), 1.75 (s, 6H), 1.48 (s, 3H). **^{13}C NMR** (100 MHz, $CDCl_3$): δ 141.98, 141.18, 140.50, 139.65, 137.73, 137.47, 131.88, 131.83, 130.96, 130.50, 126.81, 126.57, 125.68, 125.30, 121.64, 121.52, 114.12, 113.95, 36.26, 36.21, 34.48, 31.13, 27.24. **MS** (ESI) *m/z* calcd for $C_{37}H_{31}F_3N_2$: 560.66 $[M]^+$; found 560.57.

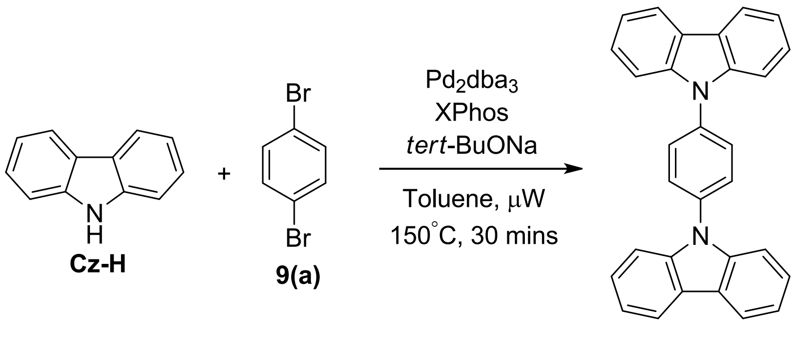

13(a)

1,4-di(9*H*-carbazol-9-yl)benzene (**13(a)**) Following **GP3**. **9(a)** (0.12 g, 0.5 mmol, 1.0 eq.), **Cz-H** (0.18 g, 1.05 mmol, 2.1 eq.), $Pd_2(dba)_3$ (23 mg, 25μmol, 5 mol%), XPhos (24 mg, 50 μL, 10 mol%), sodium *tert*-butoxide (0.12 g, 1.25 mmol, 2.5 eq.), and toluene (2 mL). The crude material was purified *via* column chromatography using *n*-hexane/$CHCl_3$ (9:1 v/v) as the eluent followed by recrystallization from MeOH/$CHCl_3$ to afford the product as white solid. Yield: 0.18 g (86%). **m.p.** 312-313°C. **^{1}H NMR** (500 MHz, $CDCl_3$): δ 8.20 (d, *J* = 7.5 Hz, 4H), 7.83 (s, 4H), 7.58 (d, *J* = 8.0 Hz, 4H), 7.49 (t, *J* = 8.0 Hz, 4H), 7.35 (t, *J* = 7.5 Hz, 4H). **^{13}C NMR** (125 MHz, $CDCl_3$): δ 140.7, 136.6, 128.3, 126.1, 123.5, 120.4, 120.3, 109.7. **HRMS** (ESI/Q-TOF) *m/z* calcd for $C_{30}H_{20}N_2$: 409.1626 $[M]^+$; found 409.1633.

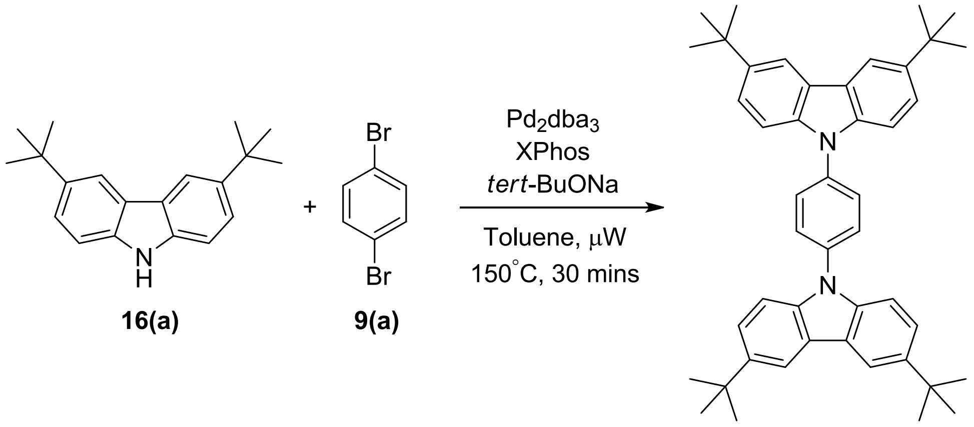

13(b)

1,4-bis(3,6-di-*tert*-butyl-9*H*-carbazol-9-yl)benzene (**13(b)**) Following **GP3**. **9(a)** (0.12 g, 0.5 mmol, 1.0 eq.), **16(a)** (0.29 g, 1.05 mmol, 2.1 eq.), $Pd_2(dba)_3$ (23 mg, 25μmol, 5 mol%), XPhos (24 mg, 50 μL, 10 mol%), sodium *tert*-butoxide (0.12 g, 1.25 mmol, 2.5 eq.), and toluene (2 mL). The crude material was recrystallized from MeOH/$CHCl_3$ to afford the product as white solid. Yield: 0.28 g (89%). **m.p.** 348-350°C. **^{1}H NMR** (500 MHz, $CDCl_3$): δ 8.18 (d, *J* = 1.5 Hz, 4H), 7.78 (s, 4H), 7.53 (dd, *J* = 8.0 Hz, 4H), 7.49 (d, *J* = 8.5 Hz, 4H), 1.50 (s, 36H). **^{13}C NMR** (125 MHz, $CDCl_3$): δ 143.1, 139.1, 136.7, 127.8, 123.7, 123.5, 116.3, 109.2, 34.8, 32.0. **HRMS** (ESI/Q-TOF) *m/z* calcd for $C_{46}H_{52}N_2$: 632.4130 $[M]^+$; found 632.4123.

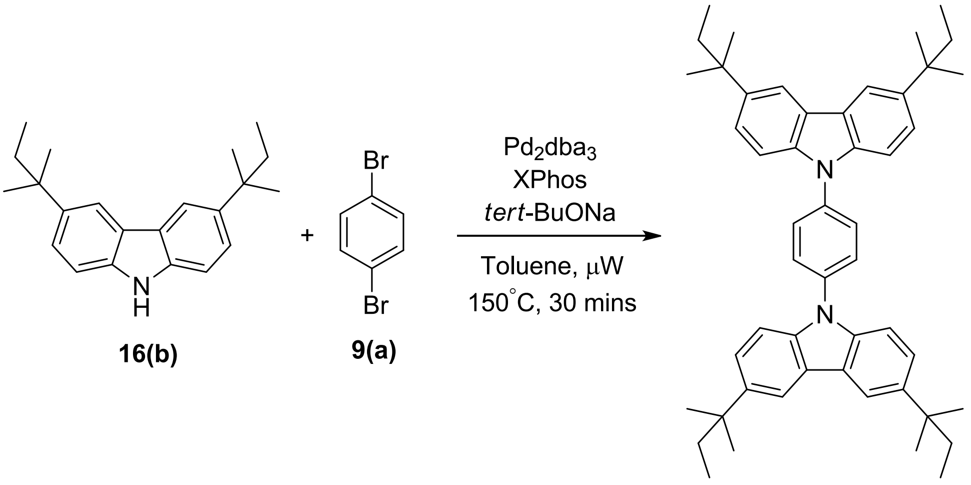

16(b) 9(a) 13(c)

1,4-bis(3,6-di-*tert*-pentyl-9*H*-carbazol-9-yl)benzene (**13(c)**) Following **GP3**. **9(a)** (0.12 g, 0.5 mmol, 1.0 eq.), **16(b)** (0.32 g, 1.05 mmol, 2.1 eq.), $Pd_2(dba)_3$ (23 mg, 25µmol, 5 mol%), XPhos (24 mg, 50 µL, 10 mol%), sodium *tert*-butoxide (0.12 g, 1.25 mmol, 2.5 eq.), and toluene (2 mL). The crude material was purified *via* column chromatography using *n*-hexane as the eluent to afford a colourless, viscous oil. The oil was sonicated in methanol followed by the addition of minimal toluene to precipitate the product as flocculent, white solid. Yield: 0.31 g (90%). **m.p.** 280-281°C. **^{1}H NMR** (500 MHz, $CDCl_3$): δ 8.12 (d, *J* = 1.5 Hz, 4H), 7.79 (s, 4H), 7.50 (d, *J* = 8.5 Hz, 4H), 7.45 (dd, *J* = 8.5, 1.5 Hz, 4H), 1.80 (q, *J* = 7.5 Hz, 8H), 1.46 (s, 24H), 0.75 (6.5 Hz, 12H). **^{13}C NMR** (125 MHz, $CDCl_3$): δ 141.3, 139.0, 136.6, 127.7, 124.7, 124.2, 123.4, 117.2, 109.1, 38.0, 37.4, 29.1, 9.3. **HRMS** (ESI/Q-TOF) *m/z* calcd for $C_{50}H_{60}N_2$: 688.4756 $[M]^+$; found 688.4773.

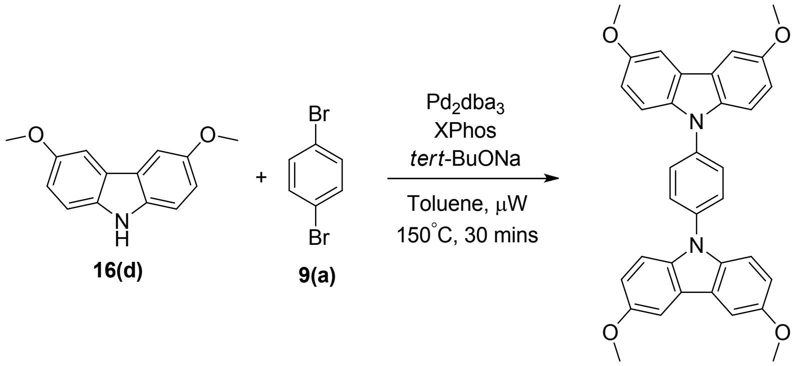

16(d) 9(a) 13(d)

1,4-bis(3,6-dimethoxy-9*H*-carbazol-9-yl)benzene (**13(d)**) Following **GP3**. **9(a)** (0.12 g, 0.50 mmol, 1.0 eq.), **16(d)** (0.24 g, 1.05 mmol, 2.1 eq.), $Pd_2(dba)_3$ (23 mg, 25 µmol, 5 mol%), XPhos (24 mg, 50 µmol, 10 mol%), sodium *tert*-butoxide (0.12 g, 1.25 mmol, 2.5 eq.), and toluene (2 mL). The residue was recrystallized from *n*-hexane/acetone to afford the product as off-white solid. Yield: 84 mg (32%). **m.p.** 257-259°C. **^{1}H NMR** (500 MHz): δ 7.76 (s, 4H), 7.58 (d, *J* = 2.5 Hz, 4H), 7.47 (d, *J* = 8.5 Hz, 4H), 7.09 (dd, *J* = 9.0, 3.0 Hz, 4H), 3.40 (s, 12H).

^{13}**C NMR** (125 MHz, $CDCl_3$): δ 154.2, 136.6, 136.1, 127.8, 123.8, 115.3, 110.7, 102.9, 56.1. **HRMS** (ESI/Q-TOF) *m/z* calcd for $C_{34}H_{28}N_2O_4$: 528.2049 $[M]^+$; found 528.2042.

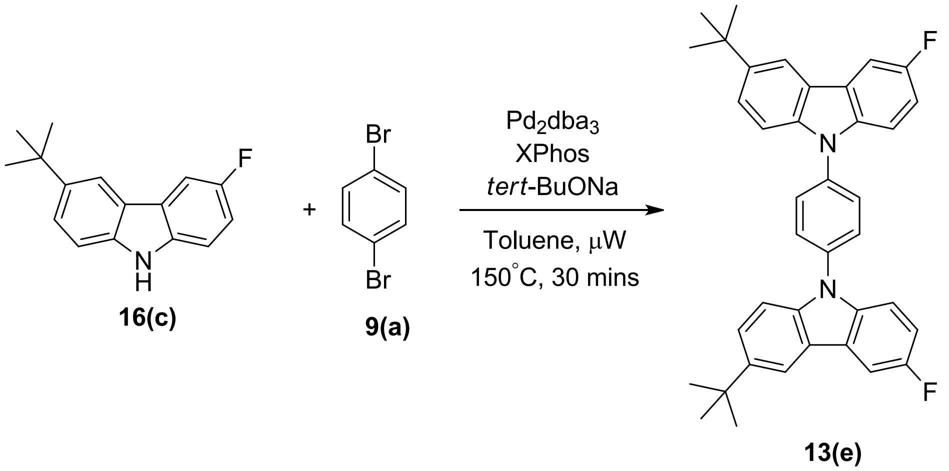

16(c) **9(a)** **13(e)**

1,4-bis(3-(*tert*-butyl)-6-fluoro-9*H*-carbazol-9-yl)benzene (**13(e)**) Following **GP3**. **9(a)** (0.12 g, 0.50 mmol, 1.0 eq.), **16(c)** (0.25 g, 1.05 mmol, 2.1 eq.), $Pd_2(dba)_3$ (23 mg, 25 μmol, 5 mol%), XPhos (24 mg, 50 μL, 10 mol%), sodium *tert*-butoxide (0.12 g, 1.25 mmol, 2.5 eq.), and toluene (2 mL). The crude material was recrystallized from MeOH/$CHCl_3$ to afford the product as off-white solid. Yield: 0.22 g (80%). **m.p.** 170-172°C. ^{1}**H NMR** (500 MHz, $CDCl_3$): δ 8.12 (d, *J* = 1.5 Hz, 2H), 7.84 (dd, *J* = 9.0, 3.0 Hz, 2H), 7.78 (s, 4H), 7.58 (dd, *J* = 9.0, 1.5 Hz, 2H), 7.50 (d, *J* = 8.5 Hz), 7.47 (dd, *J* = 8.5, 4.0 Hz, 2H), 7.18 (td, *J* = 9.0, 3.0 Hz, 2H), 1.48 (s, 18H). ^{13}**C NMR** (125 MHz, $CDCl_3$): δ 157.9 (d, *J* = 234.9 Hz), 143.5, 139.6, 137.3, 136.6, 128.1, 124.7, 124.4 (d, *J* = 9.5 Hz), 122.8 (d, *J* = 3.6 Hz), 116.7, 113.5 (d, *J* = 25.1 Hz), 110.3 (d, *J* = 9.5 Hz), 109.5, 10.60 (d, *J* = 23.8 Hz), 34.8, 31.9. ^{19}**F NMR** (471 MHz, $CDCl_3$): δ -123.5. **HRMS** (ESI/Q-TOF) *m/z* calcd for $C_{38}H_{34}N_2F_2$: 556.2690 $[M]^+$; found 556.2676.

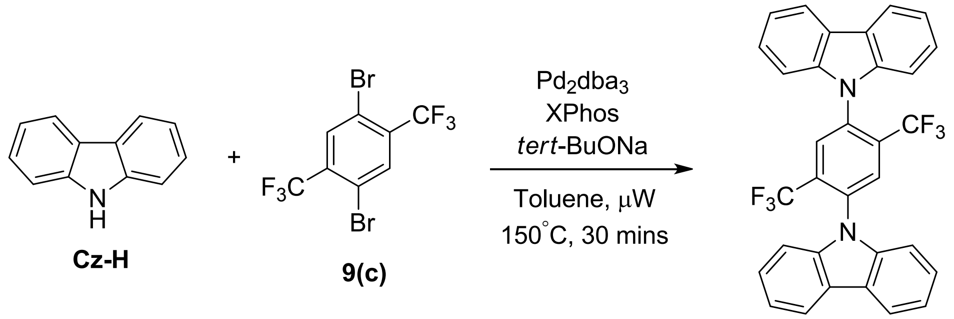

Cz-H **9(c)** **13(f)**

9,9'-(2,5-bis(trifluoromethyl)-1,4-phenylene)bis(9*H*-carbazole) (**13(f)**). Following **GP3**. **9(c)** (0.19 g, 0.50 mmol, 1.0 eq.), **Cz-H** (0.18 g, 1.05 mmol, 2.1 eq.), $Pd_2(dba)_3$ (24 mg, 25 μmol, 5 mol%), XPhos (25 mg, 50 μmol, 10 mol%), sodium *tert*-butoxide (0.12 g, 1.25 mmol, 2.5 eq.), and toluene (2 mL). **Note: 13(f)** did not form. Only one spot observed during TLC analysis of the reaction mass. Upon isolation and characterization by NMR experiments of the spot, it belongs to unreacted carbazole.

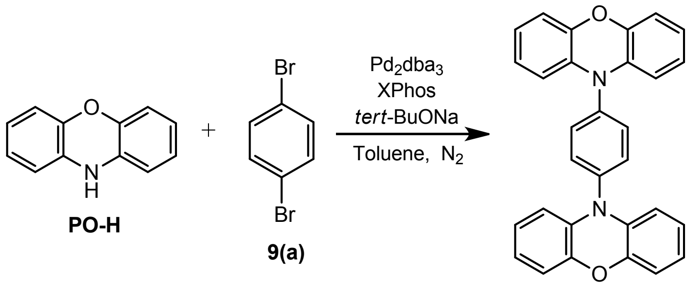

1,4-di(10*H*-phenoxazin-10-yl)benzene (**14(a)**). Following **GP2**. **9(a)** (0.5 g, 2.12 mmol, 1.0 eq.), **PO-H** (0.85 g, 4.63 mmol, 2.2 eq.), $Pd_2(dba)_3$ (0.09 g, 98 μmol, 5 mol%), XPhos (0.07 g, 0.14 mmol, 7 mol%), sodium *tert*-butoxide (0.40 g, 4.23 mmol, 2.2 eq.), and dry toluene (15 mL). The crude material was purified *via* column chromatography using *n*-hexane/ethyl acetate (4:1 v/v) as the eluent to afford the product as white powder. Yield: 0.85 g (91%). **m.p.** 249°C. **^{1}H NMR** (400 MHz, $CDCl_3$): δ 7.59 (s, 4H), 6.77 – 6.64 (m, 12H), 6.08 – 5.97 (m, 4H). **^{13}C NMR** (100 MHz, $CDCl_3$): δ 144.16, 139.19, 134.16, 133.84, 123.48, 121.87, 115.83, 113.31. **MS** (ESI) *m/z* calcd for $C_{30}H_{20}N_2O_2$: 440.15 $[M]^+$; found 439.95.[7]

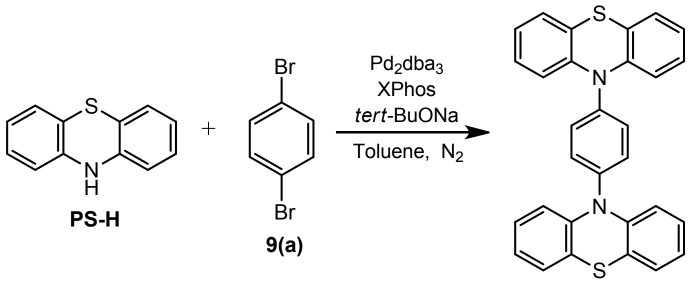

1,4-di(10*H*-phenothiazin-10-yl)benzene (**14(b)**) Following **GP2**. **9(a)** (0.5 g, 2.12 mmol, 1.0 eq.), **PS-H** (0.92 g, 4.61 mmol, 2.2 eq.), $Pd_2(dba)_3$ (0.09 g, 98 μmol, 5 mol%), XPhos (0.07 g, 0.14 mmol, 7 mol%), sodium *tert*-butoxide (0.40 g, 4.23 mmol, 2.2 eq.), and dry toluene (15 mL). The crude material was purified *via* column chromatography using *n*-hexane/ethyl acetate (4:1 v/v) as the eluent to afford the product as white powder. Yield: 0.94 g (94%). **m.p.** 259°C. **^{1}H NMR** (400 MHz, DMSO-d_6): δ 7.54 (s, 4H), 7.19 (d, J = 7.5 Hz, 4H), 7.09 (t, J = 7.1 Hz, 4H), 6.98 (t, J = 7.2 Hz, 4H), 6.55 (d, J = 7.9 Hz, 4H). **^{13}C NMR** (100 MHz, DMSO-d_6): δ 143.19, 130.08, 127.58, 127.14, 123.53, 121.83, 118.15. **MS** (ESI) *m/z* calcd for $C_{30}H_{20}N_2S_2$: 472.11 $[M]^+$; found 471.99.[8]

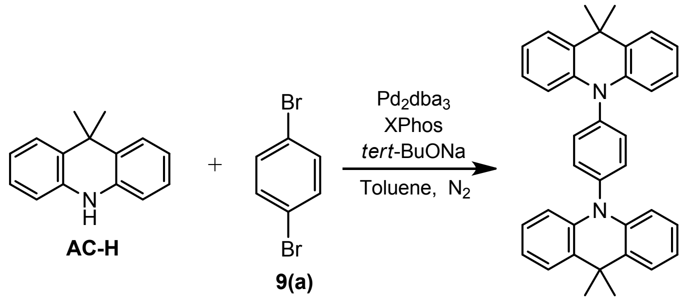

14(c)

1,4-bis(9,9-dimethylacridin-10(9*H*)-yl)benzene (**14(c)**) Following **GP2**. **9(a)** (0.5 g, 2.12 mmol, 1.0 eq.), **AC-H** (0.97 g, 4.63 mmol, 2.2 eq.), $Pd_2(dba)_3$ (0.09 g, 98 µmol, 5 mol%), XPhos (0.07 g, 0.14 mmol, 7 mol%), sodium *tert*-butoxide (0.40 g, 4.23 mmol, 2.2 eq.), and dry toluene (15 mL). The crude material was purified *via* column chromatography using *n*-hexane/ethyl acetate (4:1 v/v) as the eluent to afford the product as white powder. Yield: 0.83 g (83%). **^{1}H NMR** (400 MHz, $CDCl_3$): δ 7.61 (s, 4H), 7.50 (d, *J* = 7.7 Hz, 4H), 7.09 (t, *J* = 7.6 Hz, 4H), 6.99 (t, *J* = 7.4 Hz, 4H), 6.45 (d, *J* = 8.2 Hz, 4H), 1.72 (s, 12H). **^{13}C NMR** (100 MHz, $CDCl_3$): δ 141.12, 140.92, 134.09, 130.48, 126.63, 125.43, 121.04, 114.12, 36.19, 31.17. **MS** (ESI) *m/z* calcd for $C_{36}H_{32}N_2$: 492.26 $[M]^+$; found 492.43

3.3. Acidic condensation between substituted 1,2-phenylene diamine and benzil derivatives into quinoxaline skeleton

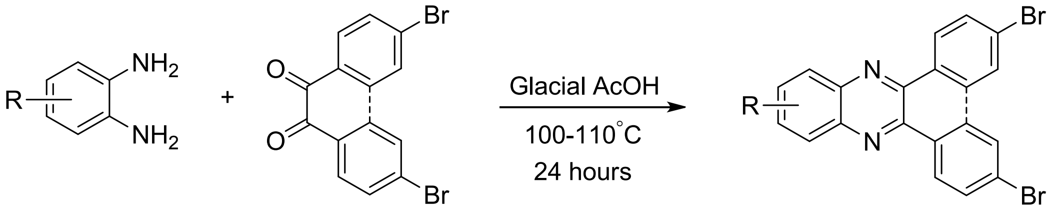

General procedure for acidic condensation: 1,2-phenylene diamine derivative and brominated benzil derivative were weighed into a reaction flask followed by the addition of glacial AcOH. The mixture was heated to 100-110°C for 24 hours. Then, after cooling down to room temperature, the reaction mass was poured into water to precipitate out the product. The precipitate was filtered and collected. The crude material was purified through column chromatography using appropriate solvent combination.

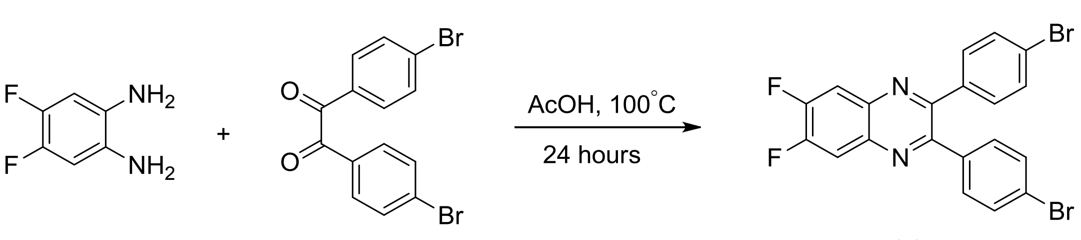

15(a)

2,3-bis(4-bromophenyl)-6,7-difluoroquinoxaline (**15(a)**). 4,5-difluorobenzene-1,2-diamine (2.54 g, 17.6 mmol, 1.3 eq.), 1,2-bis(4-bromophenyl)ethane-1,2-dione (5.00 g, 13.5 mmol, 1.0 eq.), and 100 mL glacial acetic acid. Combination of *n*-hexane/DCM (4:1 v/v) as the eluent

during column chromatography to obtain white solid followed by recrystallization to afford the pure product. Yield: 6.0 g (92%). **^{1}H NMR** (400 MHz, $CDCl_3$): δ 7.89 (t, *J* = 9.2 Hz, 2H), 7.51 (d, *J* = 8.1 Hz, 4H), 7.38 (d, *J* = 8.1 Hz, 4H). **^{13}C NMR** (100 MHz, $CDCl_3$): δ 154.30, 154.12, 152.29, 151.72, 151.54, 138.78, 138.72, 138.66, 137.23, 131.93, 131.50, 124.21, 115.04, 114.97, 114.91, 114.85. **MS** (ESI) *m/z* calcd for $C_{20}H_{10}Br_2F_2N_2$: 476.12 $[M]^+$; found 477.03.[9]

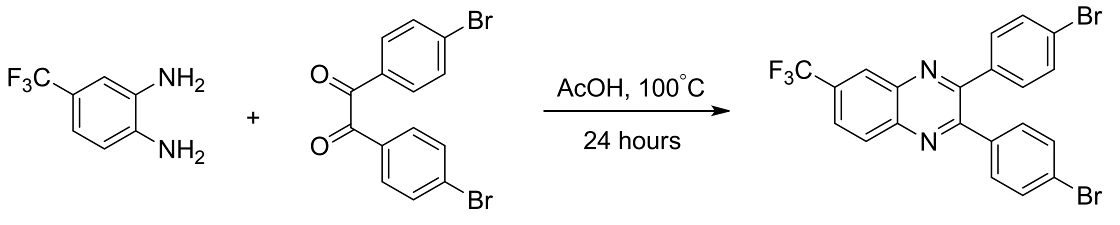

15(b)

2,3-bis(4-bromophenyl)-6-(trifluoromethyl)quinoxaline (**15(b)**). 4-(trifluoromethyl)benzene-1,2-diamine (1.72 g, 9.78 mmol, 1.3 eq.), 1,2-bis(4-bromophenyl)ethane-1,2-dione (3.00 g, 8.15 mmol, 1.0 eq.), and 70 mL glacial acetic acid. Combination of *n*-hexane/DCM (4:1 v/v) as the eluent during column chromatography. Isolated product appeared as white solid. Yield: 3.50 g (84%). **^{1}H NMR** (400 MHz, $CDCl_3$): δ 8.47 (s, 1H), 8.27 (d, *J* = 8.7 Hz, 1H), 7.96 (d, *J* = 8.8 Hz, 1H), 7.53 (d, *J* = 8.2 Hz, 4H), 7.43 (dd, *J* = 8.0, 3.5 Hz, 4H). **^{13}C NMR** (100 MHz, $CDCl_3$): δ 154.03, 153.46, 142.35, 140.34, 137.20, 137.15, 131.99, 131.57, 131.53, 130.59, 127.43, 126.12, 124.53, 124.45. **MS** (ESI) *m/z* calcd for $C_{21}H_{11}Br_2F_3N_2$: 508.14 $[M]^+$; found 508.98.[10]

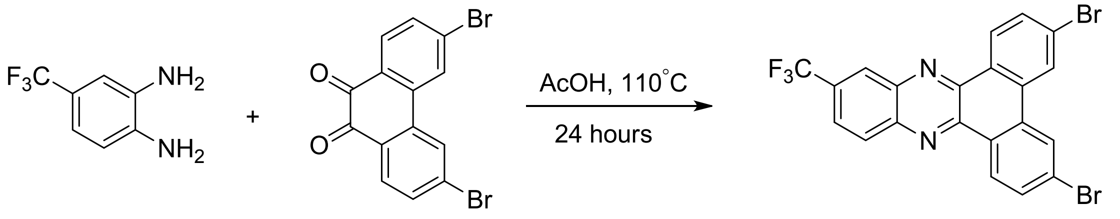

15(c)

3,6-dibromo-11-(trifluoromethyl)dibenzo[*a,c*]phenazine (**15(c)**). 4-(trifluoromethyl)benzene-1,2-diamine (2.00 g, 5.40 mmol, 1.0 eq.), 3,6-dibromophenanthrene-9,10-dione (1.05 g, 5.96 mmol, 1.1 eq.), and 70 mL glacial acetic acid. Combination of *n*-hexane/THF (4:1 v/v) as the eluent during column chromatography. Isolated product appeared as yellow solid. Yield: 2.50 g (90%). The NMR data cannot be recorded as the compound hardly dissolves in deuterated solvents.

3.4. Microwave-assisted aromatic nucleophilic substitution (S_NAr) of 15(a) into 16

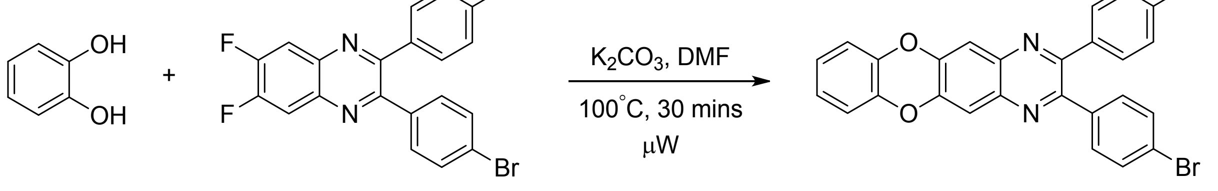

15(a) **16**

2,3-bis(4-bromophenyl)benzo[5,6][1,4]dioxino[2,3-*g*]quinoxaline (**16**). **15(a)** (1.00 g, 2.1 mmol, 1.0 eq.), pyrocatechol (0.46 g, 4.20 mmol, 2.0 eq.), potassium carbonate (0.87 g, 6.30 mmol, 3.0 eq.) and dry DMF (15 mL) were added in a G30 reaction vial under argon atmosphere. The vial was irradiated with microwave energy at 100°C for 30 minutes. Then, mixture was poured into water and the precipitate formed was collected the *via* suction filtration. The solid collected was washed with water. The target compound was obtained as yellow powder and is pure enough to proceed with the next step without any additional purification steps. Yield: 1.05 g (91%). **m.p.** 306°C. **^{1}H NMR** (400 MHz, $CDCl_3$): δ 7.53 – 7.44 (m, 6H), 7.36 (d, *J* = 8.2 Hz, 4H), 7.00 (s, 4H). **^{13}C NMR** (100 MHz, $CDCl_3$): δ 151.03, 145.49, 140.90, 139.77, 137.75, 131.80, 131.49, 124.62, 123.68, 116.83, 112.85. **MS** (ESI) *m/z* calcd for $C_{26}H_{14}Br_2N_2O_2$: 546.22 $[M]^+$; found 546.76.

3.5. Microwave-assisted, Two-fold Buchwald-Hartwig Coupling (Contd.)

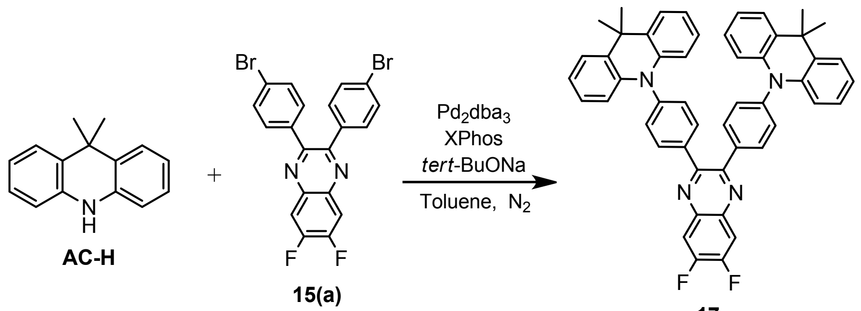

AC-H + **15(a)** → **17**

10,10'-((6,7-difluoroquinoxaline-2,3-diyl)bis(4,1-phenylene))bis(9,9-dimethyl-9,10-dihydroacridine) (**17**). Following **GP2**. **15(a)** (0.60 g, 1.20 mmol, 1.0 eq.), **AC-H** (0.50 g, 2.78 mmol, 2.3 eq.), $Pd_2(dba)_3$ (50 mg, 60 µmol, 5 mol%), XPhos (40 mg, 80 µmol, 7 mol%), sodium *tert*-butoxide (0.12 g, 1.29 mmol, 1.1 eq.) and dry toluene (15 mL). The crude material was purified *via* column chromatography using *n*-hexane/ethyl acetate (4:1 v/v) as the eluent to afford the product as yellow powder. Yield: 0.80 g (86%). **m.p.** 340°C. **^{1}H NMR** (400 MHz, $CDCl_3$): δ 8.03 (t, *J* = 9.2 Hz, 2H), 7.83 (d, *J* = 8.2 Hz, 4H), 7.43 (dd, *J* = 14.0, 7.9 Hz, 8H), 6.87 (dt, *J* = 20.7, 7.3 Hz, 8H), 6.32 (d, *J* = 8.0 Hz, 4H), 1.69 (s, 12H). **^{13}C NMR** (100 MHz, $CDCl_3$): δ 153.31, 142.54, 140.74, 138.31, 132.55, 131.59, 130.43, 126.65, 125.28, 121.01, 114.14, 36.16, 31.03, 29.85. **MS** (ESI) *m/z* calcd for $C_{50}H_{38}F_2N_4$: 732.88 $[M]^+$; found 732.71.

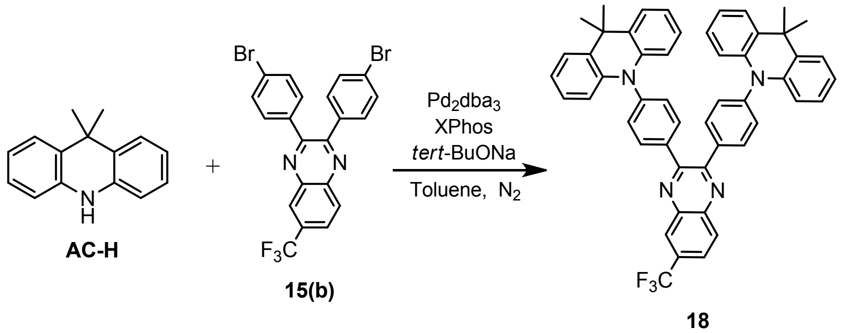

10,10'-((6-(trifluoromethyl)quinoxaline-2,3-diyl)bis(4,1-phenylene))bis(9,9-dimethyl-9,10-dihydroacridine) (**18**). Following **GP2**. **15(b)** (0.30 g, 0.59 mmol, 1.0 eq.), **AC-H** (0.27 g, 1.29 mmol, 2.2 eq.), $Pd_2(dba)_3$ (20 mg, 20 µmol, 3 mol%), XPhos (10 mg, 40 µmol, 7 mol%), sodium *tert*-butoxide (0.12 g, 1.29 mmol, 2.2 eq.) and dry toluene (15 mL). The crude material was purified *via* column chromatography using *n*-hexane/ethyl acetate (4:1 v/v) as the eluent to afford the product as yellow powder. Yield: 0.3 g (66%). **m.p.** 273°C. **^{1}H NMR** (400 MHz, $CDCl_3$): δ 8.61 (s, 1H), 8.41 (d, *J* = 8.8 Hz, 1H), 8.05 (d, *J* = 8.8 Hz, 1H), 7.92 – 7.83 (m, 4H), 7.45 (t, *J* = 8.0 Hz, 8H), 6.87 (dt, *J* = 15.2, 7.2 Hz, 8H), 6.33 (d, *J* = 7.9 Hz, 4H), 1.69 (s, 12H). **^{13}C NMR** (100 MHz, $CDCl_3$): δ 155.03, 154.47, 142.79, 142.73, 142.55, 140.73, 140.54, 138.23, 132.62, 132.57, 131.62, 130.74, 130.47, 126.66, 126.31, 125.30, 121.04, 114.16, 36.17, 31.03. **MS** (ESI) *m/z* calcd for $C_{51}H_{39}F_3N_4$: 764.31 $[M]^+$; found 764.42

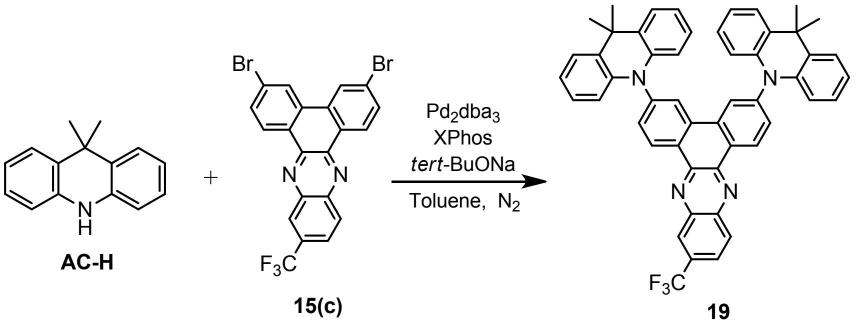

3,6-bis(9,9-dimethylacridin-10(9*H*)-yl)-11-(trifluoromethyl)dibenzo[*a,c*]phenazine (**19**). Following **GP2**. **15(c)** (0.30 g, 0.59 mmol, 1.0 eq.), **AC-H** (0.20 g, 1.30 mmol, 2.3 eq.), $Pd_2(dba)_3$ (20 mg, 20 µmol, 3 mol%), XPhos (10 mg, 40 µmol, 7 mol%), sodium *tert*-butoxide (0.12 g, 1.30 mmol, 2.3 eq.), dry toluene (15 mL). The crude material was purified *via* column chromatography using *n*-hexane/ethyl acetate (4:1 v/v) as the eluent to afford the product as red powder. Yield: 0.35 g (77%). **m.p.** 319°C. **^{1}H NMR** (400 MHz, $CDCl_3$): δ 9.73 (t, *J* = 7.6 Hz, 2H), 8.75 (s, 1H), 8.54 (d, *J* = 8.9 Hz, 1H), 8.44 (s, 2H), 8.10 (d, *J* = 8.9 Hz, 1H), 7.80 (d, *J* = 8.4 Hz, 2H), 7.51 – 7.43 (m, 4H), 6.96 – 6.89 (m, 8H), 6.33 (dd, *J* = 5.5, 3.0 Hz, 4H), 1.73 (s, 12H). **^{13}C NMR** (100 MHz, $CDCl_3$): δ 144.51, 144.39, 143.81, 143.38, 141.34, 140.72, 134.83, 134.62, 132.15, 132.09, 131.04, 130.29, 129.93, 129.83, 129.76, 126.63, 126.38, 125.75, 121.06, 114.17, 36.18, 31.82. **MS** (ESI) *m/z* calcd for $C_{51}H_{37}F_3N_4$: 762.30 $[M]^+$; found 762.69.

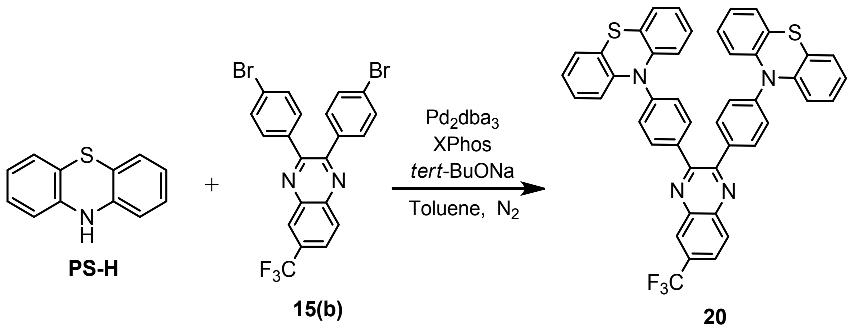

10,10'-((6-(trifluoromethyl)quinoxaline-2,3-diyl)bis(4,1-phenylene))bis(10*H*-phenothiazine) (**20**). Following **GP2**. **15(b)** (0.30 g, 0.59 mmol, 1.0 eq.), **PS-H** (0.25 g, 1.29 mmol, 2.2 eq.), $Pd_2(dba)_3$ (20 mg, 20 µmol, 3 mol%), XPhos (10 mg, 40 µmol, 7 mol%), sodium *tert*-butoxide (0.12 g, 1.29 mmol, 2.2 eq.) and dry toluene (15 mL). The crude material was purified *via* column chromatography using *n*-hexane/ethyl acetate (4:1 v/v) as the eluent to afford the product as yellow powder. Yield: 0.18 g (40%). The compound decomposes at 406°C. **^{1}H NMR** (400 MHz, DMSO-d_6): δ 8.58 (s, 1H), 8.41 (d, J = 8.7 Hz, 1H), 8.18 (d, J = 8.8 Hz, 1H), 7.73 (d, J = 7.9 Hz, 4H), 7.41 – 7.33 (m, 4H), 7.14 (d, J = 5.7 Hz, 4H), 6.88 (d, J = 5.4 Hz, 8H), 6.36 (t, J = 6.5 Hz, 4H). **^{13}C NMR** (100 MHz, DMSO-d_6): δ 155.12, 154.49, 142.84, 142.80, 141.92, 141.80, 139.61, 136.88, 136.80, 132.41, 130.76, 127.62, 127.37, 127.03, 123.40, 123.35, 121.67, 121.50, 117.81, 117.67. **MS** (ESI/Q-TOF) *m/z* calcd for $C_{45}H_{28}F_3N_4S_2$: 745.1707 $[M+H]^+$; found 745.1723.

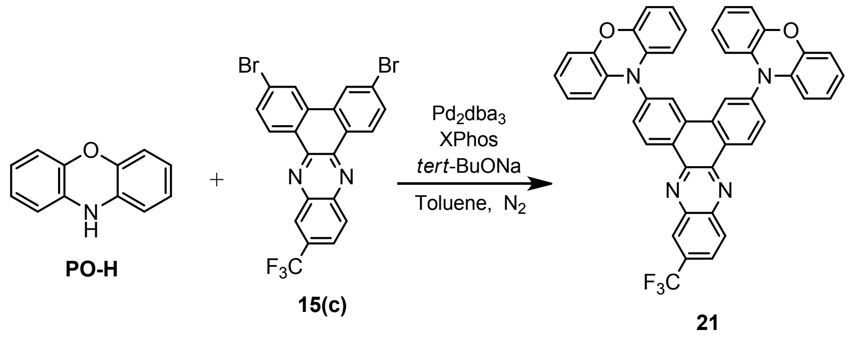

10,10'-(11-(trifluoromethyl)dibenzo[a,c]phenazine-3,6-diyl)bis(10H-phenoxazine) (**21**). Following **GP2. 15(c)** (0.30 g, 0.59 mmol, 1.0 eq.), **PO-H** (0.2g, 1.3mmol, 2.2 eq.), $Pd_2(dba)_3$ (20 mg, 20 µmol, 3 mol%), XPhos (10 mg, 40 µmol, 7 mol%), sodium *tert*-butoxide (0.12 g, 1.30 mmol, 2.2 eq.) and dry toluene (15 mL). The crude material was purified *via* column chromatography using *n*-hexane/ethyl acetate (4:1 v/v) as the eluent to afford the product as red powder. Yield: 0.32 g (75%). **m.p.** 398°C. **^{1}H NMR** (400 MHz, $CDCl_3$): δ 8.53 (s, 3H), 8.33 (d, J = 8.7 Hz, 3H), 7.99 (d, J = 8.8 Hz, 3H), 7.72 (d, J = 8.1 Hz, 12H), 7.34 (d, J = 8.1 Hz, 12H), 7.13 (d, J = 6.7 Hz, 13H), 6.95 – 6.82 (m, 24H), 6.50 (d, J = 5.9 Hz, 12H). **^{13}C NMR** (100 MHz, $CDCl_3$): δ 154.85, 154.29, 143.52, 143.41, 143.37, 142.45, 140.41, 136.56, 136.51, 132.18, 132.14, 130.60, 127.49, 127.42, 127.28, 127.15, 126.05, 123.99, 123.85, 123.69, 123.65, 118.80, 118.70, 34.81, 31.74, 22.80, 14.27. **MS** (ESI/Q-TOF) *m/z* calcd for $C_{45}H_{25}F_3N_4O_2$: 710.1929 $[M]^+$; found 710.1926.

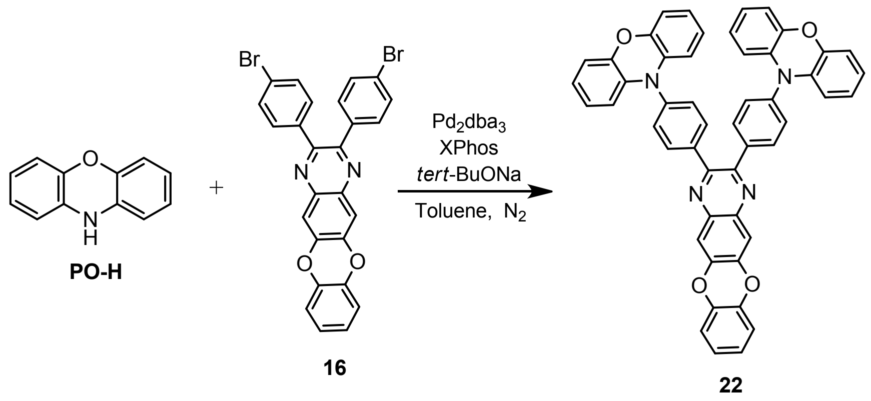

2,3-bis(4-(10*H*-phenoxazin-10-yl)phenyl)benzo[5,6][1,4]dioxino[2,3-*g*]quinoxaline (**22**). Following **GP2**. **16** (0.30 g, 0.54 mmol, 1.0 eq.), **PO-H** (0.52 g, 1.2 mmol, 2.2 eq.), $Pd_2(dba)_3$ (20 mg, 20 µmol, 4 mol%), XPhos (10 mg, 30 µmol, 6 mol%), sodium *tert*-butoxide (0.11 g, 1.20 mmol, 2.2 eq.) and dry toluene (15 mL). The crude material was purified *via* column chromatography using *n*-hexane/ethyl acetate (4:1 v/v) as the eluent to afford the product as yellow powder. Yield: 0.30 g (73%). **m.p.** 335°C. **^{1}H NMR** (400 MHz, $CDCl_3$): δ 7.74 (d, *J* = 8.1 Hz, 4H), 7.62 (s, 2H), 7.37 (d, *J* = 8.1 Hz, 4H), 7.04 (s, 4H), 6.69 (d, *J* = 7.8 Hz, 4H), 6.62 (t, *J* = 7.6 Hz, 4H), 6.51 (t, *J* = 7.6 Hz, 4H), 5.95 (d, *J* = 7.9 Hz, 4H). **^{13}C NMR** (100 MHz, $CDCl_3$): δ 151.70, 145.69, 144.03, 140.90, 139.95, 139.81, 139.12, 134.07, 132.68, 130.98, 124.70, 123.54, 121.70, 116.88, 115.69, 113.27, 112.99. **MS** (ESI/Q-TOF) *m/z* calcd for $C_{50}H_{30}N_4O_4$: 750.2267 $[M]^+$; found 750.2294.

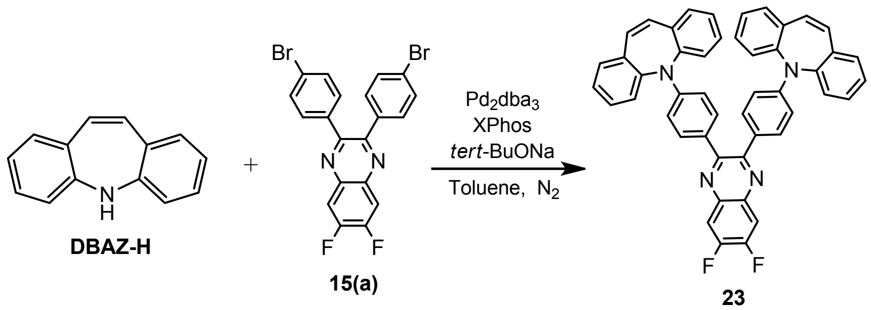

5,5'-((6,7-difluoroquinoxaline-2,3-diyl)bis(4,1-phenylene))bis(5*H*-dibenzo[*b,f*]azepine)) (**23**). Following **GP2**. **15(a)** (0.20 g, 0.42 mmol, 1.0 eq.), **DBAZ-H** (0.17 g, 0.92 mmol, 2.2 eq.), $Pd_2(dba)_3$ (10 mg, 20 µmol, 5 mol%), XPhos (10 mg, 20 µmmol, 5 mol%), sodium *tert*-butoxide (80 mg, 0.92 mmol, 2.2 eq.) and dry toluene (15 mL). The crude material was purified *via* column chromatography using *n*-hexane/ethyl acetate (4:1 v/v) as the eluent to afford the product as yellow powder. Yield: 0.17 g (57%). **m.p.** 323°C. **^{1}H NMR** (400 MHz, $CDCl_3$): δ 7.71 (t, *J* = 9.4 Hz, 2H), 7.51 (d, *J* = 3.9 Hz, 8H), 7.45 (d, *J* = 7.6 Hz, 4H), 7.38 (dt, *J* = 8.0, 4.1 Hz, 4H), 7.18 (d, *J* = 8.5 Hz, 4H), 6.79 (s, 4H), 6.21 (d, *J* = 8.5 Hz, 4H). **^{13}C NMR** (100 MHz, $CDCl_3$): δ 153.60, 149.73, 142.87, 138.04, 136.37, 130.66, 130.53, 130.27, 130.25, 129.85, 128.64, 127.38, 114.41, 111.95. **MS** (ESI) m/z calcd for $C_{48}H_{30}F_2N_4$: 700.24 $[M]^+$; found 700.82.

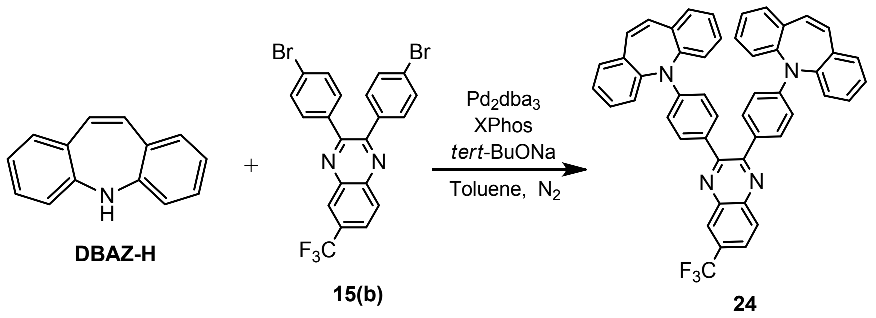

5,5'-((6-(trifluoromethyl)quinoxaline-2,3-diyl)bis(4,1-phenylene))bis(5*H*-dibenzo[*b,f*]azepine) (**24**). Following **GP2**. **15(b)** (0.30 g, 0.59 mmol, 1.0 eq.), **DBAZ-H** (0.25 g, 1.2 mmol, 2.0 eq.), $Pd_2(dba)_3$ (20 mg, 20 µmol, 3 mol%), XPhos (10 mg, 40 µmol, 7 mol%), sodium *tert*-butoxide (0.12 g, 1.29 mmol, 2.2 eq.) and dry toluene (15 mL). The crude material was purified *via* column chromatography using *n*-hexane/ethyl acetate (4:1 v/v) as the eluent to afford the product as yellow powder. Yield: 0.20 g (46%). The compound decomposes at 403°C. **^{1}H NMR** (400 MHz, $CDCl_3$): δ 8.28 (s, 1H), 8.06 (d, *J* = 8.8 Hz, 1H), 7.75 (d, *J* = 8.7 Hz, 1H), 7.52 (d, *J* = 3.9 Hz, 8H), 7.46 (d, *J* = 7.6 Hz, 4H), 7.39 (dt, *J* = 7.8, 4.0 Hz, 4H), 7.26 – 7.20 (m, 5H), 6.79 (s, 4H), 6.22 (d, *J* = 8.3 Hz, 4H). **^{13}C NMR** (100 MHz, $CDCl_3$): δ 155.25, 154.71, 149.99, 149.91, 142.84, 142.80, 142.00, 139.83, 136.36, 130.66, 130.54, 130.42, 130.33, 130.24, 129.92, 129.87, 128.59, 127.42, 126.87, 124.49, 111.98, 32.08, 29.85, 29.81, 29.51, 22.85, 14.28. **MS** (ESI) *m/z* calcd for $C_{49}H_{31}F_3N_4$: 732.25 $[M]^+$; found 732.57.

3.6. Microwave-assissted, Two-fold, Buchwald-Hartwig Amination of Aryl Chloride

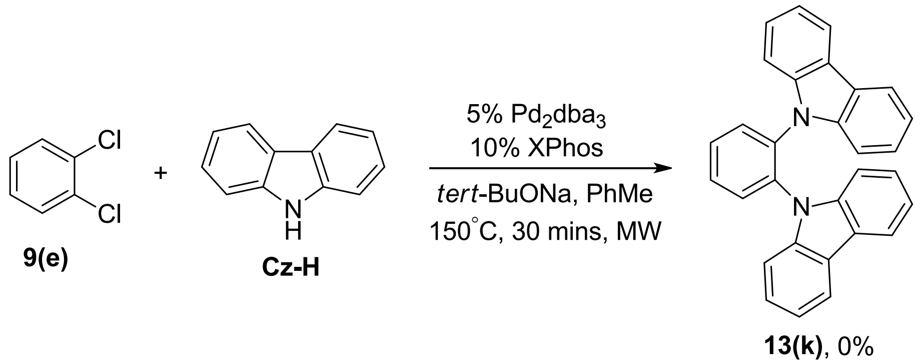

Scheme S1. Buchwald-Hartwig cross-coupling between **9(e)** and **Cz-H** under microwave irradiation condition.

The feasibility of the presented system against cross-coupling with aryl dichloride was also studied as suggested by Reviewer 2. The aryl dichloride used was 1,2-dichlorobenzene (**9(e)**) because the *para* isomer was not available during the investigation. Using the same cross-coupling condition as shown in the **Scheme 4** of the main text, neither the two-fold (**13k**) nor the one-fold coupling product was observed. This was confirmed through TLC profiling and mass spectrometric analysis of the crude reaction mass. This is observation is ascribed to the C-Cl bonding being less labile than C-Br bonding.

3.7. Friedel-Crafts alkylation of carbazole into 25(a) and 25(b)

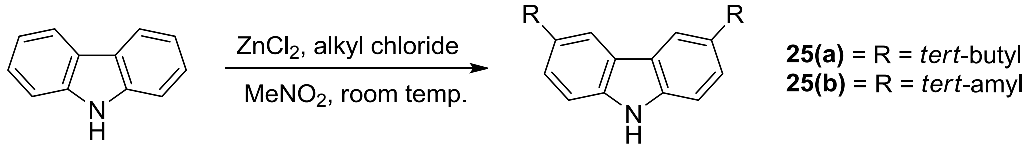

General procedure for Friedel-Crafts Alkylation: Carbazole (**Cz-H**) (3.34 g, 20.0 mmol, 1.0 eq.) and anhydrous $ZnCl_2$ (8.20 g, 60.0 mmol, 3.0 eq.) were suspended in 55 mL of $MeNO_2$ at room temperature. To the suspension was added *tert*-butyl chloride (4.8 mL, 44.0 mmol, 2.2 eq.) OR *tert*-amyl chloride (5.4 mL, 44.0 mmol, 2.2 eq.) in one portion. A drying tube packed with anhydrous $CaCl_2$ was installed at the opening of the reaction flask to maintain an anhydrous reaction environment. The reaction mixture was continued to stir at room temperature for 4 hours. Upon the addition of alkyl chloride, the reaction turns into a deep green, homogenous solution almost instantaneously before precipitation of fine solid particles. Then, the reaction mass was quenched by pouring into water and extracted against chloroform. The chloroform extracts were combined, washed with brine, dried over anhydrous Na_2SO_4, filtered, and concentrated under vacuum condition to afford brownish grey solid. The crude material was purified through column chromatography using combination of *n*-hexane/ethyl acetate (EA) as the eluent to afford the desired product.

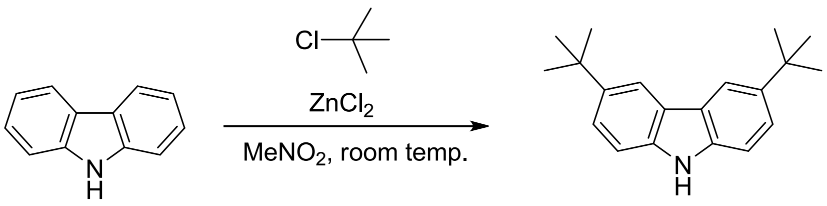

25(a)

3,6-di-*tert*-butyl-9*H*-carbazole (**25(a)**).[11] Combination of *n*-hexane/EA (97:3 v/v) as the eluent during column chromatography followed by recrystallization from *n*-hexane/DCM. Isolated product appeared as needle-like, white solid. Yield: 3.55 g (64%). **^{1}H NMR** (400 MHz, $CDCl_3$): δ 8.08 (s, 2H), 7.83 (br s, NH), 7.46 (dd, *J* = 8.8, 2 Hz, 2H), 7.32 (d, *J* = 8.8 Hz, 2H), 1.46 (s, 18H). **^{13}C NMR** (100 MHz, $CDCl_3$): δ 142.3, 138.1, 123.6, 123.4, 116.3, 110.1, 34.8, 32.1. **HRMS** (ESI/Q-TOF) *m/z* calcd for $C_{20}H_{26}N$: 280.2059 $[M+H]^+$; found 280.2057. The spectral data is consistent with reported data in literature.[11]

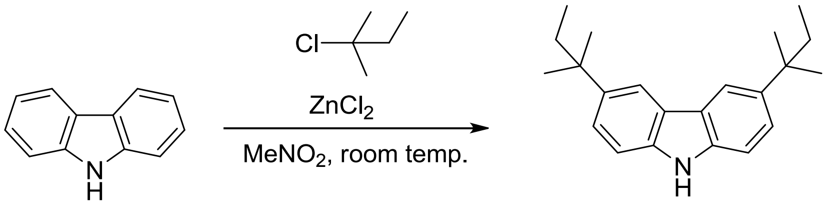

25(b)

3,6-di-*tert*-pentyl-9H-carbazole (**25(b)**).[11] Combination of *n*-hexane/EA (95:5 v/v) as the eluent during column chromatography followed by sonication in *n*-hexane to remove the oily

impurities. Isolated product appeared as granular white solid. Yield: 3.78 g (61%). **^{1}H** NMR (400 MHz, $CDCl_3$): δ 8.01 (s, 2H), 7.81 (br s, NH), 7.39 (d, *J* = 8.5 Hz, 2H), 7.32 (d, *J* = 8.5 Hz, 2H), 1.75 (q, *J* = 7.4 Hz, 4H), 1.45 (s, 12H), 0.71 (t, *J* = 7.4 Hz, 6H). **^{13}C NMR** (100 MHz, $CDCl_3$): δ 140.4, 138.0, 124.0, 123.3, 117.1, 110.0, 37.9, 37.4, 29.1, 9.3. **HRMS** (ESI/Q-TOF) *m/z* calcd for $C_{22}H_{30}N$: 308.2372 $[M+H]^+$; found 308.2374.

3.8. Two-step synthesis of carbazole 25(c) and carbazole 25(d).

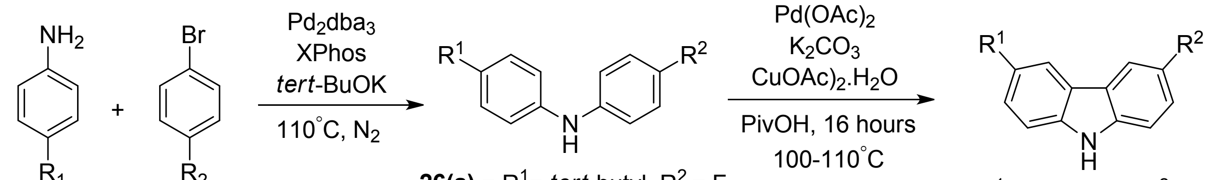

26(a) = R^1= *tert*-butyl, R^2 = F
26(b) = R^1 = R^2 = OMe

25(c) = R^1 = *tert*-butyl, R^2 = F
25(d) = R^1 = R^2 = OMe

The synthesis of **25(c)** and **25(d)** consisted of two steps. The first step involves Buchwald-Hartwig amination of aniline with aryl bromide into diphenylamine motif. Then, followed by oxidative cyclization of the diphenylamine derivatives into their respective carbazole derivatives.

General procedure for Buchwald-Hartwig amination of aniline derivatives: Pd_2dba_3 (1 mol%), XPhos (4 mol%), appropriate aryl bromide (1.0 eq.), appropriate aniline derivative (1.2 eq.), and potassium *tert*-butoxide (*tert*-BuOK, 1.5 eq.) were weighed into a reaction flask followed by the addition of toluene. The reaction mixture was flushed with N_2 gas (balloon) first before heating it up to 110°C for appropriate amount of time. Once the reaction has completed, the reaction mass was cooled down first to room temperature before diluting with either $CHCl_3$ or EA. Then, the resulting solution was filtered through Celite. The filtrate was collected and concentrated under reduced pressure. The crude material was purified *via* column chromatography using *n*-hexane/EA combination as the eluent to afford the desired product.

General procedure for oxidative cyclization: The diphenylamine derivative (1.0 eq.) was weighed into a headspace vial followed by the addition of $Pd(OAc)_2$ (10 mol%), K_2CO_3 (10 mol%), $Cu(OAc)_2.H_2O$ (2.5 eq.), and pivalic acid (PivOH). The vial was sealed with a rubber septum and the mixture was heated to 100-110°C. The reaction mass was heated for 16 hours before cooling down to room temperature. The resulting dark reaction mass was diluted with either DCM or EA and filtered through a silica plug. The filtrate was collected and washed with EDTA solution (0.1 M), saturated $NaHCO_3$ solution, and brine. The filtrate was then dried over anhydrous Na_2SO_4, filtered and concentrated under diminished pressure. The crude material was purified through column chromatography using combination of *n*-hexane/EA as the eluent to afford the desired product.

for the cyclization of **26(a) into **26(c)**, please read the details below as procedure deviates slightly from the general procedure.*

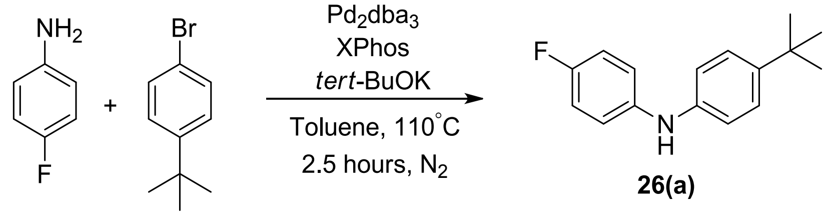

26(a)

4-(*tert*-butyl)-*N*-(4-fluorophenyl)aniline (**26(a)**). 1-bromo-4-*tert*-butylbenzene (1.8 mL, 10.0 mmol, 1.0 eq.), 4-fluoroaniline (1.2 mL, 12.0 mmol, 1.2 eq.), Pd_2dba_3 (0.09 g, 0.1 mmol, 1 mol%), XPhos (0.19 g, 0.4 mmol, 4 mol%), potassium *tert*-butoxide (1.68 g, 15.0 mmol, 1.5 eq.), and toluene (40 mL). 2.5 hours reaction time. Combination of *n*-hexane/EA (95:5 v/v) as the eluent during column chromatography. Isolated product appeared as brownish yellow oil that solidifies upon standing in the fridge. Yield: 2.29 g (91%). **^{1}H NMR** (400 MHz, $CDCl_3$): δ 7.27 (d, *J* = 8.4 Hz, 2H), 7.00-6.93 (m, 6H). **^{13}C NMR** (100 MHz, $CDCl_3$): δ 157.9 (d, *J* = 238.4 Hz), 144.1, 141.1, 139.5, 126.3, 119.9 (d, *J* = 6.6 Hz), 117.3, 115.9 (d, *J* = 21.9 Hz), 34.2, 31.5. **^{19}F NMR** (376 MHz, $CDCl_3$): δ -122.8. **HRMS** (ESI/Q-TOF) *m/z* calcd for $C_{16}H_{19}FN$: 244.1496 $[M+H]^+$; found 144.1491. The spectral data is consistent with reported data in literature.[12]

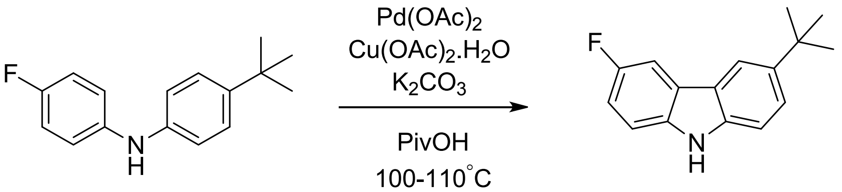

26(a) **25(c)**

3-(*tert*-butyl)-6-fluoro-9*H*-carbazole (**25(c)**).[13] **26(a)** (1.22 g, 5.0 mmol, 1.0 eq.), palladium(II) acetate (0.11 g, 0.5 mmol, 10mol%), potassium carbonate (0.07 g, 0.5 mmol, 10 mol%), copper(II) acetate monohydrate (2.50 g, 12.5 mmol, 2.5 eq.), and pivalic acid (5 mL). After 16 hours of reaction time, the reaction remains incomplete, thus, an additional $Pd(OAc)_2$ (0.11 g, 0.5 mmol, 10 mol%) was added. The reaction mass was continued to heat for another 16 hours. Combination of n-hexane/EA (9:1 v/v) as the eluent during column chromatography. Isolated product appeared as beige solid. Yield: 0.89 g (73%). **^{1}H NMR** (400 MHz, $CDCl_3$): δ 8.00 (d, *J* = 2.0 Hz, 1H), 7.91 (br s, NH), 7.73 (dd, *J* = 8.8, 2.8 Hz, 1H), 7.51 (dd, *J* = 8.4, 1.6 Hz, 1H), 7.35 (d, *J* = 8.8 Hz, 1H), 7.29 (dd, *J* = 9.0, 4.4 Hz, 1H), 7.12 (td, *J* = 9.0, 2.8 Hz, 1H), 1.43 (s, 9H). **^{13}C NMR** (100 MHz, $CDCl_3$): δ 157.5 (d, *J* = 233.6 Hz), 142.6, 138.7, 136.3, 124.6, 124.2 (d, *J* = 9.5 Hz), 122.9 (d, *J* = 4.8 Hz), 116.6, 113.4 (d, *J* = 25.7 Hz), 111.1 (d, *J* = 9.5 Hz), 110.5, 105.9 (d, *J* = 23.8 Hz), 34.8, 32.0. **^{19}F NMR** (376 MHz, $CDCl_3$): δ -124.7. **HRMS** (ESI/Q-TOF) *m/z* calcd for $C_{16}H_{16}FN$: 241.1266 $[M]^+$; found 241.1271. The spectral data is consistent with reported data in literature.[12]

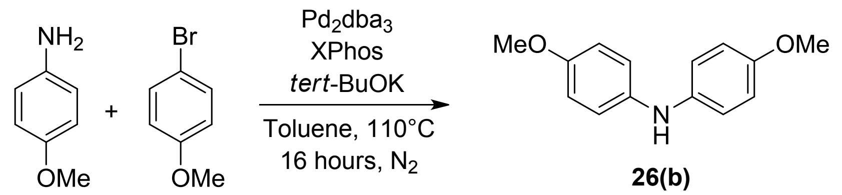

26(b)

Bis(4-methoxyphenyl)amine (**26(b)**). 4-bromoanisole (3.8 mL, 30.0 mmol, 1.0 eq.), *p*-anisidine (4.43 g, 36.0 mmol, 1.2 eq.), Pd_2dba_3 (0.28 g, 0.3 mmol, 1 mol%), XPhos (0.34 g, 0.72 mmol, 2.4 mol%), potassium *tert*-butoxide (5.05 g, 45.0 mmol, 1.5 eq.), and toluene (90 mL). 16 hours reaction time. Combination of *n*-heptane/EA (9:1 v/v) as the eluent during column chromatography. Isolated product appeared as yellow solid. Yield: 4.78 g (95%). **^{1}H NMR** (400 MHz, DMSO-d_6): δ 6.87 (d, J = 8.8 Hz, 4H), 6.76 (d, J = 8.8 Hz, 4H), 3.64 (s, 6H), 3.33 (br s, NH). **^{13}C NMR** (100 MHz, DMSO-d_6): δ 153.3, 138.5, 118.6, 115.1, 55.7. **HRMS** (ESI/Q-TOF) *m/z* calcd for $C_{14}H_{16}NO_2$: 230.1175 $[M+H]^+$; found 230.1172.

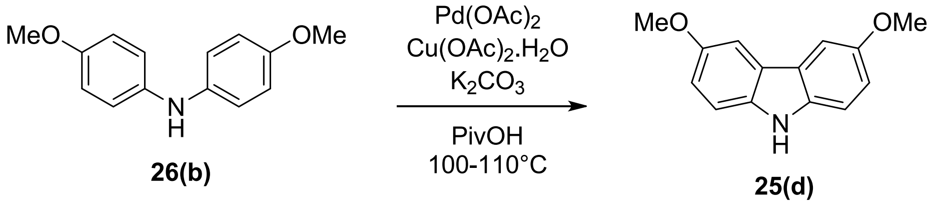

26(b) **25(d)**

3,6-dimethoxy-9*H*-carbazole (**25(d)**).[13] **26(b)** (1.15 g, 5.0 mmol, 1.0 eq.), palladium(II) acetate (0.11 g, 0.5 mmol, 10 mol%), potassium carbonate (0.07 g, 0.5 mmol, 10 mol%), copper(II) acetate monohydrate (2.50 g, 12.5 mmol, 2.5 eq.), and pivalic acid (7 mL).Combination of *n*-hexane/ethyl acetate (85:15 v/v) as the eluent during column chromatography. Isolated product appeared as off-white solid. Yield: 0.93 g (82%). **^{1}H NMR** (400 MHz, DMSO-d_6): δ 10.75 (br s, NH), 7.62 (d, J = 2.8 Hz, 2H), 7.29 (d, J = 8.8 Hz, 2H), 6.94 (dd, J = 8.8, 2.8 Hz, 2H), 3.79 (s, 6H). **^{13}C NMR** (100 MHz, DMSO-d_6): δ 153.1, 135.8, 123.3, 115.4, 112.2, 103.2, 56.1. **HRMS** (ESI/Q-TOF) *m/z* calcd for $C_{14}H_{14}NO_2$: 228.1019 $[M+H]^+$; found 228.1017. The spectral data is consistent with reported data in literature.[14]

4. Copies of ^{1}H, ^{13}C, and ^{19}F Spectrums

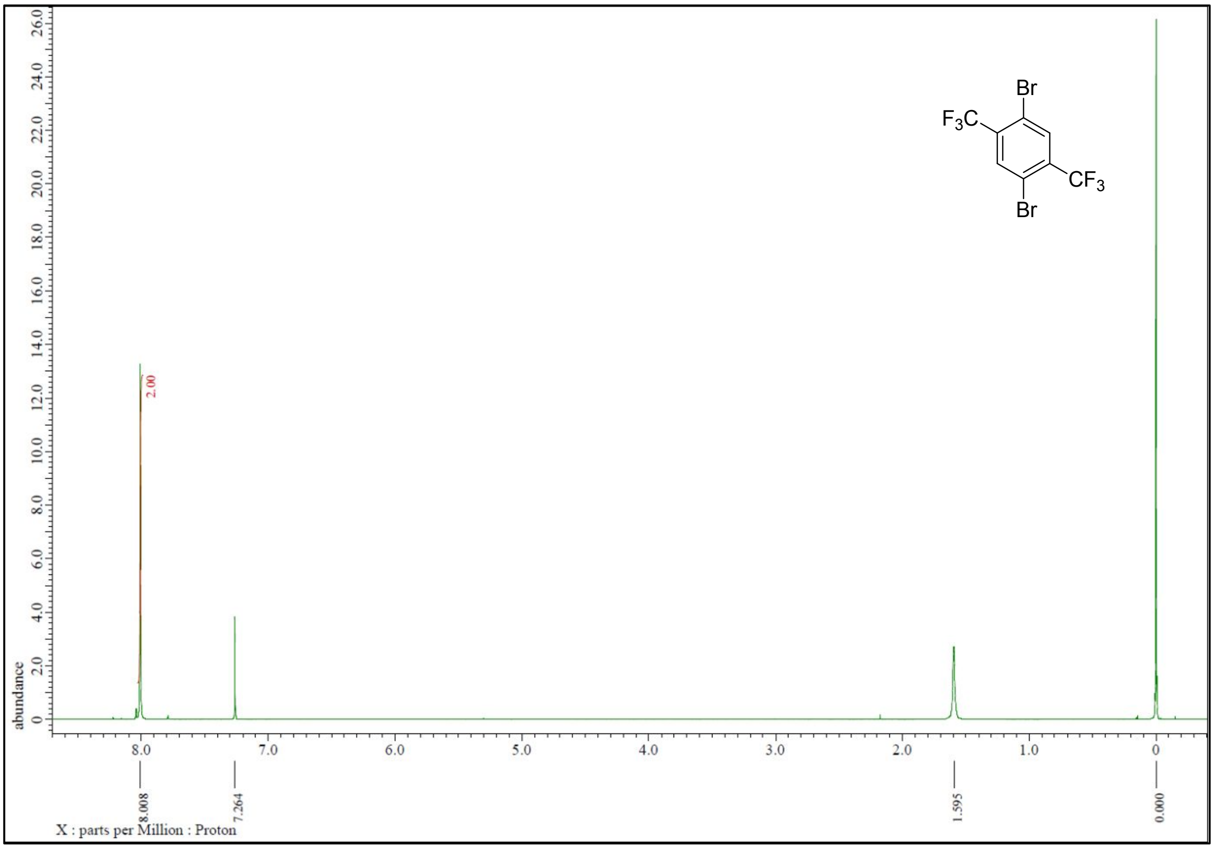

Figure S5: ^{1}H NMR spectrum **9(c)** (400 MHz, $CDCl_3$).

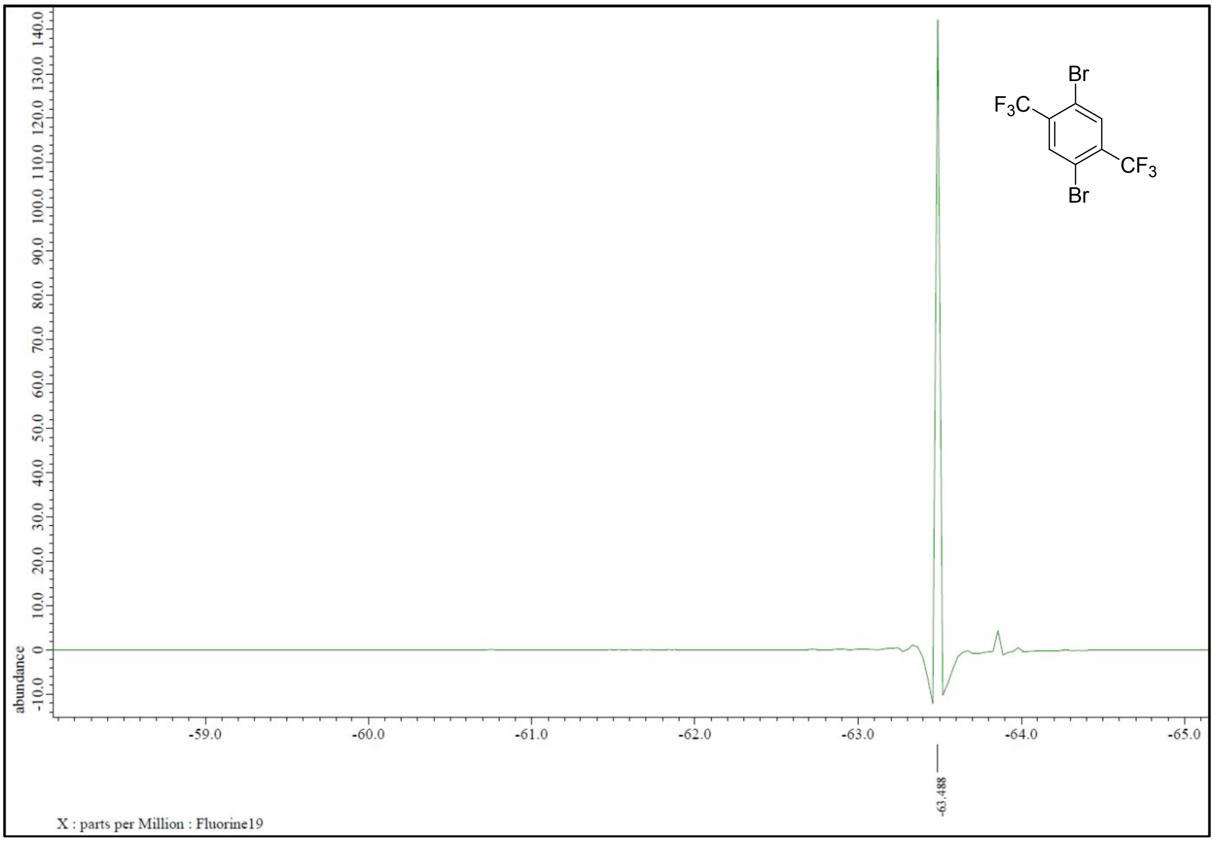

Figure S6: ^{19}F NMR spectrum **9(c)** (376 MHz, $CDCl_3$).

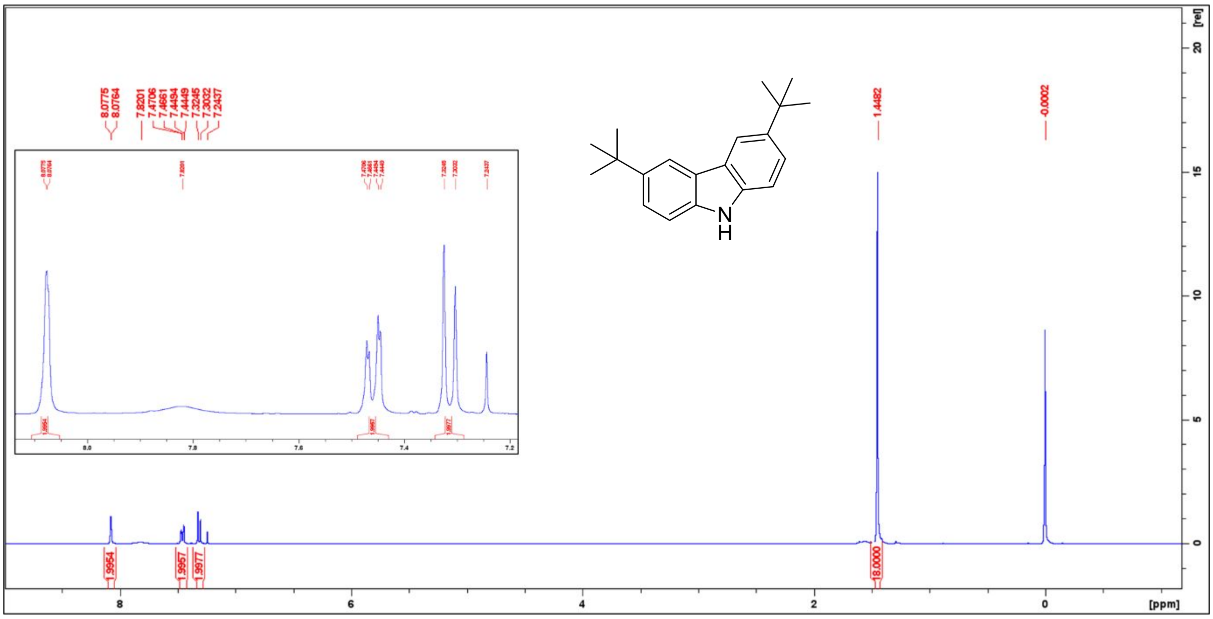

Figure S7: ^{1}H NMR spectrum **25(a)** (400 MHz, $CDCl_3$).

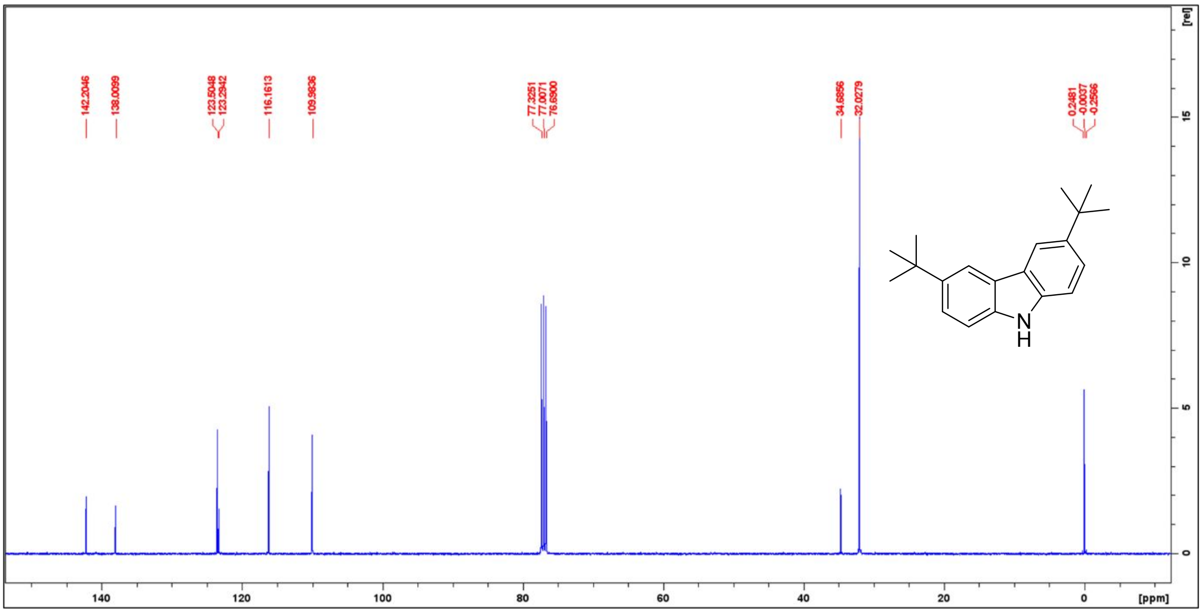

Figure S8: ^{13}C NMR spectrum **25(a)** (100 MHz, $CDCl_3$).

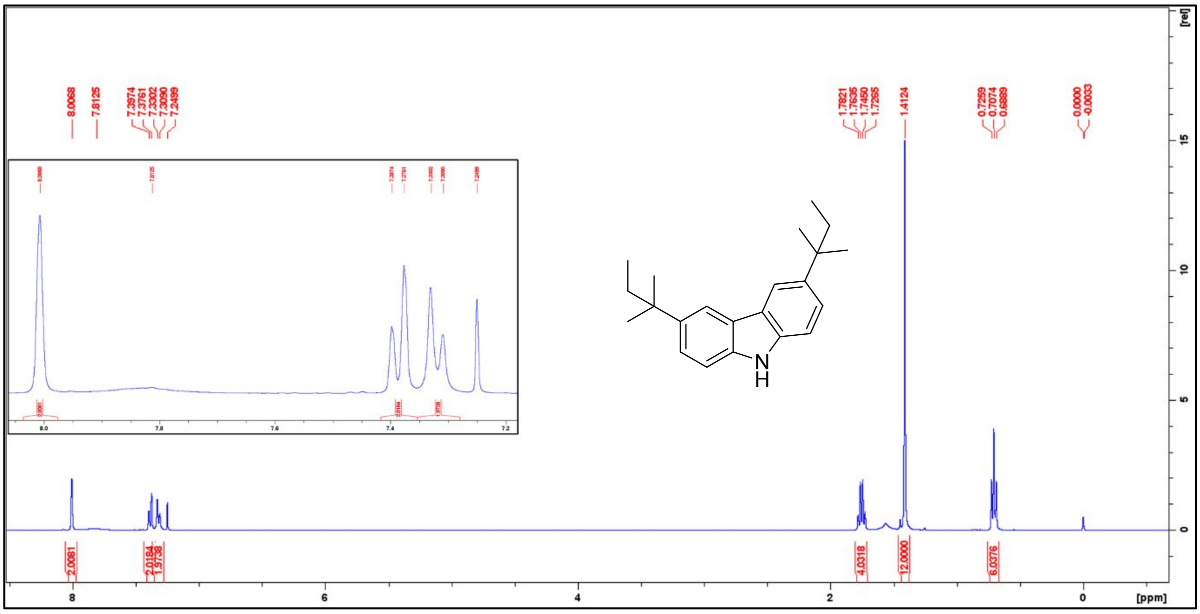

Figure S9: ^{1}H NMR spectrum **25(b)** (400 MHz, $CDCl_3$).

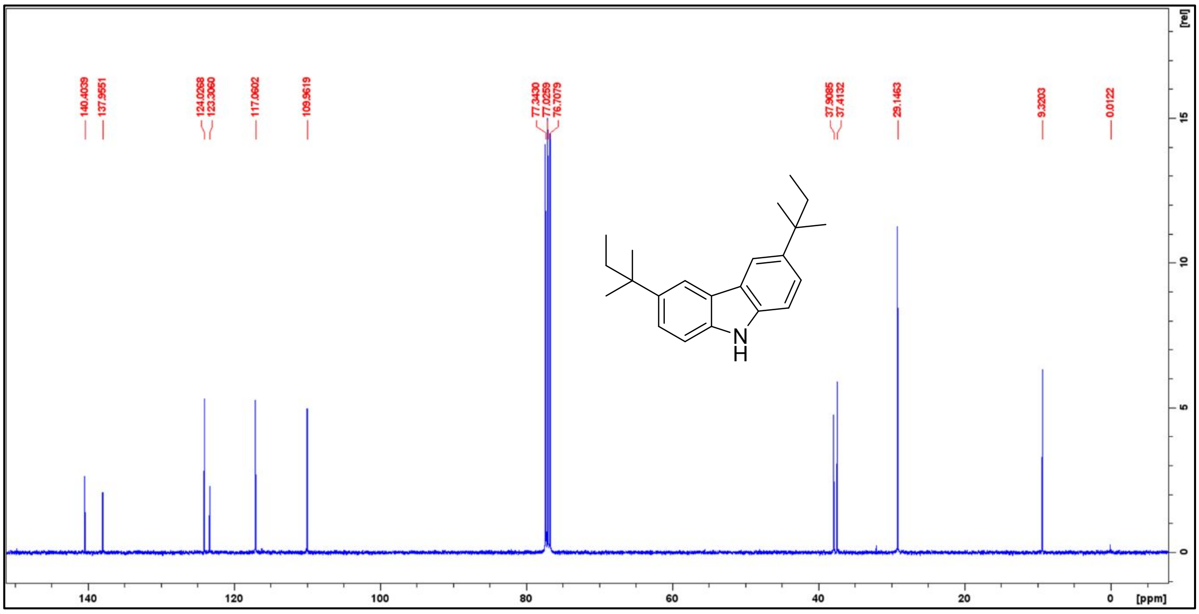

Figure S10: ^{13}C NMR spectrum **25(b)** (100 MHz, $CDCl_3$).

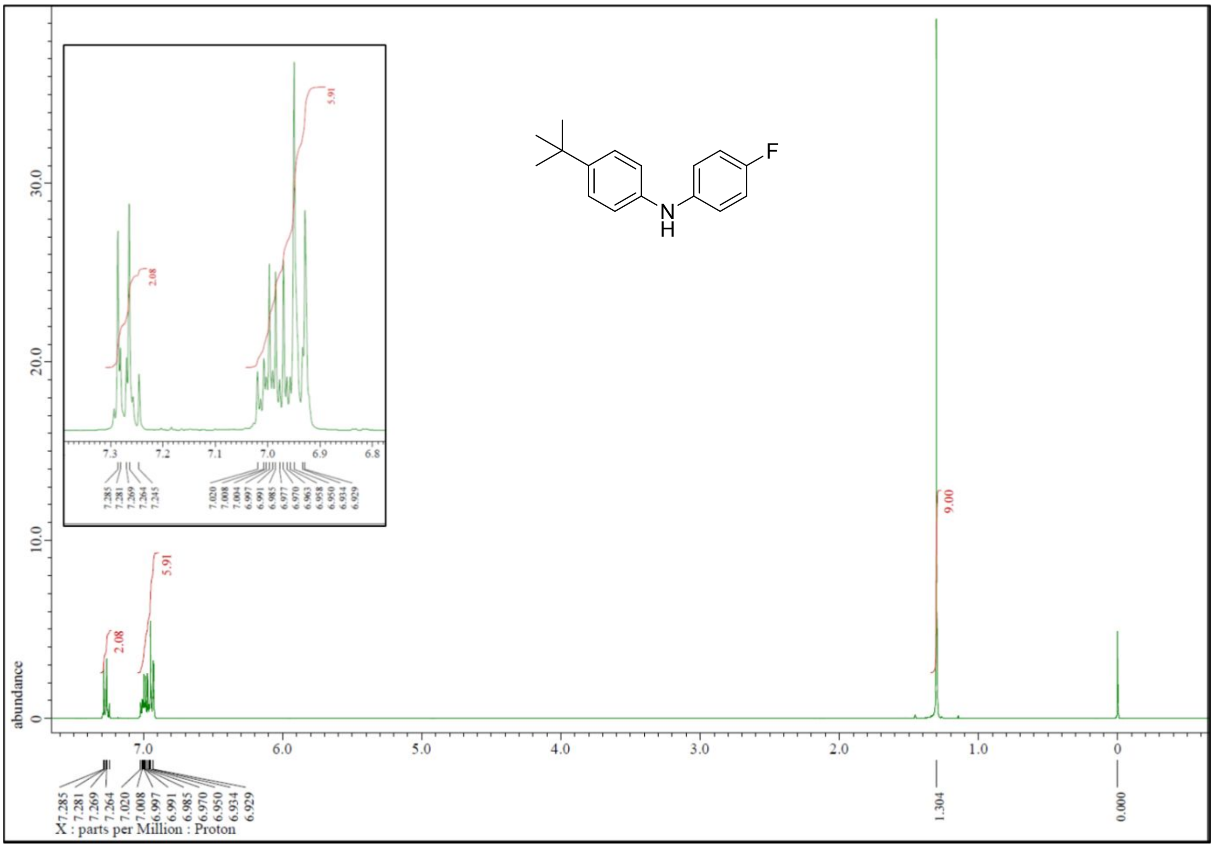

Figure S11: ^{1}H NMR **26(a)** (400 MHz, $CDCl_3$).

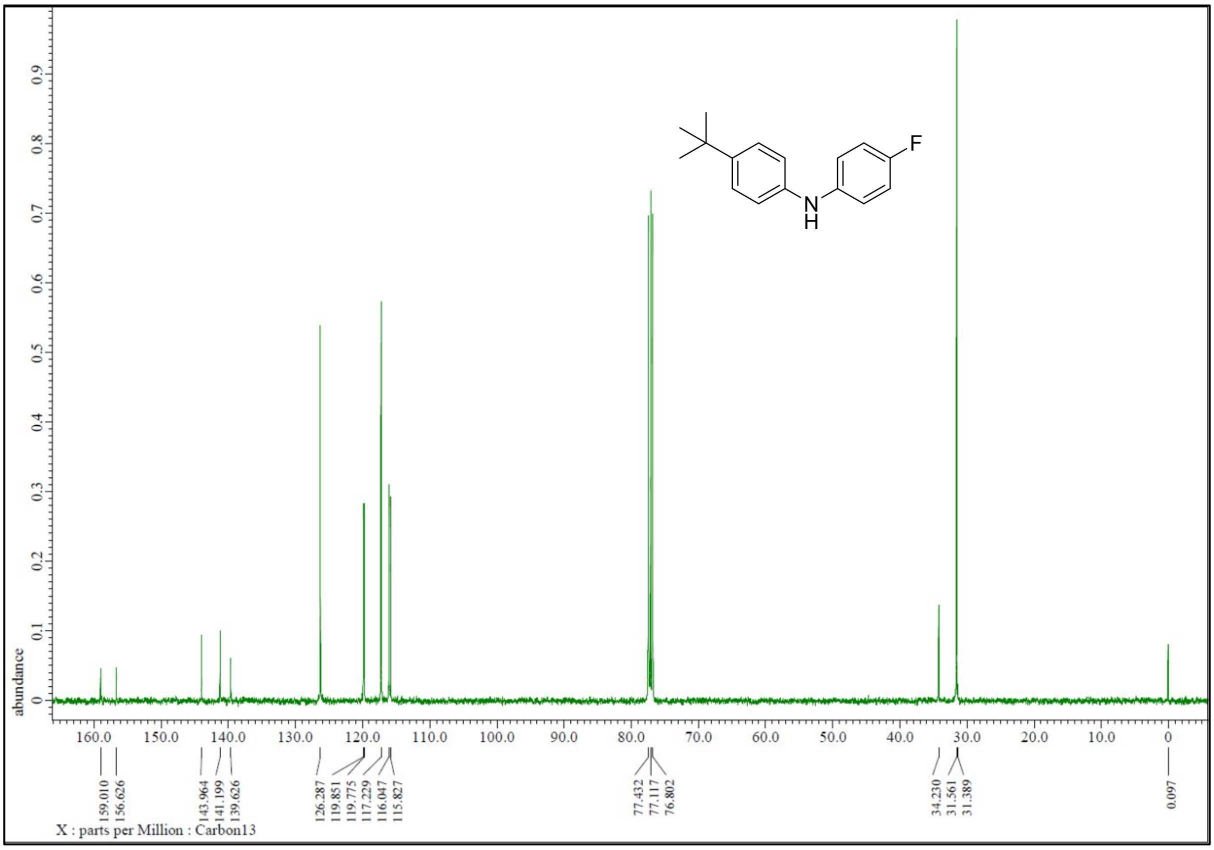

Figure S12: ^{13}C NMR spectrum **26(a)** (100 MHz, $CDCl_3$).

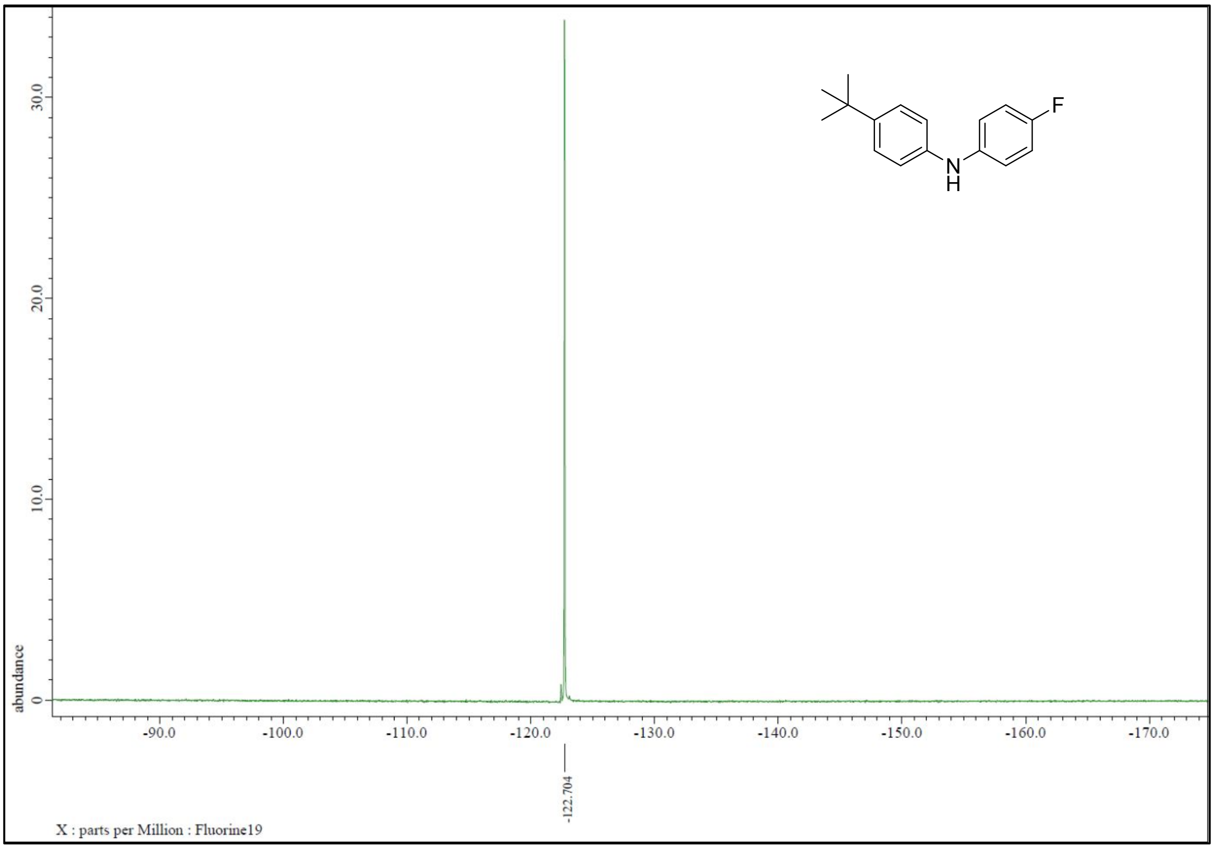

Figure S13: ^{19}F NMR spectrum **26(a)** (376 MHz, $CDCl_3$).

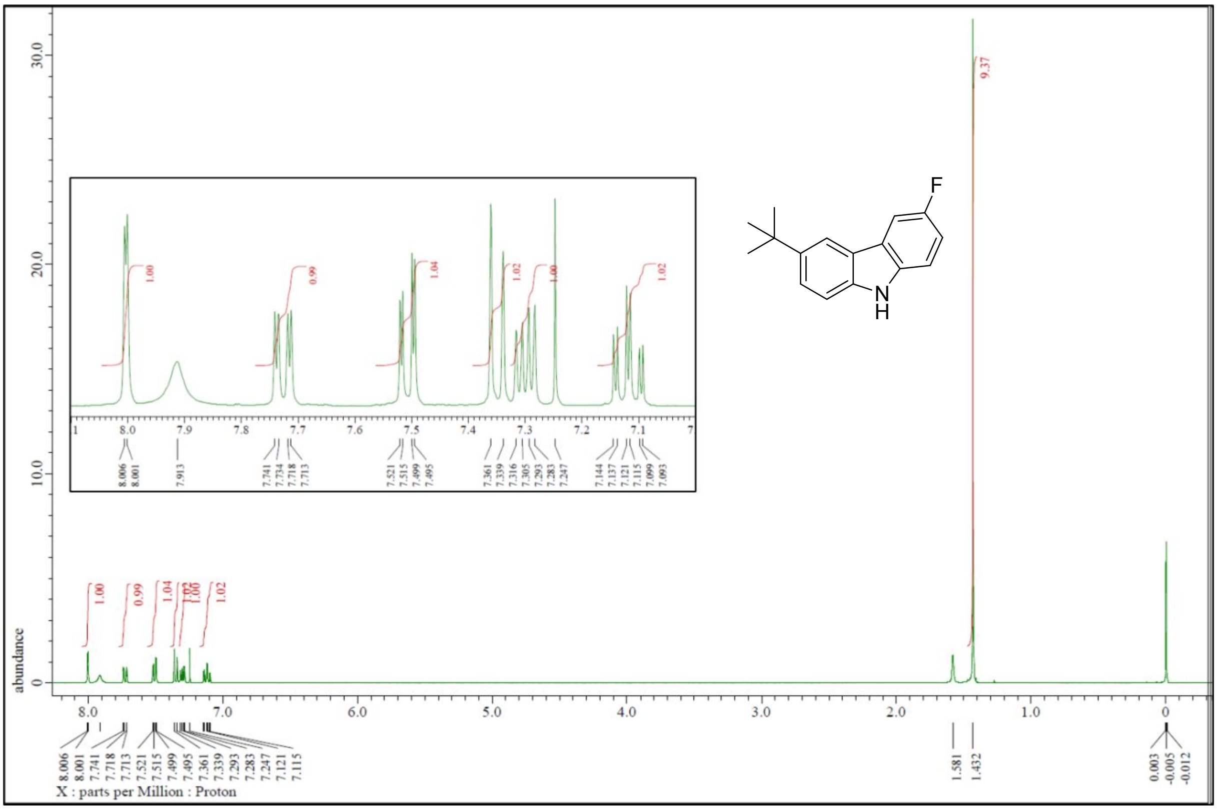

Figure S14: ^{1}H NMR spectrum **25(c)** (400 MHz, $CDCl_3$).

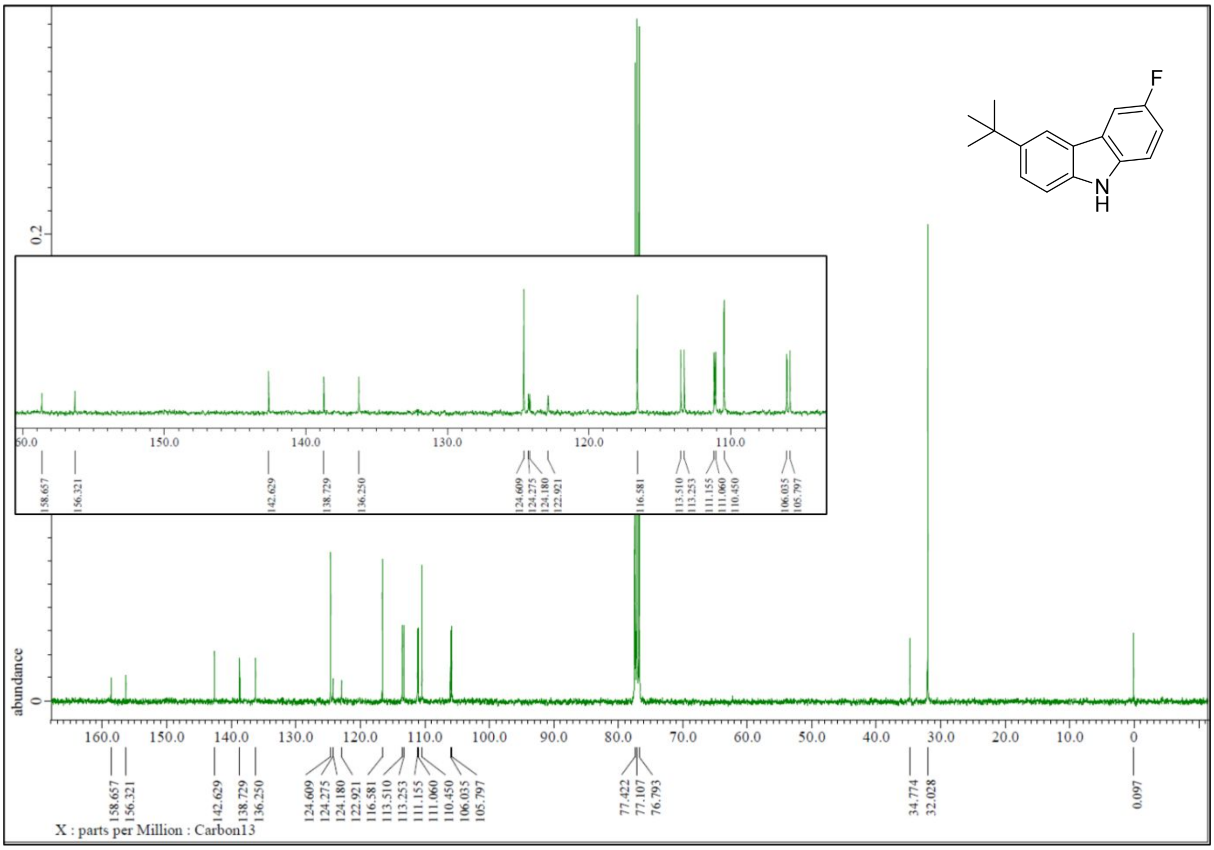

Figure S15: ^{13}C NMR spectrum **25(c)** (100 MHz, $CDCl_3$).

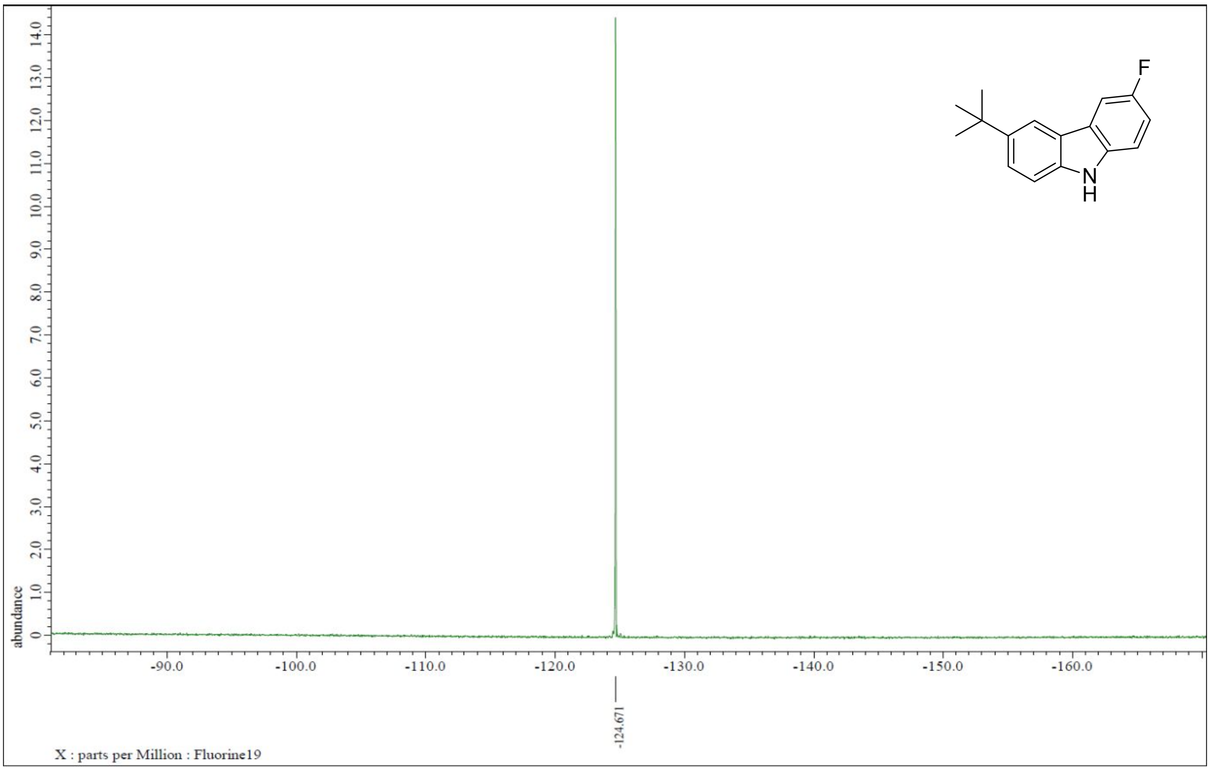

Figure S16: ^{19}F NMR spectrum **25(c)** (376 MHz, $CDCl_3$).

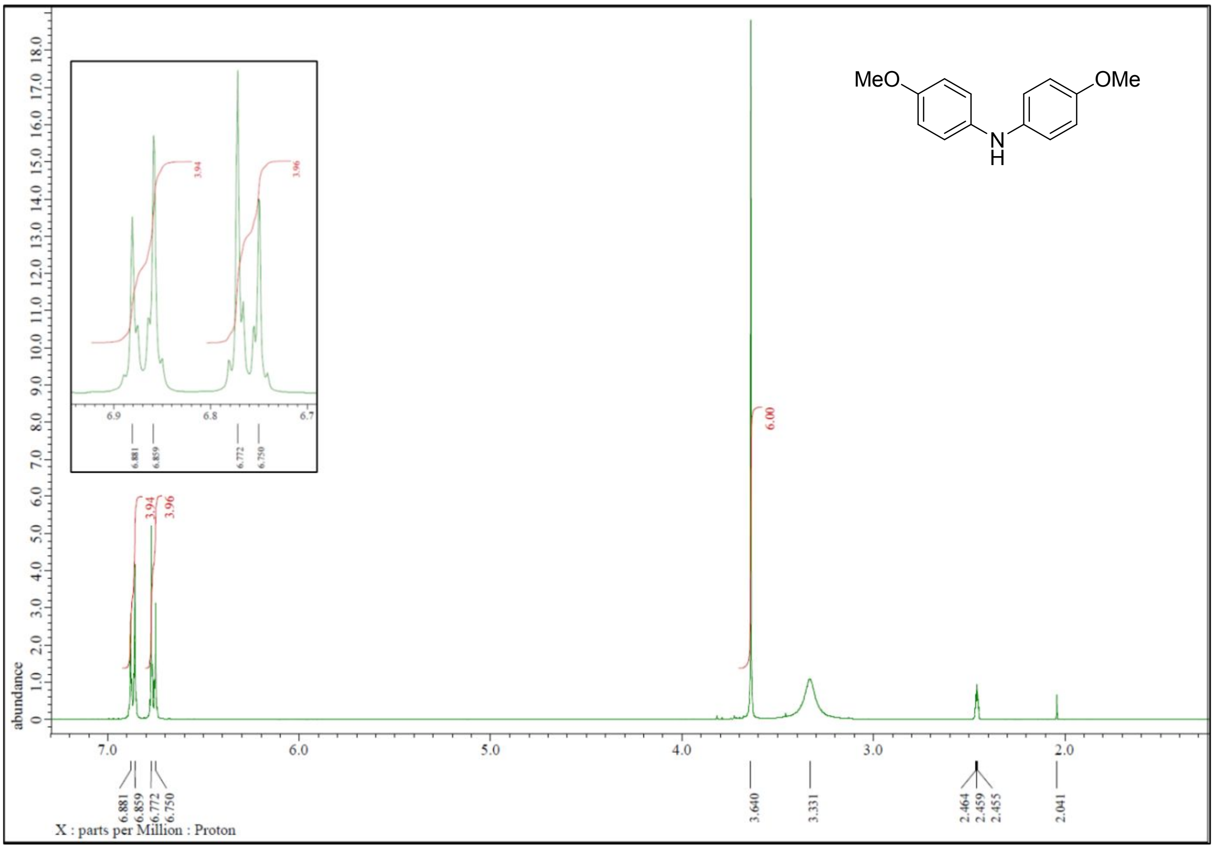

Figure S17: ^{1}H NMR spectrum **26(b)** (400 MHz, [D_6]DMSO).

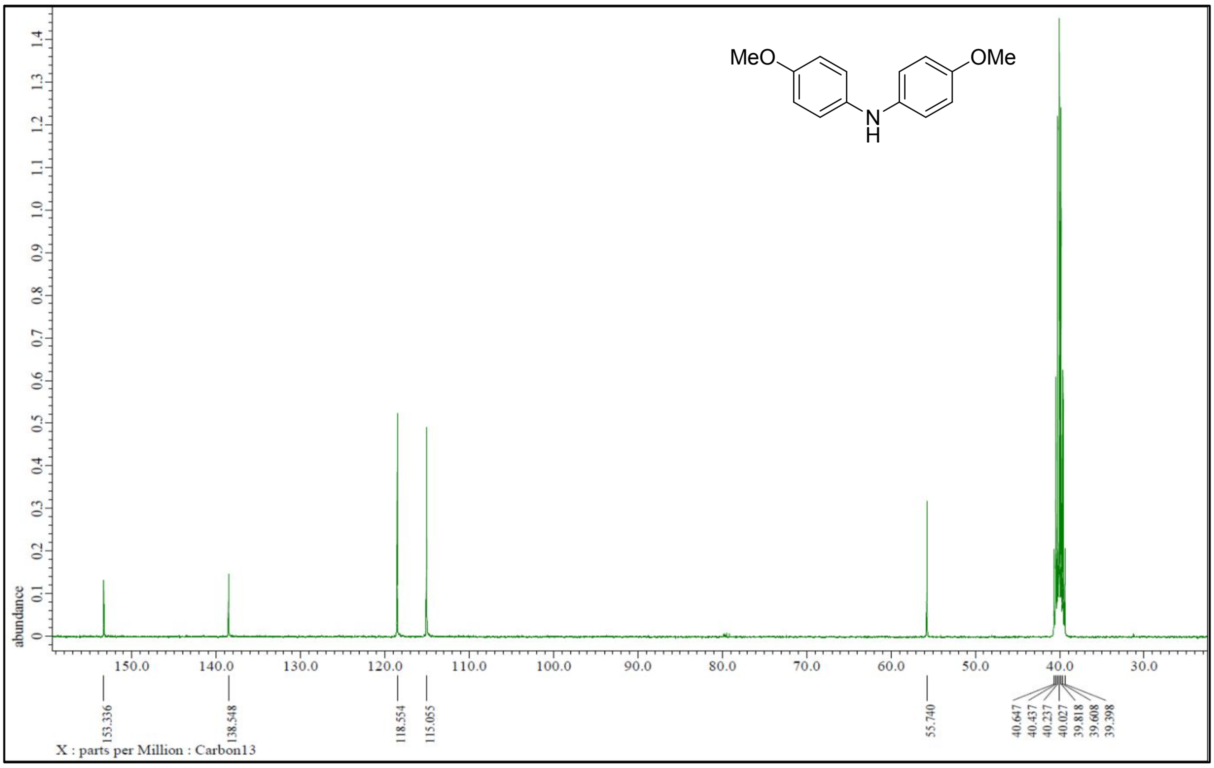

Figure S18: ^{13}C NMR spectrum **26(b)** (100 MHz, [D_6]DMSO).

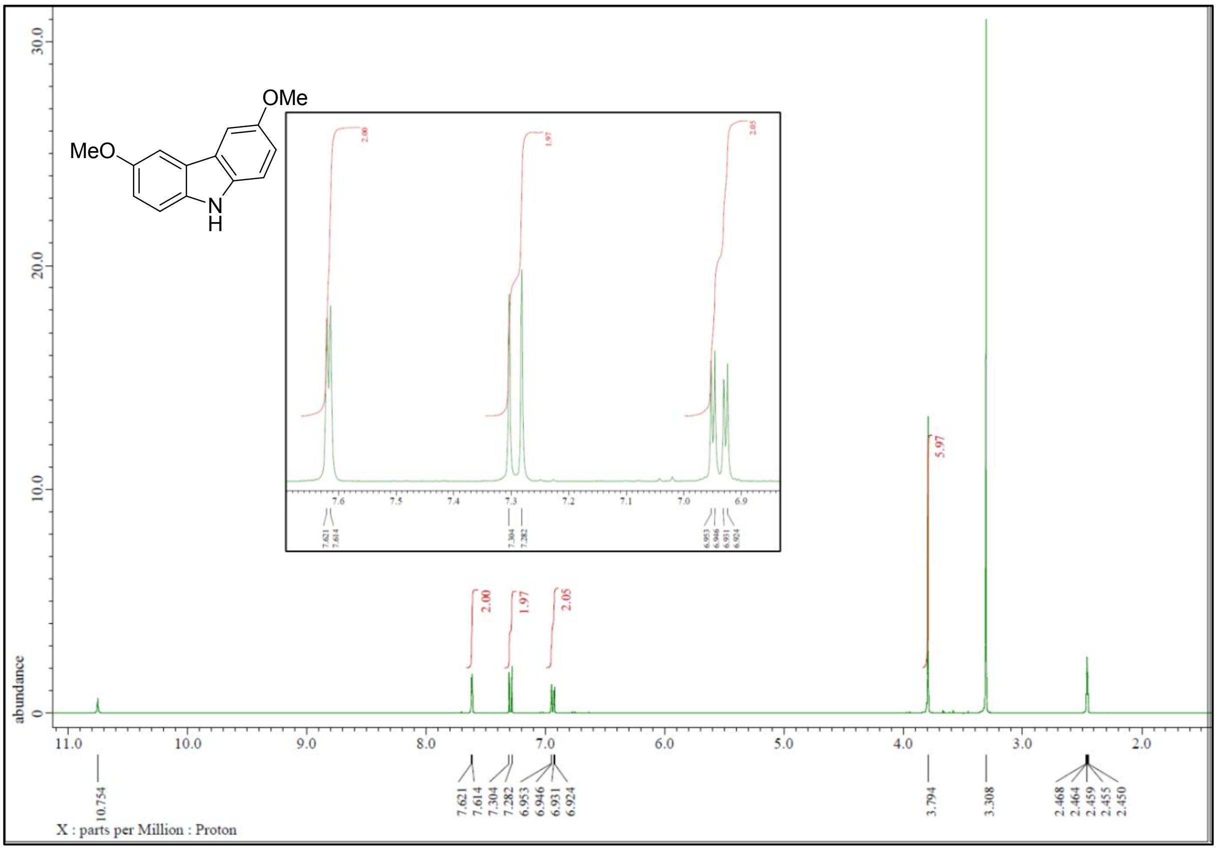

Figure S19: ^{1}H NMR spectrum **25(d)** (400 MHz, $[D_6]DMSO$).

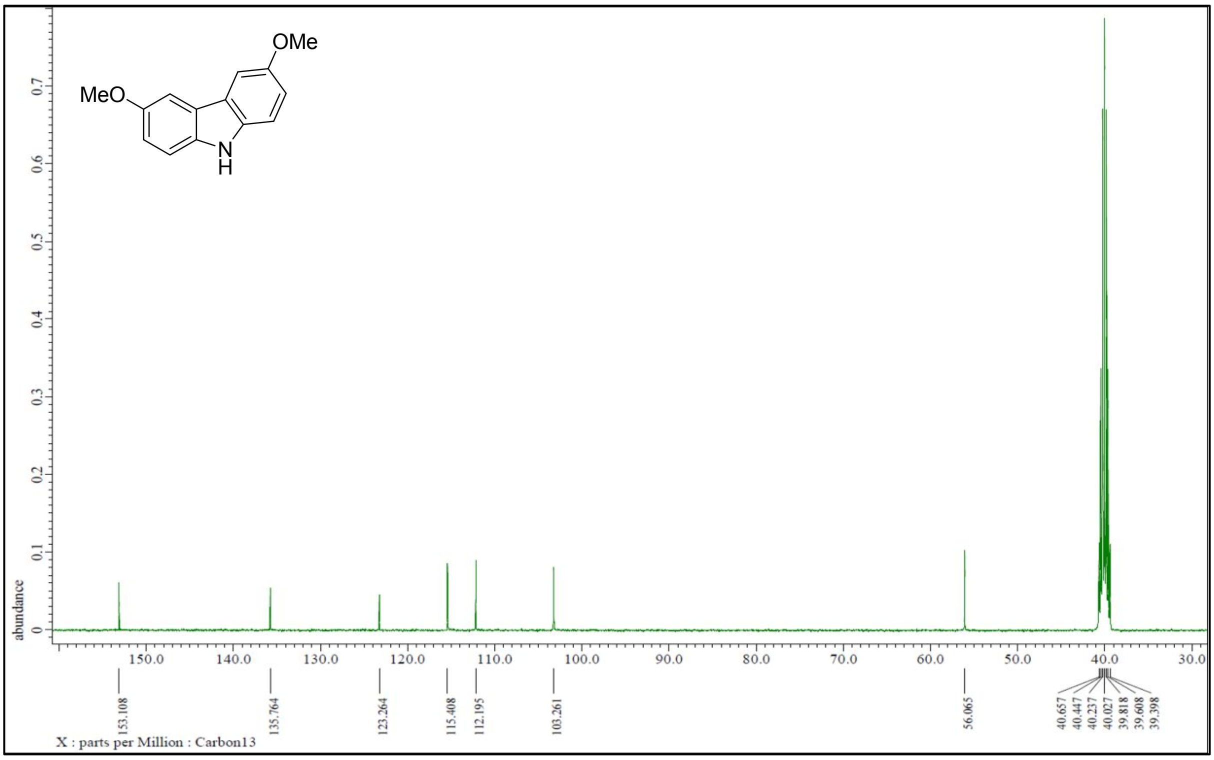

Figure S20: ^{13}C NMR spectrum **25(d)** (100 MHz, $[D_6]DMSO$).

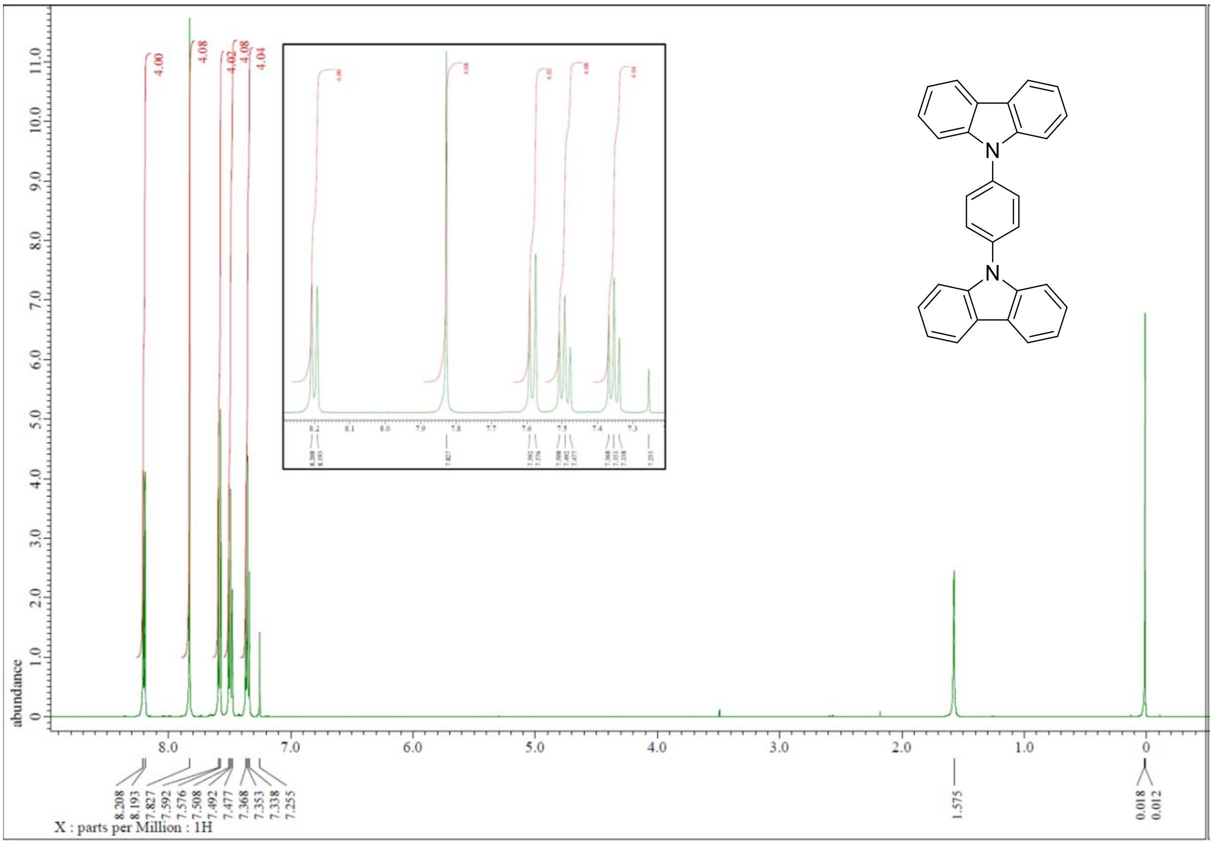

Figure S21: ^{1}H NMR spectrum **13(a)** (500 MHz, $CDCl_3$).

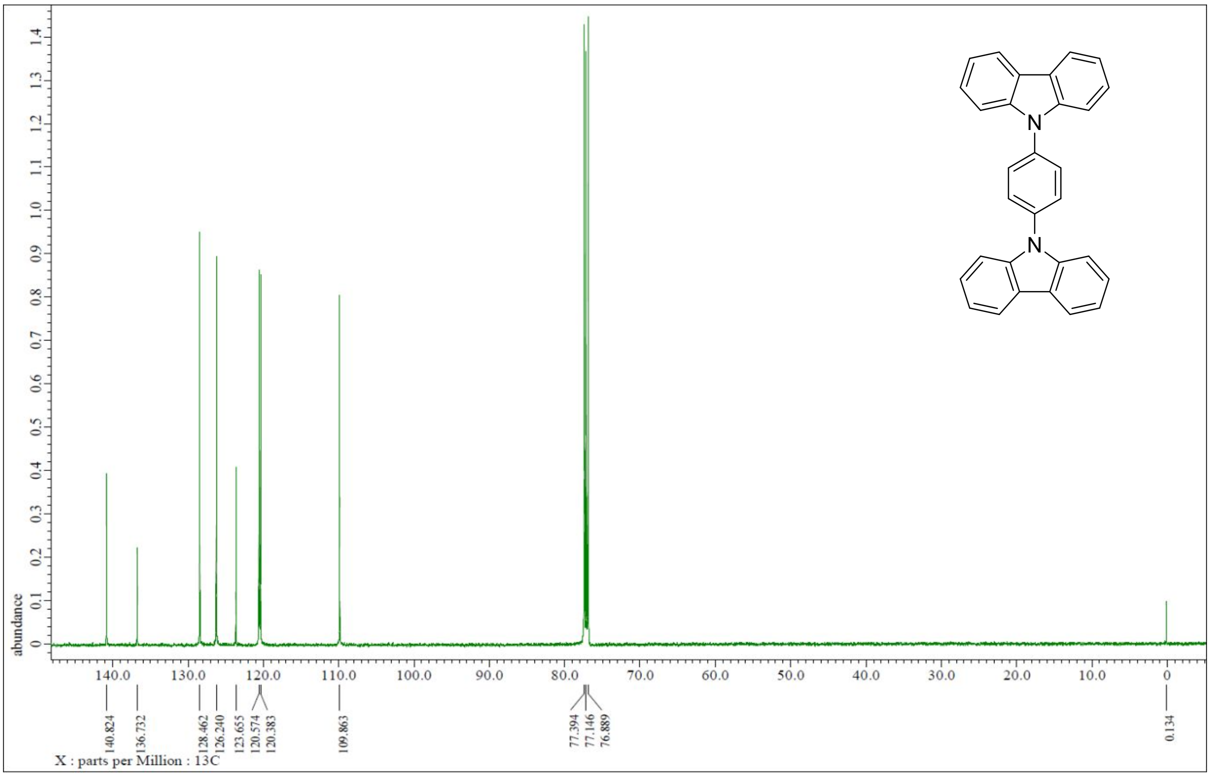

Figure S22: ^{13}C NMR spectrum **13(a)** (125 MHz, $CDCl_3$).

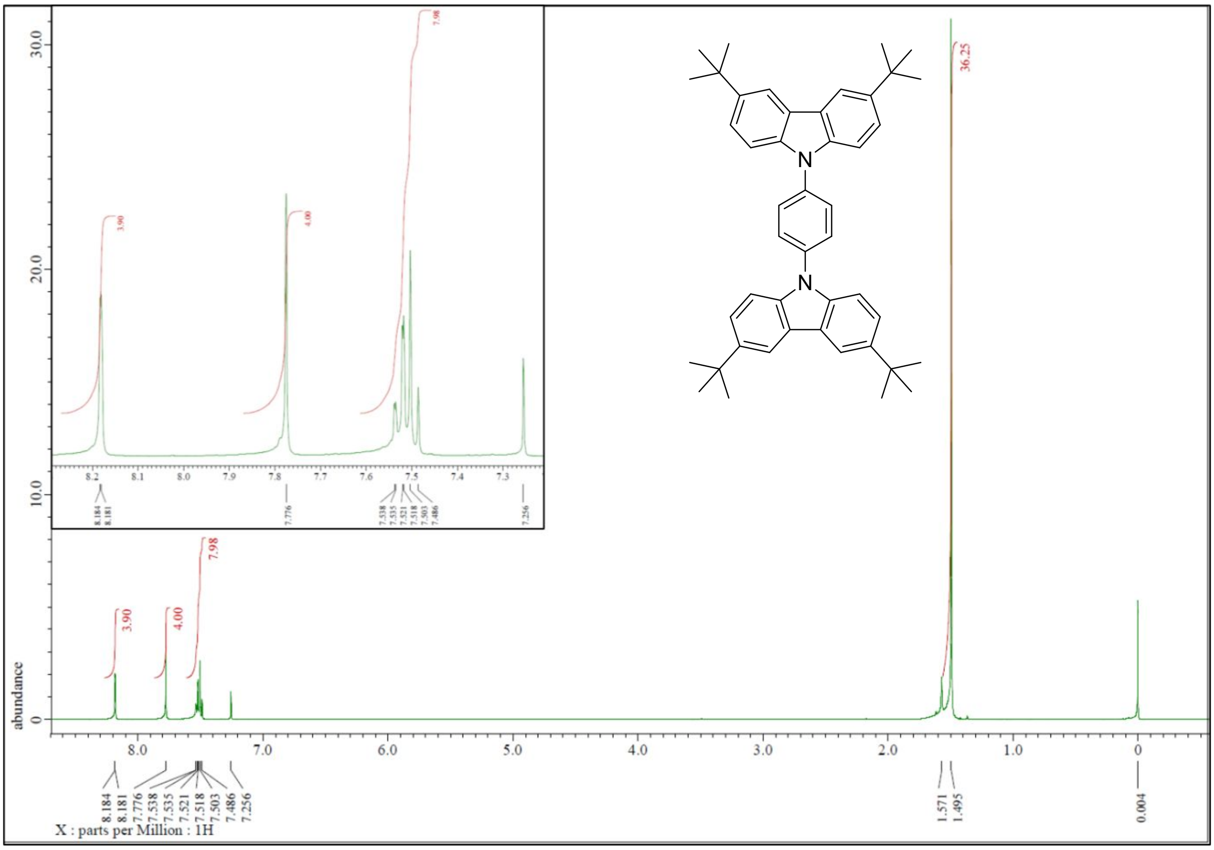

Figure S23: ^{1}H NMR spectrum **13(b)** (500 MHz, $CDCl_3$).

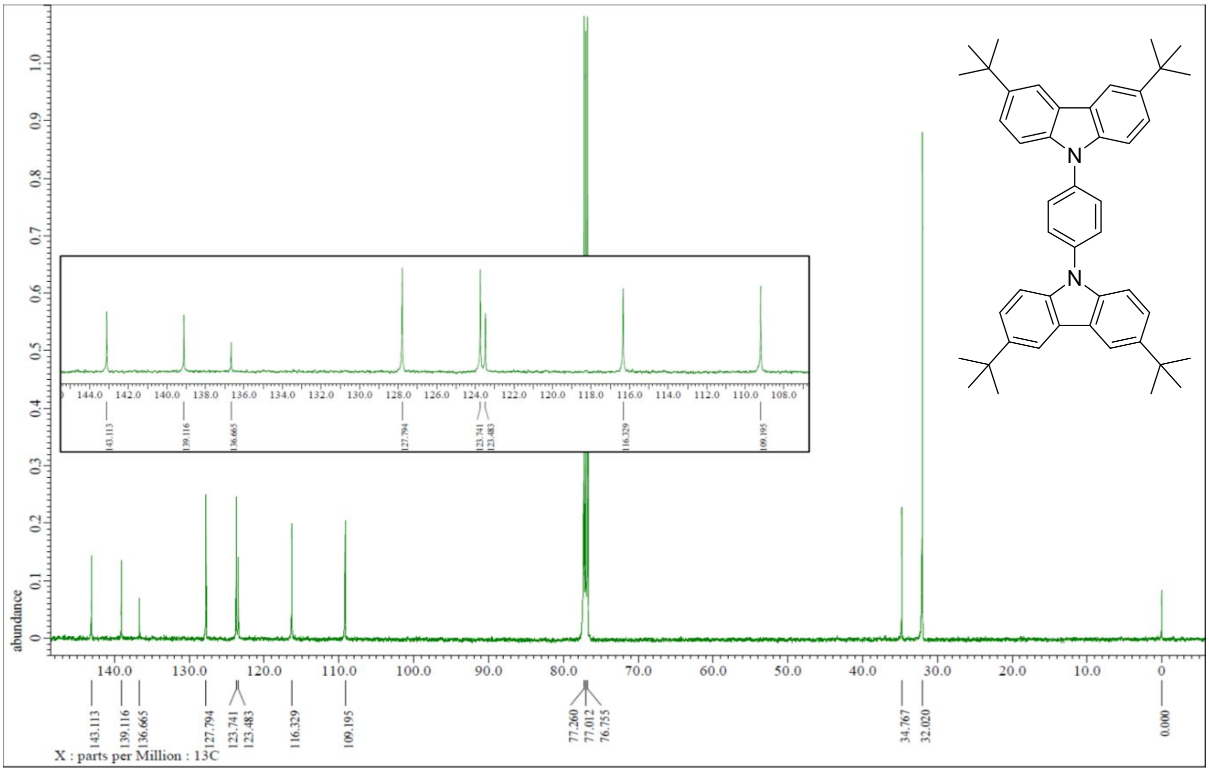

Figure S24: ^{13}C NMR spectrum **13(b)** (125 MHz, $CDCl_3$).

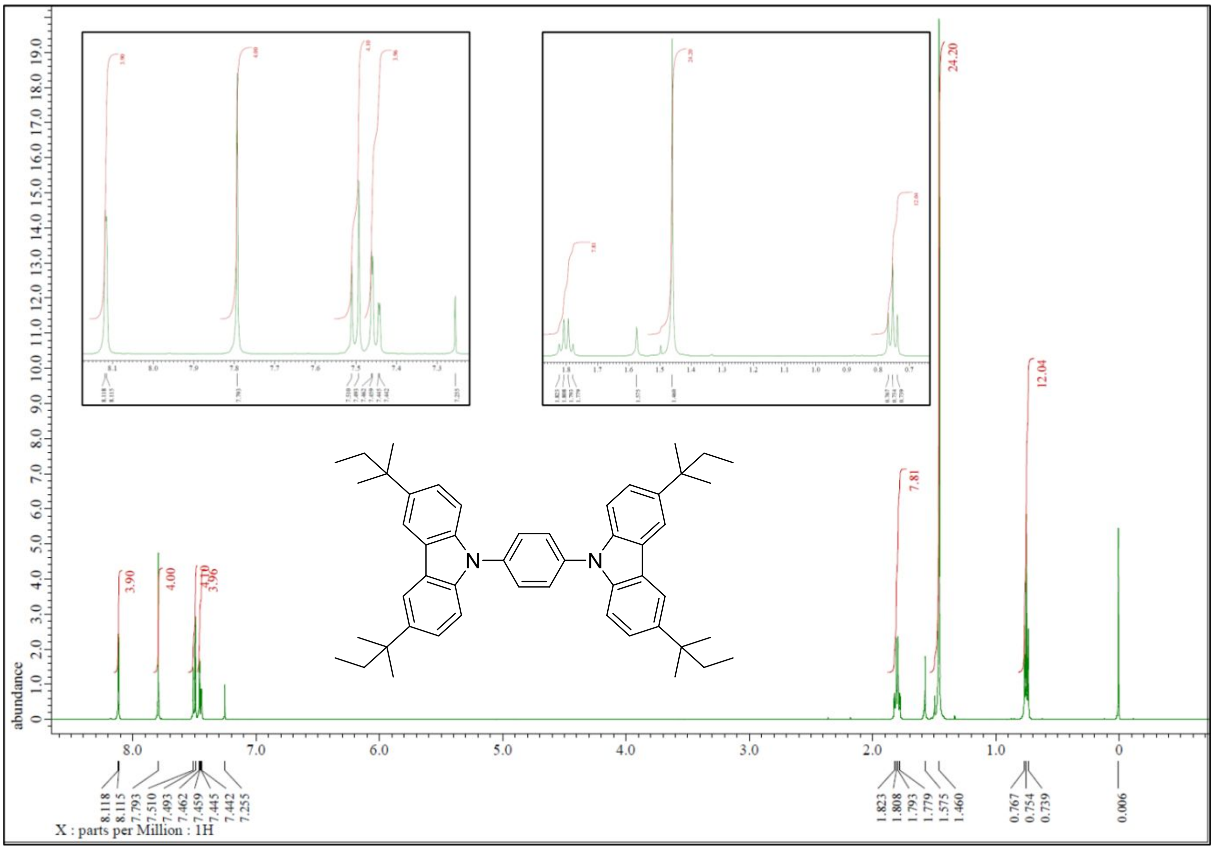

Figure S25: ^{1}H NMR spectrum **13(c)** (500 MHz, $CDCl_3$).

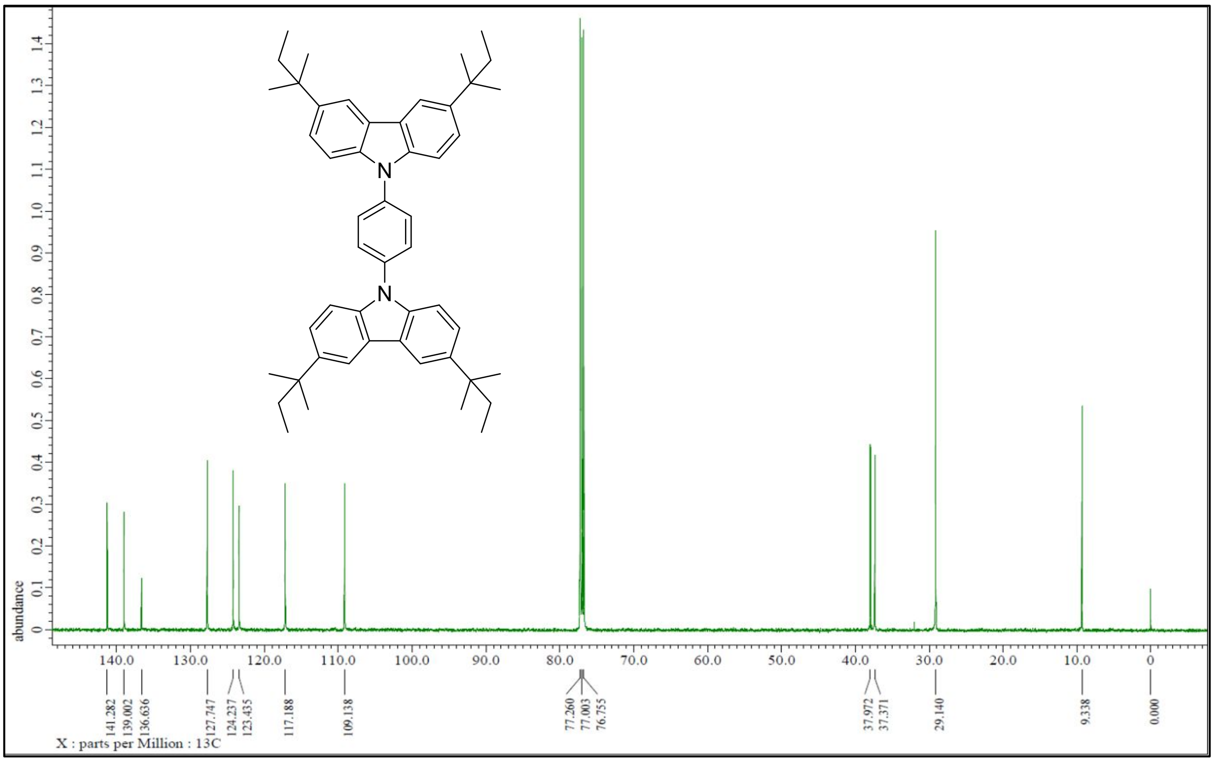

Figure S26: ^{13}C NMR spectrum **13(c)** (125 MHz, $CDCl_3$).

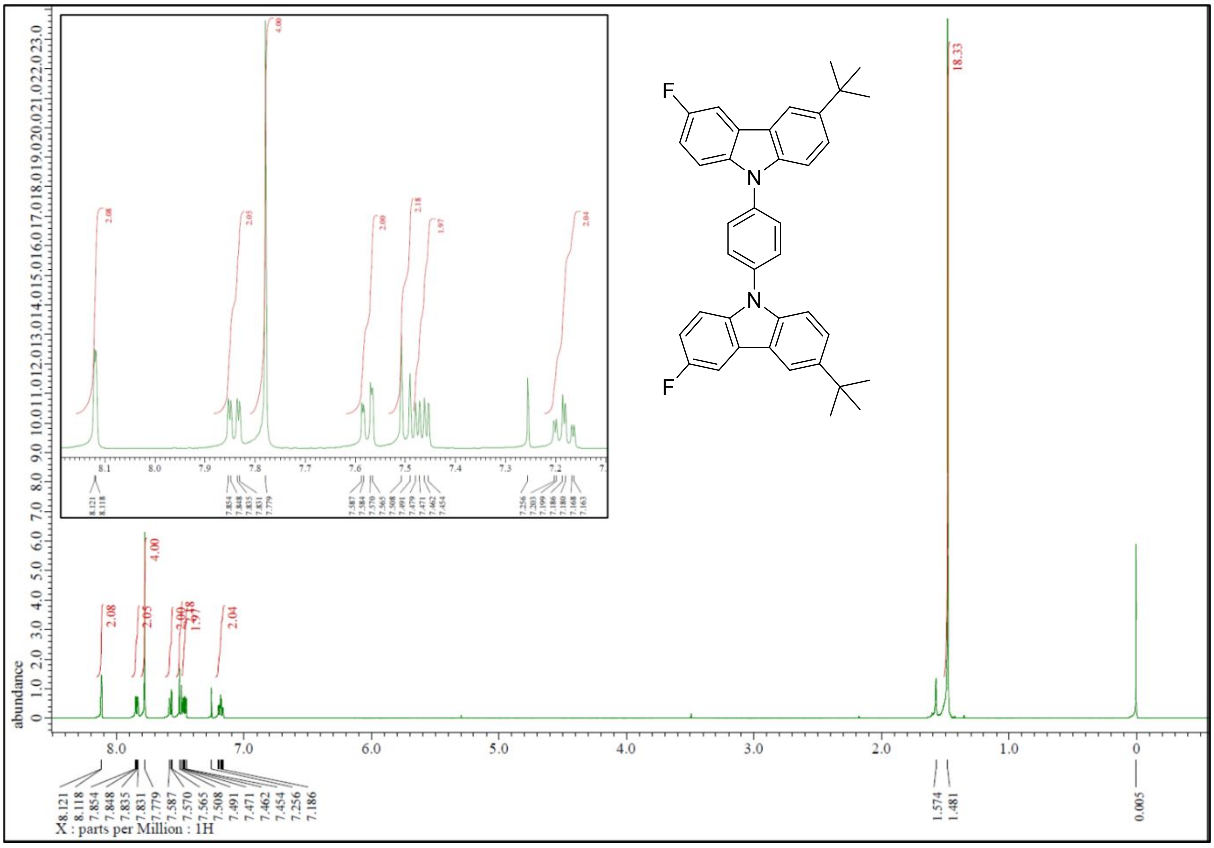

Figure S27: ^{1}H NMR spectrum **13(e)** (400 MHz, $CDCl_3$).

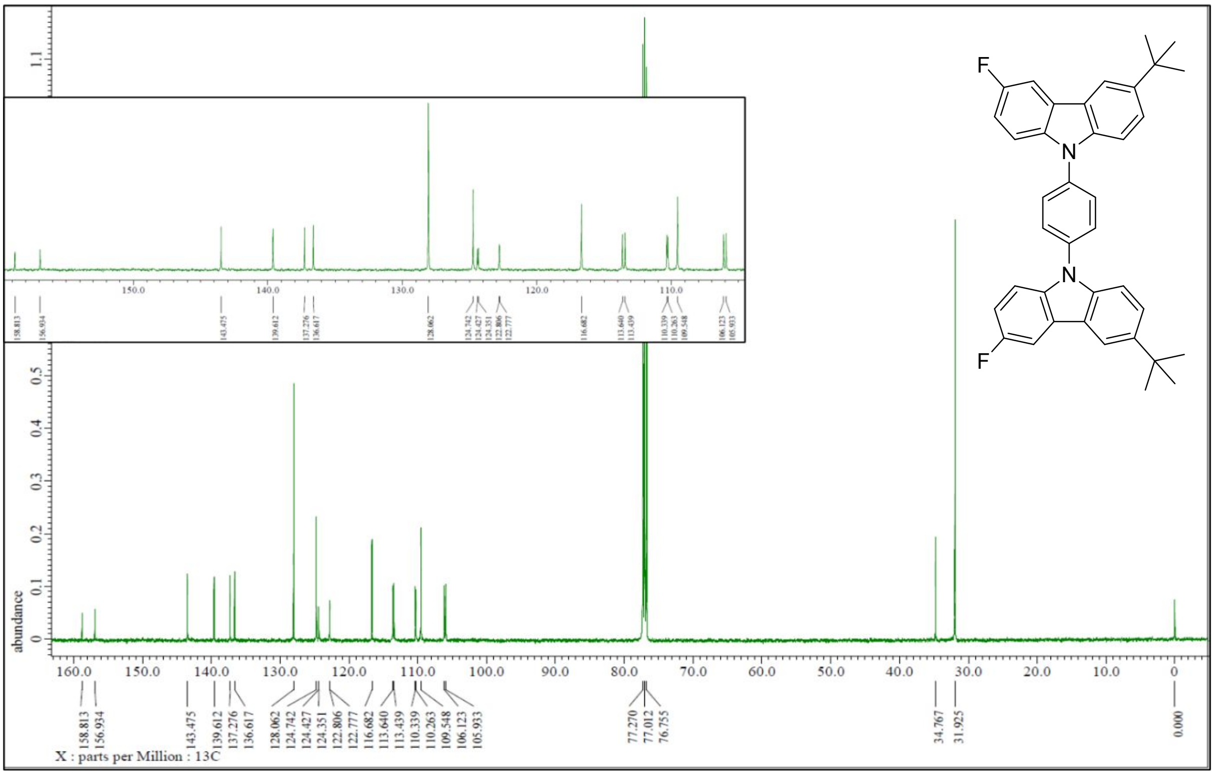

Figure S28: ^{13}C NMR spectrum **13(e)** (125 MHz, $CDCl_3$).

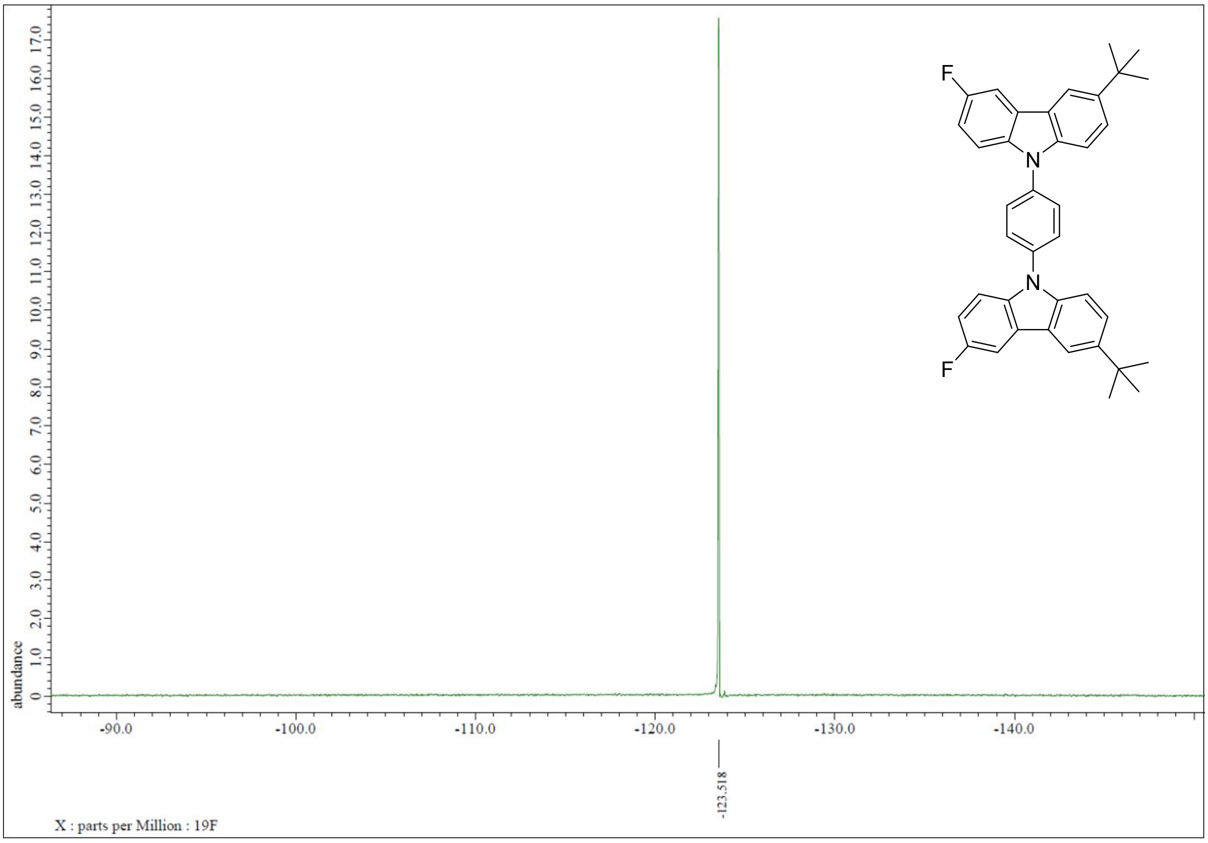

Figure S29: ^{19}F NMR spectrum **13(e)** (470 MHz, $CDCl_3$).

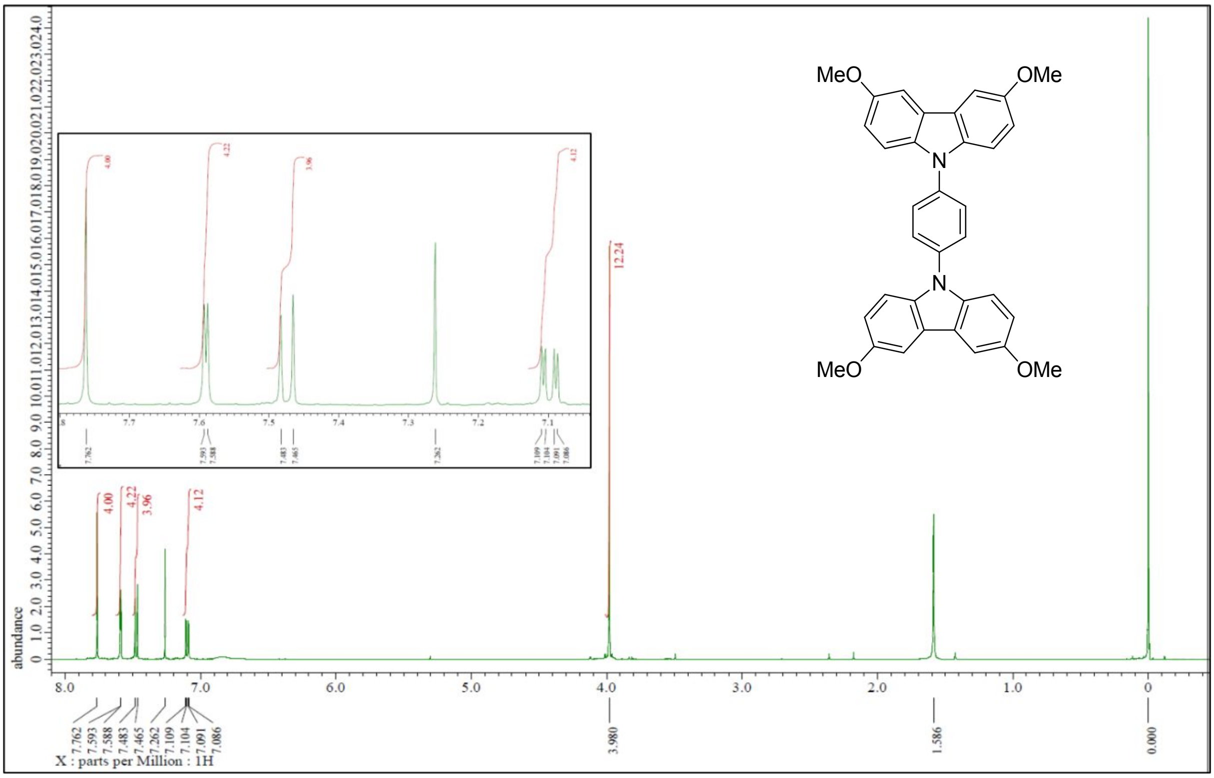

Figure S30: ^{1}H NMR spectrum **13(d)** (500 MHz, $CDCl_3$).

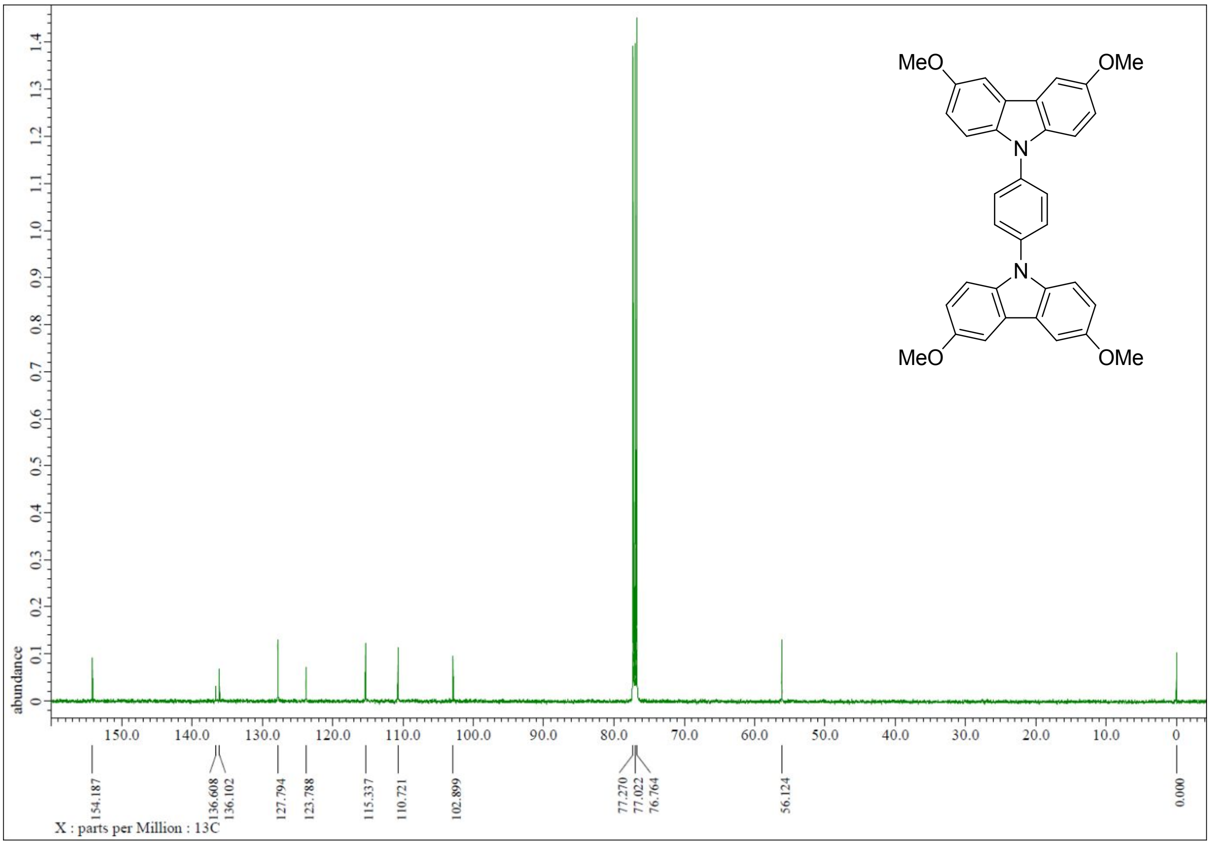

Figure S31: ^{13}C NMR spectrum **13(d)** (125 MHz, $CDCl_3$).

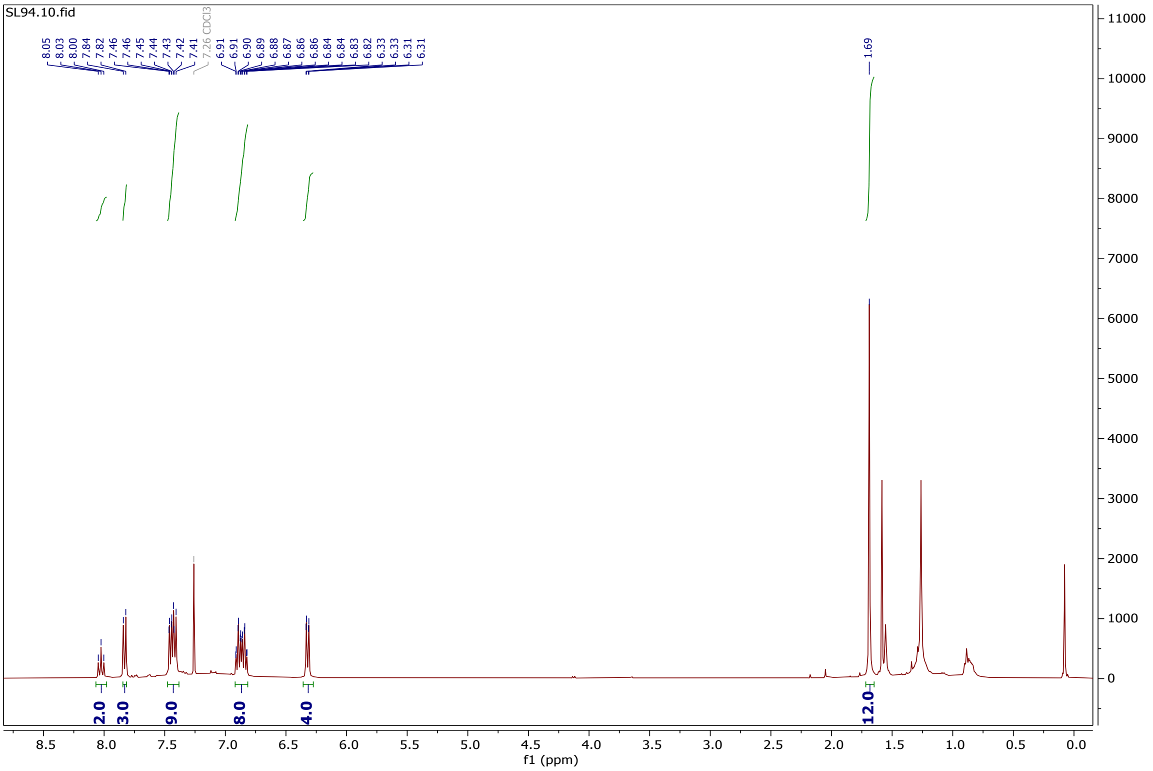

Figure S32: ^{1}H NMR spectrum **17** (400 MHz, $CDCl_3$)

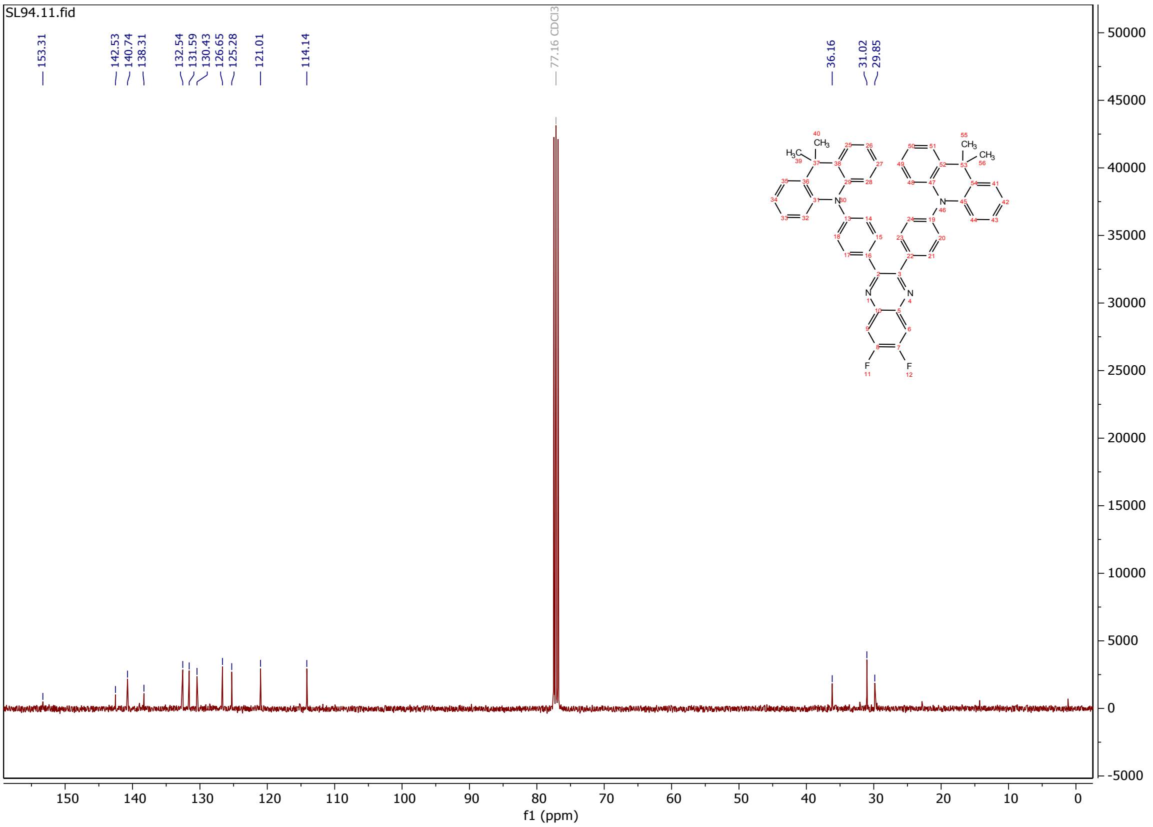

Figure S33: ^{13}C NMR spectrum **17** (100 MHz, $CDCl_3$).

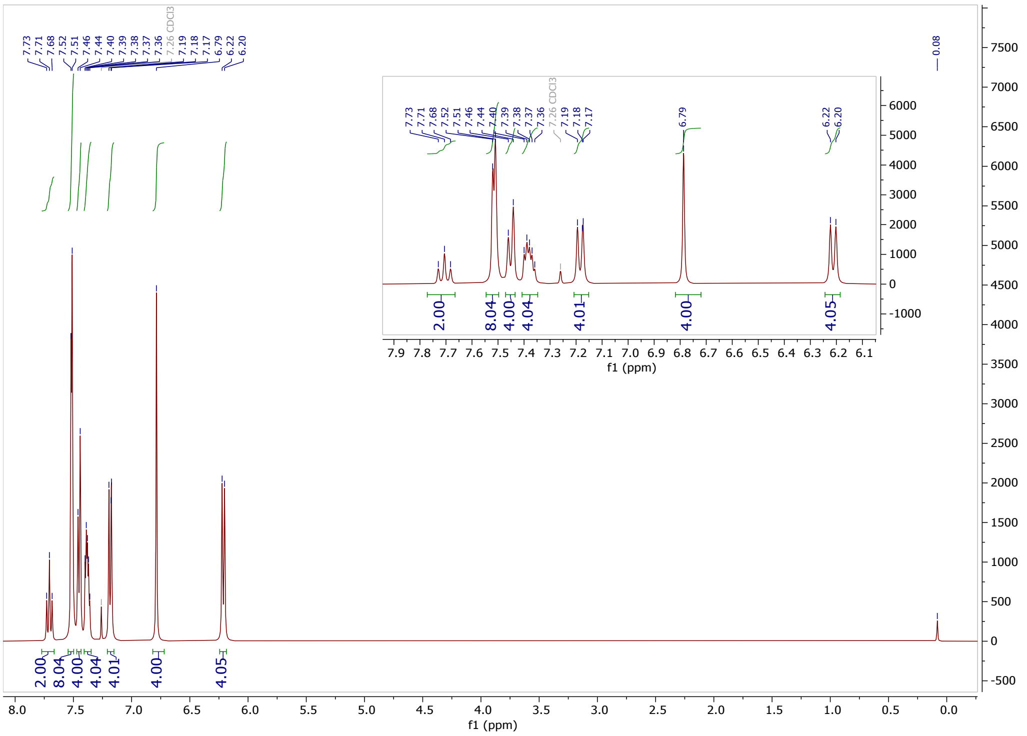

Figure S34: ^{1}H NMR spectrum **23** (400 MHz, $CDCl_3$).

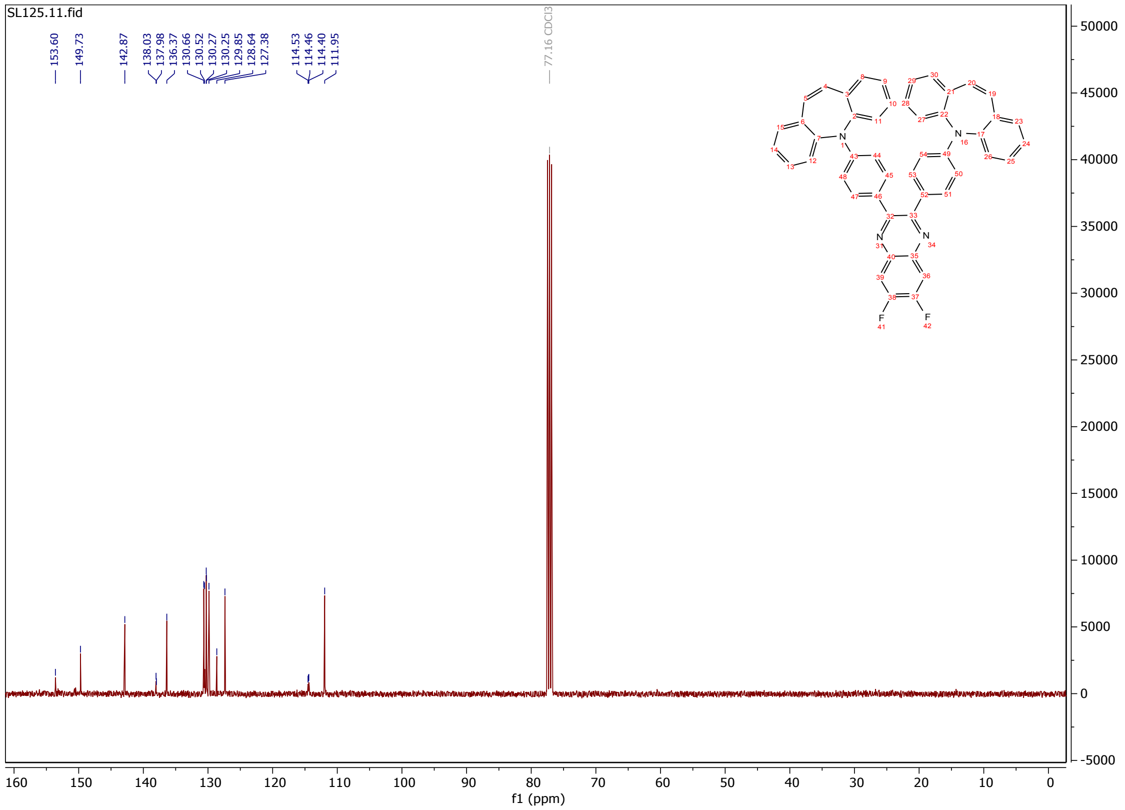

Figure S35: ^{13}C NMR spectrum **23** (100 MHz, $CDCl_3$).

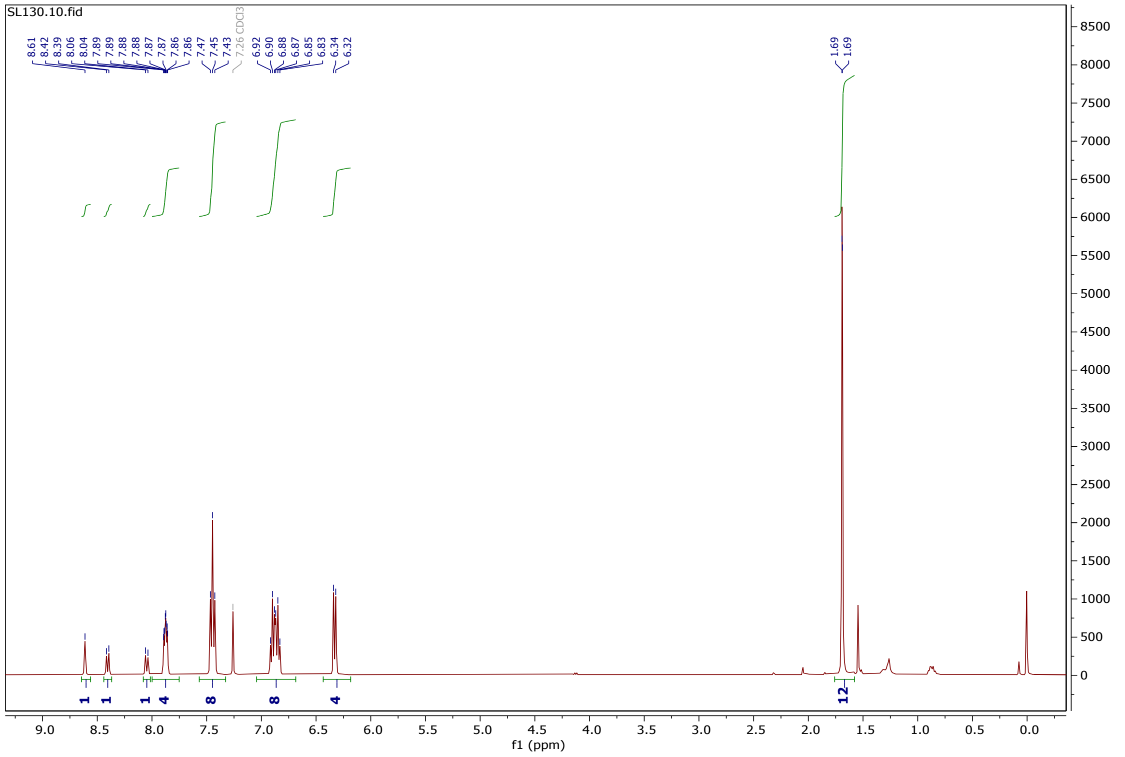

Figure S36: ^{1}H NMR spectrum **18** (400 MHz, $CDCl_3$).

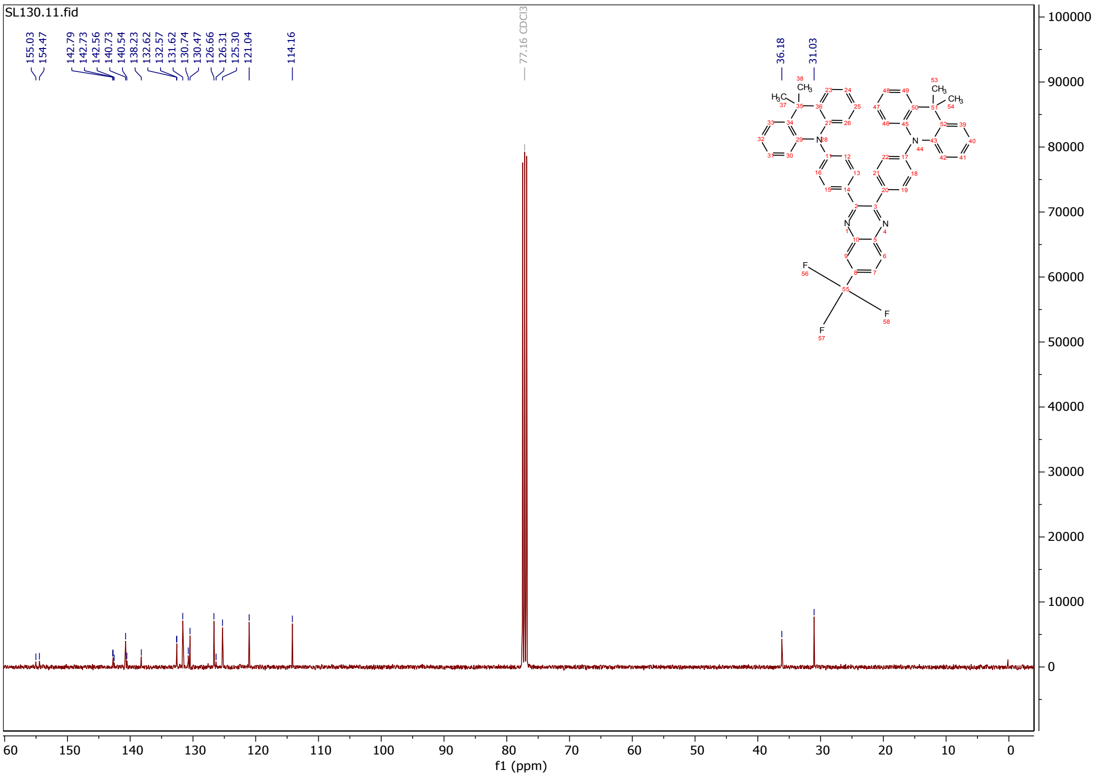

Figure S37: ^{13}C NMR spectrum **18** (100 MHz, $CDCl_3$).

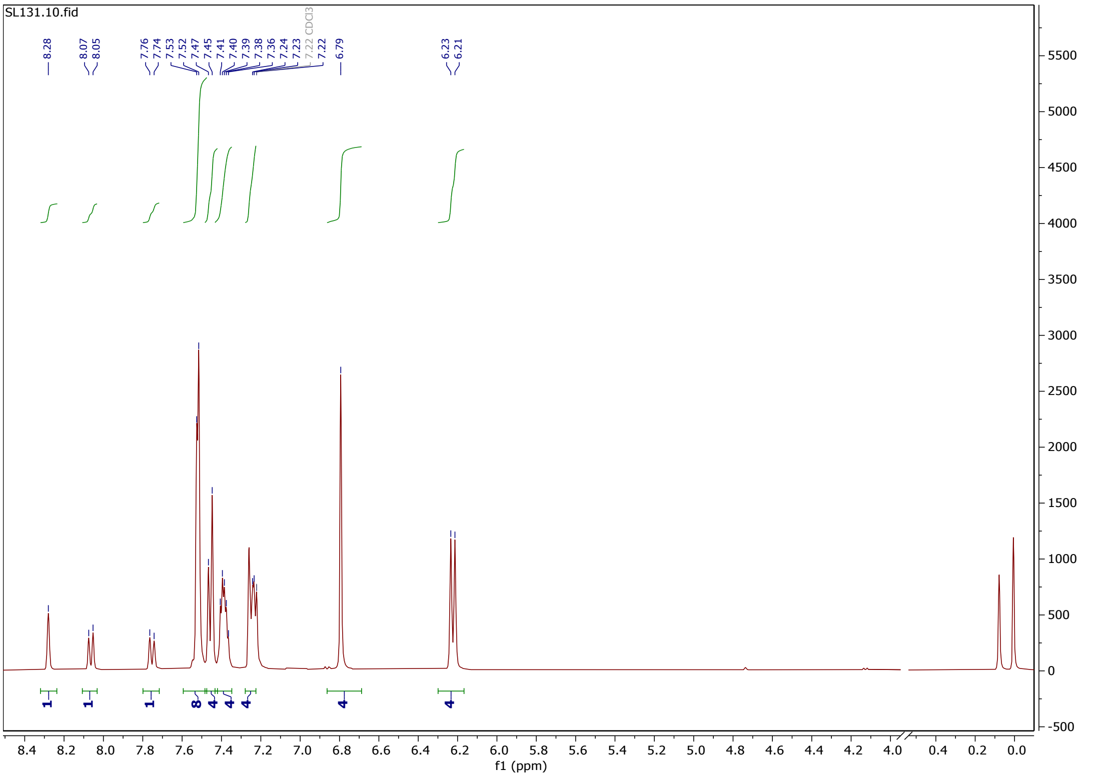

Figure S38: ^{1}H NMR spectrum **24** (400 MHz, $CDCl_3$).

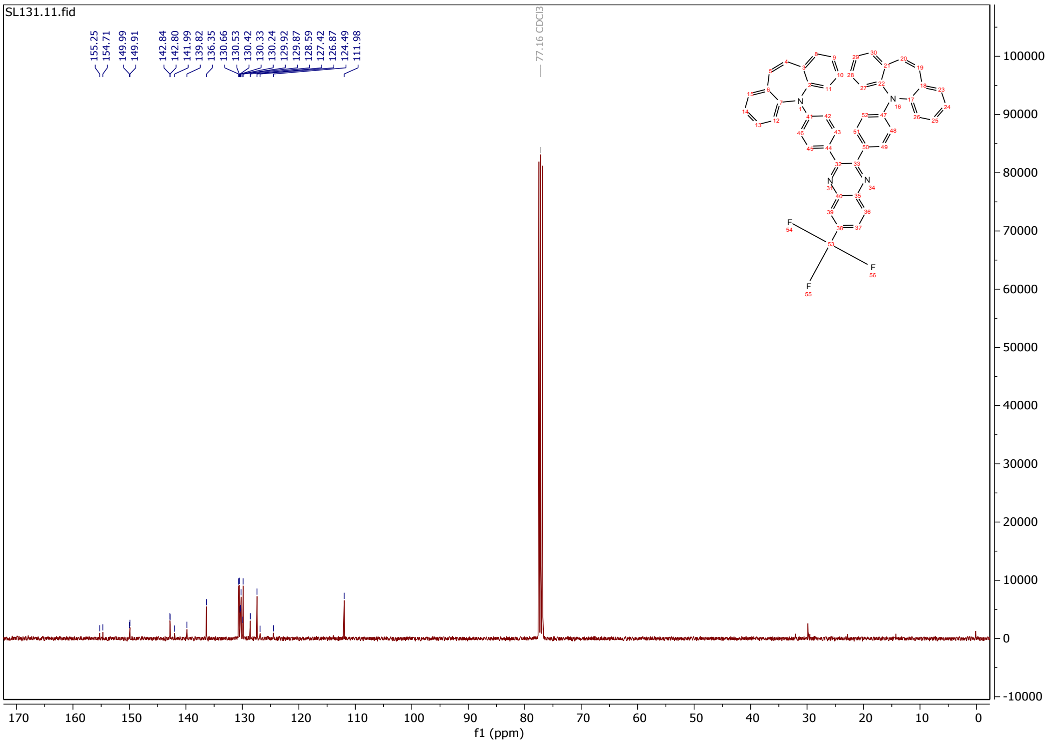

Figure S39: ^{13}C NMR spectrum **24** (100 MHz, $CDCl_3$).

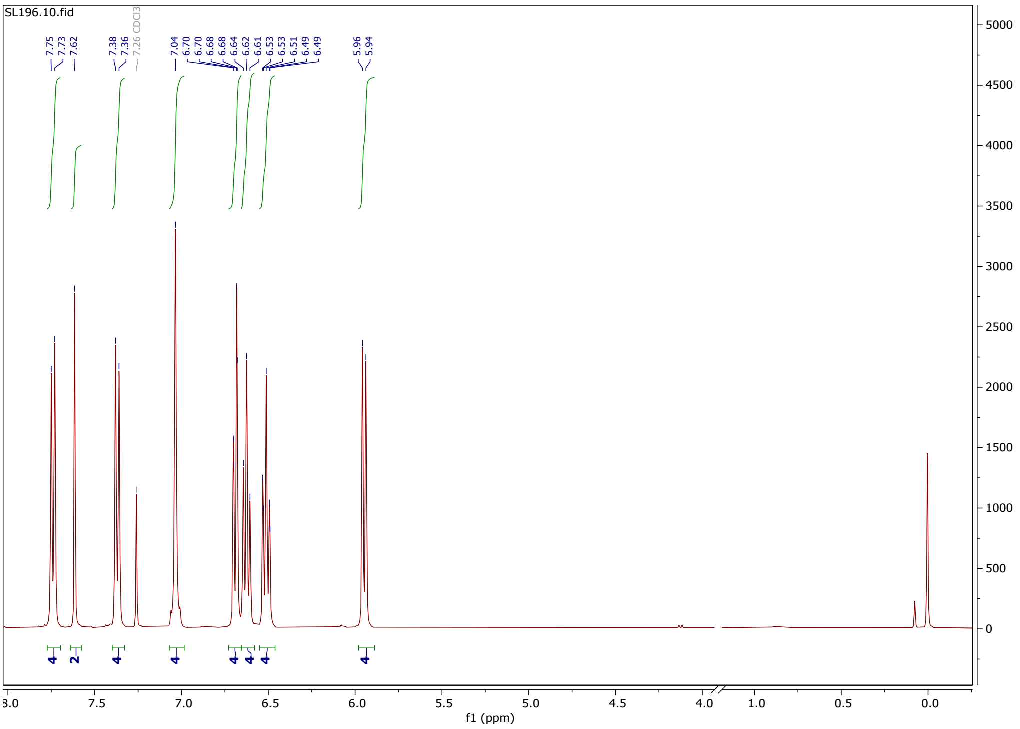

Figure S40: ^{1}H NMR spectrum **22** (400 MHz, $CDCl_3$).

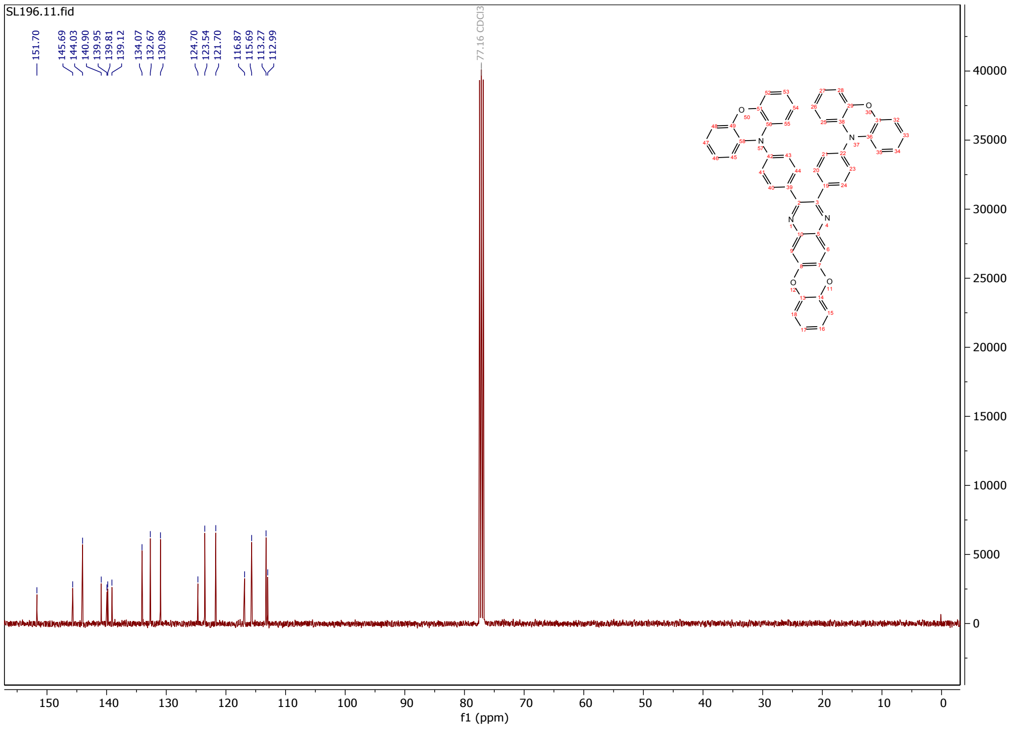

Figure S41: ^{13}C NMR spectrum **22** (100 MHz, $CDCl_3$).

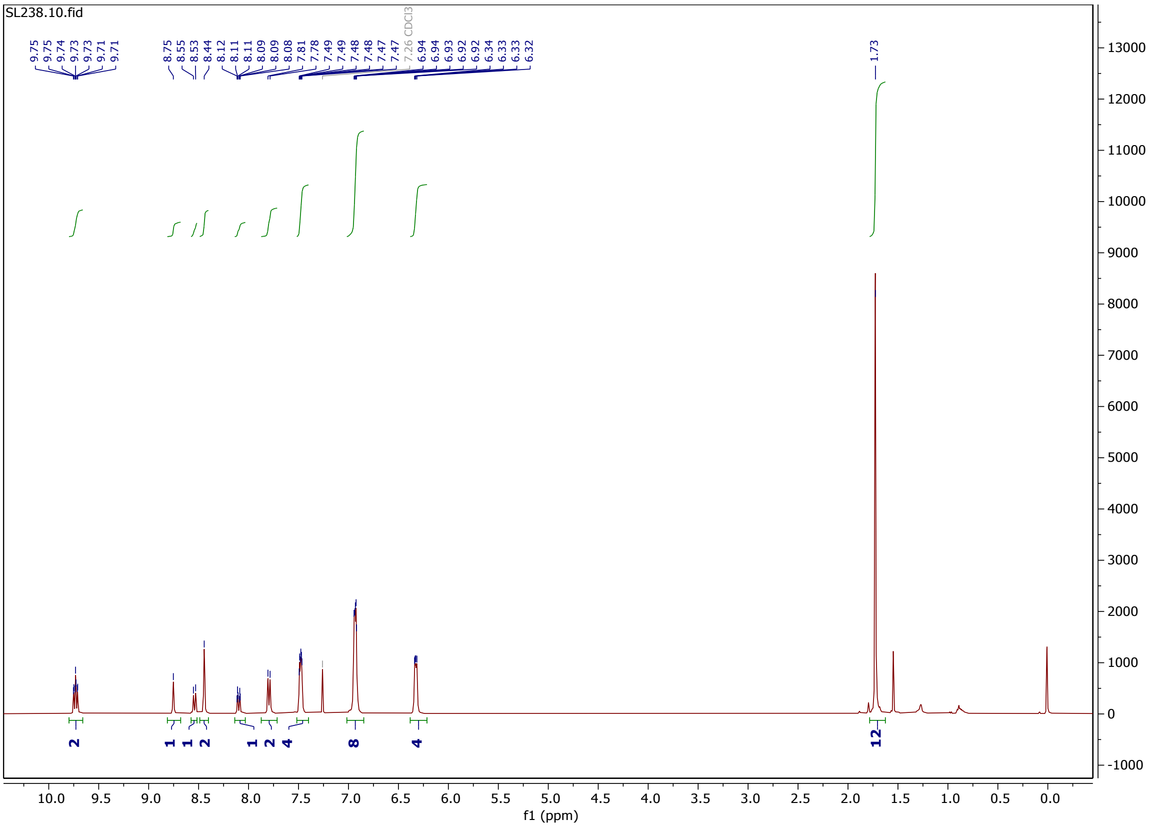

Figure S42: ^{1}H NMR spectrum **19** (400 MHz, $CDCl_3$).

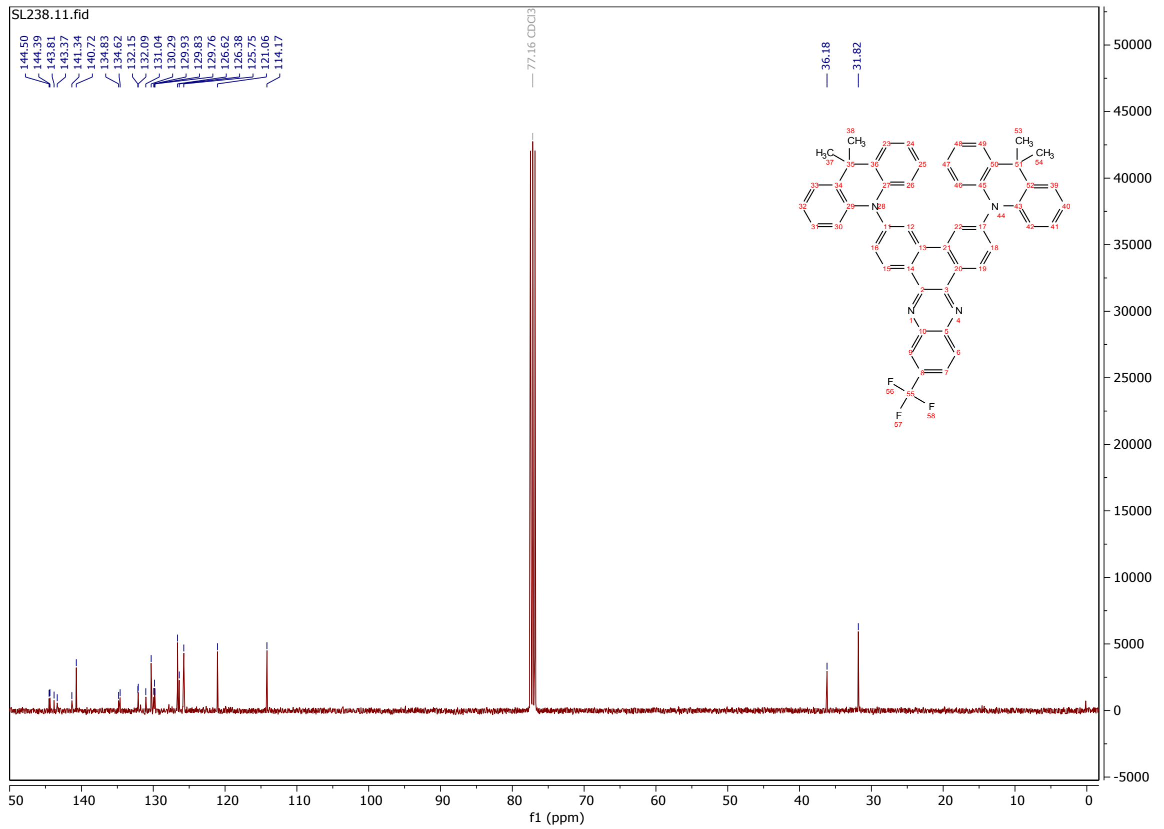

Figure S43: ^{13}C NMR spectrum **19** (100 MHz, $CDCl_3$).

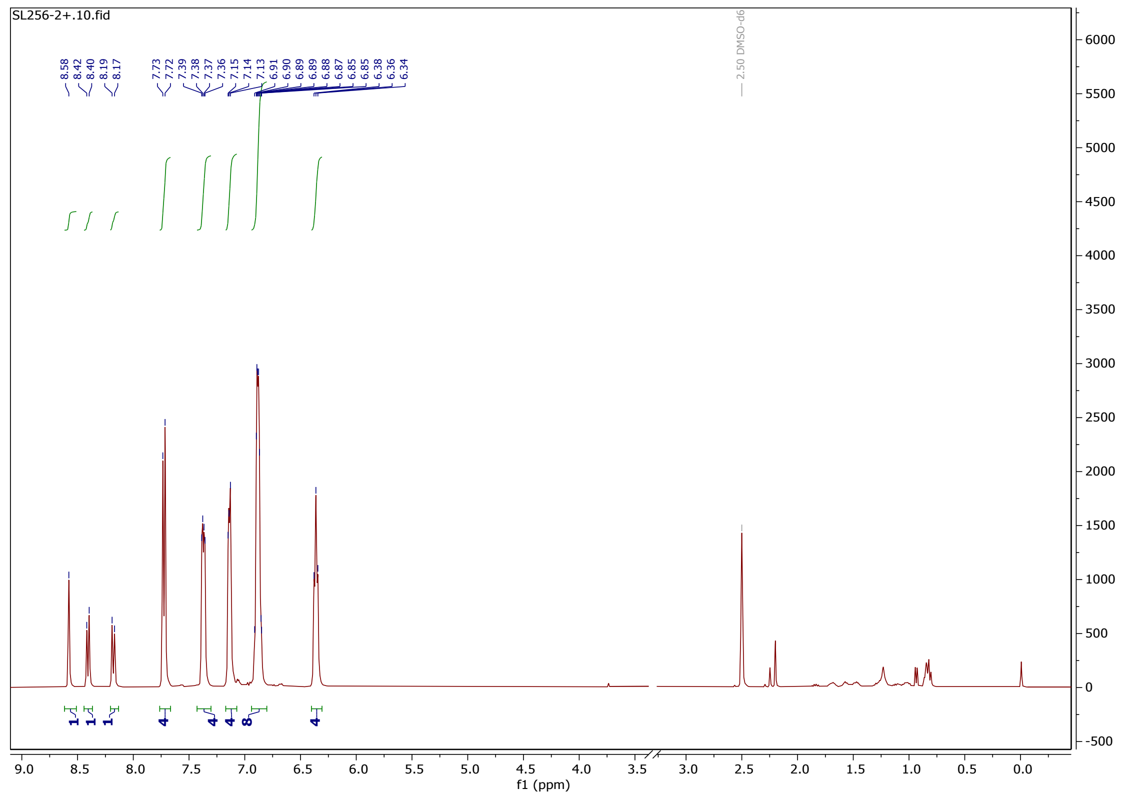

Figure S44: ^{1}H NMR spectrum **20** (400 MHz, [D$_6$]DMSO).

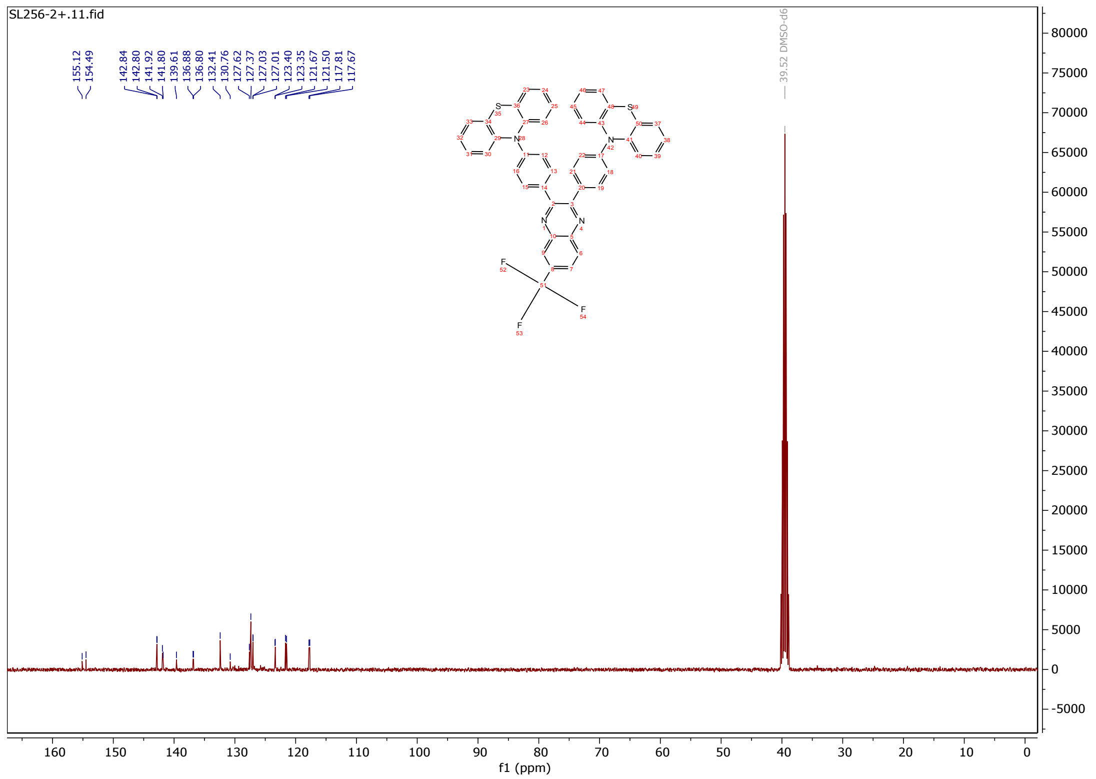

Figure S45: ^{13}C NMR spectrum **20** (100 MHz, [D$_6$]DMSO).

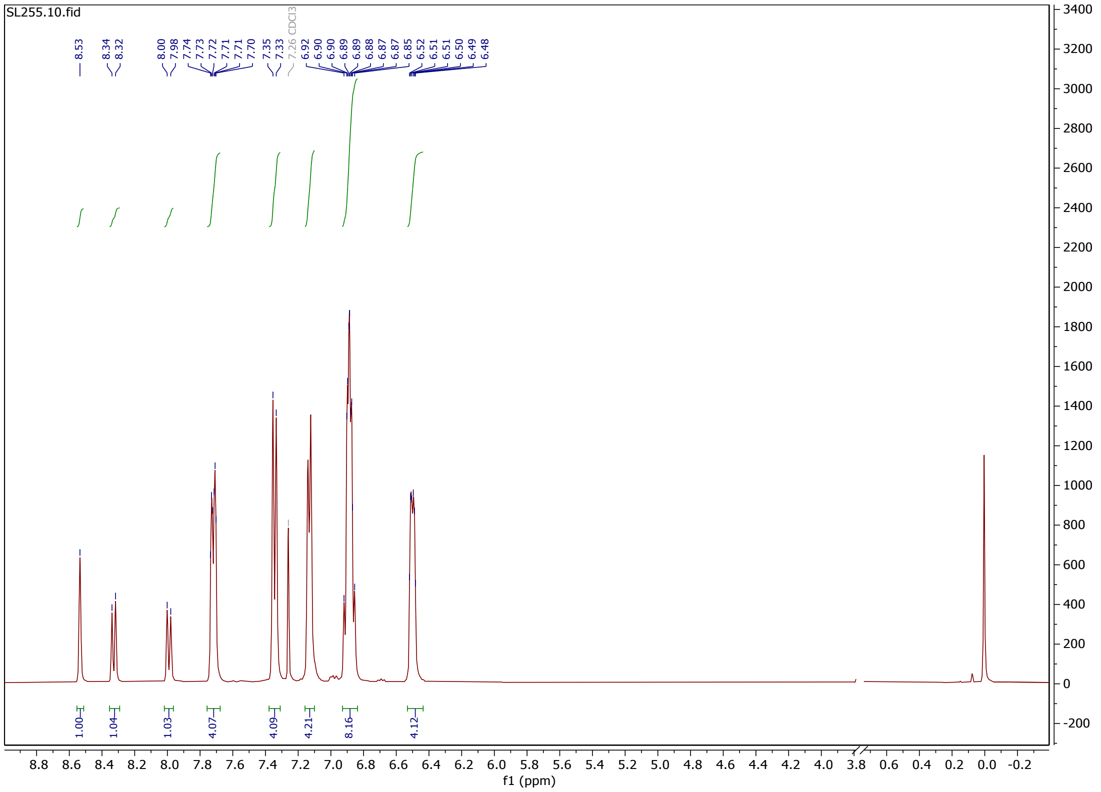

Figure S46: ^{1}H NMR spectrum **21** (400 MHz, $CDCl_3$).

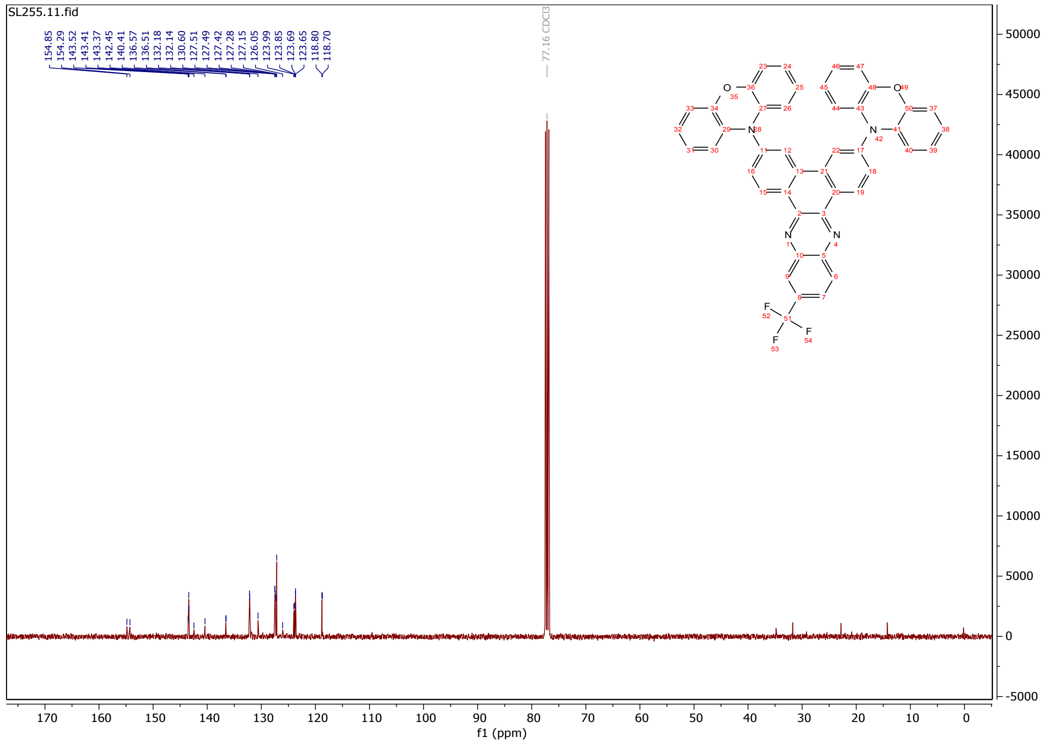

Figure S47: ^{13}C NMR spectrum **21** (100 MHz, $CDCl_3$).

5. DFT Computation (Mechanistic Studies)

Table S2. Absolute energies for the calculated structures involved in the mechanistic study.

Structures	$G_{correction}$ (Hartree)	E (Hartree)	G (Hartree)	v_{imag} (cm^{-1})
**Cat (L_nPd)**	0.641981	-1756.534409	-1755.892428	
**RE1_P_Cz_R=H**	0.177376	-3322.156794	-3321.979418	
**RE1_R_Cz_R=H**	0.854250	-5078.724317	-5077.870067	
**RE1_TS_Cz_R=H**	0.854630	-5078.681270	-5077.826640	-361.19
**RE2_P_Cz_R=H**	0.330440	-1264.827062	-1264.496622	
**RE2_R_Cz_R=H**	1.010375	-3021.396040	-3020.385665	
**RE2_TS_Cz_R=H**	1.007477	-3021.352269	-3020.344792	-346.56
**RE1_P_Ac_R=F**	0.231500	-3638.592407	-3638.360907	
**RE1_R_Ac_R=F**	0.916884	-5395.162622	-5394.245738	
**RE1_TS_Ac_R=F**	0.912764	-5395.127439	-5394.214675	-367.09
**RE2_P_Ac_R=F**	0.461728	-1699.200521	-1698.738793	
**RE2_R_Ac_R=F**	1.145922	-3455.771530	-3454.625608	
**RE2_TS_Ac_R=F**	1.144427	-3455.740370	-3454.595943	-363.17
**RE1_P_Ac_R=CF3**	0.248904	-4114.286435	-4114.037531	
**RE1_R_Ac_R=CF3**	0.928245	-5870.854689	-5869.926444	
**RE1_TS_Ac_R=CF3**	0.932458	-5870.822027	-5869.889569	-355.59
**RE2_P_Ac_R=CF3**	0.475600	-2174.899089	-2174.423489	
**RE2_R_Ac_R=CF3**	1.158451	-3931.469136	-3930.310685	
**RE2_TS_Ac_R=CF3**	1.161712	-3931.437712	-3930.276000	-350.04
**RE1_P_Cz_R=CF3**	0.169474	-3996.348711	-3996.179237	
**RE1_R_Cz_R=CF3**	0.851037	-5752.921509	-5752.070472	
**RE1_TS_Cz_R=CF3**	0.853738	-5752.865083	-5752.011345	-352.37
**RE2_P_Cz_R=CF3**	0.319373	-1939.021922	-1938.702549	
**RE2_R_Cz_R=CF3**	1.001523	-3695.595236	-3694.593713	
**RE2_TS_Cz_R=CF3**	1.001667	-3695.543739	-3694.542072	-245.99

6. Optimized structures from DFT Calculations involved in mechanistic study

Cat (L_nPd)

Pd	-0.3450591	-1.2485138	-1.2928397
P	1.4255259	-0.0681558	-0.4712917
C	3.1014369	-0.8648768	-0.3336437
H	3.7795739	-0.2164428	0.2364873
C	2.9850549	-2.2063248	0.4052893
H	2.2581909	-2.8363938	-0.1247287
H	2.5872899	-2.0465208	1.4135423
C	4.3319909	-2.9293568	0.4800173
H	5.0246339	-2.3409228	1.0976033
H	4.2069109	-3.8960238	0.9798213
C	4.9375839	-3.1227438	-0.9121467
H	4.2926729	-3.7939718	-1.4955197
H	5.9166309	-3.6083298	-0.8381807
C	5.0611539	-1.7858988	-1.6466907
H	5.7791049	-1.1468268	-1.1140767
H	5.4633829	-1.9389938	-2.6539907
C	3.7118049	-1.0642638	-1.7291087
H	3.0143529	-1.6521938	-2.3414337
H	3.8396189	-0.0988878	-2.2318817
C	1.7727629	1.6166592	-1.1969777
H	2.0156059	1.3930162	-2.2462277
C	0.4757649	2.4372542	-1.1871897
H	-0.3305691	1.8535432	-1.6465007
H	0.1804639	2.6165382	-0.1443007
C	0.6466129	3.7820752	-1.8973587
H	0.8357929	3.6051922	-2.9649597
H	-0.2846141	4.3559912	-1.8322397
C	1.8092539	4.5806152	-1.3032747
H	1.5704289	4.8434642	-0.2634657
H	1.9442209	5.5229202	-1.8450467
C	3.1027859	3.7625782	-1.3254877
H	3.9224129	4.3297042	-0.8707787
H	3.3907569	3.5701172	-2.3681687
C	2.9279169	2.4253682	-0.5956527
H	2.7174229	2.6264782	0.4621693
H	3.8642989	1.8569332	-0.6360837
C	1.0211529	0.3155452	1.3128533
C	2.0409969	0.7050092	2.1912783
C	1.7997869	0.9813672	3.5321493
H	2.6181349	1.2812472	4.1792273
C	0.5096009	0.8654342	4.0322973
H	0.3023159	1.0697632	5.0779983
C	-0.5168871	0.4728552	3.1827413
C	-0.2944161	0.1964452	1.8295543

C	-1.4527821	-0.2427898	0.9862853
C	-2.4412031	0.6474372	0.5693923
C	-2.5037351	2.0845062	1.0694783
H	-1.5296421	2.3333262	1.5026833
C	-2.7927671	3.1087062	-0.0368137
H	-2.1368521	2.9700662	-0.8984527
H	-2.6476551	4.1237462	0.3470263
H	-3.8271901	3.0409382	-0.3895457
C	-3.5575251	2.2159752	2.1804713
H	-4.5525931	1.9722872	1.7921683
H	-3.5870111	3.2386542	2.5712053
H	-3.3526451	1.5374552	3.0127623
C	-3.4240431	0.2050702	-0.3411057
H	-4.1900951	0.9046052	-0.6623817
C	-3.4130381	-1.0701088	-0.8721637
C	-4.4249341	-1.5049428	-1.9156017
C	-5.8678751	-1.3367378	-1.4231157
H	-6.5764641	-1.7236218	-2.1626767
H	-6.1063511	-0.2810818	-1.2561877
H	-6.0276961	-1.8684048	-0.4807157
C	-2.4397761	-1.9888328	-0.4127177
H	-2.5527061	-3.0354218	-0.6831057
C	-1.4832401	-1.6197378	0.5618253
C	-0.7634001	-2.6835468	1.3926823
H	0.1963779	-2.2633018	1.7082863
C	-1.5851541	-2.9767358	2.6574043
H	-1.7497681	-2.0686118	3.2432903
H	-1.0682491	-3.7032318	3.2928073
H	-2.5638941	-3.3902398	2.3905353
C	-0.4668551	-3.9833888	0.6419183
H	-1.3834231	-4.5356348	0.4073983
H	0.1566319	-4.6355118	1.2616303
H	0.0634929	-3.7860728	-0.2958087
H	-1.5244791	0.3663392	3.5721023
H	3.0581609	0.7926882	1.8255023
H	-4.2592881	-2.5734688	-2.1034607
C	-4.2014361	-0.7614318	-3.2394437
H	-4.3556831	0.3149532	-3.1082637
H	-3.1799611	-0.9115178	-3.6021247
H	-4.8991121	-1.1128528	-4.0069507

RE1_P_Cz_R=H

C	2.2918009	-0.7468417	0.8568291
C	1.2897359	-0.8642237	-0.1070289
C	3.5865069	-2.3985387	-0.2982439
C	3.4415749	-1.5238387	0.7709141
H	2.1703799	-0.0393807	1.6702081
H	4.2194339	-1.4357127	1.5201901

C	2.6051389	-2.5212037	-1.2733829
H	2.7309929	-3.2129507	-2.0979429
C	1.4482249	-1.7572947	-1.1672379
H	0.6588949	-1.8564667	-1.9047739
Br	5.1903489	-3.4683667	-0.4323179
C	-0.3751201	0.7719493	-0.9961109
C	-0.7383391	-0.0288647	1.0885221
C	0.1565679	1.0737853	-2.2496749
C	-1.5589841	1.3775723	-0.5280649
C	-0.6740001	-0.7295907	2.2927071
C	-1.7919961	0.8642193	0.8063181
C	-0.5347371	1.9834893	-3.0384489
H	1.0808819	0.6225473	-2.5937979
C	-2.2358201	2.2884713	-1.3400919
C	-1.6796081	-0.5023097	3.2226081
H	0.1262579	-1.4336027	2.4935671
C	-2.7906131	1.0759653	1.7577141
C	-1.7217261	2.5840203	-2.5946779
H	-0.1432451	2.2378713	-4.0183639
H	-3.1496091	2.7604593	-0.9918369
C	-2.7269181	0.3937953	2.9644581
H	-1.6553171	-1.0349127	4.1680961
H	-3.6065561	1.7626093	1.5532061
H	-2.2374101	3.2891003	-3.2379769
H	-3.4951181	0.5490453	3.7145681
N	0.1183779	-0.0808017	-0.0099329

RE1_R_Cz_R=H

Pd	-0.3025286	-0.4858468	-0.1392825
P	-1.4567386	1.5156592	-0.1100095
C	-2.8390446	1.7797412	-1.3242895
H	-3.0218956	2.8613042	-1.3819205
C	-2.3931716	1.2956042	-2.7123125
H	-2.1376506	0.2316552	-2.6412045
H	-1.4837306	1.8227482	-3.0211125
C	-3.4921346	1.4754322	-3.7609855
H	-3.6895106	2.5466652	-3.9050625
H	-3.1457596	1.0852782	-4.7238775
C	-4.7809606	0.7752482	-3.3271205
H	-4.6003046	-0.3061578	-3.2602085
H	-5.5669766	0.9226142	-4.0749235
C	-5.2410496	1.2890692	-1.9622095
H	-5.4965896	2.3549942	-2.0444535
H	-6.1500066	0.7686222	-1.6438815
C	-4.1562096	1.1053582	-0.8960735
H	-3.9918566	0.0360932	-0.7344745
H	-4.5108586	1.5198712	0.0524665
C	-2.2261396	1.8331682	1.5452065

H	-2.9962526	1.0517522	1.6023535
C	-1.2243766	1.5704362	2.6741235
H	-0.7472346	0.5964372	2.5255335
H	-0.4264686	2.3227572	2.6309095
C	-1.9067556	1.6284642	4.0418855
H	-2.6319776	0.8062432	4.1140825
H	-1.1661056	1.4673012	4.8322955
C	-2.6290236	2.9626112	4.2472515
H	-1.8852676	3.7698822	4.2924805
H	-3.1569896	2.9655972	5.2063635
C	-3.6064276	3.2484522	3.1034905
H	-4.0760046	4.2282022	3.2404925
H	-4.4146846	2.5047812	3.1187785
C	-2.9024586	3.1959402	1.7423905
H	-2.1411966	3.9851192	1.7042665
H	-3.6209536	3.4027372	0.9409975
C	-0.2933526	2.9013192	-0.4131515
C	-0.7615796	4.2080322	-0.6074645
C	0.1110804	5.2621852	-0.8395875
H	-0.2758466	6.2653642	-0.9851865
C	1.4793584	5.0174272	-0.8909105
H	2.1754054	5.8283502	-1.0799985
C	1.9541874	3.7251912	-0.7137085
C	1.0898584	2.6502292	-0.4703425
C	1.7237164	1.2886232	-0.3576015
C	2.3899164	0.9078562	0.8337825
C	2.3985024	1.8039932	2.0671655
H	1.5990474	2.5431752	1.9540465
C	2.1672054	1.0637092	3.3887185
H	1.2196834	0.5255752	3.3942135
H	2.1543844	1.7813842	4.2154215
H	2.9587274	0.3373862	3.5953765
C	3.7291144	2.5721002	2.1390775
H	4.5665024	1.8757212	2.2522115
H	3.7333704	3.2516112	2.9973895
H	3.9056954	3.1640962	1.2379635
C	3.1690484	-0.2469668	0.8295445
H	3.6529134	-0.5477248	1.7512765
C	3.3437764	-1.0255658	-0.3117145
C	4.1773374	-2.2951208	-0.3170475
C	5.4044644	-2.1327518	-1.2278295
H	5.9679164	-3.0695308	-1.2781105
H	6.0712954	-1.3549198	-0.8388365
H	5.1234184	-1.8556878	-2.2479035
C	2.7314324	-0.6032958	-1.4908005
H	2.8680594	-1.1960338	-2.3909135
C	1.9287274	0.5346732	-1.5462355
C	1.4566644	1.0614992	-2.8974965

H	0.7267754	1.8549222	-2.7146825
C	2.6444624	1.6977062	-3.6356285
H	3.1028764	2.4912902	-3.0388265
H	2.3191404	2.1311432	-4.5867865
H	3.4137294	0.9476402	-3.8468595
C	0.7723854	0.0166172	-3.7789155
H	1.4443384	-0.8056728	-4.0424015
H	0.4316524	0.4800802	-4.7105275
H	-0.0994936	-0.4050138	-3.2731325
H	3.0193754	3.5278082	-0.7810515
H	-1.8270736	4.4099442	-0.5795965
H	3.5377224	-3.0766368	-0.7471435
C	4.6067074	-2.7648038	1.0735395
H	5.3353714	-2.0767558	1.5196595
H	3.7558514	-2.8652008	1.7523585
H	5.0858994	-3.7449968	0.9977955
C	-2.6356426	-1.5908458	1.3212025
C	-1.9913276	-1.4919808	0.0877385
C	-4.4229026	-2.8280318	0.3140905
C	-3.8582706	-2.2514418	1.4415035
H	-2.1912676	-1.1563488	2.2116175
H	-4.3550156	-2.3151468	2.4031675
C	-3.7803126	-2.7933378	-0.9154815
H	-4.2167626	-3.2763458	-1.7824825
C	-2.5604266	-2.1277098	-1.0186825
H	-2.0560566	-2.1169278	-1.9796245
Br	-6.1419536	-3.7162318	0.4625815
C	0.8023774	-3.3023518	-0.8607005
C	0.9180904	-2.8671438	1.2939555
C	0.6167094	-3.2484478	-2.2477045
C	1.3041774	-4.4877858	-0.2573975
C	0.8683494	-2.2691878	2.5586435
C	1.3788414	-4.2020998	1.1538245
C	0.9457384	-4.3594308	-3.0095175
H	0.2287814	-2.3502638	-2.7112505
C	1.6347834	-5.5933858	-1.0431775
C	1.3003394	-2.9952648	3.6573135
H	0.5052844	-1.2525958	2.6616555
C	1.8086454	-4.9183718	2.2726145
C	1.4562524	-5.5262728	-2.4179005
H	0.8052314	-4.3258688	-4.0864935
H	2.0275024	-6.4957048	-0.5820495
C	1.7733674	-4.3116998	3.5206165
H	1.2734354	-2.5374138	4.6424155
H	2.1723094	-5.9368358	2.1660315
H	1.7076674	-6.3790428	-3.0405615
H	2.1078884	-4.8556478	4.3982755
N	0.5997094	-2.3146458	0.0746605

RE1_TS_Cz_R=H

Pd	0.3156762	0.4837580	-0.0699926
P	1.4509902	-1.5168440	0.0087264
C	3.0050262	-1.6707660	-0.9955926
H	3.3930292	-2.6958290	-0.9168816
C	2.7042442	-1.3765720	-2.4720986
H	2.2153002	-0.3968030	-2.5363036
H	1.9976312	-2.1161680	-2.8657996
C	3.9749072	-1.3536130	-3.3245346
H	4.4270292	-2.3551260	-3.3366516
H	3.7159072	-1.1117450	-4.3612346
C	4.9865492	-0.3474010	-2.7732836
H	4.5585642	0.6628250	-2.8280566
H	5.8958922	-0.3434990	-3.3834476
C	5.3230742	-0.6635160	-1.3158726
H	5.8314722	-1.6368080	-1.2647236
H	6.0167412	0.0823750	-0.9142786
C	4.0638582	-0.6969860	-0.4462676
H	3.6343762	0.3090610	-0.4044686
H	4.3391282	-0.9648390	0.5791064
C	2.0101752	-1.9120630	1.7392014
H	2.5914512	-1.0137360	1.9969514
C	0.8169842	-1.9707240	2.6983664
H	0.1677542	-1.1050010	2.5381554
H	0.2187462	-2.8626310	2.4683834
C	1.2791712	-2.0388710	4.1549504
H	1.8068042	-1.1087160	4.4080404
H	0.4103192	-2.1007780	4.8193434
C	2.2123532	-3.2316400	4.3815194
H	1.6435042	-4.1611380	4.2417464
H	2.5801532	-3.2387580	5.4130124
C	3.3874052	-3.2137260	3.3996704
H	4.0132292	-4.1015440	3.5404404
H	4.0233942	-2.3427530	3.6090094
C	2.9062622	-3.1399830	1.9449404
H	2.3342652	-4.0473850	1.7119654
H	3.7698452	-3.1198970	1.2717484
C	0.3901252	-2.9219250	-0.5246336
C	0.9308442	-4.2029980	-0.6947806
C	0.1523012	-5.2735860	-1.1156596
H	0.5980552	-6.2554950	-1.2380296
C	-1.1961708	-5.0707530	-1.3861996
H	-1.8190018	-5.8933670	-1.7231486
C	-1.7441278	-3.8029610	-1.2376476
C	-0.9778038	-2.7126720	-0.8070136
C	-1.6882168	-1.3844930	-0.7387826
C	-2.4849368	-1.0487550	0.3746794

C	-2.5771768	-1.9540770	1.5929454
H	-1.6968708	-2.6022490	1.5894744
C	-2.5887928	-1.1794910	2.9177684
H	-1.8445078	-0.3798040	2.9300484
H	-2.3860898	-1.8592650	3.7516994
H	-3.5643778	-0.7186180	3.1050294
C	-3.8158408	-2.8588020	1.5069694
H	-4.7276348	-2.2543420	1.4515344
H	-3.8854078	-3.4987590	2.3928344
H	-3.7831498	-3.5039600	0.6256284
C	-3.2502488	0.1203780	0.3403074
H	-3.8424118	0.3759650	1.2119024
C	-3.2698608	0.9611850	-0.7660586
C	-4.1160848	2.2204530	-0.8368806
C	-5.3115758	2.0027020	-1.7776246
H	-5.8940498	2.9240120	-1.8801886
H	-5.9728958	1.2228070	-1.3837236
H	-4.9861548	1.6936850	-2.7754506
C	-2.4912168	0.6026070	-1.8663876
H	-2.4976038	1.2529650	-2.7368856
C	-1.7004868	-0.5444590	-1.8803626
C	-0.9509318	-0.9254930	-3.1492166
H	-0.1926748	-1.6649440	-2.8796936
C	-1.9088728	-1.5900620	-4.1479826
H	-2.3740368	-2.4799860	-3.7146286
H	-1.3742808	-1.8922470	-5.0544636
H	-2.7069538	-0.8973420	-4.4362736
C	-0.2170178	0.2507780	-3.7984186
H	-0.9096508	0.9935470	-4.2082166
H	0.4093612	-0.1046060	-4.6228036
H	0.4300072	0.7475370	-3.0689986
H	-2.7913538	-3.6372040	-1.4725736
H	1.9869022	-4.3665850	-0.5051156
H	-3.4830248	2.9999460	-1.2780506
C	-4.5913068	2.7280330	0.5246004
H	-5.3412118	2.0593040	0.9633464
H	-3.7625888	2.8275790	1.2306754
H	-5.0563408	3.7118600	0.4107754
C	2.3514802	2.1840060	1.3666454
C	1.5631442	2.0983760	0.1960274
C	4.2403422	2.9680560	0.1020764
C	3.6734692	2.6025090	1.3160274
H	1.9455312	1.8934300	2.3254434
H	4.2598372	2.6287480	2.2280144
C	3.4855652	2.9394270	-1.0603286
H	3.9217632	3.2265290	-2.0109266
C	2.1609832	2.5224800	-1.0131336
H	1.6102582	2.4874600	-1.9419106

Br	6.1066222	3.4925820	0.0272024
C	-0.7439948	3.4426460	-0.4670106
C	-0.6230898	2.7698740	1.6703894
C	-0.7419088	3.5164640	-1.8594746
C	-1.4522188	4.3952770	0.2947624
C	-0.4568078	2.0340440	2.8441894
C	-1.3712878	3.9619190	1.6740294
C	-1.4155818	4.5691540	-2.4678016
H	-0.2872998	2.7419460	-2.4622226
C	-2.1287958	5.4414120	-0.3284576
C	-1.0175698	2.5239360	4.0168034
H	0.0646222	1.0826720	2.8326924
C	-1.9338688	4.4392580	2.8569554
C	-2.0978858	5.5318790	-1.7140856
H	-1.4266818	4.6320990	-3.5516416
H	-2.6843498	6.1639940	0.2619094
C	-1.7461478	3.7200180	4.0298614
H	-0.9009858	1.9572030	4.9354714
H	-2.5229858	5.3515510	2.8553024
H	-2.6212328	6.3380920	-2.2175216
H	-2.1799208	4.0752340	4.9588474
N	-0.1768738	2.4695270	0.3717534

RE2_P_Cz_R=H

C	-0.6939744	0.0125021	-1.2066512
C	-1.3945104	0.0001411	-0.0000372
C	1.3945186	0.0000361	-0.0000342
C	0.6939846	-0.0116259	-1.2066632
H	-1.2430944	0.0320441	-2.1419062
H	1.2431666	-0.0306419	-2.1419022
C	0.6939986	0.0117281	1.2065628
H	1.2430866	0.0307201	2.1418418
C	-0.6939964	-0.0122859	1.2065408
H	-1.2430674	-0.0318999	2.1418228
C	-3.6217564	-0.9220339	-0.6544072
C	-3.6219024	0.9221201	0.6543128
C	-3.2613504	-2.0382999	-1.4086022
C	-4.9714724	-0.5916299	-0.4179172
C	-3.2616724	2.0385051	1.4084518
C	-4.9715444	0.5914211	0.4179948
C	-4.2843374	-2.8117199	-1.9391902
H	-2.2205904	-2.2965239	-1.5701572
C	-5.9814694	-1.3856419	-0.9619032
C	-4.2847704	2.8117411	1.9390618
H	-2.2209454	2.2969471	1.5698928
C	-5.9816874	1.3852411	0.9620248
C	-5.6320374	-2.4913589	-1.7233972
H	-4.0322494	-3.6864729	-2.5302572

H	-7.0250834	-1.1436249	-0.7859642
C	-5.6324364	2.4910681	1.7234168
H	-4.0328334	3.6866001	2.5300348
H	-7.0252504	1.1428741	0.7862698
H	-6.4061894	-3.1186789	-2.1523942
H	-6.4066674	3.1182591	2.1524598
N	-2.8076104	0.0001661	-0.0001222
C	3.6218156	0.9220751	-0.6543522
C	3.6218446	-0.9221889	0.6542578
C	3.2614606	2.0385081	-1.4083002
C	4.9715326	0.5915691	-0.4178942
C	3.2615466	-2.0385909	1.4083048
C	4.9715366	-0.5915169	0.4179628
C	4.2844906	2.8119431	-1.9388102
H	2.2206956	2.2968671	-1.5696232
C	5.9815436	1.3855961	-0.9617772
C	4.2846006	-2.8119409	1.9388628
H	2.2208106	-2.2969629	1.5697608
C	5.9816046	-1.3854679	0.9619118
C	5.6321596	2.4914481	-1.7231182
H	4.0324436	3.6868401	-2.5296842
H	7.0251506	1.1435061	-0.7858932
C	5.6322696	-2.4913449	1.7232158
H	4.0326166	-3.6868109	2.5297998
H	7.0252016	-1.1432139	0.7861948
H	6.4063446	3.1187871	-2.1520282
H	6.4064726	-3.1186179	2.1521918
N	2.8076046	-0.0001589	-0.0001642

RE2_R_Cz_R=H

Pd	0.7999838	-0.1931171	-0.1058539
P	0.4321218	2.0647179	0.2470621
C	-0.7490312	3.0174749	-0.8271799
H	-0.5456232	4.0824129	-0.6489279
C	-0.4704042	2.7248039	-2.3081759
H	-0.6362302	1.6558139	-2.4882269
H	0.5781848	2.9328399	-2.5451069
C	-1.3872222	3.5412689	-3.2216059
H	-1.1614642	4.6097449	-3.1012189
H	-1.1837432	3.2908039	-4.2680859
C	-2.8591692	3.2944729	-2.8857079
H	-3.0987072	2.2422269	-3.0938049
H	-3.5058982	3.9016689	-3.5277369
C	-3.1383412	3.5932949	-1.4111179
H	-2.9730302	4.6632249	-1.2220439
H	-4.1862762	3.3873379	-1.1695109
C	-2.2318502	2.7665919	-0.4932819
H	-2.4640432	1.7047659	-0.6191719

H	-2.4457332	3.0165449	0.5502291
C	-0.1846272	2.3764379	1.9675561
H	-1.1736742	1.8995619	1.9469461
C	0.6576148	1.6359679	3.0124041
H	0.7987258	0.5930589	2.7125801
H	1.6563618	2.0890139	3.0572791
C	0.0050958	1.7124149	4.3944541
H	-0.9534662	1.1761169	4.3671771
H	0.6337758	1.1987679	5.1292091
C	-0.2374282	3.1642679	4.8161311
H	0.7313278	3.6628119	4.9568931
H	-0.7534362	3.1992419	5.7810381
C	-1.0408262	3.9244299	3.7573371
H	-1.1615192	4.9728549	4.0492821
H	-2.0501442	3.4964809	3.6878831
C	-0.3689272	3.8421329	2.3816801
H	0.6132348	4.3278889	2.4353011
H	-0.9628662	4.3936849	1.6443331
C	2.0224448	2.9629399	0.0893821
C	2.0757988	4.3623649	0.1516121
C	3.2770788	5.0464719	0.0282891
H	3.2931428	6.1300619	0.0832661
C	4.4529108	4.3312199	-0.1733999
H	5.4000328	4.8497759	-0.2822359
C	4.4104148	2.9459589	-0.2514029
C	3.2093338	2.2372849	-0.1188629
C	3.2995268	0.7423019	-0.2894059
C	3.7759888	-0.0703681	0.7689051
C	4.0380668	0.5052809	2.1537101
H	3.5050938	1.4580319	2.2256211
C	3.5555828	-0.3939661	3.2961451
H	2.5226948	-0.7111291	3.1561501
H	3.6328078	0.1399639	4.2488301
H	4.1621388	-1.3010611	3.3800611
C	5.5365418	0.8005299	2.3302751
H	6.1195238	-0.1216401	2.2370011
H	5.7267298	1.2251869	3.3212511
H	5.9028958	1.5090559	1.5838041
C	4.1218278	-1.3956261	0.5027831
H	4.4556618	-2.0191121	1.3244461
C	4.0545638	-1.9366701	-0.7772439
C	4.4529938	-3.3664951	-1.0967259
C	5.7764638	-3.3841411	-1.8784029
H	6.0378938	-4.4087521	-2.1608499
H	6.5907758	-2.9814021	-1.2655489
H	5.7158018	-2.7862691	-2.7928169
C	3.6264138	-1.1102241	-1.8158149
H	3.5767228	-1.5240361	-2.8190289

C	3.2448888	0.2105999	-1.6074239
C	2.9350488	1.1015079	-2.8035789
H	2.5335978	2.0448619	-2.4243869
C	4.2322838	1.4314829	-3.5566489
H	4.9631648	1.9064559	-2.8961349
H	4.0305548	2.1140619	-4.3884439
H	4.6860608	0.5219079	-3.9637339
C	1.8918898	0.5150479	-3.7568919
H	2.2483168	-0.4008801	-4.2385289
H	1.6582188	1.2367769	-4.5462609
H	0.9648418	0.2829489	-3.2255449
H	5.3231248	2.3883209	-0.4371359
H	1.1632258	4.9307709	0.2953471
H	3.6691788	-3.7649681	-1.7531209
C	4.5388908	-4.2774031	0.1272941
H	5.3838828	-4.0061151	0.7715741
H	3.6210368	-4.2442481	0.7196131
H	4.6922588	-5.3117391	-0.1936969
C	-1.8869512	-0.5282791	1.1198721
C	-1.1460362	-0.5323191	-0.0622539
C	-3.2723362	-0.6636481	1.0902391
H	-1.3922612	-0.4009291	2.0778541
H	-3.8499542	-0.6166281	2.0084221
C	-3.1874932	-0.9327241	-1.3037619
H	-3.6979442	-1.1214281	-2.2430059
C	-1.8064972	-0.7686881	-1.2707559
H	-1.2463012	-0.8383871	-2.1981279
C	0.9986998	-3.1122141	-1.1835469
C	0.9227228	-3.0099791	1.0144641
C	1.0002678	-2.8303221	-2.5554799
C	1.0581228	-4.4597421	-0.7363009
C	0.8081868	-2.6003341	2.3490441
C	1.0004108	-4.3928951	0.7024251
C	1.1104538	-3.8812401	-3.4529439
H	0.9277758	-1.8072511	-2.9023989
C	1.1767268	-5.5033931	-1.6560639
C	0.8191118	-3.5613991	3.3474641
H	0.6947158	-1.5477491	2.5866801
C	1.0194348	-5.3461921	1.7226521
C	1.2103568	-5.2120231	-3.0125079
H	1.1171518	-3.6718381	-4.5192779
H	1.2420358	-6.5320911	-1.3115399
C	0.9354878	-4.9278821	3.0432761
H	0.7311778	-3.2519181	4.3853221
H	1.0960938	-6.4037431	1.4845081
H	1.3005698	-6.0128601	-3.7395559
H	0.9464908	-5.6586601	3.8456621
N	0.9713018	-2.2364271	-0.1235999

C	-3.9281202	-0.8490431	-0.1250009
C	-6.1647312	-0.0568981	-0.8719369
C	-6.1442052	-1.7867421	0.5753951
C	-5.8112392	0.9921859	-1.7203049
C	-7.5126722	-0.3598561	-0.5880709
C	-5.7671402	-2.8314571	1.4198001
C	-7.4992952	-1.4729141	0.3391931
C	-6.8361462	1.7343309	-2.2903319
H	-4.7704382	1.2138899	-1.9268719
C	-8.5258572	0.3985159	-1.1772689
C	-6.7796882	-3.5516811	2.0379261
H	-4.7220442	-3.0731861	1.5811681
C	-8.4985142	-2.2150061	0.9714191
C	-8.1825962	1.4423259	-2.0258959
H	-6.5859032	2.5541169	-2.9572769
H	-9.5686432	0.1773889	-0.9699479
C	-8.1325812	-3.2493721	1.8207311
H	-6.5157272	-4.3692991	2.7014411
H	-9.5459312	-1.9870571	0.7975701
H	-8.9611792	2.0387259	-2.4901909
H	-8.8979342	-3.8340491	2.3206611
N	-5.3420102	-0.9286951	-0.1690739

RE2_TS_Cz_R=H

Pd	-0.8305301	0.3166774	-0.3635148
P	-0.2157241	-1.9203046	-0.3348398
C	0.9533059	-2.5765616	-1.6206438
H	1.1013589	-3.6517626	-1.4506288
C	0.3723929	-2.3903286	-3.0284578
H	0.1555399	-1.3255946	-3.1783978
H	-0.5782071	-2.9280606	-3.1210488
C	1.3522859	-2.8584776	-4.1077908
H	1.5085629	-3.9417096	-4.0108258
H	0.9171899	-2.6896706	-5.0988448
C	2.6984569	-2.1408846	-3.9812308
H	2.5517269	-1.0672396	-4.1635198
H	3.3964789	-2.4998396	-4.7449828
C	3.2895339	-2.3354106	-2.5840608
H	3.5232489	-3.3988836	-2.4356518
H	4.2324279	-1.7889076	-2.4789788
C	2.3161329	-1.8770996	-1.4963038
H	2.1714919	-0.7929996	-1.5654478
H	2.7631459	-2.0686866	-0.5175238
C	0.6723279	-2.2597846	1.2606992
H	1.4792149	-1.5128196	1.2217692
C	-0.2078501	-1.8891466	2.4581232
H	-0.6605541	-0.9068206	2.2871622
H	-1.0345661	-2.6079486	2.5380472

C	0.6016819	-1.8962616	3.7557232
H	1.3694629	-1.1119716	3.6996542
H	-0.0465041	-1.6478276	4.6034212
C	1.2762619	-3.2533376	3.9760472
H	0.5017699	-4.0156846	4.1380502
H	1.8923969	-3.2310566	4.8810902
C	2.1259719	-3.6542876	2.7665162
H	2.5643039	-4.6462546	2.9208962
H	2.9642059	-2.9506966	2.6668192
C	1.3048139	-3.6399886	1.4702532
H	0.5138409	-4.3985056	1.5362552
H	1.9465719	-3.9142796	0.6258322
C	-1.6462321	-3.0791526	-0.4095118
C	-1.4365671	-4.4585726	-0.5372738
C	-2.4939991	-5.3484826	-0.6774038
H	-2.3006831	-6.4118376	-0.7755988
C	-3.7957431	-4.8615246	-0.7085698
H	-4.6331021	-5.5405406	-0.8350038
C	-4.0216081	-3.4969346	-0.5825668
C	-2.9700071	-2.5877586	-0.4180648
C	-3.3362431	-1.1356646	-0.2809668
C	-3.7639161	-0.6262256	0.9612672
C	-3.8451801	-1.5093986	2.2001522
H	-3.1817521	-2.3654016	2.0419132
C	-3.3970751	-0.8095576	3.4891482
H	-2.4276741	-0.3221076	3.3717642
H	-3.3152161	-1.5421046	4.2984152
H	-4.1159231	-0.0490796	3.8105032
C	-5.2707651	-2.0540136	2.3818252
H	-5.9821941	-1.2298676	2.5022802
H	-5.3298551	-2.6877916	3.2728612
H	-5.5876331	-2.6491656	1.5223762
C	-4.1730641	0.7071394	1.0429662
H	-4.4856831	1.0954264	2.0054962
C	-4.1684541	1.5549044	-0.0597568
C	-4.5825461	3.0156484	0.0126272
C	-5.8490311	3.2649194	-0.8213168
H	-6.1066311	4.3288594	-0.8155938
H	-6.6961591	2.7067494	-0.4075378
H	-5.7191491	2.9563064	-1.8623988
C	-3.7621771	1.0245884	-1.2844128
H	-3.7486331	1.6766794	-2.1536848
C	-3.3574121	-0.3031616	-1.4259798
C	-3.0754581	-0.8658466	-2.8128978
H	-2.5116821	-1.7941656	-2.6879718
C	-4.4001531	-1.2194776	-3.5039208
H	-4.9807251	-1.9269536	-2.9048148
H	-4.2182881	-1.6711956	-4.4844958

H	-5.0091421	-0.3205376	-3.6480728
C	-2.2306941	0.0571924	-3.6935848
H	-2.7564571	0.9829584	-3.9486948
H	-1.9757191	-0.4475506	-4.6308348
H	-1.3005381	0.3227414	-3.1815868
H	-5.0359681	-3.1113036	-0.6204608
H	-0.4238281	-4.8490046	-0.5381788
H	-3.7654811	3.5911644	-0.4423298
C	-4.7782351	3.5364414	1.4361382
H	-5.6545591	3.0769944	1.9090442
H	-3.9049511	3.3461444	2.0646602
H	-4.9444371	4.6173804	1.4160642
C	1.4566969	1.2266674	1.2534192
C	0.8161789	1.4774754	0.0183252
C	3.6353299	1.3283524	0.2087512
C	2.8374119	1.1479074	1.3369942
H	0.8779369	1.0369634	2.1459212
H	3.3051019	0.9154994	2.2893072
C	3.0224279	1.6659334	-0.9970558
H	3.6322939	1.8183084	-1.8827828
C	1.6428849	1.7695064	-1.0910208
H	1.2077749	2.0094054	-2.0510718
C	-0.9267531	3.3961834	-0.8671898
C	-1.0681941	2.9352024	1.3193772
C	-0.9090631	3.3124354	-2.2581798
C	-1.2394601	4.6121184	-0.2256648
C	-1.2124691	2.3021834	2.5522212
C	-1.3204581	4.3154314	1.1907042
C	-1.1635541	4.4650514	-2.9925048
H	-0.7466411	2.3635684	-2.7553588
C	-1.4969441	5.7585264	-0.9742678
C	-1.5501771	3.0733454	3.6577912
H	-1.1084221	1.2272244	2.6360652
C	-1.6560791	5.0784934	2.3070382
C	-1.4459381	5.6818074	-2.3608128
H	-1.1572751	4.4132154	-4.0769728
H	-1.7487691	6.6924024	-0.4804748
C	-1.7564711	4.4539964	3.5438892
H	-1.6726781	2.5902734	4.6223212
H	-1.8541101	6.1413714	2.2056982
H	-1.6473401	6.5645394	-2.9589288
H	-2.0226961	5.0315574	4.4230682
N	-0.7407711	2.3728664	0.0751942
C	5.6283719	-0.0980836	0.6219632
C	6.0317029	1.9971564	-0.0989218
C	5.0296899	-1.2851726	1.0473082
C	7.0269719	0.0115584	0.4693612
C	5.9140059	3.3243644	-0.5130938

C	7.2863039	1.3595074	0.0075472
C	5.8566529	-2.3670106	1.3143682
H	3.9544699	-1.3531786	1.1720522
C	7.8381759	-1.0894486	0.7502812
C	7.0828799	4.0030084	-0.8272648
H	4.9436419	3.8043794	-0.5811598
C	8.4472629	2.0654894	-0.3128488
C	7.2492849	-2.2742186	1.1702172
H	5.4129999	-3.3012216	1.6465382
H	8.9158679	-1.0182136	0.6366042
C	8.3388649	3.3841254	-0.7309638
H	7.0220869	5.0373544	-1.1515628
H	9.4196289	1.5885944	-0.2325988
H	7.8689929	-3.1370906	1.3915522
H	9.2326629	3.9450574	-0.9843408
N	5.0341649	1.1097824	0.2814762

RE1_P_Ac_R=F

C	2.5212277	1.4780902	1.0730075
C	1.9860037	0.5801262	0.1550065
C	4.7148377	1.1686612	0.1195645
C	3.8772297	1.7685942	1.0531145
H	1.8771177	1.9512202	1.8046635
C	4.1965847	0.2693262	-0.8043935
H	4.8309417	-0.2090918	-1.5398525
C	2.8404257	-0.0128178	-0.7745065
Br	6.5857747	1.5830632	0.1072305
F	4.3669737	2.6320692	1.9424275
F	2.3363367	-0.8743918	-1.6577585
C	-0.2477403	0.9753212	-0.7096575
C	-0.5761763	2.8051142	-2.2675815
C	-1.5891493	0.5945022	-0.8572925
C	-1.9084573	2.4411052	-2.4242245
H	-0.1693273	3.6558582	-2.8049525
C	0.1700557	-0.8720298	0.8531445
C	-2.3906563	1.3478792	-1.7186115
H	-2.5632663	2.9984922	-3.0852095
C	1.0711257	-1.5701888	1.6713615
C	-1.1592143	-1.3065568	0.7509295
H	-3.4322753	1.0672552	-1.8437025
C	0.6664547	-2.6895118	2.3807195
H	2.0967637	-1.2321788	1.7565035
C	-1.5348993	-2.4360218	1.4823785
C	-0.6479383	-3.1325748	2.2908715
H	1.3839057	-3.2122248	3.0052935
H	-2.5609643	-2.7854248	1.4154525
H	-0.9789833	-4.0067708	2.8409185
C	0.2452457	2.0816662	-1.4180485

H	1.2796117	2.3811012	-1.3003515
N	0.6043687	0.2666632	0.1539715
C	-2.1984953	-0.5953548	-0.1157205
C	-3.3419113	-0.0870298	0.7908215
H	-2.9596293	0.6235462	1.5287445
H	-4.1161343	0.4144662	0.2027025
H	-3.8157113	-0.9146328	1.3268995
C	-2.7587463	-1.5972198	-1.1502335
H	-3.2214813	-2.4562938	-0.6564775
H	-3.5214833	-1.1294398	-1.7789965
H	-1.9583453	-1.9643668	-1.7981545

RE1_R_Ac_R=F

Pd	0.1959524	0.0639997	0.3098071
P	-0.8244666	2.1367277	0.1054801
C	-0.4330366	3.4914197	1.3146311
H	-1.1942126	4.2722397	1.1849581
C	-0.5468486	2.9471477	2.7466271
H	0.1671564	2.1255447	2.8640321
H	-1.5483266	2.5350707	2.9125171
C	-0.2408676	4.0243687	3.7887141
H	-1.0050636	4.8124077	3.7410811
H	-0.2979496	3.5871537	4.7910751
C	1.1394244	4.6373047	3.5471341
H	1.9039814	3.8593147	3.6742871
H	1.3483084	5.4164747	4.2875161
C	1.2361704	5.2088547	2.1315071
H	0.5183004	6.0343947	2.0257461
H	2.2309434	5.6304037	1.9543271
C	0.9401254	4.1428187	1.0711471
H	1.7245174	3.3812607	1.0967901
H	0.9799574	4.6003987	0.0777351
C	-0.4105576	2.8539437	-1.5532729
H	0.6752024	3.0080367	-1.4775169
C	-0.6495916	1.8285737	-2.6670329
H	-0.1650786	0.8819247	-2.4146039
H	-1.7273906	1.6308447	-2.7466949
C	-0.1201016	2.3374107	-4.0088729
H	0.9725944	2.4299897	-3.9462719
H	-0.3288316	1.5997357	-4.7912079
C	-0.7273896	3.6942407	-4.3726429
H	-1.8046646	3.5699677	-4.5490919
H	-0.2946256	4.0684647	-5.3061499
C	-0.5203086	4.7132657	-3.2483709
H	-1.0027176	5.6634127	-3.5006679
H	0.5523574	4.9208937	-3.1363939
C	-1.0705316	4.1912287	-1.9154229
H	-2.1534726	4.0433607	-2.0127369

H	-0.9169076	4.9387967	-1.1286309
C	-2.6575926	2.0038767	0.1674651
C	-3.4516026	3.1592467	0.1974711
C	-4.8358816	3.0877897	0.2691801
H	-5.4251786	3.9987077	0.2894761
C	-5.4534146	1.8431097	0.3251281
H	-6.5336856	1.7671097	0.3960801
C	-4.6791386	0.6916067	0.3036251
C	-3.2820356	0.7440567	0.2176731
C	-2.5523986	-0.5703863	0.2458351
C	-2.5152566	-1.3924813	-0.9059439
C	-3.1902446	-0.9676503	-2.2079519
H	-3.3951306	0.1060287	-2.1476029
C	-2.3683756	-1.2039793	-3.4802909
H	-1.4570936	-0.6055083	-3.4954129
H	-2.9631766	-0.9232783	-4.3554209
H	-2.0827426	-2.2532233	-3.6005129
C	-4.5414416	-1.6926393	-2.3337869
H	-4.3870676	-2.7720333	-2.4349719
H	-5.0845866	-1.3424073	-3.2172649
H	-5.1737206	-1.5279473	-1.4580499
C	-1.9598826	-2.6680133	-0.8008379
H	-1.8842846	-3.2774433	-1.6952639
C	-1.4829676	-3.1730813	0.4054521
C	-0.8286326	-4.5357783	0.5381001
C	-1.8383876	-5.5614173	1.0752131
H	-1.3550346	-6.5291503	1.2424731
H	-2.6528926	-5.7076703	0.3567211
H	-2.2797276	-5.2322413	2.0209351
C	-1.6529366	-2.3978353	1.5502371
H	-1.3500786	-2.8155233	2.5061081
C	-2.1573276	-1.1007803	1.5032691
C	-2.4559226	-0.3537513	2.7992731
H	-2.6489396	0.6922467	2.5472531
C	-3.7431916	-0.9193043	3.4188381
H	-4.5839386	-0.8471403	2.7232131
H	-4.0051606	-0.3721353	4.3300521
H	-3.6112156	-1.9746423	3.6797771
C	-1.3170456	-0.3669053	3.8211741
H	-1.1105696	-1.3762343	4.1925981
H	-1.5908096	0.2476247	4.6850401
H	-0.3939976	0.0375357	3.3997231
H	-5.1575036	-0.2802343	0.3698321
H	-2.9835416	4.1369327	0.1669121
H	-0.0333506	-4.4144943	1.2850461
C	-0.1718876	-5.0358873	-0.7491989
H	-0.9162626	-5.2824643	-1.5151589
H	0.5153784	-4.2924203	-1.1612099

H	0.3930364	-5.9503093	-0.5443199
C	2.6393314	1.1161237	-1.0071749
C	2.0074494	0.8632607	0.2077881
C	4.6315264	1.8722257	0.1233381
C	3.9316414	1.6240177	-1.0487109
H	2.1520604	0.8859897	-1.9500609
C	4.0316634	1.6110977	1.3498501
H	4.5548714	1.7863377	2.2821041
C	2.7366834	1.1190577	1.3641061
Br	6.4238744	2.5573467	0.0519011
F	4.4941814	1.8615137	-2.2373379
F	2.1677894	0.9179987	2.5752381
C	1.7251454	-1.9208113	-1.1930149
C	1.3051044	-1.6354893	-3.5861179
C	2.9986094	-2.4408773	-1.4903219
C	2.5858954	-2.0974623	-3.8774379
H	0.6263644	-1.3412983	-4.3825049
C	2.0146844	-2.2233273	1.1448871
C	3.3992984	-2.5045593	-2.8279779
H	2.9407644	-2.1567153	-4.9010289
C	1.5049584	-2.1184683	2.4502961
C	3.2695534	-2.8376133	0.9694821
H	4.3934654	-2.8779823	-3.0581639
C	2.1806714	-2.6139293	3.5498761
H	0.5631094	-1.6080683	2.5772971
C	3.9357914	-3.3332383	2.0944501
C	3.4130164	-3.2391263	3.3767441
H	1.7476974	-2.5035373	4.5403121
H	4.9100974	-3.7963533	1.9646731
H	3.9629574	-3.6348363	4.2243531
C	0.8886264	-1.5655143	-2.2683639
H	-0.1142216	-1.2408633	-2.0267079
N	1.2402274	-1.7273593	0.0965171
C	3.9464954	-2.9167293	-0.3954129
C	4.3593224	-4.3758943	-0.6884889
H	3.4792294	-5.0257033	-0.6932519
H	4.8519324	-4.4615103	-1.6611369
H	5.0586334	-4.7514263	0.0636661
C	5.1969654	-2.0091683	-0.3772189
H	5.9091204	-2.3409603	0.3849751
H	5.7065364	-2.0199623	-1.3460169
H	4.9136664	-0.9770633	-0.1509149

RE1_TS_Ac_R=F

Pd	0.1917614	-0.1040867	-0.4502044
P	-0.9937126	-2.0712977	-0.1592664
C	-0.8669486	-3.4294077	-1.4181194
H	-1.3759116	-4.3161747	-1.0169144

C	-1.5445966	-3.0553917	-2.7427654
H	-1.0741946	-2.1457487	-3.1352764
H	-2.6040626	-2.8290097	-2.5794614
C	-1.4053746	-4.1807037	-3.7727034
H	-1.9555826	-5.0634427	-3.4183774
H	-1.8719746	-3.8767667	-4.7159484
C	0.0611484	-4.5559487	-3.9984744
H	0.5851614	-3.7022057	-4.4490774
H	0.1360384	-5.3857627	-4.7092964
C	0.7446184	-4.9196917	-2.6784824
H	0.2926784	-5.8357857	-2.2732874
H	1.8050334	-5.1370717	-2.8438144
C	0.6083054	-3.7886167	-1.6561494
H	1.1266404	-2.9009867	-2.0325684
H	1.1043344	-4.0685257	-0.7203114
C	-0.3571126	-2.8607327	1.3984976
H	0.7243114	-2.9052837	1.2042486
C	-0.5528316	-1.9118987	2.5867256
H	-0.1929426	-0.9151777	2.3164846
H	-1.6261456	-1.8146767	2.8036716
C	0.1834844	-2.4220357	3.8267956
H	1.2630704	-2.4192997	3.6257626
H	0.0132124	-1.7409297	4.6680446
C	-0.2615756	-3.8415177	4.1867206
H	-1.3181416	-3.8227797	4.4882236
H	0.3068494	-4.2125867	5.0458626
C	-0.0965836	-4.7887967	2.9953506
H	-0.4579586	-5.7904757	3.2518826
H	0.9709404	-4.8876887	2.7569456
C	-0.8375856	-4.2716967	1.7551926
H	-1.9151076	-4.2514507	1.9644706
H	-0.6794106	-4.9646817	0.9217696
C	-2.8117156	-1.8708247	0.0810186
C	-3.6331126	-3.0041767	0.1532006
C	-5.0118266	-2.9008417	0.2863036
H	-5.6204706	-3.7976657	0.3404886
C	-5.6005796	-1.6425917	0.3368136
H	-6.6773346	-1.5421347	0.4290696
C	-4.8025016	-0.5089667	0.2588686
C	-3.4103116	-0.5943537	0.1404536
C	-2.6419626	0.6924653	0.0528566
C	-2.4214746	1.4753193	1.2005706
C	-2.9834886	1.0793103	2.5617456
H	-3.2858476	0.0287083	2.5103956
C	-1.9818086	1.2065273	3.7150746
H	-1.0885266	0.6024773	3.5449116
H	-2.4442686	0.8637523	4.6460716
H	-1.6669586	2.2435523	3.8695446

C	-4.2366746	1.9133883	2.8718796
H	-3.9814746	2.9763613	2.9415606
H	-4.6794036	1.6070603	3.8251686
H	-4.9961076	1.8050623	2.0933336
C	-1.7211086	2.6811603	1.0748936
H	-1.5282546	3.2651683	1.9684266
C	-1.2230526	3.1264523	-0.1429664
C	-0.3873436	4.3866023	-0.3050674
C	-1.1597366	5.4483743	-1.1034594
H	-0.5322056	6.3275313	-1.2801824
H	-2.0494376	5.7697303	-0.5505244
H	-1.4877196	5.0683543	-2.0752684
C	-1.5111016	2.3640933	-1.2776844
H	-1.1615496	2.7223223	-2.2411214
C	-2.2188556	1.1648153	-1.2150364
C	-2.6750496	0.4765723	-2.4967884
H	-2.8755566	-0.5718467	-2.2632364
C	-3.9997236	1.1047003	-2.9567064
H	-4.7640356	1.0295623	-2.1779994
H	-4.3756416	0.6005893	-3.8529034
H	-3.8598496	2.1651683	-3.1927324
C	-1.6500016	0.4915473	-3.6319634
H	-1.4364626	1.5053593	-3.9868074
H	-2.0357106	-0.0760097	-4.4847514
H	-0.7080436	0.0305793	-3.3206464
H	-5.2600346	0.4751833	0.2834566
H	-3.1897246	-3.9925267	0.0918086
H	0.4899804	4.0926523	-0.8981234
C	0.1241504	4.9774643	1.0087526
H	-0.6998756	5.3596683	1.6230106
H	0.6824764	4.2441173	1.5954896
H	0.7908904	5.8189593	0.7989946
C	2.5824694	-1.0455347	1.0569936
C	2.1606104	-0.5544717	-0.2006564
C	4.2294474	-2.5125717	0.0633806
C	3.5815114	-1.9955517	1.1739656
H	2.1262444	-0.6901177	1.9716636
C	3.8288574	-2.0690257	-1.1953094
H	4.2727054	-2.4710467	-2.0985894
C	2.8282494	-1.1303407	-1.3081934
Br	5.6197304	-3.8278227	0.2452446
F	3.8946654	-2.4260297	2.4046056
F	2.4093764	-0.8256997	-2.5619304
C	2.1285854	1.8159973	1.0008446
C	1.4951684	2.2018403	3.3096786
C	3.2947844	2.5777283	1.1832066
C	2.6311774	2.9817893	3.4911316
H	0.7932994	2.0481743	4.1229356

C	2.3358654	1.9347983	-1.3706144
C	3.5097264	3.1687473	2.4289726
H	2.8353474	3.4449503	4.4509556
C	1.6189204	1.9128853	-2.5671334
C	3.4943824	2.7221273	-1.2654154
H	4.3914854	3.7788013	2.5873776
C	2.0271624	2.6581503	-3.6619724
H	0.7322844	1.2956303	-2.6203904
C	3.8747304	3.4874453	-2.3693324
C	3.1580144	3.4609063	-3.5607434
H	1.4545624	2.6198083	-4.5836484
H	4.7541114	4.1180333	-2.3079104
H	3.4843744	4.0637983	-4.4020414
C	1.2425224	1.6422953	2.0653856
H	0.3330034	1.0813453	1.8832196
N	1.8508294	1.2115683	-0.2492194
C	4.2941414	2.6769663	0.0335286
C	5.2195764	3.8910393	0.1816106
H	4.6597834	4.8310883	0.1949136
H	5.8060644	3.8200433	1.1008656
H	5.9428924	3.9259733	-0.6366944
C	5.1752144	1.4030563	0.0331956
H	5.8853534	1.4411833	-0.7992154
H	5.7343774	1.3338173	0.9718716
H	4.5769894	0.4977903	-0.0728794

RE2_P_Ac_R=F

C	0.5737463	0.0000941	-1.3027029
C	1.3583283	0.0006051	-0.1538519
C	-1.4391887	0.0007021	0.0535811
C	-0.8075747	0.0001331	-1.1882719
H	1.0445233	-0.0004379	-2.2782779
C	-0.6509747	0.0012771	1.2053251
H	-1.1204837	0.0016801	2.1820081
C	0.7276243	0.0012371	1.0910371
F	-1.5504787	-0.0004549	-2.2986439
F	1.4802433	0.0017311	2.1929151
C	3.4521783	1.2274261	-0.1492799
C	3.3694883	3.6508901	-0.1252589
C	4.8493943	1.2632501	-0.0399299
C	4.7538703	3.7034711	-0.0163779
H	2.7835073	4.5636841	-0.1602119
C	3.4516133	-1.2270969	-0.1488359
C	5.4673113	2.5143311	0.0223891
H	5.2716623	4.6548051	0.0375681
C	2.7241483	-2.4259409	-0.1923249
C	4.8488153	-1.2635209	-0.0395569
H	6.5488843	2.5601481	0.1067721

C	3.3678073	-3.6505119	-0.1238649
H	1.6453723	-2.3989939	-0.2857059
C	5.4661673	-2.5148659	0.0231571
C	4.7521793	-3.7036919	-0.0151219
H	2.7813963	-4.5630479	-0.1583919
H	6.5477233	-2.5611489	0.1074941
H	5.2695383	-4.6552449	0.0391151
N	2.7738223	0.0003111	-0.2314539
C	5.7086753	-0.0003269	0.0056401
C	6.6611883	-0.0007099	-1.2107799
H	6.0916813	-0.0006209	-2.1440029
H	7.3067443	0.8823931	-1.2044509
H	7.3062213	-0.8841949	-1.2043069
C	6.5318523	-0.0003379	1.3130831
H	7.1755273	-0.8821449	1.3737441
H	7.1759503	0.8811771	1.3734941
H	5.8679033	-0.0000619	2.1814491
C	-3.5403987	1.2172361	0.2992581
C	-3.5741117	3.5526531	0.9126841
C	-4.9311937	1.2367061	0.0985041
C	-4.9550547	3.5739141	0.7706791
H	-3.0383707	4.4469921	1.2142341
C	-3.5397637	-1.2167759	0.2991081
C	-5.6169527	2.4195401	0.3641031
H	-5.5145897	4.4838011	0.9594001
C	-2.8658547	-2.3781119	0.6859751
C	-4.9305307	-1.2369999	0.0982721
H	-6.6923507	2.4550721	0.2330701
C	-3.5722077	-3.5522659	0.9123081
H	-1.7908787	-2.3651759	0.8213271
C	-5.6156417	-2.4202669	0.3636231
C	-4.9531167	-3.5743169	0.7700941
H	-3.0359937	-4.4463279	1.2138411
H	-6.6910047	-2.4563999	0.2324771
H	-5.5121367	-4.4845659	0.9585981
N	-2.8538427	0.0004301	0.1129531
C	-5.5858017	-0.0002659	-0.5113229
C	-5.2721317	-0.0000349	-2.0293139
H	-5.7006587	-0.8905159	-2.5000829
H	-5.7010777	0.8903391	-2.4999049
H	-4.1948847	0.0002351	-2.2106429
C	-7.1065187	-0.0006999	-0.3318189
H	-7.5516957	0.8734451	-0.8131399
H	-7.5512117	-0.8750089	-0.8132909
H	-7.3909807	-0.0008709	0.7246611
C	2.7252723	2.4265861	-0.1933299
H	1.6464963	2.4001101	-0.2868599
C	-2.8671077	2.3789331	0.6861161

H	-1.7920967	2.3666161	0.8212521

RE2_R_Ac_R=F

Pd	1.3893482	0.1357744	0.3270350
P	1.5612372	-2.1817506	0.1334690
C	0.6276282	-3.3256556	1.2650590
H	1.0228942	-4.3364676	1.1000470
C	0.9013322	-2.9458766	2.7284670
H	0.6180342	-1.9003306	2.8813650
H	1.9747332	-3.0258976	2.9342700
C	0.1150692	-3.8266016	3.7021130
H	0.4731812	-4.8632096	3.6324600
H	0.3049112	-3.4938456	4.7279230
C	-1.3818598	-3.7866596	3.3922340
H	-1.7501418	-2.7606966	3.5247710
H	-1.9365518	-4.4190656	4.0929400
C	-1.6458468	-4.2321336	1.9537860
H	-1.3449528	-5.2827346	1.8376110
H	-2.7165678	-4.1804636	1.7287110
C	-0.8792238	-3.3659646	0.9500980
H	-1.2880308	-2.3534116	0.9753980
H	-1.0486608	-3.7483626	-0.0618070
C	0.9896282	-2.6674626	-1.5644410
H	-0.0734768	-2.3870646	-1.5436170
C	1.6667532	-1.8111416	-2.6394340
H	1.5566872	-0.7526856	-2.3932070
H	2.7425292	-2.0362456	-2.6522640
C	1.0653402	-2.0732256	-4.0214750
H	0.0226942	-1.7280626	-4.0236440
H	1.5940682	-1.4749656	-4.7714810
C	1.1131722	-3.5583026	-4.3863980
H	2.1610722	-3.8665636	-4.5049270
H	0.6223232	-3.7298996	-5.3499370
C	0.4612062	-4.4161526	-3.2987720
H	0.5480742	-5.4789446	-3.5484200
H	-0.6121448	-4.1889776	-3.2461920
C	1.0969152	-4.1547606	-1.9280100
H	2.1560842	-4.4387616	-1.9686780
H	0.6222192	-4.7881526	-1.1704110
C	3.3061982	-2.7487876	0.2738650
C	3.6050892	-4.1181786	0.3141950
C	4.9091342	-4.5747886	0.4473790
H	5.1099672	-5.6407346	0.4749720
C	5.9465872	-3.6553276	0.5553170
H	6.9712412	-3.9934116	0.6722070
C	5.6645062	-2.2971146	0.5246130
C	4.3565762	-1.8176756	0.3776660
C	4.1792452	-0.3247116	0.3957520

C	4.5578272	0.4521524	-0.7243360
C	5.1327082	-0.1873456	-1.9860760
H	4.9534672	-1.2657986	-1.9324790
C	4.5232262	0.3099934	-3.3015710
H	3.4678082	0.0487534	-3.3838600
H	5.0456512	-0.1526726	-4.1449420
H	4.6097462	1.3948704	-3.4148360
C	6.6552882	0.0316664	-2.0122210
H	6.8859722	1.0982354	-2.1038170
H	7.1044042	-0.4867346	-2.8653070
H	7.1343022	-0.3339276	-1.1004370
C	4.5134692	1.8434694	-0.6219960
H	4.7549582	2.4363864	-1.4982190
C	4.1470612	2.4901734	0.5542580
C	4.0404052	3.9988734	0.6745180
C	5.3298992	4.5774174	1.2764210
H	5.2338462	5.6567454	1.4309990
H	6.1776882	4.4030964	0.6043130
H	5.5634852	4.1136394	2.2396930
C	3.9044902	1.7071184	1.6805270
H	3.6902062	2.2068604	2.6206200
C	3.8937802	0.3152084	1.6324720
C	3.7770442	-0.4891066	2.9228220
H	3.5424852	-1.5222176	2.6551680
C	5.1404222	-0.4990836	3.6313290
H	5.9217112	-0.9080006	2.9843900
H	5.0974082	-1.1065756	4.5409980
H	5.4328532	0.5180664	3.9132340
C	2.6786982	-0.0129786	3.8753700
H	2.8825172	0.9869604	4.2731540
H	2.6138832	-0.6944756	4.7297860
H	1.7028242	0.0071084	3.3860190
H	6.4694722	-1.5762236	0.6257380
H	2.8064592	-4.8483816	0.2438780
H	3.2193982	4.1872114	1.3781510
C	3.6838852	4.6993004	-0.6375800
H	4.5052922	4.6499324	-1.3617750
H	2.7950062	4.2576344	-1.0951750
H	3.4849412	5.7587674	-0.4494820
C	-1.3011148	0.0816064	-0.9333640
C	-0.5851018	0.0625104	0.2621230
C	-3.4048018	-0.0648566	0.2744060
C	-2.6878818	0.0209864	-0.9162240
H	-0.7900348	0.1676514	-1.8880680
C	-2.6970248	-0.0542356	1.4741780
H	-3.2269188	-0.1194456	2.4177750
C	-1.3170768	0.0223874	1.4449120
F	-3.3582578	0.0174304	-2.0746240

F	-0.6722448	-0.0154336	2.6359900
C	0.8304642	2.4970574	-1.3016590
C	1.3506132	2.0111264	-3.6436040
C	-0.1170198	3.4479004	-1.7279020
C	0.3849012	2.9215994	-4.0636400
H	1.9434852	1.4590444	-4.3680060
C	0.4174482	2.9379634	0.9949150
C	-0.3166318	3.6318914	-3.0991540
H	0.1931012	3.0882734	-5.1183450
C	0.7210832	2.6991254	2.3469510
C	-0.5426458	3.9242774	0.6948810
H	-1.0591288	4.3544224	-3.4264430
C	0.0987512	3.3823794	3.3752870
H	1.4478912	1.9314944	2.5686320
C	-1.1604728	4.6022134	1.7506280
C	-0.8610348	4.3480834	3.0816040
H	0.3567112	3.1545884	4.4057120
H	-1.9100048	5.3551964	1.5207400
H	-1.3674428	4.8914654	3.8726830
C	1.5725422	1.8235524	-2.2908960
H	2.3515222	1.1535274	-1.9546470
N	1.0877082	2.1856994	0.0296600
C	-0.9272218	4.2759554	-0.7390910
C	-0.6387548	5.7744804	-0.9842910
H	0.4211162	5.9876994	-0.8172590
H	-0.8874258	6.0623674	-2.0101530
H	-1.2254638	6.4059894	-0.3105820
C	-2.4276448	3.9893804	-0.9578310
H	-3.0469338	4.5376034	-0.2413940
H	-2.7472488	4.2815884	-1.9627080
H	-2.6293578	2.9219094	-0.8389830
C	-5.6242928	0.9369174	0.3805600
C	-5.8104808	3.3153454	0.8132860
C	-7.0118458	0.8515984	0.1953940
C	-7.1851438	3.2515824	0.6146990
H	-5.3285678	4.2568334	1.0581400
C	-5.3607658	-1.4693376	0.0066770
C	-7.7609738	2.0251934	0.3145240
H	-7.8011648	4.1409914	0.6941870
C	-4.5159278	-2.5903326	-0.0499350
C	-6.7423008	-1.6334136	-0.1733550
H	-8.8350058	1.9778074	0.1598710
C	-5.0261908	-3.8567016	-0.2845400
H	-3.4498018	-2.4624166	0.0892010
C	-7.2235508	-2.9236796	-0.4099000
C	-6.3930518	-4.0344076	-0.4671300
H	-4.3464908	-4.7031066	-0.3236580
H	-8.2896328	-3.0647826	-0.5615340

H	-6.8084528	-5.0190006	-0.6531630
N	-4.8166248	-0.2033976	0.2560100
C	-7.7217548	-0.4598576	-0.1419430
C	-8.3830598	-0.3270986	-1.5319840
H	-8.9082558	-1.2474006	-1.8052470
H	-9.1103848	0.4901024	-1.5431010
H	-7.6274568	-0.1241666	-2.2955840
C	-8.8036908	-0.7334426	0.9268010
H	-9.5379238	0.0758624	0.9617420
H	-9.3460518	-1.6587556	0.7131610
H	-8.3476798	-0.8256346	1.9161970
C	-5.0380418	2.1706884	0.7014940
H	-3.9700928	2.2368934	0.8665710

RE2_TS_Ac_R=F

Pd	1.3982442	0.0727833	0.3903545
P	1.1095092	-2.2120977	0.1888075
C	0.1175332	-3.0839267	1.5035465
H	0.2828272	-4.1663057	1.4189545
C	0.5990072	-2.6213397	2.8872165
H	0.5480392	-1.5265337	2.9200165
H	1.6492512	-2.8995777	3.0305705
C	-0.2582408	-3.1860897	4.0215325
H	-0.1574888	-4.2798877	4.0548535
H	0.1102932	-2.8039627	4.9796435
C	-1.7293968	-2.8164217	3.8238065
H	-1.8303718	-1.7231317	3.8526235
H	-2.3401738	-3.2180787	4.6392125
C	-2.2307098	-3.3348427	2.4764395
H	-2.1996908	-4.4334647	2.4790215
H	-3.2741508	-3.0487797	2.3064385
C	-1.3834518	-2.8066627	1.3172795
H	-1.5453928	-1.7278037	1.2217675
H	-1.7420638	-3.2612497	0.3907785
C	0.1810262	-2.5612077	-1.3776435
H	-0.7655578	-2.0346967	-1.1871735
C	0.8227662	-1.8641247	-2.5814275
H	1.0605712	-0.8284197	-2.3220165
H	1.7719512	-2.3595997	-2.8283865
C	-0.1177868	-1.9055647	-3.7881085
H	-1.0192288	-1.3245487	-3.5510595
H	0.3563302	-1.4213557	-4.6489585
C	-0.5140028	-3.3433967	-4.1361215
H	0.3749262	-3.8863297	-4.4861415
H	-1.2341428	-3.3495747	-4.9607975
C	-1.0961188	-4.0733057	-2.9215425
H	-1.3206838	-5.1148037	-3.1760195
H	-2.0479458	-3.6026747	-2.6377015

C	-0.1365298	-4.0199977	-1.7260755
H	0.7948992	-4.5400107	-1.9854385
H	-0.5716568	-4.5518637	-0.8725315
C	2.6842582	-3.1563467	0.0893985
C	2.6719272	-4.5576677	0.0868485
C	3.8463052	-5.2973957	0.0424005
H	3.8054102	-6.3818057	0.0389785
C	5.0687082	-4.6357127	0.0127205
H	5.9975002	-5.1968267	-0.0100375
C	5.0979692	-3.2475287	0.0212615
C	3.9236832	-2.4852487	0.0501025
C	4.0897162	-0.9900977	0.0735755
C	4.3840512	-0.2883287	-1.1176935
C	4.5016192	-1.0079617	-2.4575235
H	4.0176542	-1.9849137	-2.3585325
C	3.8296332	-0.2803267	-3.6273075
H	2.7640382	-0.1240687	-3.4532295
H	3.9337742	-0.8757207	-4.5398795
H	4.2893802	0.6943613	-3.8202435
C	5.9828682	-1.2518957	-2.7892835
H	6.0815882	-1.8041347	-3.7293965
H	6.4831682	-1.8252187	-2.0053405
H	6.5122062	-0.2989807	-2.8967475
C	4.6797112	1.0721183	-1.0407595
H	4.9008522	1.6130783	-1.9566305
C	4.7140182	1.7614163	0.1707925
C	5.0865482	3.2319743	0.2349995
C	6.4679942	3.4834883	-0.3843695
H	6.7644672	4.5277673	-0.2446475
H	6.4612982	3.2845243	-1.4615055
H	7.2307542	2.8449563	0.0712895
C	4.4415222	1.0473063	1.3326785
H	4.4908722	1.5668793	2.2854985
C	4.1344392	-0.3170317	1.3160495
C	4.0453322	-1.0867137	2.6289455
H	3.5556842	-2.0421327	2.4229705
C	5.4635632	-1.3978697	3.1315285
H	6.0331552	-1.9611307	2.3871245
H	5.4249402	-1.9907847	4.0510215
H	6.0081862	-0.4717567	3.3447575
C	3.2303942	-0.3894827	3.7201535
H	3.6850312	0.5557733	4.0352845
H	3.1698902	-1.0322797	4.6041285
H	2.2105982	-0.1863717	3.3858955
H	6.0517132	-2.7289147	0.0156835
H	1.7247362	-5.0855417	0.1239965
H	5.1452672	3.5005923	1.2972425
C	4.0203892	4.1275993	-0.4066895

H	3.8523262	3.8631443	-1.4562045
H	3.0664822	4.0347043	0.1160905
H	4.3318272	5.1765303	-0.3654695
C	-1.1244958	0.7120843	-1.0275875
C	-0.4545788	0.8626373	0.2101645
C	-3.2922128	0.4494553	0.0523165
C	-2.4892168	0.5141363	-1.0826915
H	-0.5756738	0.7405233	-1.9600925
C	-2.6499998	0.6069883	1.2840755
H	-3.2216888	0.5281313	2.2025495
C	-1.2895648	0.8026493	1.3516745
F	-3.0585188	0.3355893	-2.2873905
F	-0.7217768	0.8286533	2.5843335
C	0.9269162	2.7544723	-1.0620115
C	1.6723002	2.6989833	-3.3672765
C	0.3559542	4.0219643	-1.2600895
C	1.1251852	3.9608763	-3.5711225
H	2.1860662	2.1801903	-4.1708515
C	0.8741952	3.0133523	1.3005535
C	0.4844842	4.6090733	-2.5199605
H	1.1981892	4.4440243	-4.5400845
C	1.5175942	2.6316553	2.4774755
C	0.3108772	4.2953393	1.1916705
H	0.0686882	5.5941063	-2.6977525
C	1.5894682	3.4882213	3.5639835
H	1.9632502	1.6468043	2.5165305
C	0.4136732	5.1527243	2.2892995
C	1.0355632	4.7606433	3.4695455
H	2.0871692	3.1660473	4.4736475
H	-0.0021218	6.1519773	2.2310915
H	1.0911092	5.4501403	4.3058315
C	1.5815432	2.1125293	-2.1136565
H	2.0468202	1.1549083	-1.9092245
N	0.8556212	2.1193793	0.1995855
C	-0.3954628	4.6863483	-0.1060815
C	-0.4636158	6.2099233	-0.2789765
H	0.5332202	6.6592243	-0.3148495
H	-0.9990358	6.4719933	-1.1948065
H	-1.0244048	6.6672493	0.5396055
C	-1.8458988	4.1476593	-0.0731155
H	-2.4011438	4.6087353	0.7501685
H	-2.3526498	4.3828203	-1.0157125
H	-1.8680668	3.0674033	0.0684035
C	-5.5997518	1.2105793	-0.2410185
C	-6.0853918	3.4782003	-0.9123755
C	-6.9701648	0.9562503	-0.0616995
C	-7.4467278	3.2292213	-0.7842595
H	-5.7315528	4.4536963	-1.2317045

C	-5.1097418	-1.1578187	-0.2014815
C	-7.8738978	1.9742703	-0.3605945
H	-8.1733008	4.0056563	-1.0002045
C	-4.2118898	-2.1605307	-0.5802545
C	-6.4685988	-1.4658247	-0.0139415
H	-8.9375308	1.7968793	-0.2468735
C	-4.6527818	-3.4611867	-0.7828365
H	-3.1669938	-1.9176857	-0.7229325
C	-6.8903648	-2.7715907	-0.2589045
C	-5.9994498	-3.7716177	-0.6376105
H	-3.9374068	-4.2265417	-1.0712975
H	-7.9367698	-3.0270377	-0.1346965
H	-6.3563758	-4.7819617	-0.8078495
N	-4.6822318	0.1684823	-0.0084245
C	-7.3760588	-0.3792297	0.5583015
C	-8.8607958	-0.6907527	0.3477935
H	-9.1321518	-1.6296217	0.8373745
H	-9.4849898	0.0832063	0.8016205
H	-9.1126588	-0.7645037	-0.7143265
C	-7.1087118	-0.2956957	2.0826475
H	-7.7166548	0.4976963	2.5292825
H	-7.3585568	-1.2477127	2.5624665
H	-6.0570388	-0.0755217	2.2847615
C	-5.1642088	2.4731723	-0.6506915
H	-4.1048608	2.6607293	-0.7750875

RE1_P_Ac_R=CF3

C	1.9161542	1.2404809	1.1266387
C	1.5872392	0.4064459	0.0640227
C	4.1976872	1.3146209	0.4066737
C	3.2141002	1.7063139	1.3164237
H	1.1316862	1.5309829	1.8141677
C	3.8871172	0.4761279	-0.6557513
H	4.6608852	0.1796479	-1.3514423
C	2.5860362	0.0197649	-0.8328343
Br	6.0276312	1.8964439	0.5406367
C	-0.6569098	0.8184499	-0.7740433
C	-1.0488028	2.8261469	-2.0764753
C	-2.0156578	0.4853219	-0.8566493
C	-2.3979338	2.5067139	-2.1737063
H	-0.6587358	3.7255119	-2.5421703
C	-0.1935658	-1.1608671	0.6085397
C	-2.8565498	1.3467329	-1.5660273
H	-3.0834798	3.1482249	-2.7165903
C	0.7276372	-1.9172011	1.3479867
C	-1.5389138	-1.5503861	0.5615237
H	-3.9105738	1.0990479	-1.6485703
C	0.3269232	-3.0536641	2.0314047

H	1.7690232	-1.6209771	1.3813517
C	-1.9083018	-2.7044291	1.2572177
C	-1.0021488	-3.4585401	1.9885487
H	1.0596112	-3.6234311	2.5937667
H	-2.9446068	-3.0268621	1.2224807
H	-1.3291588	-4.3490991	2.5142367
C	-0.1869318	1.9908039	-1.3847713
H	0.8634682	2.2480539	-1.3275723
N	0.2412862	-0.0210711	-0.0906333
C	-2.5986038	-0.7773751	-0.2229173
C	-3.7401168	-0.3765221	0.7383767
H	-3.3583468	0.2626849	1.5390527
H	-4.5278708	0.1689859	0.2118097
H	-4.2012448	-1.2568501	1.1940747
C	-3.1523118	-1.6865421	-1.3427763
H	-3.5918428	-2.5973911	-0.9257973
H	-3.9286128	-1.1713481	-1.9160733
H	-2.3531308	-1.9747411	-2.0309273
C	3.4893762	2.6231479	2.4917337
C	2.2690402	-0.8916901	-1.9993693
F	3.3681822	-1.1428431	-2.7303483
F	1.3632012	-0.3448401	-2.8184663
F	1.7888822	-2.0696421	-1.5844653
F	2.3765752	2.8285939	3.2125967
F	4.4061532	2.1051979	3.3178447
F	3.9264542	3.8218659	2.0872697

RE1_R_Ac_R=CF3

Pd	0.0985254	-0.0342138	0.4406600
P	-0.7536286	2.1363722	0.2627520
C	-0.5228606	3.3739342	1.6245740
H	-1.0194656	4.2991932	1.3049850
C	-1.2003716	2.9014612	2.9195540
H	-0.7633826	1.9445192	3.2229880
H	-2.2679666	2.7300842	2.7427450
C	-1.0194216	3.9235772	4.0454400
H	-1.5589166	4.8456242	3.7876470
H	-1.4733876	3.5389032	4.9647730
C	0.4583054	4.2480172	4.2732610
H	0.9783974	3.3451022	4.6154450
H	0.5648744	4.9988562	5.0631430
C	1.1156464	4.7387782	2.9815670
H	0.6586484	5.6905912	2.6762610
H	2.1798904	4.9360062	3.1455910
C	0.9573524	3.7122202	1.8564920
H	1.5018234	2.8077182	2.1298190
H	1.4195424	4.0892582	0.9376790
C	0.0239464	2.9262152	-1.2285180

H	1.0990124	2.8539952	-1.0138860
C	-0.2582496	2.0732012	-2.4720840
H	-0.0024126	1.0287992	-2.2708060
H	-1.3354866	2.1003992	-2.6872270
C	0.5224294	2.5795772	-3.6858950
H	1.5944444	2.4392652	-3.5045270
H	0.2708284	1.9738972	-4.5634900
C	0.2363104	4.0583752	-3.9548650
H	-0.8146576	4.1756292	-4.2532550
H	0.8450984	4.4171842	-4.7910700
C	0.4987224	4.9063102	-2.7075090
H	0.2513924	5.9556372	-2.9001840
H	1.5688984	4.8724162	-2.4626440
C	-0.3075556	4.3978342	-1.5058050
H	-1.3768976	4.4952832	-1.7306030
H	-0.1001006	5.0227262	-0.6301370
C	-2.5672706	2.2139572	-0.0664180
C	-3.2164126	3.4577502	-0.0632360
C	-4.5775366	3.5725952	-0.3086780
H	-5.0484536	4.5500872	-0.3009610
C	-5.3261866	2.4273702	-0.5546460
H	-6.3932196	2.4955512	-0.7404890
C	-4.7010586	1.1890052	-0.5475360
C	-3.3264056	1.0550252	-0.3121290
C	-2.7820396	-0.3429998	-0.2765150
C	-2.6228986	-1.0829908	-1.4684580
C	-2.9969906	-0.5065688	-2.8322570
H	-3.1459306	0.5715322	-2.7173860
C	-1.9473276	-0.7055228	-3.9321500
H	-1.0233916	-0.1684648	-3.7149250
H	-2.3360036	-0.3250188	-4.8820370
H	-1.6954166	-1.7604618	-4.0779720
C	-4.3343826	-1.1159548	-3.2869590
H	-4.2257056	-2.1917578	-3.4606300
H	-4.6708326	-0.6537618	-4.2204850
H	-5.1183946	-0.9806308	-2.5374900
C	-2.2243816	-2.4180798	-1.3795260
H	-2.0528586	-2.9722278	-2.2967430
C	-2.0219766	-3.0532228	-0.1590320
C	-1.5467656	-4.4891938	-0.0373940
C	-2.7394916	-5.4271848	0.2014250
H	-2.3980176	-6.4556518	0.3560060
H	-3.4127406	-5.4183808	-0.6632100
H	-3.3177116	-5.1243998	1.0798740
C	-2.3131696	-2.3399448	1.0016450
H	-2.2376866	-2.8546748	1.9548260
C	-2.6694606	-0.9944198	0.9800830
C	-3.1230676	-0.3068948	2.2635730

H	-3.0872526	0.7726122	2.0990380
C	-4.5872696	-0.6787138	2.5440450
H	-5.2351136	-0.4023678	1.7075120
H	-4.9498386	-0.1647008	3.4400510
H	-4.6844876	-1.7575818	2.7058240
C	-2.2514256	-0.6007718	3.4853420
H	-2.2978446	-1.6539158	3.7826140
H	-2.5991836	-0.0081088	4.3374720
H	-1.2064066	-0.3454878	3.3019310
H	-5.2849926	0.2899892	-0.7163980
H	-2.6535246	4.3616302	0.1393790
H	-0.9044796	-4.5196368	0.8521820
C	-0.7046546	-4.9637338	-1.2226440
H	-1.3069926	-5.0678228	-2.1326320
H	0.1165424	-4.2729618	-1.4302850
H	-0.2790476	-5.9476398	-1.0036910
C	2.6394214	0.7829602	-0.7119590
C	2.0065664	0.5628572	0.5181430
C	4.7286154	1.2972512	0.3336250
C	3.9769454	1.1405642	-0.8323920
H	2.0839124	0.6285852	-1.6285120
C	4.1437194	1.0772882	1.5637760
H	4.7358674	1.1871892	2.4645640
C	2.7974624	0.7049742	1.6604140
Br	6.5956864	1.7905342	0.3239110
C	1.5662374	-2.0841578	-1.0356980
C	1.5555574	-1.6010228	-3.4305390
C	2.8241544	-2.6969358	-1.1720720
C	2.8297724	-2.1481808	-3.5545070
H	1.0430314	-1.1887898	-4.2953470
C	1.4180144	-2.6220568	1.2750390
C	3.4326044	-2.6972998	-2.4308740
H	3.3431714	-2.1560128	-4.5100240
C	0.6631414	-2.6357378	2.4603330
C	2.6314384	-3.3352608	1.2444670
H	4.4238044	-3.1293318	-2.5346480
C	1.0744854	-3.3256118	3.5859860
H	-0.2556236	-2.0685308	2.4758910
C	3.0300924	-4.0208708	2.3953380
C	2.2733344	-4.0322448	3.5592030
H	0.4636054	-3.3036738	4.4839900
H	3.9763224	-4.5542328	2.3883260
H	2.6206704	-4.5749338	4.4322960
C	0.9390914	-1.5837498	-2.1920480
H	-0.0569646	-1.1779968	-2.0771570
N	0.9048124	-1.9219558	0.1811100
C	3.5421184	-3.3218358	0.0190010
C	3.9531864	-4.7659968	-0.3420820

H	3.0689564	-5.3675318	-0.5715130
H	4.6149014	-4.7848728	-1.2120880
H	4.4909384	-5.2462018	0.4795570
C	4.7997004	-2.4869478	0.3463680
H	5.3508604	-2.9269578	1.1838290
H	5.4714044	-2.4299418	-0.5163310
H	4.5162004	-1.4689068	0.6241280
C	2.3405144	0.4566342	3.0782180
C	4.5572954	1.3197352	-2.2162980
F	2.7125844	1.4832992	3.8819460
F	2.9009404	-0.6445818	3.5869320
F	1.0106174	0.3404682	3.2181990
F	5.5609684	0.4658202	-2.4506470
F	5.0252534	2.5653102	-2.4007590
F	3.6344394	1.1108892	-3.1718770

RE1_TS_Ac_R=CF3

Pd	-0.0240790	0.0417155	0.4297587
P	0.5714750	-2.2001745	0.4136977
C	0.1423680	-3.2556705	1.8757157
H	0.4851750	-4.2806755	1.6807927
C	0.8504240	-2.7447175	3.1390707
H	0.5787870	-1.6943005	3.2954437
H	1.9371370	-2.7824985	3.0014997
C	0.4514600	-3.5574965	4.3737467
H	0.8126200	-4.5893775	4.2619117
H	0.9456300	-3.1442645	5.2596037
C	-1.0665720	-3.5687885	4.5638237
H	-1.4144670	-2.5471895	4.7591617
H	-1.3360880	-4.1723265	5.4370237
C	-1.7690780	-4.1002585	3.3128787
H	-1.4993430	-5.1550045	3.1620697
H	-2.8558690	-4.0657775	3.4413617
C	-1.3814940	-3.2919565	2.0723837
H	-1.7507550	-2.2700905	2.1845317
H	-1.8804150	-3.7046775	1.1888517
C	-0.2680870	-3.0325945	-1.0222703
H	-1.3260630	-2.7722055	-0.8737953
C	0.1865550	-2.3751905	-2.3310813
H	0.0612210	-1.2905865	-2.2518823
H	1.2599330	-2.5619545	-2.4714903
C	-0.5847260	-2.9115155	-3.5382203
H	-1.6339130	-2.6103545	-3.4567463
H	-0.1943200	-2.4547315	-4.4545903
C	-0.5025400	-4.4369825	-3.6208413
H	0.5360330	-4.7372565	-3.8183773
H	-1.1040620	-4.8032235	-4.4592243
C	-0.9702660	-5.0785815	-2.3124103

H	-0.8874540	-6.1692225	-2.3693703
H	-2.0313460	-4.8455785	-2.1546163
C	-0.1584160	-4.5590305	-1.1196093
H	0.8929190	-4.8423675	-1.2575003
H	-0.5065490	-5.0381685	-0.1982383
C	2.3698540	-2.5073785	0.1549057
C	2.8842540	-3.8037415	0.2961187
C	4.2338190	-4.0788455	0.1201107
H	4.6001090	-5.0938255	0.2349767
C	5.1057970	-3.0429145	-0.1935203
H	6.1655890	-3.2356745	-0.3261203
C	4.6147130	-1.7510245	-0.3226873
C	3.2551300	-1.4564855	-0.1597863
C	2.8615980	-0.0104565	-0.2787563
C	2.8029640	0.6168745	-1.5442333
C	3.0923280	-0.1442995	-2.8341583
H	3.0569190	-1.2145345	-2.6098123
C	2.0860200	0.1233615	-3.9591123
H	1.0671290	-0.1349115	-3.6662803
H	2.3428650	-0.4787365	-4.8363713
H	2.0937150	1.1732565	-4.2695473
C	4.5108160	0.1815445	-3.3294383
H	4.5949100	1.2440875	-3.5815643
H	4.7482530	-0.3996825	-4.2262203
H	5.2657680	-0.0407035	-2.5715083
C	2.5870220	1.9923245	-1.6004573
H	2.5468300	2.4798985	-2.5717873
C	2.4421880	2.7809625	-0.4588253
C	2.2473980	4.2838775	-0.6049433
C	0.8614800	4.6249315	-1.1724703
H	0.7957790	5.6969655	-1.3850403
H	0.0820930	4.3763145	-0.4467953
H	0.6464420	4.0781855	-2.0944133
C	2.5349060	2.1473605	0.7757017
H	2.4758740	2.7412065	1.6802417
C	2.7499270	0.7694035	0.8952617
C	3.0799630	0.1780275	2.2625717
H	2.9239270	-0.9020095	2.2091497
C	4.5676350	0.4144775	2.5665317
H	5.2062210	-0.0191015	1.7921217
H	4.8405580	-0.0378805	3.5253787
H	4.7818660	1.4873565	2.6213887
C	2.2126280	0.6963545	3.4103377
H	2.3619630	1.7659605	3.5940107
H	2.4724570	0.1705795	4.3345027
H	1.1524560	0.5259595	3.2169707
H	5.2969740	-0.9358735	-0.5422433
H	2.2196250	-4.6197715	0.5582177

H	2.9956050	4.6171015	-1.3377583
C	2.4852480	5.0650495	0.6888187
H	1.7128550	4.8367405	1.4320447
H	3.4656660	4.8483385	1.1248387
H	2.4353630	6.1391685	0.4880487
C	-2.6043130	-0.4219505	-0.7979503
C	-2.0480890	0.1018965	0.4073697
C	-4.5996830	-1.1979095	0.2810607
C	-3.8333040	-1.0390145	-0.8816863
H	-2.0380130	-0.3230225	-1.7116753
C	-4.0935160	-0.7362015	1.4750507
H	-4.6664350	-0.8898465	2.3807677
C	-2.8444180	-0.1121365	1.5730777
Br	-6.3095440	-2.1012545	0.3202817
C	-1.5276090	2.1538305	-1.2388543
C	-0.9909000	2.0487325	-3.5986443
C	-2.5322750	3.1009495	-1.4931623
C	-1.9812460	2.9875395	-3.8622803
H	-0.3873500	1.6400655	-4.4026943
C	-1.4388900	2.7456355	1.0519767
C	-2.7291210	3.5107175	-2.8125383
H	-2.1692100	3.3199195	-4.8780243
C	-0.5421230	2.8375095	2.1138437
C	-2.4363890	3.7212715	0.8861847
H	-3.4883180	4.2508865	-3.0369993
C	-0.6443510	3.8541755	3.0515697
H	0.2440480	2.0995935	2.1847187
C	-2.5132090	4.7457935	1.8301697
C	-1.6393150	4.8137785	2.9109357
H	0.0586400	3.8993585	3.8777677
H	-3.2672580	5.5171975	1.7260537
H	-1.7319660	5.6216765	3.6295577
C	-0.7594090	1.6518875	-2.2894063
H	0.0436790	0.9636925	-2.0484483
N	-1.2791390	1.7147975	0.0885047
C	-3.3652430	3.6307355	-0.3249393
C	-3.9967430	4.9908365	-0.6524893
H	-3.2396550	5.7476825	-0.8782513
H	-4.6741190	4.9070205	-1.5056973
H	-4.6062970	5.3433105	0.1830927
C	-4.5134390	2.6382095	-0.0290353
H	-5.0986070	2.9875195	0.8276697
H	-5.1733550	2.5593965	-0.8989293
H	-4.1390990	1.6432455	0.2023587
C	-2.4577340	0.2424305	2.9828007
C	-4.2696170	-1.5548025	-2.2322103
F	-2.9853840	-0.6370775	3.8684197
F	-2.8971590	1.4516045	3.3657317

F	-1.1266990	0.2121505	3.2030337
F	-5.5029610	-1.1562665	-2.5596643
F	-4.2490100	-2.9013275	-2.2726033
F	-3.4551890	-1.1289795	-3.2172803

RE2_P_Ac_R=CF3

C	-0.6477017	-0.0012846	-1.1871824
C	-1.3689147	-0.0008406	0.0014796
C	1.4249023	-0.0007116	0.0404106
C	0.7418223	-0.0012476	-1.1767404
H	-1.1914047	-0.0015816	-2.1239084
C	0.7028943	-0.0005056	1.2311806
H	1.2462293	-0.0002216	2.1683786
C	-0.6859617	-0.0006056	1.2222826
C	-3.4609287	-1.2285706	-0.1762894
C	-3.3700937	-3.6489766	-0.2741584
C	-4.8561887	-1.2630906	-0.3027384
C	-4.7533117	-3.7013776	-0.3949464
H	-2.7818767	-4.5608126	-0.2607554
C	-3.4601737	1.2281124	-0.1765594
C	-5.4700137	-2.5136446	-0.4048604
H	-5.2682577	-4.6521566	-0.4774524
C	-2.7288447	2.4247624	-0.1653314
C	-4.8554257	1.2634854	-0.3028684
H	-6.5511207	-2.5611436	-0.4901874
C	-3.3678117	3.6484464	-0.2750654
H	-1.6513697	2.3995944	-0.0599834
C	-5.4684757	2.5144064	-0.4051244
C	-4.7510217	3.7016914	-0.3955654
H	-2.7790077	4.5599084	-0.2620204
H	-6.5495637	2.5626174	-0.4902834
H	-5.2653987	4.6527694	-0.4781634
N	-2.7896487	-0.0004286	-0.0488524
C	-5.7171217	0.0004564	-0.2910504
C	-6.6363657	0.0006924	-1.5320964
H	-6.0427447	0.0004684	-2.4500424
H	-7.2866107	-0.8775896	-1.5448954
H	-7.2860467	0.8793904	-1.5449574
C	-6.5733117	0.0007644	0.9952706
H	-7.2143427	0.8866264	1.0368266
H	-7.2151027	-0.8845476	1.0367576
H	-5.9309297	0.0004574	1.8801096
C	3.5098923	-1.2190866	0.3351886
C	3.4573643	-3.5698376	0.8954656
C	4.9147773	-1.2384596	0.3336136
C	4.8437703	-3.5913076	0.9537766
H	2.8837783	-4.4692046	1.0947016
C	3.5091803	1.2189374	0.3348226

C	5.5541133	-2.4301076	0.6684696
H	5.3724753	-4.5053206	1.2017576
C	2.7896853	2.3892374	0.5933686
C	4.9140633	1.2391094	0.3332226
H	6.6369743	-2.4646266	0.6936476
C	3.4553883	3.5698414	0.8943906
H	1.7069603	2.3833094	0.5621846
C	5.5527493	2.4312134	0.6676756
C	4.8417843	3.5921174	0.9526516
H	2.8812983	4.4689384	1.0933876
H	6.6355963	2.4662954	0.6927836
H	5.3699843	4.5064994	1.2003376
N	2.8473313	-0.0003026	0.0686466
C	5.6634713	0.0004634	-0.1502884
C	5.6254403	0.0002034	-1.6993424
H	6.1300493	0.8909404	-2.0868434
H	6.1305293	-0.8903856	-2.0865594
H	4.5996783	-0.0001356	-2.0712234
C	7.1303843	0.0009424	0.2928776
H	7.6525983	-0.8719986	-0.1055014
H	7.6521313	0.8740154	-0.1058244
H	7.2282663	0.0011834	1.3823746
C	-2.7303667	-2.4256716	-0.1645824
H	-1.6528997	-2.4011866	-0.0589724
C	2.7910183	-2.3896966	0.5940656
H	1.7082903	-2.3843436	0.5628626
C	1.5063873	-0.0015536	-2.4806214
C	-1.4460177	-0.0001926	2.5309046
F	2.2881683	1.0817274	-2.5929684
F	0.6742373	-0.0024316	-3.5341124
F	2.2891493	-1.0842336	-2.5919474
F	-0.6050547	-0.0005306	3.5782786
F	-2.2271987	-1.0815856	2.6484316
F	-2.2261867	1.0819094	2.6482796

RE2_R_Ac_R=CF3

Pd	-1.4266256	-0.2027935	0.4134566
P	-1.3850956	2.1444195	0.2816926
C	-0.6651076	3.1740965	1.6493186
H	-0.8136266	4.2252555	1.3709076
C	-1.4032396	2.9327545	2.9739066
H	-1.3247636	1.8736955	3.2396996
H	-2.4686526	3.1598325	2.8550296
C	-0.8129836	3.7863805	4.1005646
H	-0.9965326	4.8482055	3.8847586
H	-1.3304086	3.5610675	5.0389976
C	0.6920414	3.5525595	4.2509516
H	0.8680634	2.5125575	4.5508216

H	1.0978324	4.1885155	5.0445216
C	1.4215614	3.8179315	2.9324426
H	1.3278974	4.8809735	2.6684986
H	2.4916044	3.6076005	3.0355646
C	0.8476494	2.9591645	1.8029646
H	1.0499554	1.9102925	2.0253606
H	1.3645974	3.1875785	0.8646096
C	-0.3869106	2.6068585	-1.2152274
H	0.5807614	2.1252485	-1.0211024
C	-0.9849066	1.9521825	-2.4669594
H	-1.1589226	0.8878335	-2.2827074
H	-1.9655686	2.4037285	-2.6718624
C	-0.0692806	2.1306905	-3.6795914
H	0.8587694	1.5726295	-3.5114764
H	-0.5423736	1.6918985	-4.5650374
C	0.2592804	3.6054715	-3.9223754
H	-0.6555856	4.1372575	-4.2184614
H	0.9661504	3.7038945	-4.7525474
C	0.8299124	4.2596435	-2.6611614
H	1.0233294	5.3230685	-2.8373294
H	1.7951404	3.7970665	-2.4138404
C	-0.1246516	4.0961885	-1.4715874
H	-1.0709516	4.6013245	-1.7023004
H	0.2979624	4.5856835	-0.5873564
C	-3.0328116	2.9252965	-0.0000874
C	-3.1457896	4.3235335	0.0223656
C	-4.3604636	4.9625505	-0.1835014
H	-4.4125066	6.0461385	-0.1619974
C	-5.5026966	4.2026795	-0.4077784
H	-6.4634686	4.6834055	-0.5612854
C	-5.4089846	2.8187095	-0.4232724
C	-4.1890826	2.1572935	-0.2303214
C	-4.2293506	0.6575525	-0.2283034
C	-4.3891376	-0.0566385	-1.4351604
C	-4.5318416	0.6531305	-2.7796804
H	-4.2431426	1.7000855	-2.6445654
C	-3.6635126	0.0848695	-3.9084484
H	-2.5992856	0.2062855	-3.7033314
H	-3.8835696	0.6137115	-4.8412024
H	-3.8528996	-0.9791945	-4.0805224
C	-6.0087716	0.6283865	-3.2090814
H	-6.3355076	-0.3997175	-3.3977984
H	-6.1524466	1.2044715	-4.1287594
H	-6.6625846	1.0464085	-2.4393334
C	-4.5363476	-1.4440825	-1.3778074
H	-4.6102136	-1.9985365	-2.3076874
C	-4.5709816	-2.1381235	-0.1733654
C	-4.6854906	-3.6487635	-0.0858684

C	-6.1397896	-4.0580825	0.1917436
H	-6.2183726	-5.1420965	0.3220286
H	-6.7855586	-3.7679905	-0.6447654
H	-6.5259226	-3.5774215	1.0959306
C	-4.5380846	-1.3965915	1.0057336
H	-4.6479436	-1.9238805	1.9486446
C	-4.3489406	-0.0175485	1.0142926
C	-4.4694966	0.7588575	2.3211026
H	-4.0245226	1.7452695	2.1698436
C	-5.9557876	0.9725625	2.6465446
H	-6.4690516	1.4986005	1.8367456
H	-6.0682356	1.5630585	3.5615186
H	-6.4573546	0.0105455	2.7967876
C	-3.7461086	0.1204735	3.5078076
H	-4.1897426	-0.8391345	3.7942746
H	-3.8119436	0.7804235	4.3786776
H	-2.6897176	-0.0453715	3.2901496
H	-6.3006976	2.2199455	-0.5786464
H	-2.2698116	4.9346955	0.2062816
H	-4.0758816	-3.9475395	0.7767076
C	-4.1335976	-4.3826755	-1.3088314
H	-4.7591176	-4.2221765	-2.1946774
H	-3.1157896	-4.0598515	-1.5422114
H	-4.1163146	-5.4596865	-1.1171844
C	1.2006824	-0.3253355	-0.8093874
C	0.5629614	-0.3508085	0.4373426
C	3.3858544	-0.2882225	0.2041436
C	2.5813214	-0.2940125	-0.9375734
H	0.6046144	-0.3337465	-1.7139914
C	2.7765754	-0.4147345	1.4411386
H	3.4038224	-0.4338385	2.3258896
C	1.3844034	-0.4578995	1.5650976
C	-0.8917036	-2.6271225	-1.1313914
C	-0.8123336	-2.1564755	-3.5267594
C	0.0319984	-3.6721915	-1.3121134
C	0.1415164	-3.1556555	-3.6989674
H	-1.1606246	-1.5713105	-4.3733314
C	-1.1830436	-3.1191185	1.1758776
C	0.5363614	-3.9008625	-2.5956334
H	0.5680184	-3.3570835	-4.6758564
C	-1.8811876	-2.8851565	2.3722136
C	-0.3273036	-4.2350875	1.1029186
H	1.2834384	-4.6757395	-2.7381784
C	-1.7548726	-3.7168155	3.4700646
H	-2.5173746	-2.0136805	2.4193976
C	-0.2144526	-5.0608615	2.2244826
C	-0.9151106	-4.8243245	3.4000066
H	-2.3068696	-3.4961695	4.3792726

H	0.4608844	-5.9101095	2.1896976
H	-0.7955146	-5.4880185	4.2501096
C	-1.3223176	-1.9114895	-2.2639124
H	-2.0789986	-1.1534925	-2.1116394
N	-1.3996786	-2.2445285	0.1092186
C	0.5435074	-4.4854205	-0.1268734
C	0.5531664	-5.9855425	-0.4832864
H	-0.4594816	-6.3334395	-0.7056594
H	1.1835994	-6.1849885	-1.3531244
H	0.9523894	-6.5889845	0.3354306
C	1.9806794	-4.0186575	0.1971246
H	2.3876834	-4.5747595	1.0489256
H	2.6438914	-4.1583735	-0.6635114
H	1.9733894	-2.9562595	0.4551306
C	5.6877034	-1.1254975	0.3113396
C	6.1208504	-3.4327055	0.8844456
C	7.0656564	-0.8872815	0.1723536
C	7.4877164	-3.2010845	0.8025036
H	5.7406354	-4.4158025	1.1438346
C	5.2357334	1.2644325	0.3019506
C	7.9428044	-1.9357865	0.4449426
H	8.1971734	-3.9980115	0.9991486
C	4.3386394	2.2870915	0.6315116
C	6.6051134	1.5481455	0.1630796
H	9.0112374	-1.7728365	0.3621056
C	4.7950874	3.5780345	0.8602596
H	3.2812874	2.0738195	0.7183806
C	7.0373354	2.8465065	0.4291406
C	6.1521084	3.8616685	0.7785926
H	4.0807514	4.3558135	1.1149226
H	8.0918524	3.0836975	0.3473016
H	6.5217894	4.8635775	0.9696296
N	4.7887424	-0.0590875	0.1068146
C	7.5227954	0.4585705	-0.3856984
C	7.3414044	0.4197935	-1.9240894
H	7.6339074	1.3805105	-2.3605734
H	7.9655144	-0.3701115	-2.3545444
H	6.3033894	0.2215665	-2.1959244
C	8.9986284	0.7392245	-0.0825994
H	9.6359974	-0.0311255	-0.5233074
H	9.3123324	1.6849085	-0.5314154
H	9.1923834	0.7803825	0.9934006
C	5.2238034	-2.4000425	0.6475546
H	4.1611824	-2.5868925	0.7280946
C	3.2030504	-0.2124005	-2.3076014
C	0.8899504	-0.6419915	2.9790766
F	1.6107964	0.1121485	3.8430426
F	1.0238284	-1.9116625	3.3758836

F	-0.3934496	-0.2917495	3.1660906
F	4.1519024	-1.1421605	-2.4851594
F	3.7692344	0.9913375	-2.5194804
F	2.2935894	-0.3892815	-3.2821474

RE2_TS_Ac_R=CF3

Pd	1.4515488	0.1540795	0.4040556
P	1.2769718	-2.1680055	0.4179216
C	0.6435998	-3.0305015	1.9360816
H	0.6889538	-4.1131275	1.7581176
C	1.5223958	-2.7118915	3.1535786
H	1.5622528	-1.6255105	3.2859286
H	2.5474458	-3.0565315	2.9750616
C	0.9701218	-3.3519955	4.4303816
H	1.0240298	-4.4461545	4.3426896
H	1.6004238	-3.0719555	5.2812526
C	-0.4809342	-2.9338185	4.6779816
H	-0.5233322	-1.8500855	4.8402036
H	-0.8648942	-3.4103515	5.5861036
C	-1.3592902	-3.2907445	3.4779176
H	-1.3917752	-4.3835625	3.3627226
H	-2.3901412	-2.9579375	3.6369056
C	-0.8239002	-2.6616955	2.1906186
H	-0.9240362	-1.5757985	2.2536696
H	-1.4406882	-2.9842245	1.3479646
C	0.0947598	-2.6745935	-0.9229464
H	-0.7962992	-2.0706755	-0.6947844
C	0.6221898	-2.2060575	-2.2849044
H	0.9007648	-1.1487325	-2.2237184
H	1.5383238	-2.7634285	-2.5239884
C	-0.4116642	-2.4193825	-3.3919484
H	-1.2682172	-1.7636395	-3.2125194
H	0.0157138	-2.1230855	-4.3563104
C	-0.8956402	-3.8702755	-3.4415684
H	-0.0660422	-4.5219965	-3.7498294
H	-1.6818362	-3.9786135	-4.1957494
C	-1.4073822	-4.3276415	-2.0730354
H	-1.7190162	-5.3769015	-2.1135004
H	-2.2947222	-3.7422605	-1.7962384
C	-0.3303742	-4.1463015	-0.9967464
H	0.5390338	-4.7649965	-1.2544614
H	-0.7054632	-4.5030325	-0.0315024
C	2.8489608	-3.0629595	0.0624996
C	2.8983568	-4.4595225	0.1773176
C	4.0703118	-5.1712735	-0.0409264
H	4.0736948	-6.2522945	0.0535596
C	5.2344698	-4.4858365	-0.3680994
H	6.1636488	-5.0230175	-0.5292394

C	5.2042368	-3.1028185	-0.4806884
C	4.0266158	-2.3707735	-0.2821764
C	4.1328368	-0.8799935	-0.4331834
C	4.2186208	-0.3116205	-1.7206344
C	4.1110588	-1.1554605	-2.9859824
H	3.6885138	-2.1256325	-2.7082804
C	3.1975558	-0.5460505	-4.0569054
H	2.1987738	-0.3308545	-3.6720774
H	3.0900228	-1.2440435	-4.8930694
H	3.6130128	0.3832085	-4.4607664
C	5.5046358	-1.4068455	-3.5829134
H	5.9778398	-0.4604865	-3.8662004
H	5.4317948	-2.0310785	-4.4793024
H	6.1637448	-1.9109285	-2.8721984
C	4.4943838	1.0513505	-1.8333484
H	4.5737468	1.4937745	-2.8230394
C	4.6673598	1.8752305	-0.7259524
C	5.0147758	3.3429445	-0.9022574
C	3.9487738	4.2775305	-0.3203424
H	4.2335208	5.3220855	-0.4835244
H	3.8260788	4.1272535	0.7569286
H	2.9781328	4.1100745	-0.7921054
C	4.5759278	1.2945725	0.5382586
H	4.7367978	1.9151155	1.4152986
C	4.3378188	-0.0722585	0.7120046
C	4.5286698	-0.7100035	2.0850266
H	4.0503838	-1.6922815	2.0677226
C	6.0302098	-0.9341375	2.3243866
H	6.4716598	-1.5473205	1.5339446
H	6.1961468	-1.4404345	3.2807106
H	6.5623508	0.0230565	2.3467496
C	3.9125518	0.0630635	3.2511346
H	4.3624538	1.0538035	3.3774166
H	4.0732228	-0.4876485	4.1832936
H	2.8359148	0.1875515	3.1211316
H	6.1140058	-2.5616525	-0.7213014
H	2.0040998	-5.0086745	0.4505326
H	5.0646998	3.5304465	-1.9822704
C	6.3988688	3.6503545	-0.3135114
H	6.4017408	3.5155465	0.7736486
H	7.1655948	2.9942615	-0.7364014
H	6.6811058	4.6877735	-0.5187924
C	-1.1052632	0.6466055	-0.8791594
C	-0.4411162	0.8679105	0.3654366
C	-3.2793302	0.4543305	0.1505666
C	-2.4644922	0.4768365	-0.9906574
H	-0.5211312	0.6407855	-1.7870234
C	-2.6651852	0.6430555	1.3739596

H	-3.2830542	0.5880965	2.2631576
C	-1.2901142	0.8524215	1.5156606
C	0.7593238	2.7352015	-1.1545654
C	1.2867818	2.5982175	-3.5148914
C	0.1080458	3.9635415	-1.3550764
C	0.6454888	3.8131945	-3.7255514
H	1.7476158	2.0636375	-4.3395274
C	0.9792438	3.1373275	1.1650306
C	0.0751718	4.4850975	-2.6493614
H	0.5926378	4.2434975	-4.7203284
C	1.8403398	2.8829215	2.2291326
C	0.3422148	4.3844685	1.0579816
H	-0.4115452	5.4358655	-2.8330344
C	2.0384368	3.8208445	3.2308236
H	2.3638548	1.9379725	2.2456276
C	0.5670328	5.3228215	2.0654616
C	1.3924488	5.0484705	3.1515936
H	2.7049778	3.5960045	4.0578146
H	0.0940308	6.2963595	2.0091016
H	1.5384158	5.7993925	3.9213776
C	1.3507678	2.0748335	-2.2320004
H	1.8778128	1.1479495	-2.0335174
N	0.8262468	2.1638935	0.1425656
C	-0.5404332	4.6626655	-0.1590804
C	-0.7045622	6.1686315	-0.4059074
H	0.2571638	6.6589915	-0.5831274
H	-1.3546522	6.3509505	-1.2650124
H	-1.1900692	6.6483745	0.4473436
C	-1.9484742	4.0680525	0.0738966
H	-2.4150082	4.5427365	0.9438466
H	-2.5815382	4.2364995	-0.8034524
H	-1.9029422	2.9966005	0.2578776
C	-5.6397022	1.1075745	0.2602296
C	-6.2489772	3.3764345	0.8345046
C	-6.9971602	0.7667385	0.1232636
C	-7.5946312	3.0414385	0.7564046
H	-5.9455172	4.3860735	1.0937396
C	-5.0091932	-1.2384165	0.2186636
C	-7.9511992	1.7452785	0.3983166
H	-8.3626982	3.7814565	0.9555816
C	-4.0308292	-2.1954365	0.5101096
C	-6.3516862	-1.6285435	0.0796006
H	-9.0041422	1.5009085	0.3162726
C	-4.3770952	-3.5244215	0.7079186
H	-2.9963912	-1.8910135	0.5910106
C	-6.6746382	-2.9652625	0.3114476
C	-5.7079732	-3.9150365	0.6276006
H	-3.5994312	-4.2479625	0.9378866

H	-7.7069072	-3.2855875	0.2279996
H	-5.9943212	-4.9483065	0.7932556
N	-4.6603672	0.1163925	0.0611496
C	-7.3567842	-0.6055275	-0.4425784
C	-7.1981532	-0.5362805	-1.9825084
H	-7.4201782	-1.5120315	-2.4273444
H	-7.8862582	0.2078465	-2.3970184
H	-6.1814262	-0.2553075	-2.2625694
C	-8.8027372	-1.0023145	-0.1245714
H	-9.5045472	-0.2820415	-0.5517924
H	-9.0469482	-1.9669605	-0.5762084
H	-8.9787002	-1.0657205	0.9533876
C	-5.2759812	2.4159435	0.5948616
H	-4.2294422	2.6806275	0.6735996
C	-3.0768632	0.3051115	-2.3560484
C	-0.8394922	1.0180725	2.9416656
F	-1.6273892	0.3111745	3.7878556
F	-0.8994612	2.2902595	3.3663916
F	0.4154448	0.5782955	3.1742556
F	-4.0739912	1.1779385	-2.5641744
F	-3.5847052	-0.9318845	-2.5279384
F	-2.1812442	0.4937705	-3.3426744

RE1_P_Cz_R=CF3

C	1.9416518	-0.0496201	0.7080505
C	0.9771128	-0.4268991	-0.2187905
C	3.5957548	-1.3398361	-0.4472635
C	3.2574618	-0.4958671	0.6115755
H	1.6510268	0.6068449	1.5187965
C	2.6418148	-1.7250681	-1.3808335
H	2.9185728	-2.3812681	-2.1954765
C	1.3327368	-1.2738411	-1.2717425
Br	5.3759648	-2.0266301	-0.7019195
C	-1.3201112	-0.5205331	0.7421275
C	-0.8286122	1.2594709	-0.5694425
C	-1.2685512	-1.7102851	1.4628105
C	-2.4542772	0.3145629	0.7476365
C	-0.1952192	2.1790619	-1.4005155
C	-2.1396472	1.4543049	-0.0927665
C	-2.3921042	-2.0561991	2.2016285
H	-0.3873062	-2.3428921	1.4429365
C	-3.5721522	-0.0579241	1.4950465
C	-0.9108302	3.3147629	-1.7552245
H	0.8141468	2.0098989	-1.7605225
C	-2.8389172	2.6022169	-0.4673295
C	-3.5336762	-1.2418261	2.2190625
H	-2.3862072	-2.9768261	2.7763665
H	-4.4573262	0.5705119	1.5106325

C	-2.2192442	3.5266309	-1.2971695
H	-0.4477522	4.0525419	-2.4026525
H	-3.8519542	2.7690949	-0.1141365
H	-4.3947572	-1.5428441	2.8062045
H	-2.7510262	4.4235199	-1.5966565
N	-0.3449172	0.0516359	-0.0714165
C	0.3044118	-1.7021581	-2.2964455
C	4.2522878	-0.0461811	1.6636195
F	4.7587638	-1.0859111	2.3368535
F	5.2701278	0.6320789	1.1209605
F	3.6688598	0.7626399	2.5611105
F	0.8441318	-2.5248451	-3.2103515
F	-0.7134652	-2.3534241	-1.7223755
F	-0.1967732	-0.6489011	-2.9523875

RE1_R_Cz_R=CF3

Pd	0.2613812	-0.2597938	0.4617077
P	0.4775802	2.0488162	0.2024007
C	1.1043842	3.1290922	1.5696637
H	1.0246642	4.1634172	1.2083097
C	0.2318912	3.0031752	2.8274587
H	0.2339512	1.9609562	3.1609717
H	-0.8055488	3.2658362	2.5922727
C	0.7511392	3.8974102	3.9569217
H	0.6334032	4.9514592	3.6698367
H	0.1402102	3.7447952	4.8526737
C	2.2242402	3.6163752	4.2609067
H	2.3291972	2.5902052	4.6346167
H	2.5816692	4.2838872	5.0517397
C	3.0815292	3.7764932	3.0039457
H	3.0479562	4.8227022	2.6686137
H	4.1293732	3.5476092	3.2227317
C	2.5862922	2.8650292	1.8783847
H	2.7195152	1.8266622	2.1839827
H	3.2010842	3.0070832	0.9832367
C	1.5792972	2.4291052	-1.2407713
H	2.5156072	1.9132352	-0.9874843
C	1.0134312	1.7735092	-2.5061173
H	0.7739272	0.7249752	-2.3035633
H	0.0661072	2.2628122	-2.7682603
C	1.9913122	1.8895662	-3.6761013
H	2.8977422	1.3175852	-3.4465223
H	1.5510492	1.4367812	-4.5708943
C	2.3601552	3.3513442	-3.9407333
H	1.4684232	3.8930872	-4.2846893
H	3.1008022	3.4158582	-4.7441183
C	2.8965582	4.0229412	-2.6736603
H	3.1147182	5.0789202	-2.8644753

H	3.8443442	3.5494152	-2.3848793
C	1.9025312	3.9042002	-1.5114283
H	0.9805232	4.4369132	-1.7762513
H	2.3162432	4.3902612	-0.6217413
C	-1.1448538	2.7869702	-0.2290663
C	-1.3024928	4.1784242	-0.2917013
C	-2.5229628	4.7534292	-0.6166963
H	-2.6203258	5.8331092	-0.6616443
C	-3.6171348	3.9340502	-0.8746393
H	-4.5815838	4.3673262	-1.1189573
C	-3.4755118	2.5556292	-0.8013883
C	-2.2487278	1.9570362	-0.4867673
C	-2.2350418	0.4561882	-0.3840043
C	-2.3190528	-0.3357528	-1.5569083
C	-2.3099988	0.2794212	-2.9544473
H	-1.9840338	1.3204342	-2.8669983
C	-1.3787458	-0.4123368	-3.9579473
H	-0.3286658	-0.3176108	-3.6802523
H	-1.5007838	0.0466062	-4.9442493
H	-1.5976798	-1.4792378	-4.0591923
C	-3.7414458	0.2851612	-3.5189873
H	-4.1010328	-0.7391838	-3.6614313
H	-3.7694268	0.7923012	-4.4885423
H	-4.4418548	0.7918352	-2.8510803
C	-2.5607418	-1.7002608	-1.4255793
H	-2.5985898	-2.3058828	-2.3238903
C	-2.7561918	-2.3134558	-0.1899203
C	-3.0118168	-3.8038308	-0.0462493
C	-4.4236858	-4.0574798	0.5052147
H	-4.5837638	-5.1282918	0.6636957
H	-5.1815948	-3.7008708	-0.2014083
H	-4.5874148	-3.5499788	1.4601467
C	-2.7298838	-1.5105398	0.9476357
H	-2.8960198	-1.9730878	1.9158907
C	-2.4752728	-0.1395328	0.8872787
C	-2.7063118	0.7242372	2.1242067
H	-2.2533298	1.7022082	1.9445627
C	-4.2162258	0.9419042	2.3081937
H	-4.6650688	1.3982052	1.4218077
H	-4.4088078	1.5979152	3.1630987
H	-4.7199568	-0.0134818	2.4887917
C	-2.0870388	0.1755862	3.4094817
H	-2.5328798	-0.7777198	3.7094907
H	-2.2470338	0.8854242	4.2273607
H	-1.0123728	0.0280002	3.2960767
H	-4.3345488	1.9158892	-0.9751553
H	-0.4617778	4.8290562	-0.0762873
H	-2.2888168	-4.1666498	0.6953607

C	-2.7924388	-4.6002478	-1.3329903
H	-3.5510008	-4.3568418	-2.0869643
H	-1.8025598	-4.4230148	-1.7614383
H	-2.8750718	-5.6699708	-1.1211773
C	2.8938732	-0.6336208	-0.6838263
C	2.2382272	-0.5177208	0.5479777
C	5.0255862	-0.9558998	0.3522667
C	4.2603122	-0.8536678	-0.8102253
H	2.3233092	-0.5723318	-1.5989623
C	4.4119032	-0.8701098	1.5854877
H	5.0076282	-0.9602798	2.4860947
C	3.0321552	-0.6596138	1.6886997
Br	6.9391182	-1.2027508	0.3350237
C	0.1162442	-3.2053318	1.4651807
C	0.5082972	-3.0526038	-0.7004003
C	-0.2355798	-2.9673048	2.7988487
C	0.3674172	-4.5320388	1.0240687
C	0.6364882	-2.6194768	-2.0249383
C	0.6314302	-4.4304608	-0.3897633
C	-0.3262138	-4.0445338	3.6663337
H	-0.4232938	-1.9601878	3.1454467
C	0.2687082	-5.6039108	1.9134717
C	0.9030732	-3.5536318	-3.0131163
H	0.4928692	-1.5734088	-2.2668033
C	0.8998752	-5.3587828	-1.3974423
C	-0.0771988	-5.3570298	3.2338817
H	-0.5923168	-3.8674188	4.7045807
H	0.4532342	-6.6185708	1.5710797
C	1.0399912	-4.9177248	-2.7062033
H	1.0025782	-3.2236708	-4.0435093
H	0.9892982	-6.4155618	-1.1612213
H	-0.1575128	-6.1788708	3.9383497
H	1.2486002	-5.6289688	-3.4989043
N	0.1947252	-2.3099868	0.4176237
C	4.8465932	-0.9611458	-2.1995483
C	2.5235412	-0.6428358	3.1097137
F	2.5096362	-1.8669408	3.6432417
F	1.2878812	-0.1284008	3.2430297
F	3.3252822	0.1144912	3.8959697
F	5.5177242	-2.1016938	-2.3803663
F	5.6845682	0.0575832	-2.4587143
F	3.8868362	-0.9183938	-3.1434373

RE1_TS_Cz_R=CF3

Pd	-0.1424395	0.2046339	0.4284389
P	0.1129265	-2.0883581	0.3308009
C	-0.3830615	-3.1456581	1.7634109
H	-0.1621295	-4.1933381	1.5169399

C	0.4203195	-2.7666991	3.0165209
H	0.2784785	-1.6985191	3.2166469
H	1.4898545	-2.9254701	2.8377499
C	-0.0335865	-3.5732341	4.2358639
H	0.1942635	-4.6360351	4.0743109
H	0.5352175	-3.2576841	5.1170329
C	-1.5348285	-3.4067261	4.4787619
H	-1.7483055	-2.3573021	4.7136549
H	-1.8489465	-4.0008771	5.3434149
C	-2.3365965	-3.8090301	3.2390979
H	-2.1999995	-4.8829291	3.0496789
H	-3.4059925	-3.6485141	3.4081269
C	-1.8959855	-3.0146941	2.0069259
H	-2.1446055	-1.9613651	2.1539649
H	-2.4607885	-3.3459751	1.1287669
C	-0.8306575	-2.7649211	-1.1178311
H	-1.8413085	-2.3585481	-0.9659391
C	-0.2802205	-2.1673601	-2.4201441
H	-0.2047375	-1.0771191	-2.3289751
H	0.7421145	-2.5384871	-2.5742581
C	-1.1433395	-2.5526251	-3.6232951
H	-2.1340995	-2.0995071	-3.5154081
H	-0.7055155	-2.1426491	-4.5405051
C	-1.2886915	-4.0717121	-3.7350791
H	-0.3085865	-4.5173971	-3.9545631
H	-1.9491075	-4.3287611	-4.5695471
C	-1.8286875	-4.6649051	-2.4314601
H	-1.9058895	-5.7544961	-2.5101251
H	-2.8435455	-4.2844511	-2.2555381
C	-0.9414345	-4.2903541	-1.2378381
H	0.0582325	-4.7201571	-1.3860091
H	-1.3510445	-4.7300301	-0.3226751
C	1.8698005	-2.5120101	0.0191749
C	2.3069795	-3.8414671	0.0751039
C	3.6386055	-4.1752321	-0.1332751
H	3.9529095	-5.2127481	-0.0851441
C	4.5621915	-3.1678511	-0.3898441
H	5.6094095	-3.4076041	-0.5436021
C	4.1427035	-1.8448001	-0.4379441
C	2.8016985	-1.4900351	-0.2453801
C	2.4736535	-0.0200551	-0.2939821
C	2.4400925	0.6598229	-1.5382731
C	2.6059205	-0.0685311	-2.8680391
H	2.4487715	-1.1342751	-2.6852661
C	1.6110705	0.3914189	-3.9623741
H	0.9654755	1.2030399	-3.6228921
H	0.9645315	-0.4299021	-4.2822331
H	2.1386265	0.7510829	-4.8508521

C	4.0491855	0.0975549	-3.3754221
H	4.2675555	1.1538599	-3.5653361
H	4.1885765	-0.4512181	-4.3121021
H	4.7815425	-0.2720241	-2.6541301
C	2.3776075	2.0487749	-1.5425851
H	2.3913085	2.5621849	-2.5006971
C	2.3422705	2.8128039	-0.3731361
C	2.3967395	4.3326949	-0.4704461
C	1.2766875	4.9095409	-1.3462491
H	1.4773445	5.9629119	-1.5656021
H	0.3232925	4.8534109	-0.8197981
H	1.1599135	4.3818889	-2.2965781
C	2.3634815	2.1301569	0.8369589
H	2.3521445	2.6892029	1.7649079
C	2.4568495	0.7311019	0.9077739
C	2.7963515	0.0777429	2.2447239
H	2.5638245	-0.9876491	2.1699069
C	4.3073105	0.2055639	2.4951449
H	4.8871495	-0.2427941	1.6843419
H	4.5837725	-0.2951701	3.4284329
H	4.5930925	1.2598599	2.5751549
C	2.0140395	0.6295619	3.4361329
H	2.2516005	1.6793399	3.6375919
H	2.2634725	0.0615839	4.3379649
H	0.9399055	0.5471879	3.2728459
H	4.8687185	-1.0576101	-0.6148151
H	1.5953155	-4.6304001	0.2962699
H	3.3527605	4.5573699	-0.9658041
C	2.3995745	5.0415579	0.8851609
H	1.4688685	4.8470709	1.4305789
H	3.2432585	4.7348689	1.5114969
H	2.4753595	6.1224279	0.7357309
C	-2.7927095	0.1019969	-0.7317331
C	-2.1404935	0.5321019	0.4642819
C	-4.9586345	-0.0276231	0.2816999
C	-4.1443745	-0.1424191	-0.8513411
H	-2.2017795	-0.0404321	-1.6201851
C	-4.3609575	0.2552599	1.4879169
H	-4.9695775	0.2560349	2.3847089
C	-2.9833675	0.4759429	1.6159089
Br	-6.8618955	-0.3703571	0.2794869
C	-1.4760575	2.6026719	-1.0983311
C	-1.6767045	2.8151259	-3.4729711
C	-1.9815085	3.9075339	-0.9455971
C	-2.2221185	4.0944819	-3.3229041
H	-1.5357515	2.4017439	-4.4669751
C	-1.2612485	3.0989159	1.0713149
C	-2.3637505	4.6526719	-2.0578181

H	-2.5121965	4.6616619	-4.2013021
C	-0.7840665	3.1641869	2.3754879
C	-1.8263095	4.2366809	0.4590179
H	-2.7454025	5.6622139	-1.9378391
C	-0.9390695	4.3547839	3.0780349
H	-0.2849405	2.3177969	2.8254279
C	-1.9755445	5.4229509	1.1714769
C	-1.5408095	5.4722579	2.4911559
H	-0.5702845	4.4182599	4.0970609
H	-2.3979355	6.3021659	0.6942609
H	-1.6420255	6.3910329	3.0595719
C	-1.2952115	2.0624729	-2.3681231
H	-0.8239235	1.0957709	-2.4971001
N	-1.1728325	2.0358569	0.1532149
C	-2.5523945	0.5304879	3.0545889
C	-4.6511505	-0.6090011	-2.1950131
F	-3.1824805	-0.4274951	3.7808039
F	-2.8439025	1.6875709	3.6687459
F	-1.2339055	0.2999709	3.2354679
F	-5.7448495	0.0440569	-2.5954461
F	-4.9408695	-1.9247131	-2.1773471
F	-3.7275595	-0.4355741	-3.1641221

RE2_P_Cz_R=CF3

C	-0.6765878	0.0252506	-1.2101347
C	-1.3942888	0.0233006	-0.0181697
C	1.3941402	0.0235216	0.0190463
C	0.7126762	0.0254726	-1.2016147
H	-1.2215408	0.0237776	-2.1461347
C	0.6764372	0.0260306	1.2110033
H	1.2214022	0.0250626	2.1469963
C	-0.7128358	0.0260336	1.2024803
C	-3.6210748	1.1341856	-0.2001637
C	-3.5955908	-1.1293324	-0.2006197
C	-3.2662028	2.4790796	-0.1553677
C	-4.9508788	0.7123096	-0.3930427
C	-3.2096348	-2.4657234	-0.1563127
C	-4.9344968	-0.7382184	-0.3934767
C	-4.2831718	3.4112966	-0.3076837
H	-2.2371398	2.7837296	0.0024063
C	-5.9549498	1.6696356	-0.5405817
C	-4.2048118	-3.4209824	-0.3092467
H	-2.1737478	-2.7463564	0.0014143
C	-5.9163418	-1.7181854	-0.5415937
C	-5.6143988	3.0143946	-0.4978157
H	-4.0424128	4.4689396	-0.2743057
H	-6.9866728	1.3659976	-0.6881547
C	-5.5447778	-3.0547924	-0.4994767

H	-3.9397448	-4.4728214	-0.2764577
H	-6.9548068	-1.4386664	-0.6894247
H	-6.3851018	3.7694086	-0.6104457
H	-6.2981958	-3.8269914	-0.6125897
N	-2.8080408	0.0114676	-0.0688727
C	3.6211432	1.1342286	0.2004733
C	3.5953732	-1.1292984	0.2009583
C	3.2664432	2.4791536	0.1556713
C	4.9510262	0.7121606	0.3924903
C	3.2092442	-2.4656594	0.1569683
C	4.9344362	-0.7383534	0.3930003
C	4.2836632	3.4112456	0.3071913
H	2.2373202	2.7839276	-0.0015287
C	5.9553322	1.6693546	0.5392193
C	4.2044002	-3.4210014	0.3094893
H	2.1732732	-2.7461834	-0.0003267
C	5.9162632	-1.7184074	0.5407353
C	5.6149532	3.0141606	0.4965143
H	4.0430572	4.4689216	0.2738333
H	6.9871042	1.3655526	0.6861333
C	5.5445172	-3.0549654	0.4989873
H	3.9392112	-4.4728184	0.2770033
H	6.9548372	-1.4389664	0.6879403
H	6.3858372	3.7690846	0.6085133
H	6.2978892	-3.8272444	0.6118153
N	2.8079052	0.0116356	0.0697593
C	-1.4765888	0.0240626	2.5087503
C	1.4764402	0.0235896	-2.5078847
F	2.2485102	-1.0643124	-2.6210797
F	2.2641642	1.1008156	-2.6152677
F	0.6398002	0.0324436	-3.5576087
F	-0.6398588	0.0338786	3.5584433
F	-2.2477058	-1.0644654	2.6222063
F	-2.2651998	1.1006356	2.6159413

RE2_R_Cz_R=CF3

Pd	-0.9101540	0.0973902	-0.3755828
P	-1.7343210	-2.0512258	-0.1329928
C	-1.6410740	-3.2539508	-1.5344308
H	-2.1681910	-4.1635718	-1.2151748
C	-2.3516690	-2.7005088	-2.7783388
H	-1.8892880	-1.7474528	-3.0521968
H	-3.4047280	-2.5000908	-2.5515668
C	-2.2465250	-3.6675878	-3.9604298
H	-2.7991240	-4.5887818	-3.7290208
H	-2.7263120	-3.2226058	-4.8383428
C	-0.7877250	-4.0141238	-4.2642448
H	-0.2547130	-3.1061458	-4.5708918

H	-0.7306900	-4.7198988	-5.0992938
C	-0.1005290	-4.5986038	-3.0285648
H	-0.5770550	-5.5522668	-2.7620488
H	0.9505810	-4.8163088	-3.2427838
C	-0.1839450	-3.6356428	-1.8410848
H	0.3838110	-2.7343058	-2.0786418
H	0.2923610	-4.0838108	-0.9624588
C	-0.9372260	-2.9167958	1.2996872
H	0.1340680	-2.8520538	1.0666892
C	-1.1870380	-2.1208368	2.5866462
H	-0.9602410	-1.0626368	2.4212952
H	-2.2558210	-2.1733838	2.8333592
C	-0.3674280	-2.6778168	3.7508292
H	0.6994940	-2.5409838	3.5389512
H	-0.5842180	-2.1083908	4.6609092
C	-0.6626440	-4.1641188	3.9667062
H	-1.7033340	-4.2819588	4.2984702
H	-0.0287000	-4.5644398	4.7642262
C	-0.4553800	-4.9634238	2.6771572
H	-0.7134010	-6.0157688	2.8351502
H	0.6073200	-4.9366408	2.4008322
C	-1.2859860	-4.3944768	1.5196032
H	-2.3512610	-4.4872888	1.7650902
H	-1.1078480	-4.9832788	0.6139132
C	-3.5140330	-1.9604898	0.2886132
C	-4.2965220	-3.1214288	0.3496292
C	-5.6443100	-3.0649028	0.6764752
H	-6.2311390	-3.9765238	0.7194522
C	-6.2324490	-1.8313358	0.9364142
H	-7.2875790	-1.7667828	1.1818892
C	-5.4689840	-0.6744178	0.8625282
C	-4.1053820	-0.7118948	0.5443832
C	-3.3970000	0.6129432	0.4269472
C	-3.0660010	1.3511172	1.5945842
C	-3.3034250	0.7865832	2.9923952
H	-3.4593620	-0.2922458	2.8992682
C	-2.1538420	1.0060982	3.9826012
H	-1.2296530	0.5269612	3.6587072
H	-2.4232050	0.5815942	4.9548562
H	-1.9390300	2.0679112	4.1346102
C	-4.5890760	1.3955602	3.5794432
H	-4.4688780	2.4746692	3.7204152
H	-4.8131970	0.9482412	4.5529472
H	-5.4514130	1.2387472	2.9278372
C	-2.6461090	2.6706382	1.4572342
H	-2.3576000	3.2175252	2.3477762
C	-2.5811260	3.3102272	0.2202402
C	-2.0997680	4.7415062	0.0574612

C	-3.2649580	5.6559772	-0.3520828
H	-2.9061920	6.6743102	-0.5303138
H	-4.0203860	5.6945372	0.4406382
H	-3.7538280	5.3060432	-1.2659818
C	-2.9921640	2.6004382	-0.9048488
H	-2.9730060	3.0988882	-1.8694138
C	-3.3913980	1.2651262	-0.8398378
C	-4.0304690	0.6093262	-2.0605538
H	-4.0915440	-0.4658048	-1.8752958
C	-5.4669910	1.1327222	-2.2168398
H	-6.0608000	0.9441822	-1.3186438
H	-5.9628310	0.6449372	-3.0620898
H	-5.4635320	2.2124602	-2.3991048
C	-3.2545760	0.7956432	-3.3645018
H	-3.2046950	1.8464622	-3.6672148
H	-3.7503250	0.2463522	-4.1712738
H	-2.2349210	0.4172012	-3.2769758
H	-5.9358070	0.2899512	1.0337352
H	-3.8508260	-4.0862468	0.1320002
H	-1.3758860	4.7247792	-0.7673848
C	-1.3831020	5.3027082	1.2865532
H	-2.0785720	5.4539622	2.1210042
H	-0.5732510	4.6477652	1.6188792
H	-0.9465400	6.2758772	1.0454622
C	1.6172020	-0.7186678	0.7541202
C	0.9907740	-0.5072408	-0.4808578
C	2.9944950	-0.7713548	0.8938582
H	1.0238640	-0.7746858	1.6554222
C	3.2047810	-0.5231038	-1.4781278
H	3.8414540	-0.4355508	-2.3524378
C	1.8144180	-0.4761048	-1.6100918
C	0.3237510	2.7573512	-1.4450048
C	0.7636600	2.4118342	0.6865452
C	-0.2178070	2.7576612	-2.7358228
C	1.3095240	3.7174802	-1.0955058
C	0.7671860	1.9730042	2.0153942
C	1.6078550	3.4815522	0.2946252
C	0.2287380	3.7015902	-3.6478028
H	-0.9625950	2.0276792	-3.0181338
C	1.7484610	4.6589952	-2.0276168
C	1.6327010	2.5694712	2.9187852
H	0.0907000	1.1859842	2.3242772
C	2.4650720	4.0814182	1.2200132
C	1.2056460	4.6487992	-3.3040278
H	-0.1871560	3.7046012	-4.6514258
H	2.4998960	5.3937902	-1.7520298
C	2.4855190	3.6143722	2.5270942
H	1.6491900	2.2229892	3.9480452

H	3.1092540	4.9033312	0.9184042
H	1.5344190	5.3748502	-4.0406698
H	3.1572740	4.0605122	3.2530732
N	-0.0155650	1.9671142	-0.3619358
C	3.8052100	-0.6291318	-0.2354248
C	6.1559910	-1.5032228	-0.3315058
C	5.8673200	0.7106392	0.0243542
C	5.9607060	-2.8649698	-0.5472488
C	7.4424960	-0.9255478	-0.2890668
C	5.3139310	1.9718652	0.2314362
C	7.2557090	0.4951202	-0.0619658
C	7.0912140	-3.6526718	-0.7197918
H	4.9627560	-3.2901478	-0.5737838
C	8.5618910	-1.7404378	-0.4616998
C	6.1980680	3.0335722	0.3548622
H	4.2410290	2.1201172	0.3060172
C	8.1226970	1.5824502	0.0660172
C	8.3796160	-3.1001318	-0.6767818
H	6.9731850	-4.7184938	-0.8890268
H	9.5601920	-1.3144178	-0.4290888
C	7.5867490	2.8460622	0.2729042
H	5.7998260	4.0296922	0.5217642
H	9.1972950	1.4403312	0.0008632
H	9.2417430	-3.7449048	-0.8131748
H	8.2482820	3.7005772	0.3732572
N	5.2109980	-0.5081378	-0.1259738
C	3.6045610	-0.9254258	2.2644822
C	1.3349370	-0.3954208	-3.0370568
F	1.8679600	0.6385222	-3.6936248
F	-0.0012250	-0.3171828	-3.1685108
F	1.7087560	-1.5110488	-3.7126258
F	4.4113240	-1.9970628	2.3235452
F	4.3207790	0.1442892	2.6230212
F	2.6579040	-1.0966638	3.2116092

RE2_TS_Cz_R=CF3

Pd	0.9297619	0.2439175	0.5460571
P	0.6907379	-2.0522545	0.2928661
C	-0.1053671	-3.0187285	1.6581621
H	-0.1014071	-4.0838035	1.3898851
C	0.6751959	-2.8557145	2.9703341
H	0.7624769	-1.7882305	3.2036731
H	1.6930019	-3.2443105	2.8476801
C	-0.0265431	-3.5730395	4.1268931
H	-0.0228021	-4.6558205	3.9390741
H	0.5335549	-3.4116795	5.0541651
C	-1.4694151	-3.0884655	4.2855971
H	-1.4645571	-2.0248215	4.5494101

H	-1.9614381	-3.6206345	5.1066041
C	-2.2572371	-3.2739005	2.9867861
H	-2.3526271	-4.3470095	2.7693281
H	-3.2733481	-2.8794965	3.0931761
C	-1.5692991	-2.5768825	1.8110471
H	-1.6099581	-1.4944255	1.9601041
H	-2.1203581	-2.7855555	0.8879061
C	-0.3445361	-2.4462825	-1.2008479
H	-1.2637171	-1.8670175	-1.0267949
C	0.3233579	-1.8756565	-2.4558639
H	0.5716789	-0.8235715	-2.2829059
H	1.2738779	-2.3988965	-2.6242849
C	-0.5650991	-2.0256075	-3.6916779
H	-1.4610641	-1.4104835	-3.5685499
H	-0.0371961	-1.6451835	-4.5733159
C	-0.9810841	-3.4828535	-3.9028219
H	-0.0937621	-4.0844525	-4.1451899
H	-1.6614971	-3.5610195	-4.7569859
C	-1.6443241	-4.0500545	-2.6452649
H	-1.9067801	-5.1027705	-2.7950189
H	-2.5811261	-3.5109625	-2.4532119
C	-0.7270821	-3.9146455	-1.4239189
H	0.1834909	-4.5034015	-1.5953509
H	-1.2221851	-4.3338215	-0.5414919
C	2.2987639	-2.9084485	0.0116531
C	2.3736739	-4.3066295	0.0720541
C	3.5748549	-4.9823965	-0.0995769
H	3.6002999	-6.0660295	-0.0466059
C	4.7392709	-4.2582455	-0.3263599
H	5.6895099	-4.7672935	-0.4518969
C	4.6837799	-2.8718075	-0.3809449
C	3.4803029	-2.1744405	-0.2233979
C	3.5499239	-0.6740515	-0.2799629
C	3.6752479	-0.0092495	-1.5218969
C	3.7437169	-0.7653695	-2.8443609
H	3.3491439	-1.7725365	-2.6806149
C	2.9291339	-0.1253365	-3.9760649
H	1.8837829	0.0259895	-3.7014809
H	2.9501129	-0.7740175	-4.8574789
H	3.3423669	0.8427195	-4.2769749
C	5.2074409	-0.9044115	-3.2942039
H	5.6541599	0.0835315	-3.4496819
H	5.2700179	-1.4581295	-4.2365569
H	5.8121559	-1.4302955	-2.5520139
C	3.8371149	1.3721925	-1.5249259
H	3.9110499	1.8891505	-2.4775019
C	3.8954919	2.1312875	-0.3555029
C	4.0815089	3.6375525	-0.4602749

C	3.9795599	4.3745085	0.8751891
H	4.0230019	5.4544455	0.7078491
H	4.8139059	4.1102345	1.5357501
H	3.0455529	4.1582735	1.3981921
C	3.8017109	1.4563475	0.8559461
H	3.8597409	2.0139155	1.7836291
C	3.6411769	0.0641095	0.9228801
C	3.7608059	-0.6422055	2.2701221
H	3.2795569	-1.6201125	2.1801161
C	5.2443229	-0.8814735	2.5890591
H	5.7296719	-1.4741035	1.8089611
H	5.3525509	-1.4143865	3.5391441
H	5.7753699	0.0731005	2.6708251
C	3.0757319	0.0897345	3.4254421
H	3.5614779	1.0430985	3.6577521
H	3.1162949	-0.5253435	4.3299441
H	2.0262949	0.2856425	3.1975251
H	5.5954439	-2.3038185	-0.5370339
H	1.4755559	-4.8833905	0.2660791
H	3.2767109	4.0051385	-1.1115449
C	5.4214989	3.9674485	-1.1374709
H	6.2561079	3.6151155	-0.5212929
H	5.5116799	3.4990805	-2.1214529
H	5.5292249	5.0486625	-1.2688469
C	-1.5481561	0.9543515	-0.8021769
C	-0.9104341	1.1660135	0.4698691
C	-3.7733331	0.8572045	0.1231511
C	-2.8989131	0.8051615	-0.9766079
H	-0.9378921	0.8836795	-1.6848699
C	-3.2019691	1.0105685	1.3673021
H	-3.8652631	0.9697915	2.2239861
C	-1.8241611	1.1222095	1.5861901
C	0.5110679	2.9197525	-0.8978229
C	0.9279609	3.1964535	-3.2369849
C	0.4783329	4.3139165	-0.7469769
C	0.8385369	4.5851335	-3.0999609
H	1.1398139	2.7602455	-4.2081259
C	0.4706939	3.3209485	1.3199781
C	0.6295109	5.1524465	-1.8487389
H	0.9643279	5.2215655	-3.9696589
C	0.7393919	3.2378955	2.6790661
C	0.4475949	4.5740575	0.6801661
H	0.6118519	6.2307925	-1.7240199
C	0.8695189	4.4201975	3.4030071
H	0.8810049	2.2863215	3.1653951
C	0.5757019	5.7506615	1.4102541
C	0.7660789	5.6675355	2.7847601
H	1.0726309	4.3624925	4.4674321

H	0.5661309	6.7128995	0.9076671
H	0.8763349	6.5730955	3.3721351
C	0.7747279	2.3558605	-2.1409409
H	0.9288289	1.2892285	-2.2430399
N	0.3508099	2.2732645	0.3627631
C	-6.1603441	1.5620445	-0.1929269
C	-7.1994261	3.6649165	-0.5667039
C	-7.4157701	0.9197255	-0.1761529
C	-8.4588391	3.0459235	-0.5552939
H	-7.1340891	4.7374875	-0.7213919
C	-5.7602161	-0.6267295	0.1654661
C	-8.5742091	1.6763775	-0.3613219
H	-9.3510231	3.6462985	-0.7007859
C	-5.1524951	-1.8571265	0.4043201
C	-7.1583651	-0.4885015	0.0544961
H	-9.5496751	1.1992825	-0.3523479
C	-5.9784171	-2.9655315	0.5278651
H	-4.0739771	-1.9352025	0.4868471
C	-7.9675181	-1.6192465	0.1801601
C	-7.3727381	-2.8515905	0.4161151
H	-5.5344471	-3.9389815	0.7135231
H	-9.0467301	-1.5341395	0.0942601
H	-7.9904441	-3.7382285	0.5159471
N	-5.1639001	0.6166975	0.0068241
C	-6.0327051	2.9352995	-0.3863109
H	-5.0562411	3.4078445	-0.3987689
C	-3.4223691	0.5040755	-2.3566959
C	-1.4830551	1.0028215	3.0453641
F	-2.3264861	0.1471685	3.6741321
F	-1.5827231	2.1543065	3.7326711
F	-0.2464081	0.5031805	3.2789451
F	-4.4744671	1.2604005	-2.6846049
F	-3.8046771	-0.7860315	-2.4556989
F	-2.4880681	0.7034995	-3.3085729

7. Optimized Molecular structure From TD-DFT Calculations

10(a)

54

-1.6108290909124628e+03 frame 26 xyz file generated by TeraChem

C	-1.3937323293	-0.1779170034	-0.2823121496
C	-0.7901206688	1.0722034929	-0.3078251585
C	0.6053326303	1.1726262882	-0.2886303642
C	1.3585389727	-0.0101143843	-0.2711282980
C	0.7551608617	-1.2603058340	-0.2979833964
C	-0.6404235243	-1.3607188634	-0.2899818471
N	-1.2814070018	-2.6183898195	-0.2835488274
N	1.2463956672	2.4302877165	-0.2811529324
C	1.1980718922	3.2444634947	0.8639646330
C	1.8066565380	4.5143819906	0.8234764673
O	2.4499765985	4.9737325327	-0.2933505623
C	2.5347675264	4.1589168707	-1.3900586004
C	1.9354746363	2.8843965369	-1.4190476716
C	-1.2397171030	-3.4301763715	0.8636116276
C	-1.8480249757	-4.7002011848	0.8222786290
O	-2.4846568781	-5.1621162164	-0.2973030554
C	-2.5632937608	-4.3495452423	-1.3961312518
C	-1.9637235824	-3.0751556683	-1.4244826898
C	-3.2460837680	-4.8432145055	-2.5011859780
C	-3.3447782607	-4.0815565789	-3.6697964237
C	-2.7458042596	-2.8256812840	-3.7166581464
C	-2.0584266964	-2.3287658624	-2.6041735718
C	-0.6329781889	-3.0297252044	2.0598015627
C	-0.6075121813	-3.8773124912	3.1728541641
C	-1.1988655286	-5.1360820904	3.1121164260
C	-1.8256901530	-5.5403146965	1.9292132853
C	0.5842273079	2.8465141063	2.0573382510
C	0.5522385892	3.6964544454	3.1684294789
C	1.1439417270	4.9550968770	3.1084835465
C	1.7777203869	5.3568739638	1.9284305286
C	3.2238654148	4.6501871806	-2.4922508498
C	3.3293806344	3.8859053686	-3.6585712835
C	2.7309635096	2.6297918563	-3.7060344575
C	2.0372823779	2.1352344566	-2.5963934057
F	-2.7225685439	-0.2644081893	-0.2791246739
F	2.6872572775	0.0764266005	-0.2547185226
H	-1.4080048951	1.9740238199	-0.3240828082
H	1.3731259538	-2.1621719658	-0.3063643806
H	-3.6908766980	-5.8395964914	-2.4233638624
H	-3.8833939045	-4.4774787998	-4.5358377790
H	-2.8040075183	-2.2164654518	-4.6236038289
H	-1.5838831780	-1.3457644725	-2.6609115441

H	-0.1713090895	-2.0413485471	2.1243472333
H	-0.1175424166	-3.5382437983	4.0905884789
H	-1.1806608136	-5.8062429348	3.9766789569
H	-2.3102755371	-6.5168908231	1.8382694554
H	0.1221322708	1.8582777950	2.1211208199
H	0.0568985125	3.3593368193	4.0839961825
H	1.1206372537	5.6270930483	3.9714960897
H	2.2627738472	6.3332942896	1.8382984564
H	3.6681650059	5.6467641971	-2.4140991779
H	3.8729127957	4.2799761701	-4.5223855539
H	2.7945541135	2.0185507568	-4.6112577831
H	1.5633491598	1.1519441085	-2.6534934583

10(b)
54
-2.2565158511924169e+03 frame 92 xyz file generated by TeraChem

C	-1.4052261576	-0.1982505052	-0.3668188394
C	-0.8371390172	1.0697741680	-0.3784307972
C	0.5525164111	1.2103833020	-0.3412906247
C	1.3359383119	0.0501751105	-0.2875395500
C	0.7689640435	-1.2179213108	-0.3064156482
C	-0.6210424525	-1.3590143382	-0.3507884035
N	-1.2022851969	-2.6555191720	-0.3481931568
N	1.1361515224	2.5058854309	-0.3325218769
C	1.4149860392	3.0940289958	0.9224517108
C	1.6610959494	4.4777357890	1.0181886186
S	1.4720525979	5.5086823763	-0.4047067418
C	2.0034121155	4.3732473595	-1.6505313328
C	1.7188038176	2.9990336268	-1.5228399931
C	-1.5125333140	-3.2354735891	0.9034996773
C	-1.7374574320	-4.6221842700	1.0057889774
S	-1.4839196761	-5.6614236568	-0.4008727700
C	-2.0034152932	-4.5472858391	-1.6710301757
C	-1.7460782818	-3.1670179427	-1.5491954579
C	-2.5752740792	-5.0676063203	-2.8345255580
C	-2.8572612327	-4.2355500333	-3.9188673868
C	-2.5722757555	-2.8744840056	-3.8210580089
C	-2.0342107296	-2.3429334462	-2.6475517899
C	-1.6020784966	-2.4606905870	2.0697817088
C	-1.8980728509	-3.0508281559	3.3001129300
C	-2.1415367849	-4.4208756775	3.3882565589
C	-2.0706652269	-5.2006423404	2.2327146013
C	1.4490925494	2.3316052589	2.0997215895
C	1.7133953183	2.9302033193	3.3330914102
C	1.9793046626	4.2962823508	3.4153447660
C	1.9623161416	5.0637839875	2.2497076724
C	2.6128097481	4.8719826633	-2.8042969054
C	2.9060632752	4.0250397054	-3.8740898196

C	2.5954154073	2.6700915421	-3.7709194052
C	2.0204295463	2.1592475990	-2.6056773572
F	-2.7329722037	-0.3188253118	-0.3771428184
F	2.6615166273	0.1734810148	-0.2137225681
H	-1.4760954768	1.9559743012	-0.4064344633
H	1.4078949744	-2.1040643251	-0.2762722254
H	-2.7794322824	-6.1420862829	-2.8879519473
H	-3.2922540118	-4.6526066833	-4.8320458957
H	-2.7807279410	-2.2038489738	-4.6605097214
H	-1.8315255739	-1.2711616921	-2.5973927044
H	-1.4276803318	-1.3830449932	2.0221369537
H	-1.9494841552	-2.4215299868	4.1941689443
H	-2.3850140551	-4.8847601821	4.3488206471
H	-2.2566116487	-6.2786779706	2.2754747458
H	1.2548955268	1.2571660696	2.0588817036
H	1.7213642095	2.3100477444	4.2347755640
H	2.1977908697	4.7667514089	4.3786393585
H	2.1655285113	6.1388709060	2.2871683846
H	2.8372479427	5.9420983780	-2.8624092760
H	3.3695475107	4.4261615188	-4.7802892070
H	2.8113930908	1.9883832516	-4.5994558334
H	1.7964337287	1.0918500263	-2.5503476530

10(c)
70
-1.6961064655108596e+03 frame 30 xyz file generated by TeraChem

C	-1.3695213412	-0.1853752843	-0.3641796006
C	-0.7911483314	1.0781753972	-0.3724679370
C	0.6011786386	1.2022938150	-0.3636385437
C	1.3784447172	0.0369830701	-0.3614366118
C	0.8000902670	-1.2265584981	-0.3714484432
C	-0.5922527194	-1.3506894829	-0.3653553874
N	-1.2178195650	-2.6226553497	-0.3643267037
N	1.2268213201	2.4742254050	-0.3609958985
C	1.4258409396	3.1331026612	0.8599356526
C	2.0422380906	4.4006840020	0.8988417328
C	2.5482782930	5.1137680653	-0.3550595836
C	2.3076268373	4.2730373542	-1.6083946252
C	1.6741041992	3.0141391851	-1.5744586150
C	-1.4177761204	-3.2826326537	0.8558770075
C	-2.0334261815	-4.5506274446	0.8930734372
C	-2.5375153140	-5.2630823819	-0.3619729294
C	-2.2964679522	-4.4209713862	-1.6143097869
C	-1.6637189217	-3.1617355065	-1.5786656174
C	-2.7088248865	-4.9127739023	-2.8603915094
C	-2.5195553917	-4.2076296700	-4.0473428061
C	-1.8921194814	-2.9628786337	-3.9970042513
C	-1.4669882353	-2.4471676101	-2.7772085733

C	-0.9991732639	-2.6767489516	2.0575662539
C	-1.1632706857	-3.3199078969	3.2798292751
C	-1.7549361135	-4.5820994388	3.3298348855
C	-2.1819669436	-5.1711049757	2.1411782433
C	1.0054476203	2.5265369191	2.0606517543
C	1.1685891902	3.1685898768	3.2836206939
C	1.7610406035	4.4303478766	3.3353157867
C	2.1897753285	5.0200494204	2.1476169124
C	2.7213876218	4.7657288749	-2.8536591591
C	2.5327552362	4.0617873686	-4.0414238811
C	1.9045452795	2.8173603794	-3.9927788329
C	1.4780423070	2.3007737254	-2.7738276364
C	-1.7953378480	-6.6080746401	-0.5094139713
C	-4.0513134355	-5.5278271860	-0.2161976033
C	1.8076561248	6.4596758953	-0.5019724693
C	4.0622122071	5.3768064897	-0.2075629262
F	-2.6961887558	-0.3016305674	-0.3739739729
F	2.7051260342	0.1532456068	-0.3678034490
H	-1.4237129318	1.9697133178	-0.3809398905
H	1.4326680038	-2.1180943587	-0.3790388659
H	-3.1959521726	-5.8921426566	-2.9036842257
H	-2.8555880455	-4.6281850845	-4.9999201861
H	-1.7263945867	-2.3838883117	-4.9115026435
H	-0.9721636695	-1.4735388809	-2.7535044082
H	-0.5364192626	-1.6876168040	2.0321140761
H	-0.8233290184	-2.8263419679	4.1962437109
H	-1.8854496114	-5.1030005885	4.2831771722
H	-2.6510483002	-6.1592878032	2.1845182123
H	0.5420110072	1.5377589260	2.0338325787
H	0.8272763007	2.6745157805	4.1992502898
H	1.8908371881	4.9503813355	4.2892286858
H	2.6594937351	6.0078709796	2.1922880579
H	3.2091768369	5.7448275722	-2.8956230521
H	2.8698884403	4.4830078567	-4.9933186239
H	1.7392881512	2.2393064366	-4.9079543366
H	0.9826533898	1.3274009453	-2.7514595431
H	-0.7090992257	-6.4487755201	-0.6077988858
H	-2.1433107151	-7.1567835334	-1.4001382869
H	-1.9702795102	-7.2524273253	0.3676690421
H	-4.6063307628	-4.5822576117	-0.1038275340
H	-4.2588753940	-6.1551476202	0.6659879659
H	-4.4465282008	-6.0561131808	-1.0991640829
H	0.7213323112	6.3016252354	-0.6014327135
H	2.1569761732	7.0088156850	-1.3919019804
H	1.9825424067	7.1030685368	0.3758196916
H	4.6161471322	4.4305562224	-0.0955802132
H	4.2695388815	6.0030483685	0.6754462847
H	4.4588020633	5.9055440567	-1.0896464887

11(a)
60
-2.0856624889119998e+03 frame 19 xyz file generated by TeraChem

C	-1.4177719348	-0.0493595078	-0.2924198240
C	-0.6914572693	1.1413934685	-0.2883278134
C	0.7055446168	1.1338480236	-0.2849322846
C	1.3848110808	-0.0948765494	-0.2859590664
C	0.6585225434	-1.2856469361	-0.2924012101
C	-0.7384871371	-1.2781291380	-0.2955677461
N	-1.4450037123	-2.5097932743	-0.3008456211
N	1.4121929727	2.3654319918	-0.2761537398
C	1.6109867148	3.0439554922	0.9407847864
C	2.1799927260	4.3314530880	0.9165469781
O	2.5317332359	4.9429515630	-0.2563872631
C	2.1948840123	4.3428011550	-1.4395521184
C	1.6265438741	3.0554834698	-1.4839628707
C	-1.6557385845	-3.1971138296	0.9091317024
C	-2.2249564588	-4.4841582125	0.8698098999
O	-2.5636857774	-5.0879812208	-0.3109179543
C	-2.2182862348	-4.4778247226	-1.4864573593
C	-1.6501693707	-3.1899760846	-1.5157589336
C	-2.4593521423	-5.1770034905	-2.6629473123
C	-2.1401875946	-4.6079309407	-3.9001064860
C	-1.5754933765	-3.3360190347	-3.9427233687
C	-1.3317694048	-2.6326956001	-2.7581721382
C	-1.3425946150	-2.6471215737	2.1560864192
C	-1.5943275759	-3.3560382845	3.3355690237
C	-2.1615109669	-4.6265002210	3.2831817547
C	-2.4741067931	-5.1889179562	2.0413050473
C	1.2856258098	2.4846993955	2.1804874730
C	1.5241905939	3.1855159976	3.3674889269
C	2.0904431434	4.4569108966	3.3300092255
C	2.4159300632	5.0282439234	2.0955068545
C	2.4454339262	5.0516401039	-2.6082557181
C	2.1358501188	4.4929803143	-3.8525477431
C	1.5708078229	3.2217947989	-3.9101227953
C	1.3173661870	2.5088006529	-2.7333800760
C	-2.9307384866	0.0013792804	-0.2898904934
F	-3.4516495701	-0.6011267292	-1.3662138664
F	-3.4468031161	-0.5978854732	0.7906905177
F	-3.3763002624	1.2647205450	-0.2905451826
C	2.8977534699	-0.1453478218	-0.2780474673
F	3.4218109644	0.4580477932	-1.3524917871
F	3.4103292238	0.4532318553	0.8044214074
F	3.3435375204	-1.4086121882	-0.2783542177
H	-1.2103006692	2.1031644499	-0.2859653426
H	1.1773845598	-2.2474100119	-0.2937255835

H	-2.9043670442	-6.1733681518	-2.5845743897
H	-2.3374581181	-5.1628234284	-4.8218610334
H	-1.3199240717	-2.8725453539	-4.9003991222
H	-0.8963586881	-1.6310970877	-2.8019414400
H	-0.9057194512	-1.6465124648	2.2073941421
H	-1.3434163380	-2.8978186214	4.2969935711
H	-2.3658079274	-5.1853656507	4.2009933858
H	-2.9202166661	-6.1841756267	1.9552606496
H	0.8493695582	1.4832902161	2.2201017385
H	1.2635741961	2.7203178093	4.3229575797
H	2.2841920495	5.0096215366	4.2538149064
H	2.8621104638	6.0244240438	2.0213018738
H	2.8903086263	6.0470597770	-2.5180064955
H	2.3406776522	5.0553393470	-4.7681099672
H	1.3225257707	2.7664511847	-4.8736003935
H	0.8815453474	1.5079626847	-2.7889313312

11(b)
60
-2.7313478592921324e+03 frame 77 xyz file generated by TeraChem

C	-1.4247232005	-0.0439557290	-0.2643600478
C	-0.6855694881	1.1387321269	-0.2653780093
C	0.7120301300	1.1164728747	-0.2683058615
C	1.3758119839	-0.1204498975	-0.2701794987
C	0.6367789644	-1.3031224920	-0.2688324305
C	-0.7608732305	-1.2810320121	-0.2656717577
N	-1.5118283135	-2.4888970516	-0.2655199525
N	1.4638151441	2.3237107826	-0.2717981563
C	1.6639128626	2.9986823826	0.9571789971
C	2.6939487884	3.9528049905	1.0654027345
S	3.8174653821	4.1842216685	-0.2789824700
C	2.6898803827	3.9472566105	-1.6190375290
C	1.6625762359	2.9908297040	-1.5050814025
C	-1.7057800678	-3.1640653439	0.9645297021
C	-2.7349854767	-4.1185959652	1.0771361983
S	-3.8633092390	-4.3511845392	-0.2630179750
C	-2.7405774185	-4.1148970913	-1.6072881590
C	-1.7128776606	-3.1584683301	-1.4972596738
C	-2.9420963420	-4.8083596568	-2.8025926636
C	-2.1588913457	-4.5288696843	-3.9238424450
C	-1.1592321562	-3.5617693326	-3.8319216980
C	-0.9272642416	-2.8926830339	-2.6280504923
C	-0.9107646962	-2.9068032426	2.0907618377
C	-1.1365584256	-3.5804358381	3.2933011873
C	-2.1403977540	-4.5429111517	3.3885281941
C	-2.9320119170	-4.8150924447	2.2714472774
C	0.8745606938	2.7405620720	2.0871942596
C	1.1056908102	3.4141199203	3.2887237526

C	2.1092759735	4.3772963174	3.3793167992
C	2.8959839242	4.6494335508	2.2587614763
C	2.8874865593	4.6399939532	-2.8153816393
C	2.1012081900	4.3592958218	-3.9341753859
C	1.1032102061	3.3907968252	-3.8389631479
C	0.8749283844	2.7225256010	-2.6339010055
C	2.8893591401	-0.1845938803	-0.2729148175
F	3.4112047671	0.4101881846	-1.3523587799
F	3.4138295477	0.4092024546	0.8058577834
F	3.3239423407	-1.4531529586	-0.2742298704
C	-2.9383435889	0.0212439393	-0.2657923489
F	-3.4596232576	-0.5680868724	-1.3485443713
F	-3.4642811093	-0.5771221225	0.8096450314
F	-3.3721028303	1.2901159644	-0.2613230591
H	-1.1980475280	2.1039630076	-0.2645521053
H	1.1492418671	-2.2683642635	-0.2710034324
H	-3.7383638286	-5.5581805995	-2.8509281271
H	-2.3351279376	-5.0631139861	-4.8621852269
H	-0.5390984468	-3.3235693246	-4.7017945051
H	-0.1328755738	-2.1446956653	-2.5799662490
H	-0.1132639518	-2.1623109232	2.0401810740
H	-0.5082777015	-3.3495996507	4.1593402266
H	-2.3128189313	-5.0797919128	4.3260710473
H	-3.7310430733	-5.5618424723	2.3222389181
H	0.0775935019	1.9952366587	2.0403362907
H	0.4817522938	3.1826648610	4.1577023073
H	2.2855466565	4.9145060585	4.3159536014
H	3.6952408539	5.3961465600	2.3059792124
H	3.6832429963	5.3901756078	-2.8663965874
H	2.2739254535	4.8935936140	-4.8731486131
H	0.4813320094	3.1509896791	-4.7071475648
H	0.0817520184	1.9733558650	-2.5833628545

11(c)
76
-2.170943123988618e+03 frame 326 xyz file generated by TeraChem

C	-1.4222246175	-0.0755638664	-0.3978073599
C	-0.7389853561	1.1401816130	-0.3932748316
C	0.6572917740	1.1847927050	-0.3868557272
C	1.3777320779	-0.0178451735	-0.3874515220
C	0.6947463499	-1.2336650960	-0.3918139501
C	-0.7017831327	-1.2782926141	-0.3960246760
N	-1.3661609880	-2.5382060502	-0.3940715027
N	1.3225055053	2.4438220393	-0.3790411918
C	1.4075850114	3.1395482785	0.8442710304
C	2.0175330346	4.4125120249	0.8750082586
C	2.7554868254	4.9167545160	-0.3621645969
C	2.0214547948	4.4270146975	-1.6073911984

C	1.4127317462	3.1530853336	-1.5941030567
C	-1.4491234049	-3.2385497002	0.8272537947
C	-2.0510225658	-4.5156686359	0.8519989310
C	-2.7870244255	-5.0159153884	-0.3879893333
C	-2.0458895960	-4.5249980047	-1.6285270876
C	-1.4442162323	-3.2475972322	-1.6103962434
C	-2.0336351346	-5.2409945520	-2.8309546689
C	-1.4923594428	-4.7125190570	-4.0041663763
C	-0.9545727467	-3.4270395161	-3.9845907462
C	-0.9251270147	-2.7006311266	-2.7962566992
C	-0.9338202945	-2.6836368429	2.0111769502
C	-0.9655201610	-3.4024569237	3.2040040485
C	-1.5029310064	-4.6879336423	3.2301138247
C	-2.0419430087	-5.2236573752	2.0591425174
C	0.8879476436	2.5821468790	2.0252191852
C	0.9252186166	3.2941730299	3.2218817463
C	1.4738562422	4.5747798359	3.2551003719
C	2.0157467475	5.1129020640	2.0866479234
C	2.0238857070	5.1417071242	-2.8105807988
C	1.4895491927	4.6155803188	-3.9879775566
C	0.9431897231	3.3336687198	-3.9723649254
C	0.9001023769	2.6083280420	-2.7839260778
C	-2.9551476549	-6.5372933922	-0.3825507404
C	-4.1933202843	-4.3687552175	-0.3944356187
C	2.9172632929	6.4387626332	-0.3524486593
C	4.1640091474	4.2751828168	-0.3648009219
C	-2.9345661010	-0.0783212207	-0.4050035905
F	-3.4280290726	-0.6936138407	-1.4883915772
F	-3.4400767116	-0.6982812208	0.6703670526
F	-3.4228323758	1.1697728253	-0.4050134436
C	2.8900535868	-0.0138787140	-0.3830924233
F	3.3927947839	0.6060633494	-1.4597837610
F	3.3860017494	0.6018676749	0.6989151002
F	3.3790712078	-1.2615398179	-0.3841706141
H	-1.2929557779	2.0818555227	-0.3936862354
H	1.2490254315	-2.1753288646	-0.3902381770
H	-2.4789360164	-6.2384019834	-2.8629386505
H	-1.5074822366	-5.2986521055	-4.9281928441
H	-0.5431858623	-2.9814610952	-4.8959376774
H	-0.4886440557	-1.6993213538	-2.7943959217
H	-0.4981550500	-1.6820708660	2.0042658243
H	-0.5560490310	-2.9510563533	4.1134510951
H	-1.5195797178	-5.2682557544	4.1576531315
H	-2.4878346621	-6.2205779203	2.0962783867
H	0.4453808806	1.5835500570	2.0129338824
H	0.5118289930	2.8412956243	4.1288464613
H	1.4961423419	5.1496044980	4.1859788598
H	2.4706171569	6.1054340001	2.1298075531

H	2.4758283485	6.1362109919	-2.8401830765
H	1.5161766726	5.2006534696	-4.9123100379
H	0.5358999435	2.8898687145	-4.8865549763
H	0.4588816914	1.6091226654	-2.7852183128
H	-1.9833936629	-7.0574724403	-0.3803442097
H	-3.5272697188	-6.8706301957	-1.2616086383
H	-3.5284494099	-6.8643198972	0.4981564554
H	-4.1281930269	-3.2701393211	-0.4029642694
H	-4.7575615984	-4.6722265840	0.5036559245
H	-4.7551910887	-4.6863489980	-1.2891683876
H	1.9432862976	6.9546087045	-0.3511454250
H	3.4903316290	6.7772499787	-1.2286763340
H	3.4869780688	6.7657605982	0.5303615420
H	4.1028153146	3.1763509656	-0.3727870728
H	4.7246263160	4.5817052635	0.5344522029
H	4.7267623086	4.5947835678	-1.2581919961

12(a)
57
-1.7491754976142097e+03 frame 47 xyz file generated by TeraChem

C	-1.3195538893	-0.1363068050	-0.3864904468
C	-0.6232401485	1.0710087974	-0.3586869353
C	0.7741043020	1.0907714901	-0.3039333866
C	1.4796154925	-0.1256024894	-0.2960358564
C	0.7808038519	-1.3336067841	-0.3352111232
C	-0.6168399077	-1.3447677341	-0.3697875663
N	-1.3157553801	-2.5832912408	-0.3779799143
N	1.4538166252	2.3397358583	-0.2316348854
C	1.5670272644	2.9879832298	1.0119502170
C	2.0538594606	4.3090302088	1.0546390701
O	2.4153729668	4.9815770126	-0.0815571403
C	2.1838856284	4.3937222028	-1.2960032698
C	1.6962114368	3.0768273200	-1.4048770258
C	-1.5420326596	-3.2746221632	0.8224476206
C	-2.2260746353	-4.5068402289	0.7836914393
O	-2.6841554945	-5.0411305410	-0.3905611810
C	-2.4221743055	-4.3847692130	-1.5633673668
C	-1.7363995844	-3.1532468026	-1.5888069231
C	-2.8564439245	-4.9858970029	-2.7379212164
C	-2.6175541623	-4.3754140533	-3.9741882777
C	-1.9362220425	-3.1623082187	-4.0152974192
C	-1.4972905279	-2.5569030977	-2.8319398169
C	-1.1272112905	-2.7903953548	2.0684125045
C	-1.3766621845	-3.5143234534	3.2403600810
C	-2.0470335884	-4.7328616443	3.1854403509
C	-2.4725414993	-5.2255110274	1.9467576379
C	1.2259000939	2.3692983769	2.2193333193
C	1.3683630908	3.0433539830	3.4365183681

C	1.8517226535	4.3486886554	3.4643747301
C	2.1919344662	4.9801659221	2.2637276076
C	2.4586507853	5.1497622118	-2.4292605201
C	2.2548366688	4.6110190138	-3.7034715300
C	1.7670130832	3.3127156094	-3.8256472734
C	1.4882767346	2.5523622267	-2.6848499176
C	2.9902446354	-0.1463966400	-0.2384673788
F	3.5414298909	0.4832742971	-1.2859586491
F	3.4591149028	0.4425372112	0.8697292866
F	3.4626687111	-1.4015093790	-0.2440292896
H	-2.4134535408	-0.1503356323	-0.4149764591
H	-1.1606720471	2.0240450317	-0.3645039046
H	1.3220704493	-2.2825738358	-0.3298956934
H	-3.3825417659	-5.9421363056	-2.6595371861
H	-2.9623790469	-4.8551040377	-4.8948890510
H	-1.7347932782	-2.6710335318	-4.9722452785
H	-0.9603587464	-1.6058724666	-2.8762964864
H	-0.6030582770	-1.8329651938	2.1248534086
H	-1.0386959104	-3.1094143119	4.1992738434
H	-2.2440478078	-5.3047540265	4.0970084445
H	-3.0064714310	-6.1764127228	1.8577142648
H	0.8543123704	1.3414741155	2.2079328233
H	1.0978434211	2.5313360358	4.3647634846
H	1.9676148695	4.8815776990	4.4124595389
H	2.5725772831	6.0056053297	2.2404471215
H	2.8367173644	6.1664517558	-2.2870022858
H	2.4782770449	5.2102027161	-4.5908390457
H	1.5999237757	2.8737099049	-4.8137235861
H	1.1130661531	1.5313567010	-2.7907509448

12(b)
57
-2.3948605585723863e+03 frame 80   xyz file generated by TeraChem

C	-1.4219546689	-0.0904954025	-0.2508411004
C	-0.7202937581	1.1142219368	-0.2480344916
C	0.6779057336	1.1277863395	-0.2615003559
C	1.3756670474	-0.0930885062	-0.2842124274
C	0.6708258968	-1.2969864026	-0.2959164001
C	-0.7280388368	-1.3016333832	-0.2746749484
N	-1.4612390741	-2.5263762197	-0.2924587515
N	1.4000167158	2.3565663497	-0.2529145717
C	1.5678427874	3.0298896967	0.9806468947
C	2.5463010284	4.0363711112	1.1007167571
S	3.6656273298	4.3358860737	-0.2332950381
C	2.5672918441	4.0393943045	-1.5853022139
C	1.5870471866	3.0330869777	-1.4817498462
C	-1.5723495143	-3.2625243405	0.9093534840
C	-2.5121696313	-4.3084046116	1.0160211613

S	-3.6742841919	-4.5926998234	-0.2842120541
C	-2.6619619584	-4.1597211359	-1.6662062068
C	-1.7148246475	-3.1221424741	-1.5485644265
C	-2.8882836938	-4.7834424644	-2.8949585948
C	-2.2212472880	-4.3540758840	-4.0436332424
C	-1.3099464203	-3.3044586121	-3.9446973526
C	-1.0468716266	-2.7046553390	-2.7111932747
C	-0.7716508185	-2.9824454217	2.0286102540
C	-0.9009930222	-3.7193527864	3.2079758412
C	-1.8134214338	-4.7690593884	3.2930480139
C	-2.6112300469	-5.0638568123	2.1864711948
C	0.7938614242	2.7202376612	2.1091403364
C	0.9867369922	3.3946823755	3.3169369329
C	1.9359821744	4.4103808514	3.4177285398
C	2.7075734412	4.7336165467	2.3001303750
C	2.7467953798	4.7381319736	-2.7811619105
C	1.9932956665	4.4149326556	-3.9111148556
C	1.0428019450	3.3988223613	-3.8262980792
C	0.8300781915	2.7242955678	-2.6218652077
C	2.8883637476	-0.1202694798	-0.3004328978
F	3.3910022695	0.4798475502	-1.3875987209
F	3.4143132376	0.4895612680	0.7701415671
F	3.3535530454	-1.3795062585	-0.2999591409
H	-2.5153301621	-0.1015627489	-0.2373608164
H	-1.2579230637	2.0668112445	-0.2331373009
H	1.2108793940	-2.2466655223	-0.3214380340
H	-3.6142695925	-5.6013238667	-2.9453484900
H	-2.4189690485	-4.8359082032	-5.0056841500
H	-0.7813187135	-2.9455446902	-4.8332150978
H	-0.3181801258	-1.8927498151	-2.6584608865
H	-0.0417612203	-2.1713352545	1.9881552674
H	-0.2670916437	-3.4666604114	4.0635485564
H	-1.9111494437	-5.3543740986	4.2120775610
H	-3.3414881891	-5.8784772478	2.2276648279
H	0.0377116365	1.9341952547	2.0534763701
H	0.3758866926	3.1204083966	4.1830845747
H	2.0827545845	4.9473107109	4.3597472485
H	3.4653087478	5.5218660990	2.3556852511
H	3.5046971897	5.5269973743	-2.8240937611
H	2.1550788906	4.9523831863	-4.8503757315
H	0.4469542385	3.1246124452	-4.7028288576
H	0.0729179623	1.9382768006	-2.5767825203

12(c)
73
-1.8344519680977612e+03 frame 31 xyz file generated by TeraChem

C	-1.3224656845	-0.1231967997	-0.3557851112
C	-0.6408196435	1.0921227861	-0.3546021173

C	0.7570018130	1.1314720186	-0.3756174373
C	1.4783919102	-0.0751361684	-0.4057753853
C	0.7920063349	-1.2910206177	-0.4221552467
C	-0.6042591624	-1.3214433528	-0.3922989274
N	-1.2821182568	-2.5742290204	-0.3933849558
N	1.4180596822	2.3937415863	-0.3540016351
C	1.6139139576	3.0281856961	0.8826860712
C	2.1736794143	4.3202982976	0.9453135731
C	2.6136531694	5.0893994389	-0.2996359677
C	2.1861782430	4.3652285657	-1.5757742214
C	1.6240035386	3.0725384468	-1.5654630700
C	-1.4696830248	-3.2455489803	0.8214841328
C	-2.0946294384	-4.5097477707	0.8559791303
C	-2.5868344916	-5.2226396662	-0.4036797264
C	-2.3920450176	-4.3527291260	-1.6457668105
C	-1.7426348441	-3.1008230213	-1.6066649537
C	-2.8397981031	-4.8207725308	-2.8886213207
C	-2.6636861526	-4.1040298714	-4.0710277579
C	-2.0084398514	-2.8739282823	-4.0200148715
C	-1.5519041022	-2.3789494320	-2.8029501694
C	-1.0354504674	-2.6522164633	2.0249226302
C	-1.1979182059	-3.3009496602	3.2444093425
C	-1.8047653152	-4.5559155968	3.2914277030
C	-2.2432381586	-5.1344992187	2.1016785209
C	1.2595511592	2.3647988165	2.0745489190
C	1.4569959079	2.9625476960	3.3142769385
C	2.0165453041	4.2378924424	3.3917193463
C	2.3650156383	4.8910837591	2.2111828137
C	2.3903164993	4.9810460655	-2.8183258510
C	2.0518634613	4.3713957547	-4.0247482516
C	1.4874162409	3.0960551979	-3.9986541050
C	1.2764009420	2.4542918741	-2.7831611739
C	-1.7793836523	-6.5263857668	-0.5828661930
C	-4.0833638257	-5.5605941747	-0.2440075202
C	1.9889716776	6.4994132788	-0.2781127572
C	4.1532212623	5.2072713792	-0.2891445128
C	2.9909521937	-0.0866243048	-0.4165752306
F	3.4975196989	0.5243621930	-1.4970922604
F	3.5092665941	0.5241644236	0.6587941736
F	3.4692950247	-1.3402246726	-0.4193914003
H	-2.4162710328	-0.1507914008	-0.3297315340
H	-1.1892759234	2.0385713617	-0.3281525429
H	1.3432794291	-2.2335395181	-0.4481582964
H	-3.3422216903	-5.7922449695	-2.9341260683
H	-3.0282724989	-4.5065286420	-5.0207481619
H	-1.8463468160	-2.2889615257	-4.9314339054
H	-1.0387468641	-1.4152606535	-2.7791769976
H	-0.5625725542	-1.6680026407	2.0035585630

H	-0.8456396697	-2.8163426794	4.1609605178
H	-1.9365760276	-5.0806020472	4.2424018764
H	-2.7194688324	-6.1193325246	2.1419753806
H	0.8314327103	1.3611166264	2.0293520465
H	1.1726935125	2.4214295137	4.2227081166
H	2.1857426964	4.7189339134	4.3597141191
H	2.8118031646	5.8881451427	2.2740501291
H	2.8384886729	5.9792723810	-2.8418714678
H	2.2319598320	4.8864922232	-4.9731170692
H	1.2098490832	2.5896621482	-4.9289606234
H	0.8442177286	1.4511567639	-2.7776625192
H	-0.7050503101	-6.3076984225	-0.6970723409
H	-2.1132269899	-7.0784177670	-1.4771749162
H	-1.9025595597	-7.1904177039	0.2888504108
H	-4.6808341894	-4.6434167363	-0.1149380605
H	-4.2518328638	-6.2058757895	0.6329714386
H	-4.4657270163	-6.0990822344	-1.1259617252
H	0.8883737221	6.4406258233	-0.2842462927
H	2.3067118711	7.0887677931	-1.1525236159
H	2.2982716969	7.0588978301	0.6187989394
H	4.6210290913	4.2095535078	-0.3048719850
H	4.4988223569	5.7350045758	0.6153541715
H	4.5089848488	5.7683978783	-1.1692402024

13(a)
52
-1.2624851490845679e+03 frame 1   xyz file generated by TeraChem

C	-0.7503657546	1.1475057633	-0.3716617166
C	-1.3843598429	-0.0928159049	-0.3530689198
C	-0.6293181732	-1.2738306169	-0.3538123864
C	0.7702557324	-1.1920842490	-0.3614219948
C	1.4040935026	0.0482514577	-0.3426100585
C	0.6491147149	1.2293001804	-0.3536124494
C	4.0798053337	4.2937339011	-2.8332965065
C	3.6449144929	3.0043657403	-3.1888522966
C	2.7133665548	2.3113341717	-2.4171449135
C	2.2252626741	2.9401579351	-1.2665099149
C	2.6428401874	4.2463573592	-0.9016907530
C	3.5795941639	4.9205802097	-1.6948918100
N	1.2888233207	2.4883156926	-0.3389614036
C	1.0988747100	3.4816718240	0.6198907988
C	1.9236666992	4.5921366358	0.3043151307
C	0.2891427982	3.4725030090	1.7606036899
C	0.3011129725	4.6053416668	2.5729597819
C	1.1006457379	5.7207888273	2.2658139386
C	1.9158498536	5.7184373407	1.1368534703
C	-4.0334905448	-4.3526907889	-2.8525348149
C	-3.5988052832	-3.0632366501	-3.2080517172

C	-2.6756724779	-2.3656251538	-2.4303593259
C	-2.1959824560	-2.9898593755	-1.2737146282
C	-2.6135555392	-4.2960098846	-0.9086337338
C	-3.5416529831	-4.9749353025	-1.7079828724
N	-1.2687242149	-2.5330412938	-0.3394057987
C	-1.0849871814	-3.5229346310	0.6241803743
C	-1.9041740806	-4.6364153819	0.3047014054
C	-0.2855446350	-3.5081560811	1.7720848585
C	-0.3019319112	-4.6385070907	2.5878117078
C	-1.0957025277	-5.7570187001	2.2768628170
C	-1.9007993140	-5.7602122067	1.1406655039
H	-1.3424957354	2.0669822212	-0.3939558869
H	-2.4765269051	-0.1521990578	-0.3379764662
H	1.3624306551	-2.1116886251	-0.3756095229
H	2.4961114898	0.1077010800	-0.3192997406
H	4.8139252206	4.8081703439	-3.4609178719
H	4.0434607926	2.5325287068	-4.0928456558
H	2.3745808674	1.3132309523	-2.7080478998
H	3.9113942069	5.9276244302	-1.4225709341
H	-0.3289288545	2.6066038817	2.0128589420
H	-0.3251240421	4.6229315270	3.4708412031
H	1.0838773067	6.5946438218	2.9243189082
H	2.5456662505	6.5827703779	0.9030454442
H	-4.7608664187	-4.8707749342	-3.4849885075
H	-3.9908129604	-2.5949968753	-4.1167638726
H	-2.3369559291	-1.3674900942	-2.7212272057
H	-3.8732344218	-5.9820418743	-1.4356140153
H	0.3279816495	-2.6398550670	2.0271669758
H	0.3161229801	-4.6516741824	3.4914183669
H	-1.0825007604	-6.6288402862	2.9381382322
H	-2.5261526389	-6.6269666419	0.9038676914

13(b)

100

-1.8902880940094774e+03 frame 1 xyz file generated by TeraChem

C	-0.7253160965	1.1492712561	-0.4497035236
C	-1.3612327077	-0.0887740016	-0.4166085678
C	-0.6156877309	-1.2772971595	-0.4291581733
C	0.7847895121	-1.1901532329	-0.4359113034
C	1.4204733747	0.0480310880	-0.4032316302
C	0.6750938636	1.2365304480	-0.4295784823
C	4.5966683604	4.0476289784	-2.5577772861
C	4.2024062594	2.7194121498	-2.8220378218
C	3.1248686047	2.1061439711	-2.1804104626
C	2.4174667225	2.8409902769	-1.2262436270
C	2.7655532367	4.1890304734	-0.9640381810
C	3.8448022044	4.7767905974	-1.6276542991
N	1.3169690237	2.4898464534	-0.4389435151

C	0.9557463463	3.6028286147	0.3257292071
C	1.8251539267	4.6790270897	0.0206002695
C	-0.0383116031	3.7563462388	1.2941565574
C	-0.1757850345	5.0022266530	1.9093239573
C	0.6496797639	6.1037751265	1.6024331684
C	1.6640101321	5.9126029813	0.6554412611
C	-4.5175060602	-4.1008282680	-2.5777181399
C	-4.1206476328	-2.7742321948	-2.8462202541
C	-3.0488614964	-2.1573155351	-2.1984749173
C	-2.3503827188	-2.8865814877	-1.2334071272
C	-2.7011070450	-4.2330351584	-0.9664759280
C	-3.7742415522	-4.8246879807	-1.6366333492
N	-1.2574621575	-2.5309679336	-0.4377032608
C	-0.9037004081	-3.6392431164	0.3370671254
C	-1.7704175359	-4.7172232404	0.0302139961
C	0.0809413454	-3.7871463283	1.3160509209
C	0.2119521499	-5.0292188964	1.9402884708
C	-0.6110367822	-6.1322720389	1.6323688165
C	-1.6160257934	-5.9468157090	0.6743914698
C	5.7876136683	4.7116753296	-3.2635949765
C	0.4763431155	7.4789869177	2.2639541972
C	-5.7020179757	-4.7686732873	-3.2907613202
C	-0.4452385257	-7.5030269415	2.3049773836
C	5.2657149491	5.8032724319	-4.2150277526
C	6.7252130492	5.3477184441	-2.2215553612
C	6.6084521463	3.7087950967	-4.0825010781
C	1.7546131659	7.8394148065	3.0417754920
C	0.2234004530	8.5378556376	1.1749648757
C	-0.7030610179	7.5069329801	3.2422140341
C	-1.7293811319	-7.8545334718	3.0771295352
C	-0.1878712018	-8.5707109950	1.2256638747
C	0.7280062149	-7.5268948288	3.2906137082
C	-5.1706901657	-5.8576250373	-4.2400134016
C	-6.6435870729	-5.4089474198	-2.2549636496
C	-6.5217388880	-3.7679534000	-4.1134515932
H	-1.3237893428	2.0628959655	-0.4888913355
H	-2.4528378286	-0.1351436151	-0.3870487461
H	1.3834922921	-2.1041972447	-0.4640466206
H	2.5116132481	0.0945420138	-0.3631686087
H	4.7528630024	2.1284402615	-3.5578630754
H	2.8521605167	1.0789793231	-2.4335131107
H	4.0989120791	5.8187215986	-1.4080402040
H	-0.6945135835	2.9311562964	1.5806403084
H	-0.9636251168	5.1087271032	2.6585926348
H	2.3433979307	6.7320311272	0.3982308785
H	-4.6643482329	-2.1873981310	-3.5902929169
H	-2.7735331552	-1.1316680750	-2.4550810062
H	-4.0304547951	-5.8651739698	-1.4131413219

H	0.7347009977	-2.9603080234	1.6036035566
H	0.9925352489	-5.1311565819	2.6976908228
H	-2.2933887267	-6.7674384760	0.4158237681
H	4.6920382622	6.5736095446	-3.6732761857
H	4.6018253911	5.3714285894	-4.9826894400
H	6.1046414205	6.3048741138	-4.7289783580
H	6.2253586153	6.1397123572	-1.6410104711
H	7.5973333852	5.8058299261	-2.7197040954
H	7.0954937450	4.5917576310	-1.5089938658
H	6.0217071848	3.2618825253	-4.9021558142
H	7.0011460672	2.8920320417	-3.4535370634
H	7.4709146159	4.2218865027	-4.5398314932
H	2.6393581521	7.8742111878	2.3851780125
H	1.9552414912	7.0987355425	3.8340321554
H	1.6515591853	8.8303742678	3.5176365650
H	1.0590136058	8.5928413958	0.4588905143
H	0.1022365729	9.5382057931	1.6262014423
H	-0.6918849028	8.3052040391	0.6053254521
H	-0.5655633190	6.7925810985	4.0709651804
H	-1.6579234926	7.2757159830	2.7411377514
H	-0.7954951989	8.5128184137	3.6849881787
H	-2.6100673726	-7.8906670785	2.4151657513
H	-1.9326963903	-7.1082624011	3.8634458596
H	-1.6324692962	-8.8427620177	3.5599343337
H	-1.0193062692	-8.6303831537	0.5051251620
H	-0.0709801374	-9.5676615183	1.6854850250
H	0.7311603676	-8.3441539418	0.6596008858
H	0.5869715369	-6.8074364061	4.1143582908
H	1.6862579514	-7.3003469828	2.7939106476
H	0.8159919671	-8.5302255080	3.7400433143
H	-4.5967445288	-6.6258202909	-3.6955120393
H	-4.5044704214	-5.4227557772	-5.0039446224
H	-6.0048665178	-6.3622738194	-4.7586549904
H	-6.1455468829	-6.2014104413	-1.6735193369
H	-7.5121302500	-5.8678068064	-2.7586144684
H	-7.0193019764	-4.6553951263	-1.5427041124
H	-5.9328077204	-3.3198138164	-4.9308348312
H	-6.9189547599	-2.9519464749	-3.4863455652
H	-7.3810066558	-4.2832051615	-4.5743310754

13(c)

112

-2.0472340989262627e+03 frame 1 xyz file generated by TeraChem

C	-0.7813833499	1.0248622203	-0.5514801971
C	-1.4117567682	-0.2163946121	-0.5418890666
C	-0.6582778769	-1.3994787539	-0.5649975153
C	0.7415897023	-1.3071248376	-0.5811778207
C	1.3721068835	-0.0663841541	-0.5335852329

C	0.6186410614	1.1170247923	-0.5265193099
C	4.4479064431	4.0972888150	-2.6067375000
C	4.0535406502	2.7844706613	-2.9420741748
C	3.0072716027	2.1180137481	-2.2997649458
C	2.3285714754	2.7821654010	-1.2766105629
C	2.6828725050	4.1098968977	-0.9315373069
C	3.7329743452	4.7488852768	-1.5928652438
N	1.2511166227	2.3752436368	-0.4856981631
C	0.9101704369	3.4317915804	0.3634233081
C	1.7736951923	4.5270251352	0.1140333729
C	-0.0613336079	3.5151435072	1.3624442009
C	-0.1769541563	4.7117213691	2.0726949834
C	0.6476544214	5.8303227013	1.8306428966
C	1.6345697100	5.7100410614	0.8437192250
C	-4.5109156716	-4.2962174092	-2.7076800466
C	-4.0901028041	-2.9831242857	-3.0271313625
C	-3.0385904205	-2.3422345384	-2.3776449100
C	-2.3748784316	-3.0358040657	-1.3591155511
C	-2.7516312116	-4.3599956402	-1.0339772807
C	-3.8163379311	-4.9762064403	-1.7046743522
N	-1.2936592427	-2.6573138632	-0.5609536548
C	-0.9729856511	-3.7311555881	0.2726705372
C	-1.8526079747	-4.8061748758	0.0061763980
C	-0.0025742084	-3.8445776674	1.2742308087
C	0.0867154276	-5.0500374240	1.9644220888
C	-0.7600098627	-6.1544362379	1.7062581731
C	-1.7383705243	-6.0066400448	0.7205608264
C	5.6028993514	4.8384555054	-3.3046647086
C	0.4999024754	7.1509282629	2.5998371223
C	-5.6893571134	-4.9192537097	-3.4678864374
C	-0.5774858375	-7.4486780538	2.5091152023
C	5.0342630541	6.0957760810	-3.9882721495
C	6.6380990432	5.2568940051	-2.2446771669
C	6.2814824560	3.9371690818	-4.3605433258
C	1.8191917163	7.4875799390	3.3406153564
C	0.2075225529	8.2851727818	1.6004441232
C	-0.6568871425	7.1108356405	3.6047747808
C	-1.6088771681	-8.5157199535	2.1254051399
C	0.8517621549	-8.0093021872	2.2917585707
C	-0.7513068060	-7.1467654365	4.0081578577
C	-5.9980580033	-6.3412269834	-2.9856077832
C	-6.9523053607	-4.0342934908	-3.3110043811
C	-5.3406195535	-4.9962093466	-4.9654068297
C	7.4599486578	4.5391277743	-5.1196270740
C	2.3004901973	6.4552591100	4.3512992537
C	-7.4514370787	-3.8257138222	-1.8871644709
C	1.2228702095	-8.3616261199	0.8570868595
H	-1.3807561215	1.9388806200	-0.5697317721

H	-2.5032800850	-0.2726236140	-0.5154703214
H	1.3411921697	-2.2202667091	-0.6232197513
H	2.4634991459	-0.0115215794	-0.5009105313
H	4.5746987491	2.2471271271	-3.7370046287
H	2.7329439108	1.1059714948	-2.6078447480
H	3.9903406551	5.7742655221	-1.3081334152
H	-0.7154958984	2.6725279656	1.5992414565
H	-0.9446824332	4.7657256956	2.8479756774
H	2.3090644891	6.5461399165	0.6317840691
H	-4.6082523033	-2.4358543402	-3.8209176865
H	-2.7433481264	-1.3314907900	-2.6707672688
H	-4.0923159989	-5.9966804982	-1.4284807187
H	0.6655780086	-3.0166847210	1.5239005473
H	0.8512953055	-5.1335326372	2.7432741186
H	-2.4298626683	-6.8209896402	0.4905947337
H	4.5223380239	6.7491817149	-3.2637365312
H	4.3013984885	5.8236500047	-4.7665618577
H	5.8328846948	6.6927160331	-4.4593175141
H	6.1818325566	5.8767737926	-1.4565624508
H	7.4509652643	5.8505456607	-2.6942338005
H	7.0847324612	4.3739541411	-1.7565718956
H	5.5172216850	3.6233390814	-5.0943412379
H	6.6233158676	3.0127889063	-3.8598559740
H	1.6820738614	8.4649392631	3.8406570679
H	2.6113430109	7.6526563619	2.5874981168
H	1.0186216944	8.4020144333	0.8633321460
H	0.0979400160	9.2478828320	2.1299165683
H	-0.7253176672	8.0912776855	1.0451413688
H	-0.5307580628	6.3279790103	4.3697553420
H	-1.6219345724	6.9369876413	3.1002654959
H	-0.7279008192	8.0785264702	4.1295625588
H	-1.5586635274	-8.7905364181	1.0598758207
H	-2.6370013332	-8.1783335868	2.3386960500
H	-1.4325002095	-9.4320431792	2.7135567578
H	0.9631731313	-8.9019239900	2.9359662367
H	1.5781148415	-7.2732369213	2.6812903307
H	-1.7565949576	-6.7442574803	4.2171283629
H	-0.0137149689	-6.4101918774	4.3660977055
H	-0.6182265122	-8.0661456408	4.6049315188
H	-6.2454243150	-6.3789931578	-1.9128529686
H	-5.1463030968	-7.0192683198	-3.1605482626
H	-6.8615469750	-6.7448780399	-3.5408528632
H	-7.7548634470	-4.4854537893	-3.9244866400
H	-6.7484983023	-3.0506455983	-3.7709619927
H	-4.4404146816	-5.6115642102	-5.1301724697
H	-5.1481951428	-3.9988143410	-5.3934632130
H	-6.1731068460	-5.4474738087	-5.5331622570
H	7.1769866379	5.4364791944	-5.6944758988

H	7.8588968936	3.8073973427	-5.8414813151
H	8.2909795082	4.8186104376	-4.4515539969
H	1.5906103993	6.3254673736	5.1853755113
H	3.2648449020	6.7594730580	4.7914022051
H	2.4439497659	5.4690059152	3.8787380047
H	-7.7757043965	-4.7691871586	-1.4171440051
H	-8.3176904632	-3.1432922901	-1.8734636096
H	-6.6683782575	-3.3860458749	-1.2465046068
H	0.5997836984	-9.1767994493	0.4526871289
H	2.2717951137	-8.6968921418	0.7972497416
H	1.1107993549	-7.4923794607	0.1870442256

13(d)
68
-1.7197437902653799e+03 frame 1 xyz file generated by TeraChem

C	-0.7172988343	1.1665795782	-0.3214701536
C	-1.3762695544	-0.0599218254	-0.2971732042
C	-0.6502230992	-1.2609899461	-0.3072865477
C	0.7521661524	-1.2022785077	-0.3122421740
C	1.4109836506	0.0243225417	-0.2883464878
C	0.6850092497	1.2252872007	-0.3077092424
C	4.3911744626	4.0701318655	-2.6880837222
C	3.9880598142	2.7492058898	-2.9701541435
C	2.9825549274	2.1258093007	-2.2266711525
C	2.3815860218	2.8355259451	-1.1864260609
C	2.7684766606	4.1728239733	-0.9095985719
C	3.7738592347	4.7862014263	-1.6571454385
N	1.3498002448	2.4663307930	-0.3180965898
C	1.0681365551	3.5559123304	0.5111683217
C	1.9285958446	4.6334765904	0.1748519583
C	0.1568755163	3.6794373763	1.5602209482
C	0.0872993975	4.8934250914	2.2492580274
C	0.9218901961	5.9761039183	1.9048105559
C	1.8530521212	5.8405886007	0.8696305784
C	-4.3453975338	-4.1130590776	-2.6963399073
C	-3.9400163000	-2.7934783315	-2.9814382794
C	-2.9378442700	-2.1678358593	-2.2353130208
C	-2.3425206303	-2.8738735297	-1.1893333042
C	-2.7314242105	-4.2099752744	-0.9096645450
C	-3.7334623090	-4.8256158604	-1.6597963158
N	-1.3149612459	-2.5020059097	-0.3170901032
C	-1.0376868351	-3.5889326354	0.5171809337
C	-1.8970212059	-4.6672351497	0.1803773553
C	-0.1314134006	-3.7096015176	1.5708907782
C	-0.0653089130	-4.9215901419	2.2638217552
C	-0.8986474511	-6.0050473437	1.9188148854
C	-1.8252044630	-5.8722014111	0.8791930158
O	5.3679654846	4.7250000746	-3.3675798802

O	0.8914569982	7.1845720738	2.5233943913
O	-5.3192473644	-4.7699582167	-3.3780408072
O	-0.8713290790	-7.2118060464	2.5408307970
C	5.9856932953	4.0849697766	-4.4495510847
C	-0.0143670111	7.3825773377	3.5730338142
C	-5.9345705288	-4.1319923123	-4.4625997997
C	0.0331144817	-7.4089025101	3.5918307630
H	-1.2929067388	2.0960368888	-0.3498487293
H	-2.4691930258	-0.0930054096	-0.2755693574
H	1.3279028718	-2.1318418899	-0.3333337834
H	2.5037426022	0.0576097345	-0.2597993693
H	4.4592759056	2.1900260059	-3.7813674277
H	2.6775076487	1.1059570093	-2.4744693270
H	4.0916387295	5.8150610744	-1.4665440342
H	-0.4925204938	2.8508449448	1.8543807053
H	-0.6312584862	4.9869535028	3.0664109249
H	2.5021982415	6.6873864168	0.6300527964
H	-4.4066961534	-2.2371706363	-3.7972385641
H	-2.6309052003	-1.1491758855	-2.4856299141
H	-4.0527388033	-5.8535641776	-1.4668259104
H	0.5168909195	-2.8803773863	1.8656254418
H	0.6494718234	-5.0129126455	3.0845366071
H	-2.4735992203	-6.7194518999	0.6391927951
H	6.7144340221	4.7975130168	-4.8636066764
H	5.2611219623	3.8168327116	-5.2430980059
H	6.5242230850	3.1677605088	-4.1405101515
H	0.1281734321	8.4160278388	3.9221727790
H	0.1708535940	6.6933336369	4.4198117358
H	-1.0649108517	7.2615181564	3.2437833789
H	-6.6632044589	-4.8449159544	-4.8762035119
H	-5.2084253212	-3.8664387832	-5.2555860367
H	-6.4727881076	-3.2135772008	-4.1566336130
H	-0.1107173922	-8.4416860229	3.9424350727
H	-0.1523862335	-6.7182339483	4.4373824110
H	1.0840908781	-7.2892348716	3.2634355559

13(e)
76
-1.7745339036889154e+03 frame 1   xyz file generated by TeraChem

C	-0.1802167192	0.9202121632	-0.2025727500
C	-0.8530885685	-0.2829078442	-0.4055840422
C	-0.1382529387	-1.4755836315	-0.5906330086
C	1.2634230764	-1.4431619084	-0.5478190463
C	1.9344938199	-0.2467365470	-0.3073591334
C	1.2205739991	0.9491240300	-0.1420928465
C	4.9171934103	4.1974615037	-2.0153274013
C	4.4185687183	2.9933671729	-2.5590982980
C	3.4222278813	2.2374604443	-1.9408456854

C	2.9041754774	2.6988096283	-0.7293215305
C	3.3683246620	3.9120974169	-0.1626720627
C	4.3690760061	4.6454723552	-0.8071005002
N	1.9056955522	2.1638578946	0.0824049026
C	1.7200519272	3.0196080268	1.1692452880
C	2.6123895517	4.1169139494	1.0525493967
C	0.8604177880	2.8966967206	2.2657304498
C	0.8874513030	3.8948342986	3.2382440766
C	1.7616994447	4.9820913977	3.1090717266
C	2.6309534289	5.1139425359	2.0359861708
C	-3.6105182738	-3.8855405527	-3.6300516026
C	-3.1659476546	-2.5604945253	-3.7280429166
C	-2.2301922247	-2.0652246762	-2.8218594046
C	-1.7612944955	-2.9232281238	-1.8217151432
C	-2.2075791512	-4.2677508475	-1.7368638790
C	-3.1485620168	-4.7549665412	-2.6529075567
N	-0.8184951277	-2.6912305267	-0.8187052563
C	-0.6555770864	-3.8691439377	-0.0911020781
C	-1.5033653679	-4.8705557845	-0.6271877487
C	0.1400527002	-4.1373056525	1.0244693661
C	0.0827595003	-5.4154700578	1.5813415290
C	-0.7438220412	-6.4391014516	1.0687609567
C	-1.5368808239	-6.1415818683	-0.0464298789
C	6.0272040609	5.0135802760	-2.6957367670
F	1.7558975809	5.9241595391	4.0632200034
F	-4.5124575325	-4.3227639910	-4.5210620313
C	-0.8098624652	-7.8401831618	1.6952662243
C	5.5145027112	6.4324953402	-2.9994910339
C	-2.2533217425	-8.1315513589	2.1432336889
C	7.2418877935	5.1039002106	-1.7548499459
C	6.4884300047	4.3807299988	-4.0134413045
C	-0.3764562716	-8.8902092964	0.6569931590
C	0.1037448390	-7.9748816430	2.9187199180
H	-0.7427511935	1.8511585932	-0.0865150434
H	-1.9464462102	-0.3028564594	-0.4243480824
H	1.8279617487	-2.3669064132	-0.7032865032
H	3.0267406248	-0.2321846982	-0.2502947208
H	4.8199432553	2.6253110402	-3.5059838898
H	3.0596088461	1.3147243308	-2.4016046045
H	4.7195175530	5.5786301300	-0.3542086504
H	0.1863864844	2.0425241579	2.3689163287
H	0.2343768741	3.8470880142	4.1139037264
H	3.3033212200	5.9741683645	1.9810916514
H	-3.5665562290	-1.9308490306	-4.5272121188
H	-1.8750432586	-1.0348854980	-2.9034116081
H	-3.5168267803	-5.7838375650	-2.6204449764
H	0.7853896567	-3.3715876464	1.4629752564
H	0.7069957159	-5.6146830001	2.4555650858

H	-2.1984921798	-6.9037381241	-0.4709269509
H	5.2006283953	6.9606374847	-2.0845320839
H	4.6502971591	6.4037687014	-3.6838606640
H	6.3078043539	7.0354880828	-3.4747468645
H	-2.9611524088	-8.0967427868	1.2991627199
H	-2.5901291147	-7.3963940277	2.8931302494
H	-2.3214701564	-9.1369227722	2.5940495887
H	6.9857745564	5.5951366349	-0.8019179852
H	8.0515021180	5.6886132954	-2.2252022132
H	7.6358879422	4.1011793137	-1.5190569994
H	5.6663947089	4.3094371062	-4.7450419418
H	6.9065055465	3.3716183967	-3.8619756676
H	7.2803880627	5.0004641537	-4.4660853399
H	-1.0137768257	-8.8683710643	-0.2421400157
H	-0.4396677308	-9.9058579861	1.0852090266
H	0.6638371600	-8.7201033821	0.3328896693
H	-0.1657639796	-7.2617757898	3.7155733806
H	1.1647665034	-7.8196005985	2.6609337125
H	0.0116417582	-8.9899901736	3.3395304348

13(f) ω=0.0536
58
E = -1935.4609866205183

C	-0.6237653049	1.2091647723	-0.3537734707
C	-1.3961948507	0.0474167226	-0.3541910004
C	-0.7594753583	-1.2054409662	-0.3327886564
C	0.6370705374	-1.2617311174	-0.3121271377
C	1.4094310564	-0.1000928033	-0.3090476322
C	0.7727648636	1.1530346390	-0.3298086496
C	2.8378820810	4.8882104642	-3.3269187810
C	2.2434336048	3.6785246528	-3.7268315529
C	1.7616021204	2.7625004272	-2.7930777421
C	1.8889180033	3.0923601428	-1.4416813236
C	2.4767030893	4.3102149782	-1.0173592932
C	2.9577856952	5.2098670386	-1.9769265224
N	1.5085397289	2.3612716950	-0.3173517839
C	1.8071846875	3.1049534006	0.8233763508
C	2.4233334933	4.3188394856	0.4302905741
C	1.5821395211	2.7873433947	2.1648036221
C	1.9906540181	3.7145509914	3.1220742779
C	2.6093729154	4.9222824545	2.7541505365
C	2.8291717456	5.2301002378	1.4134481211
C	-2.6907043894	-5.0068355159	-3.3315859548
C	-2.0951092052	-3.7977308938	-3.7316271415
C	-1.6578963060	-2.8596648686	-2.7977980053
C	-1.8307022776	-3.1672650969	-1.4462622630
C	-2.4213427084	-4.3834322787	-1.0213799007
C	-2.8573478158	-5.3054051900	-1.9812374064

N	-1.4951304001	-2.4137534475	-0.3223016953
C	-1.8244047832	-3.1415086864	0.8202012836
C	-2.4178072266	-4.3667952187	0.4271904746
C	-1.6460680330	-2.8008341138	2.1630522719
C	-2.0801768157	-3.7152719391	3.1213978569
C	-2.6784133387	-4.9331657148	2.7531627601
C	-2.8505334439	-5.2646614428	1.4111682960
C	2.9216794353	-0.2100108802	-0.2767365059
F	3.4793599285	0.3740107798	-1.3432219586
F	3.4329639281	0.3690414680	0.8151143009
F	3.3184231546	-1.4893021496	-0.2713061054
C	-2.9087308174	0.1568658070	-0.3733295416
F	-3.4300882848	-0.4221235100	-1.4606474703
F	-3.4566265161	-0.4278111371	0.6977877228
F	-3.3057942209	1.4360668896	-0.3751288361
H	-1.1081880708	2.1885330412	-0.3676173808
H	1.1212043934	-2.2412288977	-0.2953571718
H	3.2098361098	5.5826471125	-4.0865164297
H	2.1593937187	3.4473149084	-4.7936207535
H	1.3093906272	1.8181101738	-3.1090956270
H	3.4204602988	6.1530299812	-1.6690740212
H	1.1090760544	1.8449092691	2.4551225192
H	1.8279667621	3.4943577914	4.1820629471
H	2.9214269292	5.6264333385	3.5315546172
H	3.3108931961	6.1719351378	1.1317886561
H	-3.0259950948	-5.7194140321	-4.0914405484
H	-1.9738800827	-3.5849846369	-4.7986738571
H	-1.2041619426	-1.9160488014	-3.1140279678
H	-3.3204007902	-6.2483454723	-1.6732698579
H	-1.1893109071	-1.8505239901	2.4539006781
H	-1.9545369560	-3.4766894259	4.1824549680
H	-3.0120136158	-5.6265674219	3.5312827503
H	-3.3159505013	-6.2145328332	1.1291009854

13(g) ω=0.0261
106
E = -2563.2494533811941

C	0.6302271378	0.0481632438	-1.2590490131
C	-0.7609237372	0.0715431595	-1.1558551564
C	-1.4084755657	0.0868082595	0.0843211817
C	-0.6301792063	0.0602242813	1.2573547856
C	0.7610903344	0.0831195971	1.1538311739
C	1.4085708847	0.0863085753	-0.0862855170
C	5.7414686334	2.9986224076	-0.8259586413
C	4.3808566226	3.3651711534	-0.9659621949
C	3.3303022108	2.4764057813	-0.7577352856
C	3.6507266151	1.1648685602	-0.3996997429
C	4.9964958119	0.7637582613	-0.2346220045

C	6.0304446686	1.6835757992	-0.4541526113
N	2.8191591638	0.0809887101	-0.1140345655
C	3.6150665292	-1.0032439388	0.2520668310
C	4.9741100567	-0.6226787828	0.1792455120
C	3.2471508018	-2.2978287113	0.6260397070
C	4.2655792058	-3.1957960444	0.9324698659
C	5.6382074009	-2.8539011316	0.8738289945
C	5.9749037572	-1.5532456291	0.4892672401
C	-5.7418631320	2.9985173337	0.8256154523
C	-4.3812499946	3.3656744347	0.9641250196
C	-3.3305226875	2.4772528553	0.7553216817
C	-3.6506444895	1.1652901190	0.3985440118
C	-4.9964266287	0.7636489103	0.2348327801
C	-6.0305845168	1.6831742432	0.4547409694
N	-2.8189776904	0.0818377323	0.1114532947
C	-3.6148698531	-1.0028878256	-0.2532756211
C	-4.9739698475	-0.6227642909	-0.1790278961
C	-3.2470985779	-2.2975291022	-0.6272436477
C	-4.2656065575	-3.1957429820	-0.9327728185
C	-5.6382596175	-2.8540580504	-0.8732651864
C	-5.9748390379	-1.5535206690	-0.4882700630
C	6.8353245630	4.0463827298	-1.0748681933
C	6.6924152257	-3.9100463686	1.2335544327
C	-6.6925081350	-3.9102057446	-1.2327216200
C	-6.8358502603	4.0459907964	1.0749971929
C	6.6507449634	5.2182836540	-0.0940854196
C	8.2405820085	3.4681353617	-0.8752189104
C	6.7294862484	4.5650594265	-2.5203102694
C	6.5368003713	-5.1273985355	0.3045350118
C	8.1197327648	-3.3719217494	1.0844129007
C	6.4945477531	-4.3503395576	2.6954999090
C	-6.6520701917	5.2179087444	0.0940910846
C	-8.2410441630	3.4673815694	0.8760671354
C	-6.7293616206	4.5647281194	2.5203662593
C	-6.5366056400	-5.1276199580	-0.3038522561
C	-8.1197836680	-3.3721053419	-1.0832030030
C	-6.4950115054	-4.3503717489	-2.6947553279
C	-1.2533055257	-0.0238421668	2.6395441122
F	-1.7379927900	1.1579070750	3.0423476123
F	-2.2460586918	-0.9145427902	2.6793150717
F	-0.3414532093	-0.4027538208	3.5481606388
C	1.2547096766	-0.0476505574	-2.6398912050
F	1.7500148731	1.1280058510	-3.0469095510
F	2.2405665280	-0.9466297358	-2.6733322390
F	0.3418452393	-0.4236337290	-3.5484752853
H	-1.3742144433	0.0522632771	-2.0601613293
H	1.3745835134	0.0721658966	2.0581672984
H	4.1312779106	4.3925320893	-1.2496525528

H	2.2929068343	2.7984485906	-0.8822064681
H	7.0639075431	1.3543018370	-0.3227356750
H	2.1977911513	-2.6033670371	0.6761328164
H	3.9812420625	-4.2102064468	1.2295983621
H	7.0205803622	-1.2430085829	0.4250004713
H	-4.1318069955	4.3933029260	1.2469530449
H	-2.2932466633	2.8000450206	0.8784736872
H	-7.0640283151	1.3534608480	0.3241818440
H	-2.1977856110	-2.6030518596	-0.6783061408
H	-3.9812934644	-4.2101192582	-1.2300212988
H	-7.0205290867	-1.2434591350	-0.4231800427
H	6.7254157816	4.8737591392	0.9511204389
H	5.6686484242	5.7030317973	-0.2174428239
H	7.4262484305	5.9871553240	-0.2569792063
H	8.3920358728	3.0996458511	0.1531584189
H	8.9957102315	4.2503667842	-1.0600576781
H	8.4465605127	2.6388421992	-1.5725136022
H	5.7537238160	5.0387055128	-2.7167571923
H	6.8538865514	3.7429763991	-3.2449859582
H	7.5113503554	5.3188498407	-2.7194762405
H	6.6720221600	-4.8380639524	-0.7511773227
H	5.5418921946	-5.5917991001	0.4000394345
H	7.2886546614	-5.8986652044	0.5471051975
H	8.3353191321	-3.0582704955	0.0492178040
H	8.8449756097	-4.1594563217	1.3493762158
H	8.3072446713	-2.5137381854	1.7511591376
H	5.4960963685	-4.7871587936	2.8613183374
H	6.6039126429	-3.4947032531	3.3829072020
H	7.2435120067	-5.1120783198	2.9742174375
H	-6.7271810191	4.8733468740	-0.9510762346
H	-5.6700516539	5.7029417441	0.2169338925
H	-7.4276961748	5.9865895217	0.2573502662
H	-8.3928816814	3.0987509246	-0.1522154286
H	-8.9962966066	4.2494373343	1.0611819980
H	-8.4465256274	2.6380988186	1.5735354439
H	-5.7535618758	5.0385224225	2.7162871538
H	-6.8532539077	3.7426488593	3.2451398627
H	-7.5112510329	5.3184025857	2.7199129809
H	-6.6716173327	-4.8384031444	0.7519233643
H	-5.5416882090	-5.5919559739	-0.3996261615
H	-7.2884673926	-5.8989179280	-0.5463290834
H	-8.3351733741	-3.0585602706	-0.0479265199
H	-8.8450945765	-4.1596057475	-1.3481016143
H	-8.3074283213	-2.5138425194	-1.7498261080
H	-5.4966220242	-4.7872190409	-2.8608831303
H	-6.6045256118	-3.4946740889	-3.3820709670
H	-7.2440737887	-5.1120658300	-2.9733532505

13(h)
118
-2.7201902856784181e+03 frame 1 xyz file generated by TeraChem

C	-1.4850197759	0.1674391288	-0.2103298036
C	-0.7116290746	1.3270584186	-0.2746097823
C	0.6874232019	1.2978619172	-0.3088017057
C	1.3283931712	0.0434365760	-0.2394491392
C	0.5543488759	-1.1168299749	-0.2662393738
C	-0.8456823606	-1.0883679530	-0.2705249729
C	-4.1287723938	-4.1516167073	-3.0397124244
C	-3.6557061834	-2.8546295574	-3.3339461205
C	-2.7918608345	-2.1528441250	-2.4905859464
C	-2.3921072145	-2.7654100396	-1.3049841883
C	-2.8193473525	-4.0775654344	-0.9894229349
C	-3.6858205659	-4.7511400776	-1.8529290181
N	-1.5348640188	-2.3138436805	-0.2969226979
C	-1.3890736541	-3.3344316421	0.6460761683
C	-2.1743846536	-4.4416806283	0.2549623416
C	-0.6427285157	-3.3676882434	1.8271817271
C	-0.6809620914	-4.5343526103	2.5865710725
C	-1.4399919185	-5.6704175246	2.2181956130
C	-2.1930833994	-5.5998633507	1.0427039369
C	3.8634349061	4.3329368003	-3.2247915369
C	3.3832944938	3.0273525553	-3.4854898022
C	2.5539291641	2.3335930327	-2.6089134156
C	2.1951164765	2.9647118318	-1.4159040663
C	2.6293892138	4.2796547385	-1.1334132209
C	3.4644420905	4.9503869102	-2.0370096695
N	1.3771616066	2.5226971206	-0.3723953925
C	1.2675085344	3.5511434351	0.5655842531
C	2.0346508980	4.6554930706	0.1312183984
C	0.5705597173	3.5934545285	1.7763526433
C	0.6409840892	4.7653974540	2.5244875715
C	1.3845029686	5.8983171232	2.1154056140
C	2.0857496950	5.8198590225	0.9090600642
C	-5.1027972633	-4.9189505145	-3.9529366547
C	-1.4044214946	-6.9203876390	3.1078567814
C	4.7885776972	5.0116803878	-4.2434161106
C	1.3870234458	7.1543264333	2.9970244208
C	-4.4662482923	-6.2652037816	-4.3446298170
C	-2.2971538407	-8.0411910070	2.5623170740
C	2.2636616178	8.2672036231	2.4108225150
C	5.1844482403	6.4272684704	-3.8077849588
C	0.0521881058	-7.4351546504	3.2372963490
C	-1.9155131951	-6.5548192202	4.5129816643
C	-6.4030960939	-5.1745586473	-3.1685521602
C	-5.4179968731	-4.1069097704	-5.2295327874
C	6.0648475418	4.1557430543	-4.4488782746

C	4.0585772184	5.1269456584	-5.5940789703
C	-0.0623810351	7.6756953472	3.1751311450
C	1.9475159826	6.7967304242	4.3852729879
C	-6.3598688359	-4.7564983578	-6.2390620024
C	0.7384602096	-7.8337645837	1.9363548646
C	6.9190869499	3.9235218003	-3.2092861799
C	-0.7944112805	8.0636240737	1.8960716681
C	2.8294236173	-0.1377545096	-0.0990535107
F	3.4410133469	-0.2776588056	-1.2841892492
F	3.4108178158	0.8751505026	0.5436242615
F	3.0893746329	-1.2555867498	0.6002166169
C	-2.9815679633	0.3570012083	-0.0372133920
F	-3.6167593218	0.5167324114	-1.2080759192
F	-3.5593132253	-0.6569778072	0.6060787781
F	-3.2182711935	1.4701122624	0.6783865092
H	-1.2045700198	2.3025742607	-0.2660631258
H	1.0475629768	-2.0919681821	-0.2521524855
H	-3.9653703706	-2.3573207847	-4.2553096149
H	-2.4559941224	-1.1487627945	-2.7610819158
H	-4.0138857531	-5.7617242474	-1.5889206082
H	-0.0431622772	-2.5126770713	2.1526333017
H	-0.0899093764	-4.5634028222	3.5075087263
H	-2.8020739547	-6.4468609041	0.7178825781
H	3.6696987312	2.5278524840	-4.4164747272
H	2.2110841929	1.3249015230	-2.8522375116
H	3.7939698817	5.9637761901	-1.7951253700
H	-0.0140160498	2.7402836106	2.1327474195
H	0.0902706783	4.8016580697	3.4697209086
H	2.6796167641	6.6648659877	0.5526465795
H	-4.2118352919	-6.8659217461	-3.4570718265
H	-3.5383713182	-6.1101221005	-4.9207899567
H	-5.1555458384	-6.8689459757	-4.9574806703
H	-2.0107117128	-8.3561009337	1.5462924212
H	-3.3564471493	-7.7358915385	2.5346386190
H	-2.2256235399	-8.9264147794	3.2165676135
H	1.9362876319	8.5808875492	1.4069270676
H	3.3186729264	7.9542586773	2.3396276321
H	2.2265430711	9.1551125571	3.0644376135
H	5.6995440679	6.4448589479	-2.8341589704
H	4.3039702668	7.0873622398	-3.7345827054
H	5.8674489951	6.8695333912	-4.5526490117
H	0.0439038760	-8.2953645504	3.9330797930
H	0.6551164479	-6.6572682161	3.7394415133
H	-2.9525422880	-6.1817382685	4.4713661316
H	-1.2975851664	-5.7734219853	4.9849425355
H	-1.8966704642	-7.4384379706	5.1747279282
H	-6.2074300477	-5.7344317564	-2.2398297135
H	-7.1202743049	-5.7651744319	-3.7624606029

H	-6.8876504296	-4.2239139571	-2.8884944025
H	-4.4645768475	-3.8754120431	-5.7382260067
H	-5.8472216377	-3.1342108307	-4.9281113372
H	6.6735368827	4.6423416657	-5.2345684157
H	5.7672635121	3.1788071815	-4.8708896010
H	3.1393646672	5.7280649348	-5.4944768477
H	3.7723654042	4.1403849729	-5.9939043399
H	4.7073754165	5.6141952547	-6.3428089708
H	-0.0261256448	8.5415922173	3.8627868460
H	-0.6489612162	6.9038979975	3.7052635314
H	2.9803699421	6.4176545732	4.3087484015
H	1.3426466400	6.0229796727	4.8856093681
H	1.9572424188	7.6857042019	5.0399590342
H	-5.9564652212	-5.7011278659	-6.6387884464
H	-6.5192547691	-4.0831415815	-7.0975651474
H	-7.3516862021	-4.9717346198	-5.8084387115
H	0.2350442093	-8.6825490598	1.4445940660
H	1.7817154148	-8.1384165918	2.1226595896
H	0.7574404212	-6.9955272369	1.2197751826
H	7.3214916063	4.8649438522	-2.7997660089
H	7.7813999668	3.2774831047	-3.4442790228
H	6.3425322587	3.4304555312	-2.4083857954
H	-0.3112133742	8.9099341194	1.3802442945
H	-1.8315141534	8.3667726527	2.1161380885
H	-0.8354917870	7.2196161181	1.1872846107

13(i) ω=0.0261

741

E = -2392.7011133429351

C	-0.6748713355	-0.0377215924	-1.2353781887
C	0.7183418381	-0.0695687414	-1.1787841635
C	1.4140656108	-0.0762260969	0.0361589400
C	0.6748379140	-0.0377384675	1.2353457993
C	-0.7184105934	-0.0695495276	1.1786657710
C	-1.4140813142	-0.0762058807	-0.0362555694
C	-5.8269979518	-2.8283354494	-0.8056387217
C	-4.5003692065	-3.2283201806	-1.0660454830
C	-3.4267908590	-2.3666230500	-0.8279599502
C	-3.6945521388	-1.0914663680	-0.3361302949
C	-5.0232433028	-0.6865834072	-0.0450942938
C	-6.0877885103	-1.5559058432	-0.2856583405
N	-2.8209974250	-0.0510265611	-0.0035066006
C	-3.5717997320	1.0051250095	0.5171072847
C	-4.9451994991	0.6535323848	0.5005994187
C	-3.1527760930	2.2496675579	0.9817484968
C	-4.1184085834	3.1453721947	1.4474299664
C	-5.4867879543	2.8057007925	1.4441364436
C	-5.9016965591	1.5576261140	0.9639968845

C	5.8269869910	-2.8284556515	0.8056485749
C	4.5004063003	-3.2283257948	1.0659595340
C	3.4267533444	-2.3667171326	0.8279107831
C	3.6946116026	-1.0914520992	0.3360505096
C	5.0232669966	-0.6866528979	0.0450668406
C	6.0878087381	-1.5559010143	0.2856431192
N	2.8209241930	-0.0510639757	0.0035325158
C	3.5718467532	1.0051291952	-0.5171230928
C	4.9452727063	0.6535429421	-0.5006162522
C	3.1528378532	2.2496261233	-0.9817257328
C	4.1184477355	3.1453801627	-1.4473697152
C	5.4868873750	2.8057243558	-1.4440861663
C	5.9017069404	1.5576375598	-0.9639333124
O	-6.9117682048	-3.6154288804	-1.0241296960
O	-6.4689513221	3.6319982045	1.8865789016
O	6.4690760687	3.6320473333	-1.8865051985
O	6.9118379256	-3.6155012140	1.0241170479
C	-6.7266629388	-4.8727883004	-1.6138260065
C	-6.1198075436	4.9064342279	2.3533351997
C	6.7266232018	-4.8728593117	1.6138067433
C	6.1198528050	4.9064647633	-2.3532229645
C	-1.3349506005	0.0927400113	-2.5963955713
F	-1.8295366878	-1.0727812927	-3.0335245564
F	-2.3270184903	0.9848788677	-2.5805202863
F	-0.4455037024	0.5037794469	-3.5143139625
C	1.3349217794	0.0927641391	2.5963232190
F	1.8295317586	-1.0727355052	3.0335170367
F	2.3269943682	0.9848961275	2.5805120174
F	0.4455093802	0.5038027865	3.5143182570
H	1.2985246692	-0.0510811777	-2.1041832070
H	-1.2985666379	-0.0510164039	2.1042172525
H	-4.2907288780	-4.2247211630	-1.4607985710
H	-2.4067125910	-2.6957333083	-1.0407623992
H	-7.1237241168	-1.2737452316	-0.0787619582
H	-2.0963515562	2.5324632658	0.9867907638
H	-3.7910637535	4.1204032546	1.8141185088
H	-6.9694059586	1.3217431049	0.9646654996
H	4.2907051100	-4.2247842938	1.4607474294
H	2.4066943719	-2.6958132636	1.0407104157
H	7.1236693018	-1.2737692257	0.0788094609
H	2.0963780170	2.5325361648	-0.9868138221
H	3.7911362435	4.1204340851	-1.8140521727
H	6.9693584114	1.3217299381	-0.9645804694
H	-7.7259263645	-5.3186388904	-1.7280162178
H	-6.1107336598	-5.5434709825	-0.9833408088
H	-6.2549727723	-4.7968178699	-2.6127831529
H	-7.0561535523	5.4010662267	2.6515173272
H	-5.6298758383	5.5162475960	1.5696970159

H	-5.4494821639	4.8581007004	3.2334832504
H	7.7258415368	-5.3187233990	1.7280156380
H	6.1107039120	-5.5435471745	0.9833264520
H	6.2549277058	-4.7969269157	2.6127579359
H	7.0561492594	5.4011329391	-2.6513797046
H	5.6299003237	5.5162875439	-1.5696103034
H	5.4495361718	4.8581876113	-3.2333754758

13(j)

82

-2.4474892241882526e+03 frame 1 xyz file generated by TeraChem

C	-1.9014261616	0.2833945736	-0.0084294313
C	-1.1881604219	1.4449702620	-0.3028485723
C	0.1951706681	1.4412737738	-0.5202987190
C	0.8872670123	0.2194676011	-0.3931689569
C	0.1637842550	-0.9516919900	-0.1589365544
C	-1.2245724893	-0.9521387125	0.0141394399
C	-4.5401951883	-4.4442588684	-2.0995220276
C	-4.0939544495	-3.2010947917	-2.6095154654
C	-3.2158391856	-2.3691151441	-1.9231713234
C	-2.7671652642	-2.7930657225	-0.6697992152
C	-3.1841585309	-4.0299132307	-0.1285628728
C	-4.0696612389	-4.8454743977	-0.8470021096
N	-1.8772537061	-2.1840263831	0.2139174487
C	-1.7124680302	-3.0178185557	1.3207331986
C	-2.5161001442	-4.1735996863	1.1458231487
C	-0.9340401527	-2.8317949013	2.4662405495
C	-0.9576597151	-3.8270522343	3.4421805876
C	-1.7498730378	-4.9688335517	3.2632627081
C	-2.5347733376	-5.1645790058	2.1355991544
C	2.6797736018	4.1661712623	-4.2027223935
C	2.2849257601	2.8239132236	-4.2658407514
C	1.6525290961	2.2275948611	-3.1756883364
C	1.4383241915	3.0038310073	-2.0343768914
C	1.8168123423	4.3692303175	-1.9862858474
C	2.4538508449	4.9570832542	-3.0853099051
N	0.8187602116	2.6651283133	-0.8265548255
C	0.7697591542	3.8044079006	-0.0218873986
C	1.3877844540	4.8805716859	-0.7027207612
C	0.2483063401	3.9763624762	1.2601056893
C	0.3326336151	5.2448757940	1.8367212516
C	0.9275953597	6.3449393630	1.1825827291
C	1.4633605995	6.1362598589	-0.0947995930
C	-5.5023698573	-5.2982198567	-2.9360437067
F	-1.7469862021	-5.9039589653	4.2230081785
F	3.2900331087	4.7075093993	-5.2654471987
C	1.0008475178	7.7435240711	1.8138187884
C	-4.8393613098	-5.6386577913	-4.2832671605
C	2.4741883693	8.1653550484	1.9553855869

C	-5.8629823284	-6.6111912647	-2.2330356736
C	-6.8011128144	-4.5114489433	-3.1865847753
C	0.2663945035	8.7463933669	0.9057099507
C	0.3517374293	7.7891983265	3.2014034844
C	2.3942696040	0.0717730699	-0.4934178076
F	2.7690446366	-0.3232295075	-1.7223460669
F	3.0548794505	1.1920797655	-0.2081300110
F	2.8196883250	-0.8697361325	0.3627248360
C	-3.3855288245	0.4543472995	0.2618671141
F	-4.0798811546	0.5726921630	-0.8809473437
F	-3.9168458623	-0.5433536558	0.9636968913
F	-3.5935111676	1.5855650382	0.9562347292
H	-1.7135959887	2.4022183314	-0.3539036666
H	0.6898495720	-1.9077088098	-0.0929796220
H	-4.4491521934	-2.8703967775	-3.5904739508
H	-2.8991822605	-1.4168093993	-2.3566103478
H	-4.3798585405	-5.7981391592	-0.4115168211
H	-0.3228634375	-1.9356907941	2.6051510749
H	-0.3680007372	-3.7348961417	4.3582598909
H	-3.1418976736	-6.0692817654	2.0454803954
H	2.4809983248	2.2601344841	-5.1818183425
H	1.3471998642	1.1797884181	-3.2242652868
H	2.7662988663	6.0048478115	-3.0889896672
H	-0.2185138850	3.1516616155	1.8061913663
H	-0.0862504753	5.3736710169	2.8372622303
H	1.9434906523	6.9592103591	-0.6339129137
H	-3.9006906072	-6.1967733452	-4.1302342713
H	-4.6007155131	-4.7325349442	-4.8634528649
H	-5.5111478862	-6.2611957816	-4.8992964211
H	2.9877778473	8.1921565942	0.9807451349
H	3.0256095278	7.4662419097	2.6059264538
H	2.5455163828	9.1737401780	2.3984790328
H	-4.9730810625	-7.2355907194	-2.0491097825
H	-6.5526739387	-7.1956975126	-2.8641913359
H	-6.3666703463	-6.4346525823	-1.2681185657
H	-6.6118258486	-3.5670432574	-3.7222548221
H	-7.3018850562	-4.2637357936	-2.2358184144
H	-7.5020911580	-5.1066573981	-3.7973197246
H	0.7119021682	8.7895515001	-0.1013742392
H	0.3108586121	9.7627905983	1.3337393665
H	-0.7950871630	8.4705597422	0.7898661930
H	0.8502770746	7.1103564224	3.9131757844
H	-0.7181231457	7.5244724741	3.1643624235
H	0.4254808974	8.8094545124	3.6133367436

14(a)
54
-1.4126856798621395e+03 frame 18 xyz file generated by TeraChem

C	-0.7036637479	1.2416729748	-0.3065155948
C	-1.3281388467	-0.0039050206	-0.2802848153
C	-0.5613271016	-1.1753719357	-0.2818566802
C	0.8360107607	-1.0868798716	-0.2853225873
C	1.4601455400	0.1589060767	-0.2622532611
C	0.6935110487	1.3302891386	-0.2850287593
N	1.3275795495	2.6024857454	-0.2863635299
N	-1.1952659628	-2.4476530135	-0.2796759741
C	-1.1536973151	-3.2588186763	0.8647633655
C	-1.7777026013	-4.5223435944	0.8331437468
O	-2.4274492044	-4.9797047106	-0.2817580261
C	-2.4849748933	-4.1763309224	-1.3889822088
C	-1.8678963508	-2.9090479024	-1.4217975010
C	1.2707866010	3.4245406239	0.8496475690
C	1.8956909207	4.6875445942	0.8145173612
O	2.5618265498	5.1333565627	-0.2953628139
C	2.6328375444	4.3200940519	-1.3944983937
C	2.0156804423	3.0527731771	-1.4236898134
C	3.3289153294	4.8031163587	-2.4956077190
C	3.4233125156	4.0396987647	-3.6643200074
C	2.8044417477	2.7937253397	-3.7149515056
C	2.1039782063	2.3071874338	-2.6055655747
C	0.6342524472	3.0406858817	2.0363619976
C	0.5987063706	3.8940066398	3.1447927217
C	1.2093171804	5.1438045035	3.0897486285
C	1.8638198757	5.5336641702	1.9161734643
C	-0.5332359153	-2.8635651558	2.0562454034
C	-0.5124713648	-3.7063366657	3.1730823306
C	-1.1222871687	-4.9566893358	3.1217583115
C	-1.7608401460	-5.3578241083	1.9432231416
C	-3.1666088435	-4.6696468828	-2.4945631651
C	-3.2458750465	-3.9170843762	-3.6714113088
C	-2.6265625912	-2.6714694105	-3.7255036448
C	-1.9408163934	-2.1744711925	-2.6115777671
H	-1.2996524026	2.1589794385	-0.3254506647
H	-2.4198802263	-0.0754407389	-0.2763994297
H	1.4320117656	-2.0043560817	-0.2875286722
H	2.5517394485	0.2305399801	-0.2442385693
H	3.7899655421	5.7920483608	-2.4146044597
H	3.9746342203	4.4266407669	-4.5262546814
H	2.8567454445	2.1819867726	-4.6210384954
H	1.6169463111	1.3306295810	-2.6580798027
H	0.1578070612	2.0591310149	2.0925093231
H	0.0859194148	3.5642161910	4.0537781430
H	1.1854643051	5.8178952766	3.9509560296
H	2.3645434714	6.5027223791	1.8303458625
H	-0.0576209018	-1.8814430949	2.1094095763
H	-0.0118212123	-3.3678970501	4.0856456137

H	-1.1099581791	-5.6225688834	3.9895693256
H	-2.2601374332	-6.3278252241	1.8597648234
H	-3.6284627225	-5.6579475101	-2.4104893181
H	-3.7858150993	-4.3121346066	-4.5368663332
H	-2.6669875887	-2.0682097141	-4.6378579557
H	-1.4532503424	-1.1983260570	-2.6667177727

14(b)
54
-2.0583666976911632e+03 frame 42 xyz file generated by TeraChem

C	-0.7039052040	1.2373041340	-0.2956108689
C	-1.3281111042	-0.0098395176	-0.2689716968
C	-0.5602668472	-1.1797480527	-0.2697105254
C	0.8365263989	-1.0916077929	-0.2760564634
C	1.4605538152	0.1557746141	-0.2589444439
C	0.6928222951	1.3255955521	-0.2791594940
N	1.3317405142	2.6052400111	-0.2849473160
N	-1.1987697602	-2.4596127764	-0.2653824081
C	-1.2627297197	-3.1539286205	0.9586916554
C	-1.8446672856	-4.4345524135	1.0792350433
S	-2.6238344326	-5.2865260614	-0.2552711214
C	-2.3720148055	-4.1786404774	-1.6048611124
C	-1.7018084934	-2.9418340081	-1.4899057563
C	1.3844036573	3.3152178920	0.9306581510
C	1.9683354634	4.5958785119	1.0407402618
S	2.7650847094	5.4278551421	-0.2959713580
C	2.5177369827	4.3067934189	-1.6355068069
C	1.8438808464	3.0728731651	-1.5113675927
C	3.0335772565	4.7143601496	-2.8691926846
C	2.8942883974	3.9249604456	-4.0109312470
C	2.2108085190	2.7168324863	-3.9074390367
C	1.6908698301	2.3036365617	-2.6796672544
C	0.8475287502	2.7507002129	2.1022513586
C	0.8559249021	3.4327449871	3.3200209217
C	1.4094275412	4.7069466194	3.4084073358
C	1.9671339291	5.2748116400	2.2626418350
C	-0.7393096098	-2.5730163620	2.1283588782
C	-0.7580402875	-3.2395926808	3.3545568442
C	-1.3093083850	-4.5139778544	3.4536299873
C	-1.8542764790	-5.0977390025	2.3097241040
C	-2.8785938144	-4.6010798639	-2.8373907837
C	-2.7327102723	-3.8244437642	-3.9870272826
C	-2.0524323438	-2.6137904289	-3.8924118880
C	-1.5423126033	-2.1854499946	-2.6656996530
H	-1.2995313131	2.1552977256	-0.3109791979
H	-2.4199863319	-0.0843297165	-0.2661040538
H	1.4320757576	-2.0097637369	-0.2758108374
H	2.5523710582	0.2303512810	-0.2482153620

H	3.5559169936	5.6761758740	-2.9256632966
H	3.3130948601	4.2598201338	-4.9644135818
H	2.0736891655	2.0773529804	-4.7850584757
H	1.1568499796	1.3532524157	-2.6281400875
H	0.4088160700	1.7522290308	2.0615674170
H	0.4214857620	2.9515861950	4.2018979552
H	1.4172353891	5.2573133374	4.3537328557
H	2.4219094501	6.2709189584	2.3078742508
H	-0.3025761225	-1.5740487417	2.0792222861
H	-0.3334672519	-2.7462185746	4.2344856214
H	-1.3250302562	-5.0523844888	4.4057214658
H	-2.3070656015	-6.0943391641	2.3631082117
H	-3.3984684950	-5.5646274837	-2.8866529158
H	-3.1438588933	-4.1710840308	-4.9396325885
H	-1.9101574817	-1.9841078134	-4.7762757418
H	-1.0106850963	-1.2333600809	-2.6213135244

14(c)
70
-1.4979615240717069e+03 frame 32 xyz file generated by TeraChem

C	-0.7270525655	1.1967192192	-0.3807478085
C	-1.3482461946	-0.0508669006	-0.3496586924
C	-0.5786134471	-1.2196670764	-0.3526066913
C	0.8178524685	-1.1275463997	-0.3605245710
C	1.4388259157	0.1203348444	-0.3393423435
C	0.6693068706	1.2890182072	-0.3625050672
N	1.3025127194	2.5670895194	-0.3693222212
N	-1.2116141549	-2.4978911316	-0.3490515890
C	-1.2261970989	-3.2501554906	0.8325916538
C	-1.8145964356	-4.5325979073	0.8649680889
C	-2.5061074355	-5.1558086912	-0.3466480484
C	-2.4424922764	-4.2283376871	-1.5596608036
C	-1.7947923900	-2.9749934452	-1.5302299030
C	1.3068168456	3.3341844469	0.8028369859
C	1.8959215027	4.6165461991	0.8244535057
C	2.5986261097	5.2241085090	-0.3886165712
C	2.5442107076	4.2820756631	-1.5907947782
C	1.8954983165	3.0295471499	-1.5514211600
C	3.1434046679	4.6761016614	-2.7953754974
C	3.1120803769	3.8899555302	-3.9454736825
C	2.4434207353	2.6665758641	-3.9018569822
C	1.8394272674	2.2445275747	-2.7224193736
C	0.7274174929	2.8216851687	1.9827597821
C	0.6941824327	3.5716465028	3.1533047660
C	1.2486752686	4.8514971988	3.1793745966
C	1.8442522546	5.3456248650	2.0205347338
C	-0.6576668484	-2.7226186890	2.0111904716
C	-0.6341044801	-3.4580745879	3.1911377678

C	-1.1878258232	-4.7380074527	3.2281623227
C	-1.7729088541	-5.2468510299	2.0703551202
C	-3.0315619513	-4.6374117727	-2.7642078852
C	-2.9914628305	-3.8652129567	-3.9234374512
C	-2.3239806368	-2.6408834815	-3.8892144787
C	-1.7297521279	-2.2041745295	-2.7101488274
C	1.9088279011	6.5530964756	-0.7651148633
C	4.0729160183	5.5007657445	-0.0246969281
C	-1.8110328037	-6.4880339661	-0.7010175739
C	-3.9830616344	-5.4300409139	0.0079511116
H	-1.3247611407	2.1130728329	-0.3995373951
H	-2.4398162049	-0.1267637229	-0.3434256905
H	1.4155284107	-2.0440991844	-0.3632240934
H	2.5303134163	0.1963458511	-0.3250166237
H	3.6549232467	5.6431471557	-2.8354000819
H	3.5975580942	4.2335349480	-4.8640777553
H	2.3868173549	2.0286775429	-4.7902050893
H	1.3173171123	1.2860452939	-2.7076742487
H	0.2937787217	1.8199243750	1.9811529220
H	0.2300186376	3.1459143401	4.0491640113
H	1.2257718351	5.4565050467	4.0909596719
H	2.2927964223	6.3439838320	2.0459297287
H	-0.2246528279	-1.7206379106	2.0010075072
H	-0.1781427895	-3.0209307436	4.0857119829
H	-1.1723513510	-5.3317327394	4.1472886699
H	-2.2210891036	-6.2451137852	2.1038398534
H	-3.5419492382	-5.6053281949	-2.7966287355
H	-3.4691773643	-4.2202616944	-4.8417480192
H	-2.2605924248	-2.0137405407	-4.7847331966
H	-1.2083811358	-1.2452008784	-2.7028370074
H	0.8531641063	6.3852153245	-1.0334515536
H	2.4117926948	7.0255515220	-1.6241298960
H	1.9416815868	7.2665273102	0.0742860686
H	4.5931296689	4.5668045947	0.2433538667
H	4.1436961064	6.1907566903	0.8316898046
H	4.6087754296	5.9629373794	-0.8696070815
H	-0.7538292715	-6.3213284799	-0.9640090728
H	-2.3066688884	-6.9729555692	-1.5573907606
H	-1.8480094902	-7.1908640683	0.1471650843
H	-4.5069021884	-4.4934754403	0.2593553178
H	-4.0604828094	-6.1090257305	0.8725299037
H	-4.5108826157	-5.9039219930	-0.8355352716

8. References

(1) Ufimtsev, I. S.; Martinez, T. J. Quantum Chemistry on Graphical Processing Units. 3. Analytical Energy Gradients, Geometry Optimization, and First Principles Molecular Dynamics. *Journal of Chemical Theory and Computation* **2009**, *5* (10), 2619-2628.

(2) Skhirtladze, L.; Keruckiene, R.; Bezvikonnyi, O.; Mahmoudi, M.; Volyniuk, D.; Leitonas, K.; Ghasemi, M.; Simokaitiene, J.; Nasir, F. H. A.; Ariffin, A.; et al. Switching thermally activated delayed fluorescence to room temperature phosphorescence for oxygen sensing: Effect of donor substituents of trifluoromethylphenyl. *Spectrochimica Acta Part A: Molecular and Biomolecular Spectroscopy* **2024**, *306*, 123531.

(3) Skhirtladze, L.; Leitonas, K.; Bucinskas, A.; Woon, K. L.; Volyniuk, D.; Keruckienė, R.; Mahmoudi, M.; Lapkowski, M.; Ariffin, A.; Grazulevicius, J. V. Turn on of room temperature phosphorescence of donor-acceptor-donor type compounds *via* transformation of excited states by rigid hosts for oxygen sensing. *Sensors and Actuators B: Chemical* **2023**, *380*, 133295.

(4) Skhirtladze, L.; Lietonas, K.; Bucinskas, A.; Volyniuk, D.; Mahmoudi, M.; Mukbaniani, O.; Woon, K. L.; Ariffin, A.; Grazulevicius, J. V. 1,4-Bis(trifluoromethyl)benzene as a new acceptor for the design and synthesis of emitters exhibiting efficient thermally activated delayed fluorescence and electroluminescence: experimental and computational guidance. *Journal of Materials Chemistry C* **2022**, *10* (12), 4929-4940.

(5) Pang, J.; Di, Z.; Qin, J.-S.; Yuan, S.; Lollar, C. T.; Li, J.; Zhang, P.; Wu, M.; Yuan, D.; Hong, M.; et al. Precisely Embedding Active Sites into a Mesoporous Zr-Framework through Linker Installation for High-Efficiency Photocatalysis. *Journal of the American Chemical Society* **2020**, *142* (35), 15020-15026.

(6) Devic, T.; Horcajada, P.; Serre, C.; Salles, F.; Maurin, G.; Moulin, B.; Heurtaux, D.; Clet, G.; Vimont, A.; Grenèche, J.-M.; et al. Functionalization in Flexible Porous Solids: Effects on the Pore Opening and the Host−Guest Interactions. *Journal of the American Chemical Society* **2010**, *132* (3), 1127-1136.

(7) Wang, Y.; Zhang, W.; Yang, J.; Gong, Y.; Zhang, J.; Fang, M.; Yang, Q.-H.; Li, Z. The key role of molecular aggregation in rechargeable organic cathodes. *Matter* **2022**, *5* (12), 4467-4479.

(8) Kim, S.-K.; Lee, J.-H.; Park, J.-W. Phenyl-Naphthyl Amine Effect of New Phenothiazine Derivatives with High T g for Hole Injection and Hole Transporting Materials. *Journal of Nanoscience and Nanotechnology* **2008**, *8* (10), 5247-5251.

(9) Kothavale, S.; Chung, W. J.; Lee, J. Y. Rational Molecular Design of Highly Efficient Yellow-Red Thermally Activated Delayed Fluorescent Emitters: A Combined Effect of Auxiliary Fluorine and Rigidified Acceptor Unit. *ACS Applied Materials & Interfaces* **2020**, *12* (16), 18730-18738.

(10) Huang, T.; Liu, D.; Li, D.; Jiang, W.; Jiang, J. Novel yellow thermally activated delayed fluorescence emitters for highly efficient full-TADF WOLEDs with low driving voltages and remarkable color stability. *New Journal of Chemistry* **2019**, *43* (34), 13339-13348.

(11) Niladari Raju, M. V.; Mohanty, M. E.; Bangal, P. R.; Vaidya, J. R. Synthesis and Ultrafast Dynamics of a Donor–Acceptor–Donor Molecule Having Optoelectronic Properties. *The Journal of Physical Chemistry C* **2015**, *119* (16), 8563-8575.

(12) Woon, K. L.; Nadiah, Z. N.; Hasan, Z. A.; Ariffin, A.; Chen, S.-A. Tuning the singlet-triplet energy splitting by fluorination at 3,6 positions of the 1,4-biscarbazoylbenzene. *Dyes and Pigments* **2016**, *132*, 1-6.

(13) Schuster, C.; Börger, C.; Julich-Gruner, K. K.; Hesse, R.; Jäger, A.; Kaufmann, G.; Schmidt, A. W.; Knölker, H.-J. Synthesis of 2-Hydroxy-7-methylcarbazole, Glycozolicine, Mukoline, Mukolidine, Sansoakamine, Clausine-H, and Clausine-K and Structural Revision of Clausine-TY. *European Journal of Organic Chemistry* **2014**, *2014* (22), 4741-4752.
(14) Liu, H.; Li, J.; Li, G.; Zhang, B.; Zhan, Q.; Liu, Z.; Zhou, C.; Li, K.; Wang, Z.; Yang, C. A simple strategy to achieve efficient thermally activated delayed fluorescent emitters *via* enhancing electron donating ability of donors. *Dyes and Pigments* **2020**, *180*, 108521.
